# High-Fidelity Sequence-Selective Duplex Formation by Recognition-Encoded Melamine Oligomers

**DOI:** 10.1021/jacs.1c02275

**Published:** 2021-06-03

**Authors:** Pavle Troselj, Peter Bolgar, Pablo Ballester, Christopher A. Hunter

**Affiliations:** † Yusuf Hamied Department of Chemistry, University of Cambridge, Lensfield Road, Cambridge CB2 1EW, U.K.; ∥ Institute of Chemical Research of Catalonia (ICIQ), Av. Països Catalans 16, 43007 Tarragona, Spain; § Catalan Institution for Research and Advanced Studies (ICREA), Pg. Lluís Companys 23, 08010 Barcelona, Spain

## Abstract

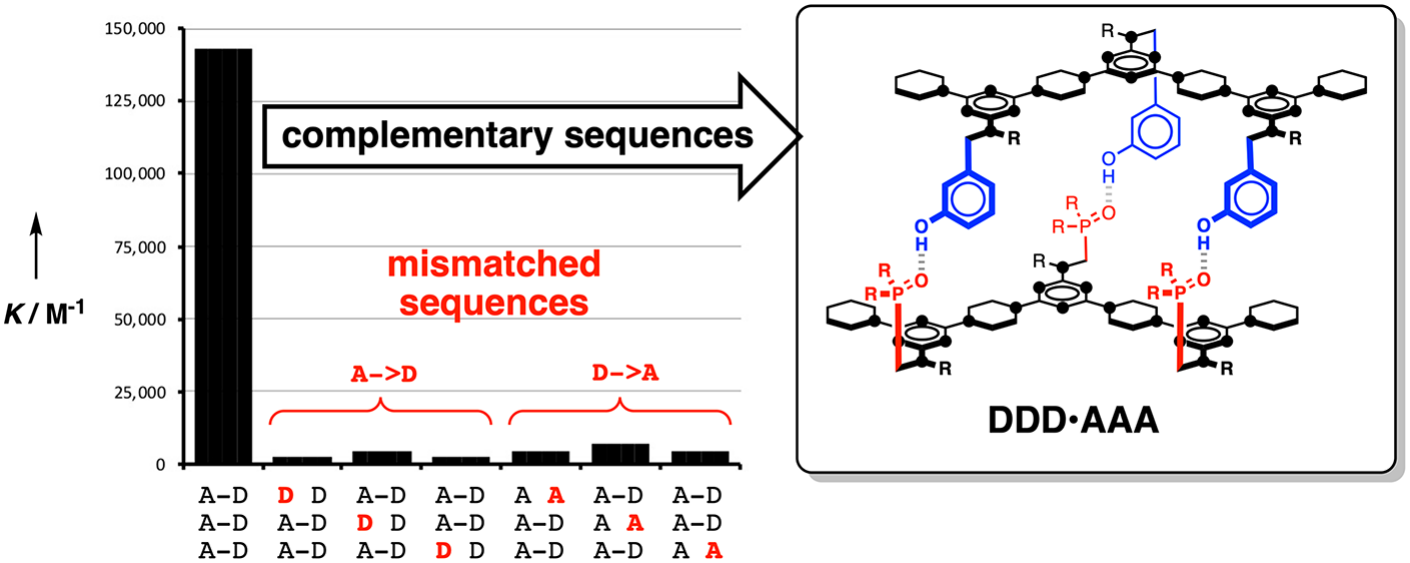

Melamine oligomers composed of repeating triazine-piperidine units and equipped with phenol and phosphine oxide side-chains form H-bonded duplexes. The melamine backbone provides sufficient rigidity to prevent intramolecular folding of oligomers up to three recognition units in length, leading to reliable duplex formation between sequence complementary oligomers. NMR spectroscopy and isothermal titration calorimetry (ITC) were used to characterize the self-assembly properties of the oligomers. For length-complementary homo-oligomers, duplex formation in toluene is characterized by an increase in stability of an order of magnitude for every base-pair added to the chain. NMR spectra of dilute solutions of the **AD** 2-mer show that intramolecular H-bonding between neighboring recognition units on the chain (1,2-folding) does not occur. NMR spectra of dilute solutions of both the **AAD** and the **ADD** 3-mer show that 1,3-folding does not take place either. ITC was used to characterize interactions between all pairwise combinations of the six different 3-mer sequences, and the sequence complementary duplexes are approximately an order of magnitude more stable than duplexes with a single base mismatch. High-fidelity duplex formation combined with the synthetic accessibility of the monomer building blocks makes these systems attractive targets for further investigation.

## Introduction

The molecular nanotechnology found in nature is based on linear oligomers, where function is encoded by the sequence of monomer building blocks. The solid-phase methods originally developed to make synthetic polypeptides and oligonucleotides have also been used to prepare nonbiological oligomers with a defined sequence of different monomer building blocks.^1−9^ However, these synthetic oligomers are largely devoid of the kind of functional properties found in biopolymers. The properties of polypeptides are determined by the folded three-dimensional structure, which is in turn encoded by sequence. Although progress has been made in the prediction of folding patterns based on sequence, prediction of the function associated with a particular folded structure remains a major challenge in protein chemistry.^10,11^ We have therefore focused our attention on motifs inspired by nucleic acids. The ladder structure found in the DNA double helix is a relatively straightforward supramolecular target. Moreover, this structure is intrinsically linked to function and forms the basis for the template-directed synthesis used in molecular replication, transcription, and translation in biological systems.^12^ The synthesis of oligomers that form duplexes has turned out to be a tractable problem,^13−46^ and we have found that some of these compounds also fold and catalyze reactions in a manner reminiscent of nascent RNA function.^47,48^

We have described a number of noncovalent base-pairing systems that have been used for duplex formation between sequence-complementary oligomers via H-bonding interactions.^41−46^ We have also used a covalent base-pairing system for replication of sequence information from a parent template oligomer to a daughter copy strand.^49^ The main factor which limits the sequence selectivity achieved in both these systems is related to the conformational flexibility of the backbone. Here we describe a new class of oligomers with a more rigid backbone that leads to high-fidelity sequence-selective duplex formation using a noncovalent base-pairing system.

**Figure 1:**
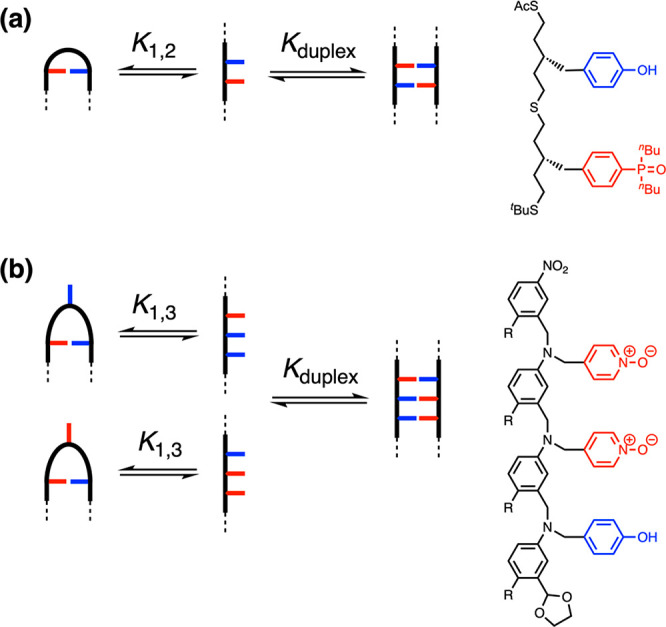
Duplex-forming oligomers. Intramolecular folding lowers duplex stability and reduces sequence-selectivity: (a) 1,2-folding on a polythioether backbone^50^ and (b) 1,3-folding on a polyaniline backbone.^43^

Figure 1 illustrates our approach to duplex-forming oligomers.^41^ The base-pairing system uses formation of a single H-bond between complementary donor (blue) and acceptor (red) side-chains attached to a nonpolar backbone. In nonpolar solvents, H-bonding interactions between the recognition units shown in Figure 1 are strong enough to ensure that the stability of the duplex increases by about an order of magnitude for every base-pair formed. The use of a single H-bond as the base-pairing motif minimizes the chances of mispairing, and the lack of polar groups on the backbone avoids any competitive H-bond equilibria. Length-complementary homo-oligomers form stable duplexes in predicable manner for a wide variety of different backbones.^41−46^ In contrast, duplex formation between the hetero-oligomers can be compromised by the competing intramolecular folding equilibria illustrated in Figure 1.^50^ The flexibility of the polythioether backbone shown in Figure 1a leads to interactions between neighboring recognition units (1,2-folding). The polyaniline backbone in Figure 1b is rigid enough to prevent 1,2-folding, but 1,3-folding significantly reduces the fidelity of sequence-selective duplex formation in these systems. Here we describe a new backbone that is sufficiently rigid to prevent 1,3-folding, leading to high-fidelity duplex formation between mixed-sequence 3-mers.

**Figure 2:**
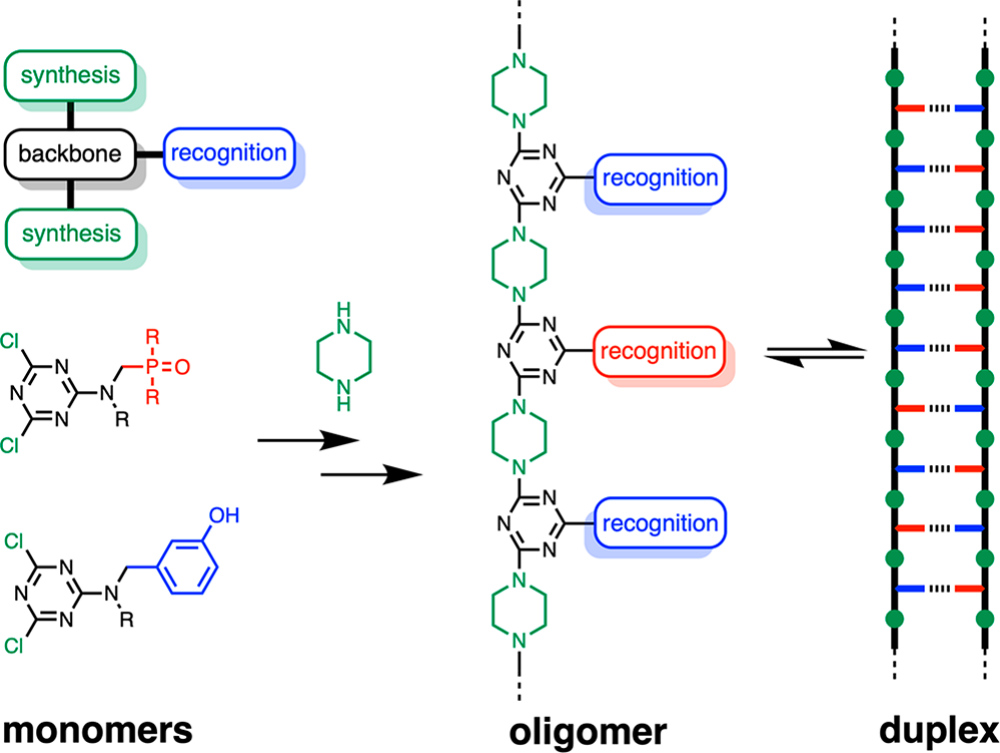
A blueprint for duplex-forming molecules. There are three key structural elements: recognition modules for base-pair formation (red and blue), functionality used for the synthesis of oligomers (green), and the backbone module which connects the three components of a monomer. Implementation in melamine oligomers equipped with a phenol·phosphine oxide base-pairing system is illustrated (R are solubilizing groups).

**Figure 3:**
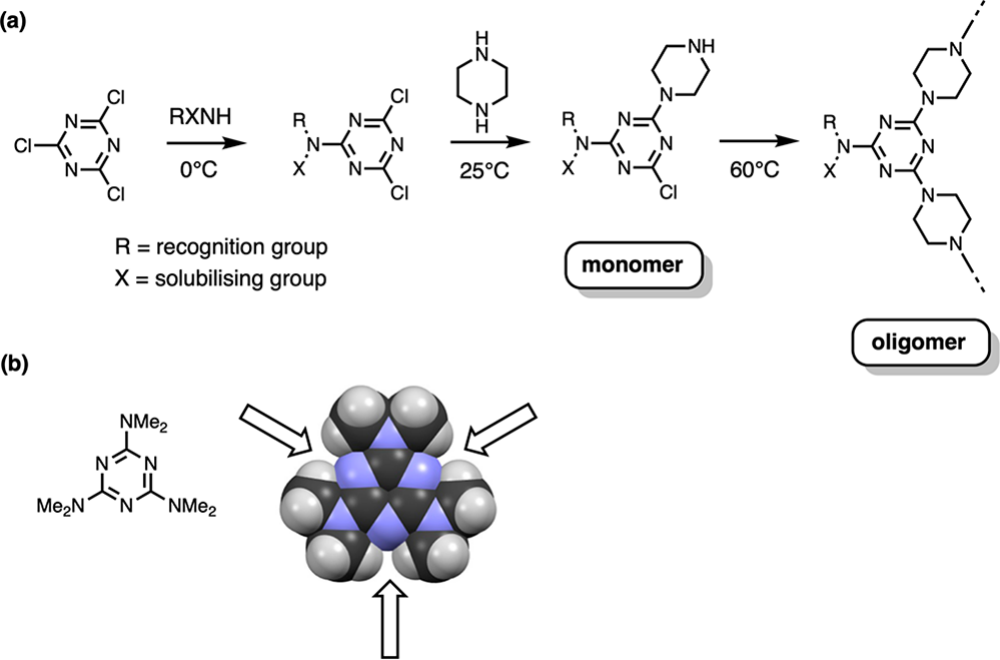
(a) Stepwise substitution of cyanuric chloride to give monomers required for the formation of melamine oligomers. (b) The X-ray crystal structure of 2,4,6-tris(dimethylamino)-1,3,5-triazine (CCDC ref code HMELAM) shows that access to the nitrogen lone pairs is sterically blocked in peralkylated melamines.

The approach to a rigid recognition-encoded melamine oligomer is illustrated in Figure 2. The monomer building blocks required for synthesis of oligomers must be equipped with a recognition unit and two reactive sites that allow polymerization. The oligomer in Figure 2 is an attractive target, because cyanuric chloride provides straightforward synthetic access to trifunctional monomers.^51−54^ Sequential temperature-controlled nucleophilic aromatic substitution reactions with secondary amines allow the stepwise functionalization of the triazine core in high yield (Figure 3a). Although the melamine backbone in Figure 2 appears to be relatively polar, the use of secondary amines ensures that the triazine nitrogen atoms are all sterically blocked from acting as H-bond acceptors (Figure 3b). The backbone is likely to adopt an extended conformation, because the piperazine linker has a strong preference for the chair conformation. A search of the Cambridge Structural Database found 79 crystal structures of nonmacrocyclic compounds which contain a piperazine linker where both nitrogen atoms are functionalized with sp^2^ carbons and where there is no disorder. The piperazine never adopts the boat conformation; the chair conformation is found in 77 of the 79 structures, and there are only two examples of a twist boat conformation.

## Results and Discussion

### Synthesis

**Scheme 1:**
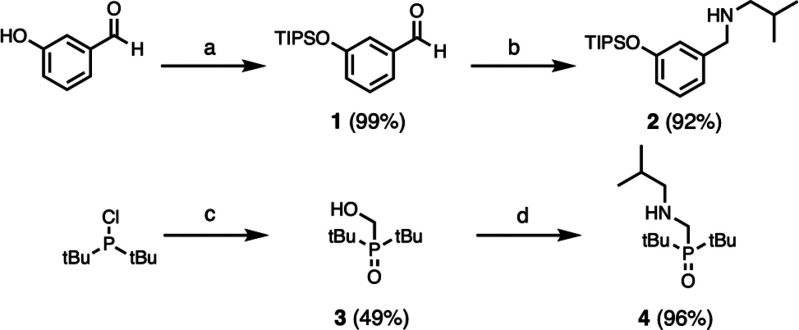
Synthesis of **2** and **4**: (a) TIPSCl, imidazole; (b) 1. *i*-butylamine, 2. NaBH_4_; (c) H_2_CO, HCl; (d) 1. MsCl, 2. *i*-butylamine

The H-bond donor and acceptor building blocks were synthesized from secondary amines **2** and **4**, which have isobutyl solubilizing groups. Secondary amine **2** was obtained by protection of 3-hydroxybenzaldehyde using triisopropylsilyl chloride, followed by reductive amination with isobutylamine (Scheme 1). Secondary amine **4** was prepared from di-*tert*-butylchlorophosphine, which was treated with formaldehyde in strong aqueous acid to give phosphine oxide **3**. Reaction of **3** with mesyl chloride, followed by microwave-assisted nucleophilic substitution with isobutylamine gave **4** (Scheme 1).

**Scheme 2:**
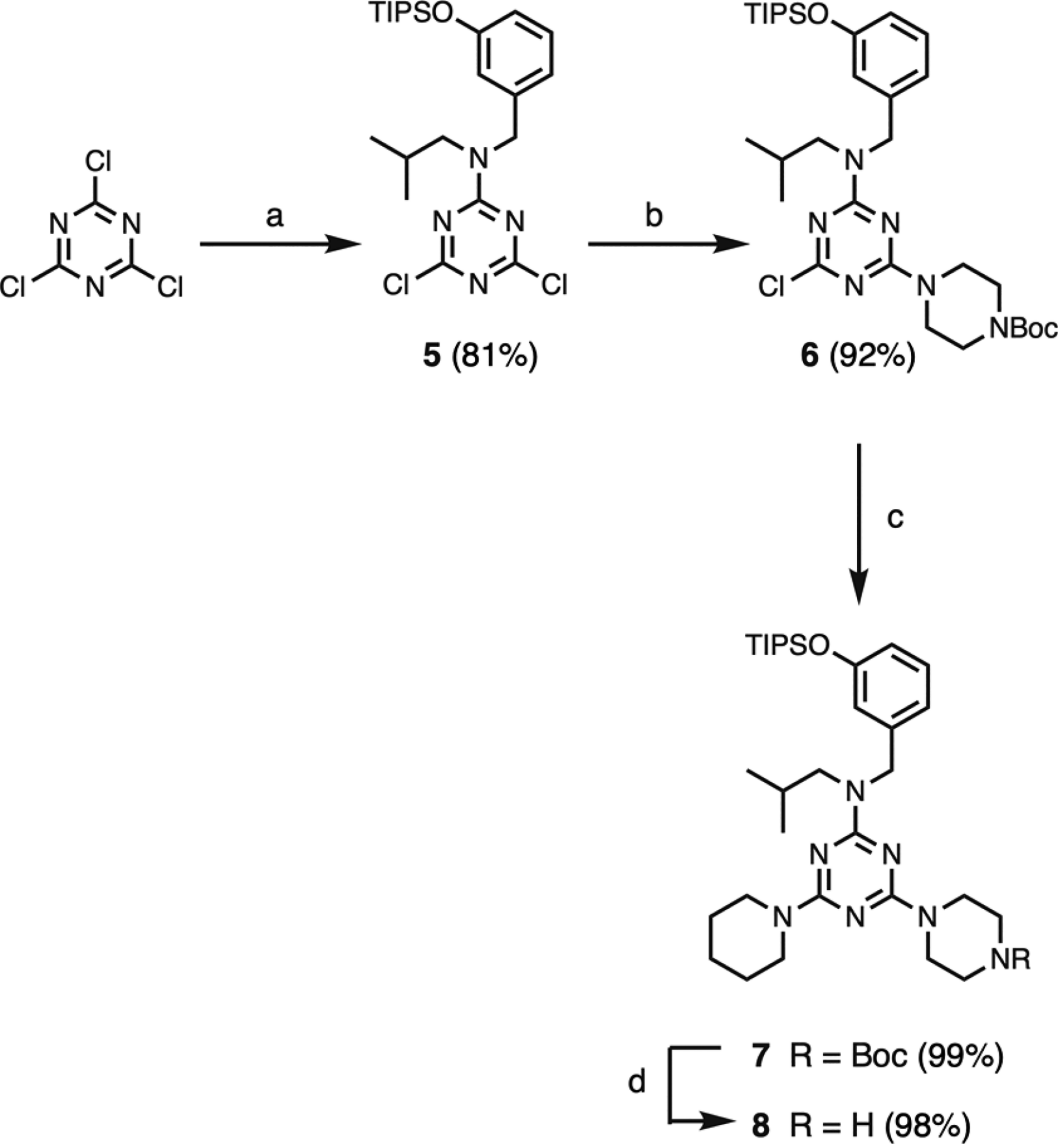
Synthesis of H-Bond Donor Monomers **5** and **8**: (a) **2**, DIPEA; (b) 1-Boc piperazine, DIPEA; (c) piperidine; (d) TFA

Scheme 2 shows the synthesis of two different H-bond donor monomers. Monomer **5** has two reactive chlorotriazine sites for coupling with amine groups and was used to build up oligomer chains. Monomer **8** has a single reactive amine group and was used to terminate the chain end of an oligomer. Cyanuric chloride was reacted with secondary amine **2** in the presence of DIPEA at −10 °C to give **5**. A second nucleophilic aromatic substitution with 1-Boc piperazine in the presence of DIPEA at 25 °C gave **6**, which was then heated with excess piperidine in refluxing THF to afford **7**. Deprotection with TFA gave **8**.

**Scheme 3:**
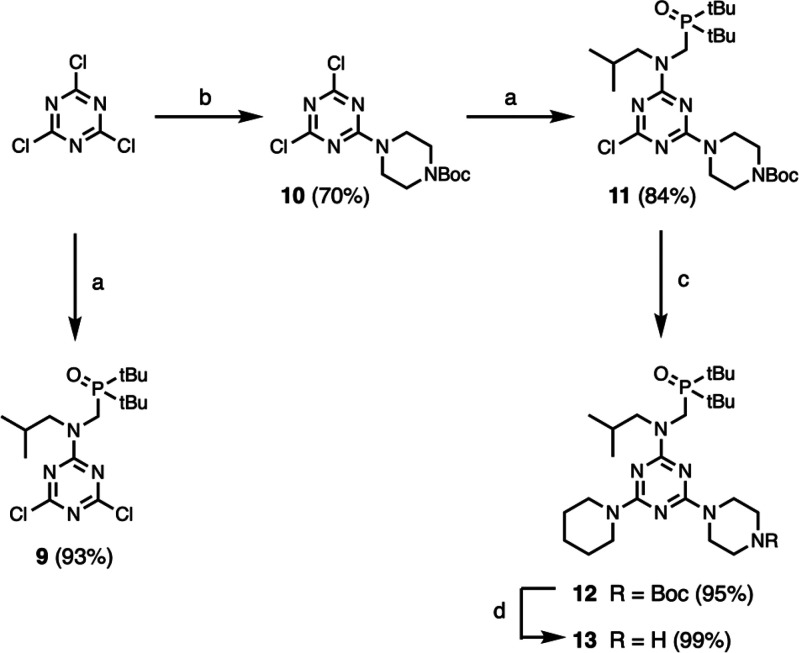
Synthesis of H-Bond Acceptor Monomers **9** and **13**: (a) **4**, DIPEA; (b) 1-Boc piperazine, DIPEA; (c) piperidine; (d) TFA

A similar strategy was used to obtain the corresponding H-bond acceptor monomers **9** and **13** (Scheme 3). Reaction of cyanuric chloride with secondary amine **4** in the presence of DIPEA at −10 °C gave **9**. Successive nucleophilic aromatic substitutions of cyanuric chloride with 1-Boc piperazine at −10 °C, secondary amine **4** at 25 °C, and piperidine at 66 °C gave **12**. Deprotection with TFA gave **13**.

**Scheme 4:**
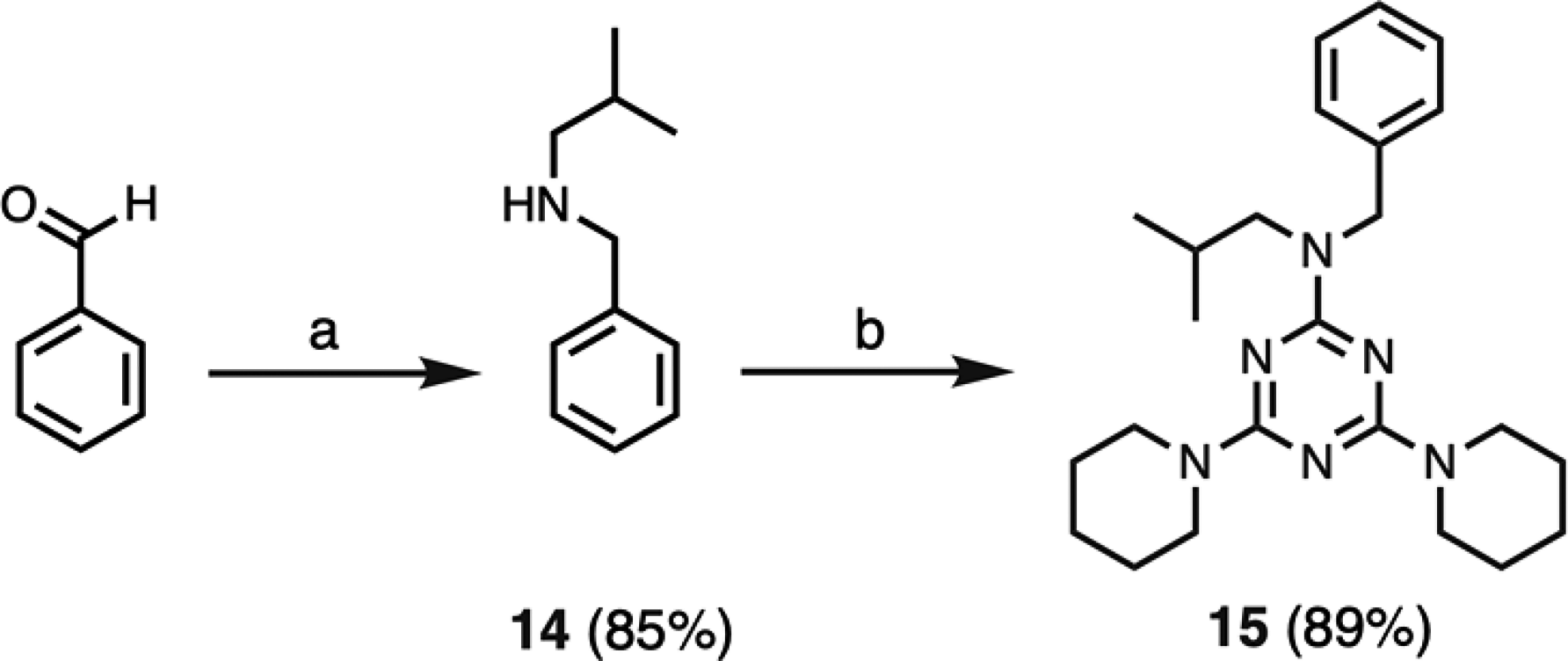
Synthesis of **15**: (a) 1. Isobutylamine, 2. NaBH_4_; (b) 1. Cyanuric Chloride, DIPEA, 2. Piperidine

Melamine derivative **15**, which has a benzyl side-chain in place of the recognition units present in the other monomers, was synthesized as a reference compound to investigate the H-bonding properties of the backbone (Scheme 4). Secondary amine **14** was prepared from benzaldehyde and isobutylamine by reductive amination and reaction with cyanuric chloride at −10 °C, followed by refluxing with piperidine in THF gave **15**.

**Scheme 5:**
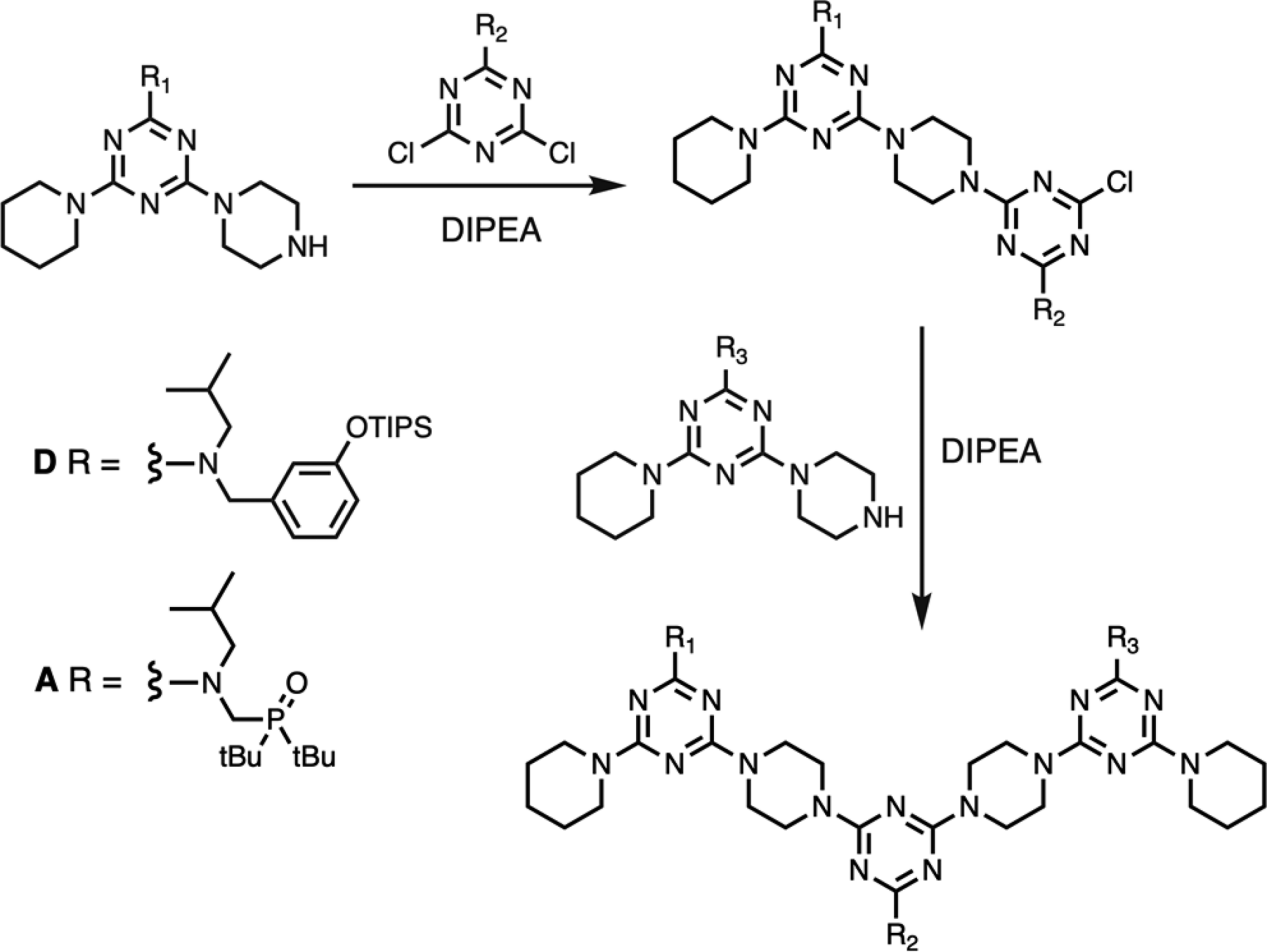
Synthetic Strategy for the Synthesis of a Mixed-Sequence 3-mer, Where R_1_, R_2_, and R_3_ Represent Different Combinations of the **D** and **A** Side Chains

A family of 13 recognition-encoded melamine oligomers of different length and sequence were synthesized as outlined in the Supporting Information. The 1-mers **D** and **A** were synthesized directly from **5** or **9** by reaction with piperidine in refluxing THF. Longer oligomers were synthesized using different combinations of the monomer building blocks **5**, **8**, **9**, and **13**, and Scheme 5 illustrates the general approach for the synthesis of mixed-sequence oligomers. Sequential piperazine-chlorotriazine coupling reactions were carried using DIPEA, and after assembly of the oligomer, any TIPS protecting groups present on phenol side chains were removed using TBAF.

### Duplex Formation by Homosequence Oligomers

NMR titration experiments were used to investigate the binding properties of the different components of the melamine oligomers in toluene. Addition of 1-mer **D** to 1-mer **A** led to large changes in the ^31^P NMR chemical shift of the signal due to the phosphine oxide group. The titration data fit well to a 1:1 binding isotherm with an association constant of 361 ± 9 M^–1^. The limiting complexation-induced change in ^31^P NMR chemical shift was 5.3 ppm, which is characteristic of formation of a phenol·phosphine oxide H-bond.^41−46^ In contrast, titration of melamine derivative **15** into *m*-cresol in toluene resulted in small changes in ^1^H NMR chemical shift, confirming that the alkyl substituents effectively shield the melamine nitrogen H-bond acceptor sites on the backbone, as suggested by the X-ray crystal structure shown in Figure 3b.

**Figure 4:**
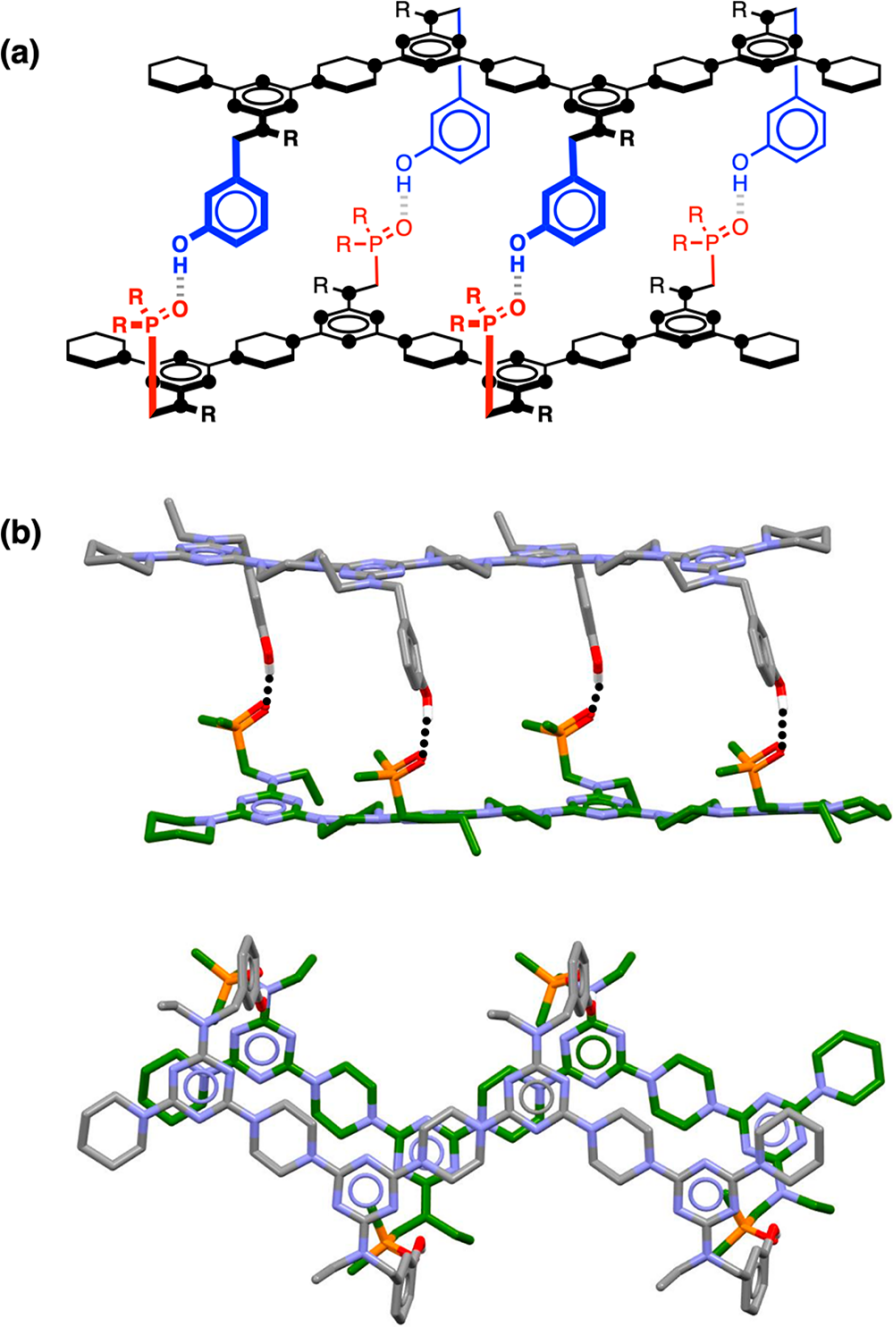
(a) Structure of the **AAAA**·**DDDD** duplex. The melamine nitrogen atoms are represented as black dots, and R’s are the solubilizing groups. (b) Side and top views of the energy minimum structure of the **AAAA**·**DDDD** duplex (MMFFs with chloroform solvation). H-bonds are indicated by black dotted lines. The solubilizing groups and hydrogen atoms are omitted for clarity.

Interactions between length complementary homo-oligomers were investigated using isothermal titration calorimetry (ITC) in toluene. The data fit well to a 1:1 binding isotherm in all cases, and the results are summarized in Table 1. The complex formed by **AA** and **DD** is an order of magnitude more stable than the **A**·**D** complex, which indicates cooperative formation of two intermolecular H-bonds in the **AA**·**DD** complex. There is a significant increase in the stability of the complex for every recognition unit added to the oligomer, which shows that cooperative H-bonding interactions propagate with increasing chain length. In other words, the duplexes are fully assembled with H-bonds between each of the complementary recognition groups (Figure 4). Table 1 shows that there are compensating increases in the magnitudes of the enthalpy and entropy changes for duplex formation as the number of H-bonding interactions increases.

**Table 1:** Association Constants for Duplex Formation between Length-Complementary Homo-oligomers Measured by ITC Titration Experiments in Toluene at 298 K^a^

complex	log *K* (M^–1^)	*ΔG*° (kJ mol^–1^)	*ΔH*° (kJ mol^–1^)	*ΔS*° (J K^–1^ mol^–1^)
**A·D**^b^	2.6	–14.6		
**AA·DD**	3.7	–20.9	–29	–27
**AAA·DDD**	5.2	–29.5	–57	–92
**AAAA·DDDD**	5.9	–33.8	–92	–195

a Errors based on repeat experiments are 0.1 in log *K*, 0.5 kJ mol^–1^ in Δ*G*°, 5 kJ mol^–1^ in Δ*H*°, and 20 J K^–1^ mol^–1^ in Δ*S*°.

b Measured by ^31^P NMR titration.

**Figure 5:**
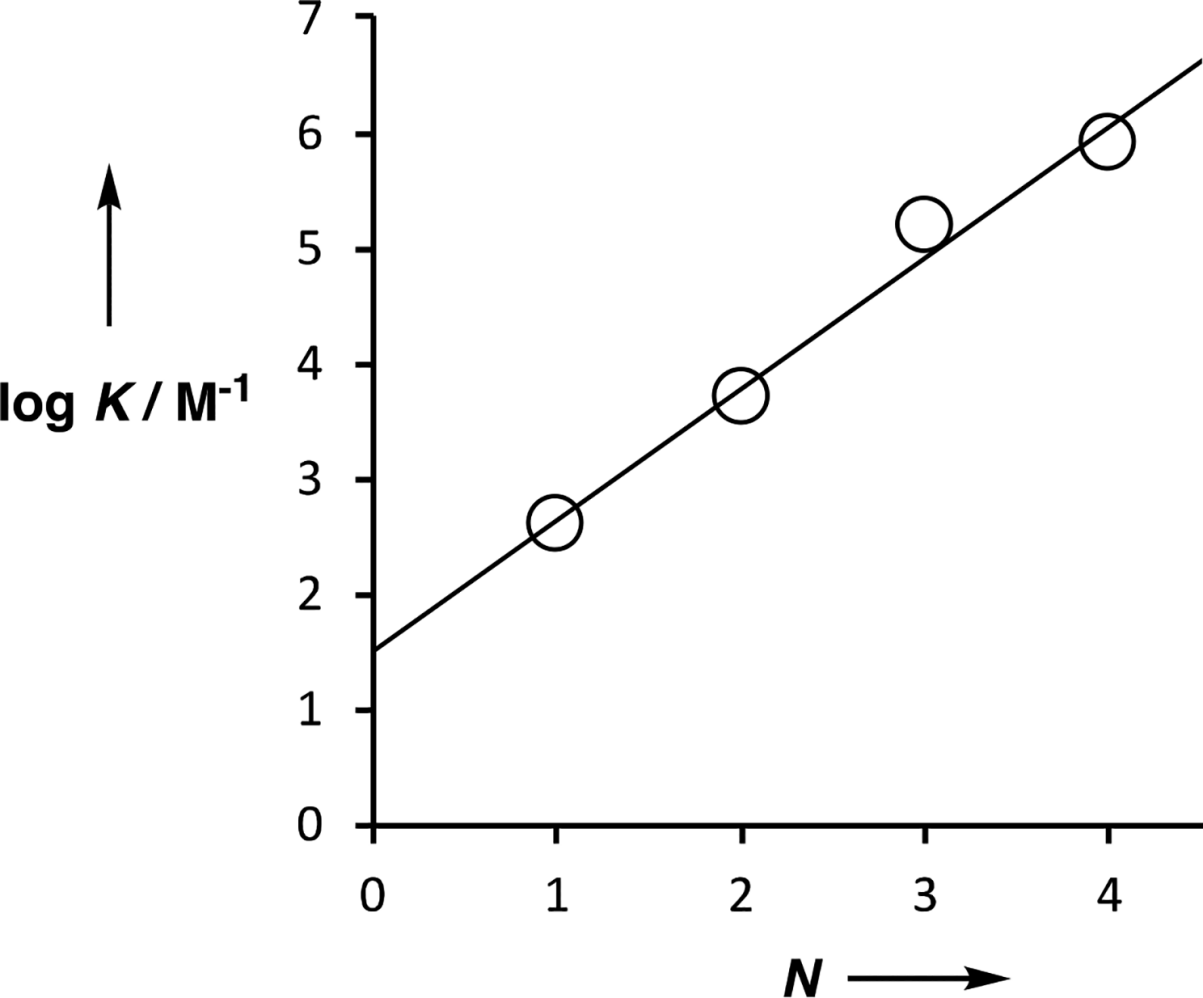
Relationship between the association constants for duplex formation between length-complementary homo-oligomers measured in toluene at 298 K (*K*) and the number of intermolecular H-bonds formed (*N*). The line of best fit shown is log *K* = 1.1*N* + 1.5.

Figure 5 shows the relationship between the association constant for duplex formation (log *K*) and the number of base-pairs (*N*). The linear relationship indicates that the stepwise effective molarities for sequential formation of each intramolecular H-bond in the duplex (EM) are approximately constant. The overall stability of the duplex is related to EM by eq 1, so the slope of the line of best fit in Figure 5 can be used to estimate the average effective molarity for intramolecular H-bonding in the duplex as 40 mM. This value is similar to the values of EM that we have reported previously for duplex formation by several different kinds of recognition-encoded oligomer.^41−46^
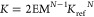
1where *K*_ref_ is the association constant for formation of a single intermolecular H-bond, which is measured using the **A**·**D** complex, and the statistical factor of 2 represents the degeneracy of the duplex.

### Folding of Mixed-Sequence Oligomers

**Figure 6:**
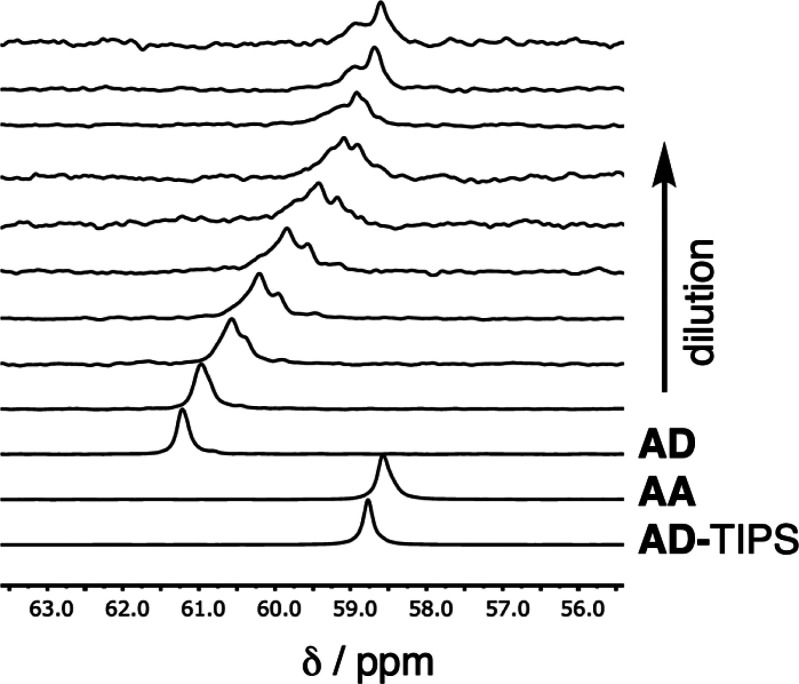
202 MHz ^31^P NMR spectra in CDCl_3_ at 298 K for dilution of the **AD** 2-mer (0.1–100 mM). The corresponding spectra of the TIPS-protected **AD** 2-mer and the **AA** 2-mer are shown for comparison.

The self-assembly properties of mixed-sequence oligomers are generally more complicated, because there are multiple competing equilibria. In contrast to homo-oligomers, hetero-oligomers can fold because of intramolecular interactions between complementary recognition sites on the same molecule, and they can self-associate because of intermolecular interactions between complementary recognition sites on different molecules. The **AD** 2-mer was used to investigate the 1,2-folding process illustrated in Figure 1a. The ^1^H NMR spectra of the melamine oligomers are complicated because of the presence of multiple rotamers, which are in slow exchange on the NMR time scale. Slow exchange rotamers are also apparent in the ^31^P NMR spectrum of **AD**, but there is only one signal, and the weighted average value can be used to simplify the analysis. Figure 6 shows a ^31^P NMR dilution experiment for **AD** in chloroform. At low concentrations of **AD**, the chemical shift is similar to the values recorded for **AA** and for the TIPS-protected **AD** 2-mer, neither of which can form H-bonds. This observation indicates that there are no intramolecular H-bonds in the single stranded monomeric state of **AD**; that is, there is no 1,2-folding. At higher concentrations of **AD**, there is a significant increase in chemical shift, which is characteristic of formation of intermolecular H-bonds. The dilution data fit well to a dimerization isotherm, and Table 2 compares the results with the corresponding values for the **AA**·**DD** duplex measured in a titration experiment in chloroform. The limiting bound chemical shifts of the two complexes are very similar, which indicates that **AD·AD** also forms a duplex with both phosphine oxide groups involved in intermolecular H-bonds. The association constant for formation of the **AD**·**AD** duplex is approximately four times lower than the value for **AA**·**DD**, because there is a 4-fold difference in degeneracy between the two duplexes, which have different symmetry.^50^

**Table 2:** Association Constants and Limiting Chemical Shifts Measured by ^31^P NMR Dilution and Titration Experiments in CDCl_3_ at 298 K^a^

complex	log *K* (M^–1^)	δ_free_(ppm)	δ_bound_(ppm)
**AA·DD**	2.6	58.6	61.6
**AD·AD**	2.0	58.6	61.8
**AAD·AAD**	2.0	58.5	61.1
**ADD·ADD**	2.5	58.5	62.2

a Errors based on repeat experiments are 0.1 in log *K*.

**Figure 7:**
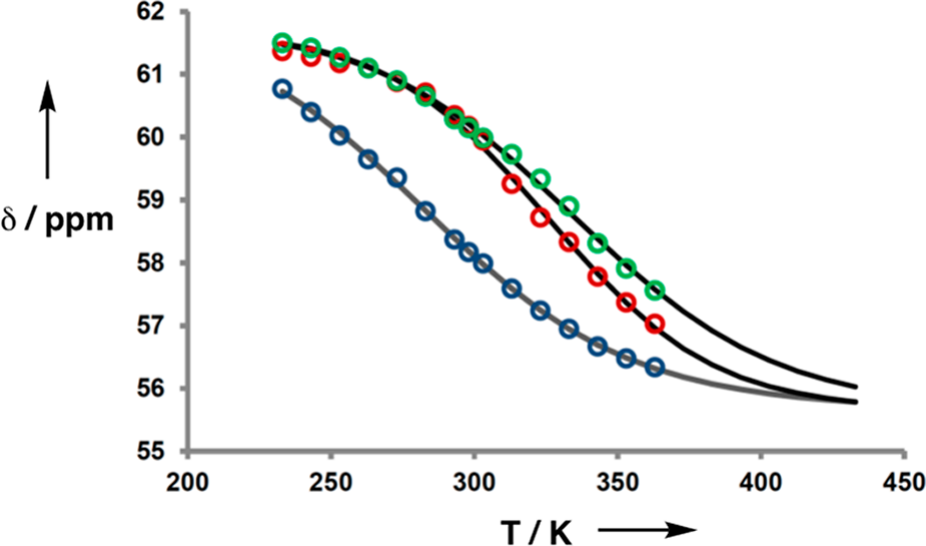
^31^P NMR chemical shifts in *d*_8_-toluene plotted as a function of temperature for the **AD** 2-mer (green), a 1:1 mixture of **AA** and **DD** (red), and a 1:1 mixture of **A** and **D** (blue). The concentration of **AD** was 6 mM, and the concentrations of **AA**, **DD**, **A**, and **D** were 3 mM, so that the concentration of fully bound duplex was the same in all experiments.

The relative stabilities of the duplexes in toluene were investigated using ^31^P NMR melting experiments. The behavior of **AD** is compared with a 1:1 mixture of **AA** and **DD** and with a 1:1 mixture of the 1-mers **A** and **D** in Figure 7. At low temperatures, the ^31^P NMR chemical shifts are all around 61 ppm, which is characteristic of complexes where all of the phosphine oxide groups are fully H-bonded (the limiting bound chemical shift of the **A·D** complex measured at 298 K in titration experiments in toluene was also 61 ppm, see the Supporting Information). At higher temperatures, the chemical shifts decrease toward the value observed for the free phosphine oxide in toluene (56 ppm). Dissociation of the **AD·AD** and **AA·DD** duplexes occurs over the same temperature range, which indicates similar stability. In contrast, the 1:1 mixture of **A** and **D** dissociates at much lower temperatures. These results are consistent with the titration experiments, which indicate the absence of intramolecular H-bonding in the monomeric single-stranded form of **AD** and formation of two H-bonds in the **AD·AD** and **AA·DD** duplexes.

The **AAD** and **ADD** 3-mers were used to investigate the 1,3-folding process illustrated in Figure 1b. The ^31^P NMR spectra of these oligomers are very similar to the spectra recorded for the **AD** 2-mer (see the Supporting Information). At low concentrations, the chemical shifts are similar to the values recorded for the TIPS-protected analogues, which cannot form H-bonds. At higher concentrations, there is a significant increase in the chemical shift of the signal due to the phosphine oxides, which is characteristic of formation of intermolecular H-bonds. The dilution data fit well to a dimerization isotherm in both cases, and the results are reported in Table 2. For both **AAD** and **ADD**, the limiting chemical shift for the single-stranded monomeric state is very similar to the free chemical shifts of **AA** and **AD**. This result indicates that there is no 1,3-folding in these systems. The limiting chemical shifts for the dimeric states indicate the formation of intermolecular H-bonds with the phosphine oxide groups. The values of the association constants for all four complexes in Table 2 are similar, which suggests that they all form duplexes with two intermolecular H-bonds.

### Self-Association of Mixed-Sequence Oligomers

The self-association observed for mixed-sequence oligomers complicates the analysis of duplex formation between sequence complementary oligomers, so the properties of the individual oligomers were characterized first. Association constants for self-association of all of the mixed-sequence oligomers were determined using ITC dilution experiments in toluene. In all cases, the data fit well to a dimerization isotherm, and the results are summarized in Table 3. The **AD** 2-mer and the four mixed-sequence 3-mers have similar self-association constants, which suggests that they all form duplexes with two phenol·phosphine oxide H-bonds. Each of the 3-mers contains the self-complementary **AD** motif, which leads to formation of a two base-pair duplex, with the third base on each strand left unpaired.

**Table 3:** Dimerization Constants Measured by ITC Dilution Experiments in *d*_8_-Toluene at 298 K^a^

sequence	log *K*(M^–1^)	*ΔG°* (kJ mol^–1^)	*ΔH°* (kJ mol^–1^)	*ΔS°* (J K^–1^ mol^–1^)
**AD**	3.2	–18.3	–40	–77
**AAD**	3.1	–17.8	–39	–70
**ADA**	3.5	–20.2	–36	–54
**ADD**	3.3	–19.0	–36	–57
**DAD**	3.4	–19.2	–39	–67

a Errors based on repeat experiments are 0.1 in log *K*, 0.5 kJ mol^–1^ in Δ*G*°, 5 kJ mol^–1^ in Δ*H*°, and 20 J K^–1^ mol^–1^ in Δ*S*°.

### Sequence Selectivity of Duplex Formation

**Figure 8:**
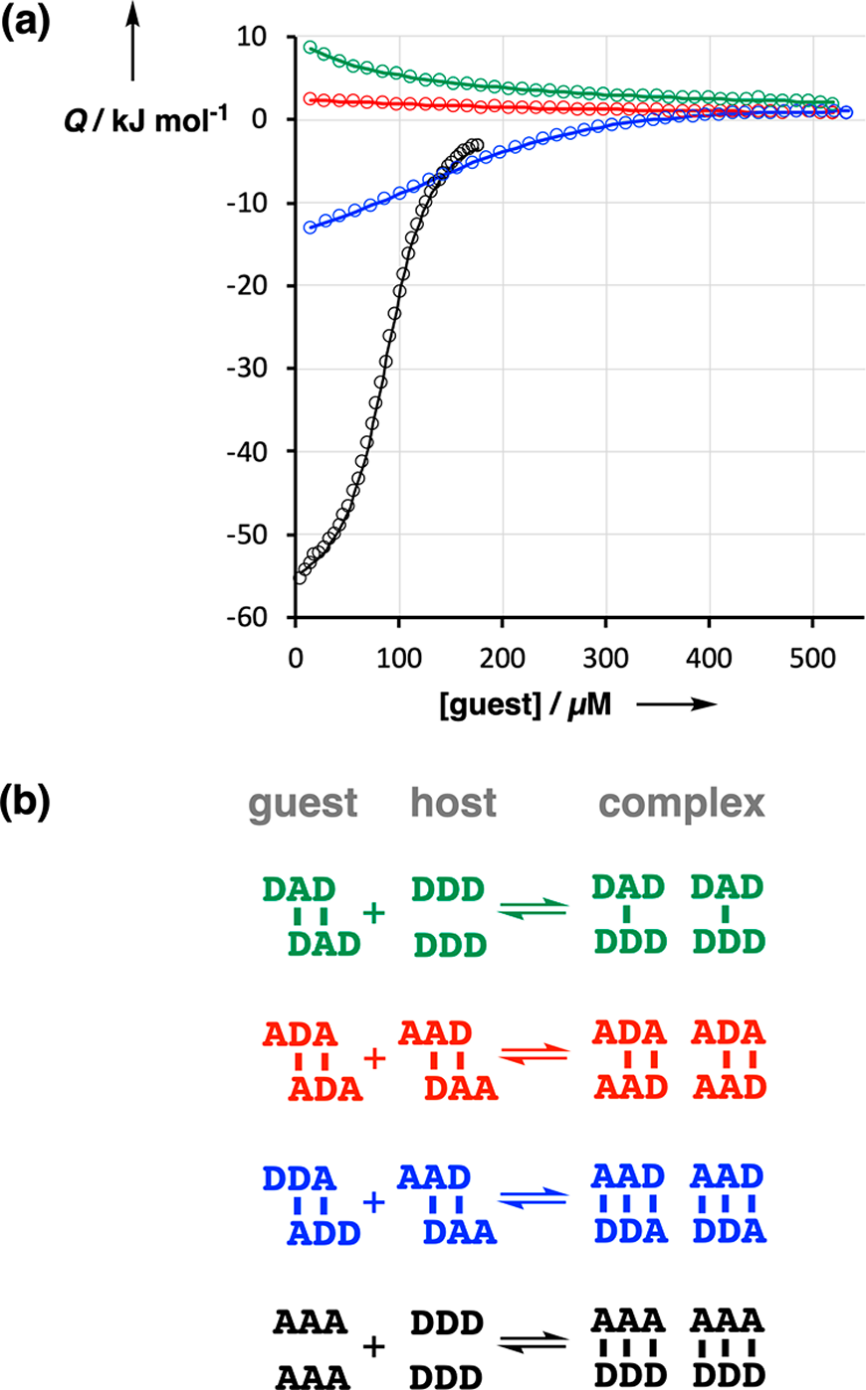
(a) Isothermal titration calorimetry data (circles) for titration of **DAD** into **DDD** (green), **ADA** into **AAD** (red), **ADD** into **AAD** (blue), and **AAA** into **DDD** (black). The lines represent the best fits to an isotherm that allows for formation of a 1:1 complex as well as dimerization of the two components. (b) Structures of the duplexes formed by self-association of the guest and the host used in the ITC experiments, and the structures of the duplexes formed in the mixture (color coding as in panel a).

Interactions between all pairwise combinations of 3-mers were investigated using ITC in toluene. For duplex formation between two mixed-sequence oligomers, self-association of both compounds competes with formation of the 1:1 complex. This leads to four different types of behavior as illustrated in Figure 8.

When two complementary 3-mers that do not self-associate are mixed (**AAA** and **DDD**, black data in Figure 8), duplex assembly leads to formation of three new intermolecular H-bonds, which results in a large release of heat (*Q*).

When two complementary 3-mers that self-associate are mixed (**AAD** and **ADD**, blue data in Figure 8), two intermolecular H-bonds are broken in each of the components, and three intermolecular H-bonds are formed in the new duplex. Thus, there is a net gain of one H-bond per **AAD·ADD** duplex formed, and the amount of heat released is roughly one-third of that observed for **AAA·DDD**.

Even less heat is released when 3-mers that are not complementary are mixed, because the products form less than three H-bonds. For example, **ADA** and **AAD** are noncomplementary 3-mers that both contain the self-complementary **AD** motif. Although these two 3-mers can interact to form a duplex with two H-bonds, both also self-associate to form duplexes with two H-bonds. Thus, there is no net change in H-bonding when these 3-mers are mixed, and the heat change measured by ITC is close to zero (red data in Figure 8).

The other type of behavior observed in the ITC experiments is an endothermic response (green data in Figure 8). When a 3-mer that self-associates is mixed with a noncomplementary 3-mer that does not self-associate, there is a net decrease in the amount of H-bonding. The example shown in Figure 8 is addition of **DAD** to **DDD**. **DAD** self-associates as a duplex with two H-bonds but can form only a singly H-bonded complex with **DDD**, which is not sufficiently stable to be significantly populated at the concentrations used in these experiments.

The titration data were fit to an isotherm that accounts for formation of a 1:1 complex between the two different 3-mers, as well as self-association of the host and the guest (see the Supporting Information).^55^ The thermodynamic parameters for self-association of the individual oligomers were measured separately in the dilution experiments, as reported in Table 3, so there are only two variables that need to be optimized in the fitting procedure, the association constant and the enthalpy change for formation of the new complex. The agreement between the fitted lines and the experimental data points illustrated in Figure 8 is excellent, despite the complexity of these systems. Sequence complementary 3-mers form the most stable complexes with association constants of the order 10^4^–10^5^ M^–1^ in toluene (Table 4). These values are an order of magnitude greater than the corresponding values for formation of two base-pair duplexes reported in Table 3, providing good evidence for the formation of fully assembled duplexes with three phenol·phosphine oxide H-bonds in all three systems.

**Table 4:** Association Constants for Duplex Formation between Sequence-Complementary 3-mers Measured by ITC Titration Experiments in Toluene at 298 K^a^

complex	log *K* (M^–1^)	*ΔG°* (kJ mol^–1^)	*ΔH°* (kJ mol^–1^)	*ΔS°* (J K^–1^ mol^–1^)
**AAA**·**DDD**	5.2	**–29.5**	–57	–92
**AAD**·**ADD**	4.2	–23.9	–45	–71
**ADA**·**DAD**	4.3	–24.6	–50	–85

a Errors based on repeat experiments are 0.1 in log *K*, 0.5 kJ mol^–1^ in Δ*G*°, 5 kJ mol^–1^ in Δ*H*°, and 20 J K^–1^ mol^–1^ in Δ*S*°.

**Figure 9:**
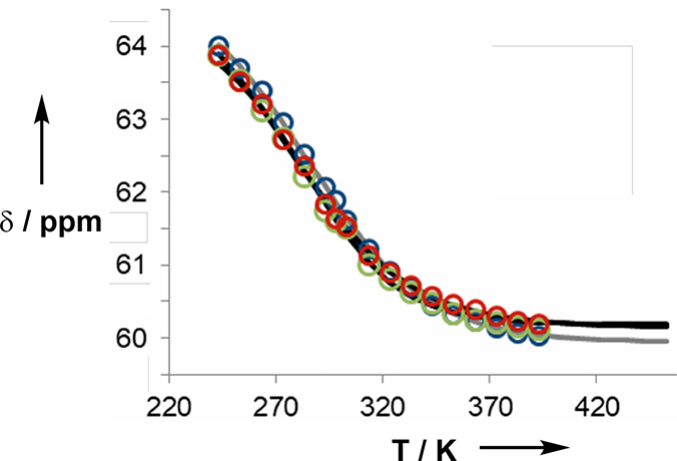
Average ^31^P NMR chemical shifts in d_4_-1,1,2,2-tetrachloroethane plotted as a function of temperature for 0.1 mM 1:1 mixtures of sequence-complementary 3-mers: **AAA**·**DDD** (blue), **AAD**·**DDA** (green), and **ADA**·**DAD** (red).

Duplex formation by sequence complementary 3-mers was confirmed using NMR melting experiments. Figure 9 shows the average ^31^P NMR chemical shift measured in d_4_-1,1,2,2-tetrachloroethane for a 1:1 mixture of **AAA** and **DDD**, a 1:1 mixture of **AAD** and **ADD**, and a 1:1 mixture of **ADA** and **DAD**. The behavior of all three duplexes is practically identical. The chemical shift decreases from a value of 64 ppm at low temperatures, which is characteristic of H-bonded phosphine oxide groups, to 60 ppm at high temperatures, which is characteristic of free phosphine oxide groups.

**Figure 10:**
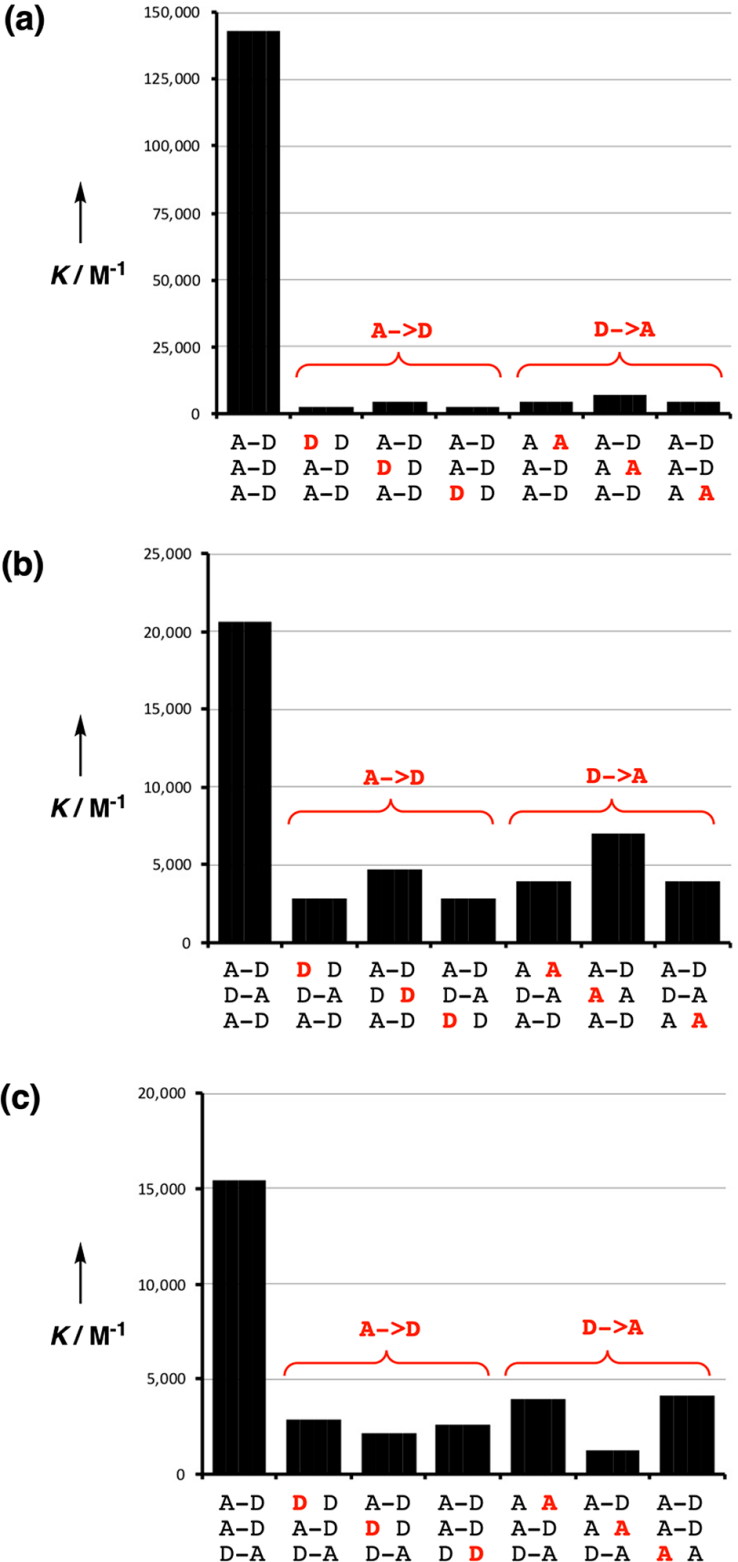
Effects of single A → D and D → A mutations (red) on the stabilities of sequence-complementary duplexes: (a) **AAA**·**DDD**, (b) **ADA**·**DAD**, and (c) **DDA**·**AAD.**.

Figure 10 illustrates the results for the introduction of single base mismatches into the 3-mer duplexes in toluene. In every case, there is a significant drop in the stability of the duplex compared with the matched sequence. The melamine backbone therefore provides much higher-fidelity sequence-selective duplex formation than the polyaniline backbone that we reported previously. The reason is illustrated in Figure 1b. 1,3-Folding of the polyaniline backbone reduces the stability of the **AAD·ADD** duplex, which results in an increase in the relative stability of duplexes formed by these oligomers with mismatched sequences that do not fold. The melamine backbone abolishes 1,3-folding and leads to the excellent sequence-selectivity illustrated in Figure 10.

The duplexes that have a single base mismatch have association constants of the order 10^3^ M^–1^ in toluene. For most combinations of 3-mer that feature two base mismatches, it was not possible to accurately determine association constants, because the 1:1 complexes are not sufficiently stable. The green data in Figure 8 shows one example: titration of **DAD** into **DDD**. This complex can form only one H-bond, but **DAD** dimerizes via formation of two H-bonds. Thus, the 1:1 complex formed by the two mismatched oligomers is never significantly populated in the titration. The ITC data for these systems fit well to an isotherm that considers only dimerization of the guest, albeit with a dimerization constant approximately 2-fold lower than the value obtained in dilution experiments in the absence of a competing host.

**Figure 11:**
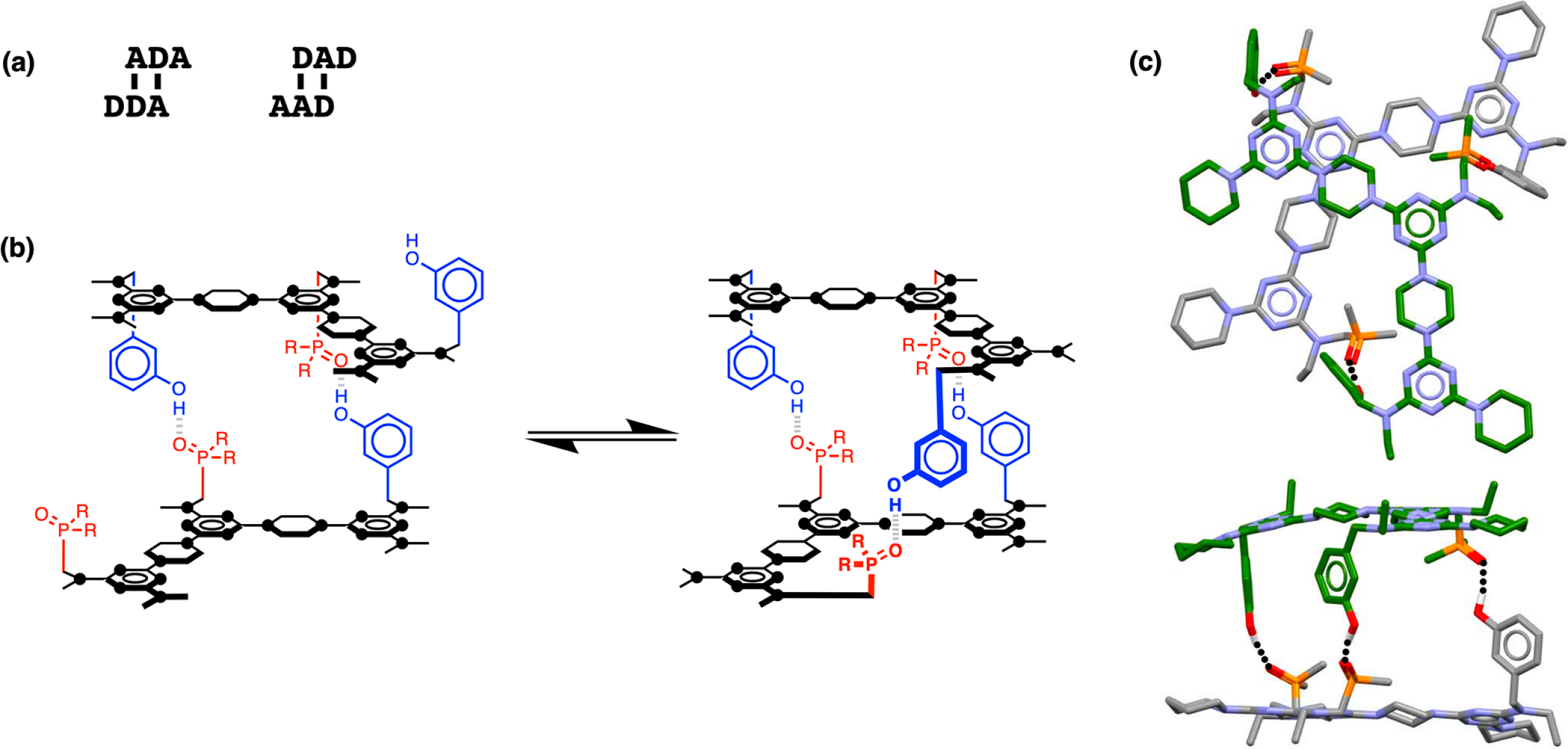
(a) Two mismatched duplexes that are expected to make two H-bonds when the sequences are arranged in a linear fashion but actually form three H-bonds based on ITC measurements. (b) Structure of the **DAD·AAD** duplex showing how the terminal dangling bases (left) can fold back to make a third H-bond (right). The melamine nitrogen atoms are represented as black dots, and the terminal piperidine and solubilizing groups are not shown. (c) Top and side views of the energy minimum structure of the **DAD**·**AAD** duplex (MMFFs with chloroform solvation). H-bonds are indicated by black dotted lines. The solubilizing groups and hydrogen atoms are omitted for clarity

There are two cases where the stability of the complex formed by noncomplementary 3-mers with two base mismatches is anomalously high: **DAD**·**AAD** and **ADA**·**DDA** have values of log *K* of 4.0 and 3.9, respectively. This result implies that three H-bonds are formed in each of these complexes. All four of the 3-mers involved contain the self-complementary **AD** motif and so would be expected to form duplexes with two H-bonds, both with themselves and with one another. However, what the **DAD**·**AAD** and **ADA**·**DDA** duplexes have in common is that the unpaired bases on opposite ends of the duplex are complementary (Figure 11a). Figure 11b shows how the structure of the **ADA**·**DDA** duplex can be reorganized to allow the unpaired terminal bases to fold back to make a third intermolecular H-bond. This feature is likely to be unique to the 3-mer duplexes, because the arrangement of the two backbones is not compatible with propagation of the base-pairing interactions in a longer duplex. The only other combination of oligomers where this arrangement of base-pairing interactions is possible is homosequence duplex **AAA·DDD**. The population of multiple isomeric complexes would account for the unusually high stability observed for this system compared with the other sequence complementary duplexes.

## Conclusion

A new family of recognition-encoded oligomers has been developed. Complementary phenol and phosphine oxide side-chains provide the recognition units, which lead to duplex formation through the formation of intermolecular H-bonds. The backbone is composed of repeating triazine-piperidine units, providing sufficient rigidity to prevent intramolecular folding of oligomers three recognition units in length. NMR spectroscopy and ITC were used to characterize the self-assembly properties of the oligomers. For length-complementary homo-oligomers, duplex formation in toluene is characterized by an increase in stability of an order of magnitude for every base-pair added to the chain. NMR spectra of dilute solutions of the **AD** 2-mer show intramolecular H-bonding between neighboring recognition units on the chain (1,2-folding) does not occur. Similarly, NMR spectra of dilute solutions of both the **AAD** and the **ADD** 3-mer show that 1,3-folding does not take place in this system either. ITC was used to characterize interactions between all pairwise combinations of the six different 3-mer sequences, and the sequence complementary duplexes are approximately an order of magnitude more stable than duplexes with a single base mismatch. High-fidelity duplex formation combined with the synthetic accessibility of the monomer building blocks makes these systems attractive targets for the investigation of the properties of longer oligomers.

## Supporting Information Available

Materials and methods, detailed synthetic procedures, full characterization including ^1^H and ^13^C NMR spectra of all compounds, and the NMR and ITC titration data (PDF)
