# Supplementary material for: High-Fidelity Sequence-Selective Duplex Formation by Recognition-Encoded Melamine Oligomers
Source: J Am Chem Soc. 2021 Jun 3;143(23):8669–78. doi: 10.1021/jacs.1c02275 (PMC8213060; doi:10.1021/jacs.1c02275)
Supplement: Supplementary file 1 — ja1c02275_si_001.pdf [file ja1c02275_si_001.pdf]

High fidelity sequence-selective duplex formation by recognition-encoded melamine oligomers.

Pavle Troselj,[‡a] Peter Bolgar,[‡a] Pablo Ballester [b,c] and Christopher A. Hunter [a,*]

[a] Yusuf Hamied Department of Chemistry, University of Cambridge, Lensfield Road, Cambridge, CB2 1EW, UK

[b] Institute of Chemical Research of Catalonia (ICIQ), Av. Països Catalans 16, 43007, Tarragona, Spain.

[c] Catalan Institution for Research and Advanced Studies (ICREA), Pg. Lluís Companys 23, 08010, Barcelona.

Supplementary Information

Synthesis of building blocks	S2
Synthesis of reference compound	S30
Synthesis of 1-mers	S34
Synthesis of 2-mers	S39
Synthesis of 3-mers	S47
Synthesis of 4-mers	S67
^{31}P NMR titration and dilution experiments	S72
Isothermal titration calorimetry experiments	S74

General experimental details

All the reagents and materials were obtained from commercial sources (Acros Organics, Alfa Aesar, Fischer Scientific, Fluorochem and Merck) and used without further purification. Dry THF and DCM were taken from the solvent purification system Pure Solv™ by Innovative Technology, Inc.. Thin layer chromatography was carried out using Silica gel 60F on glass. Flash chromatography was carried out on an automated system (CombiFlash Rf+ or Rf Lumen) using pre-packed cartridges of silica (25 µm PuriFlash® Column). NMR spectra were recorded on Bruker 400 MHz DPX400, 400 MHz AVIII400, 500 MHz DCH cryoprobe or 500 MHz TCI Cryoprobe spectrometer. The residual ^{1}H isotopologue of the deuterated solvents were used as internal standards for referencing. All ^{1}H and ^{13}C spectra were recorded at 298.0 ± 0.1 K. In chloroform-*d*, ^{1}H spectra were referenced to δ 7.26 ppm and ^{13}C spectra to δ 77.16 ppm for the solvent signal. In THF-d_8, ^{1}H spectra were referenced to δ 3.58 ppm and ^{13}C spectra to δ 67.21 ppm for the solvent signal. All chemical shifts are quoted in ppm on the δ scale. Splitting patterns are given as follows: s (singlet), br s (broad singlet), d (doublet), t (triplet), q (quartet), non (nonet), m (multiplet). A Waters LCT premier mass spectrometer was used to obtain the ES+ mass spectra. FT-IR spectra were measured on a PerkinElmer Spectrum One spectrometer equipped with an ATR cell. HRMS analyses were carried out on a Waters LCT Premier equipped with a TOF mass analyser and W optics for enhanced resolution, using 50% aqueous acetonitrile with 0.25% formic acid as mobile phase. LC-MS analyses were performed on Waters ACQUITY H-Class UPLC with an ESCi Multi-Mode Ionisation Waters SQ Detector 2 spectrometer. ITC experiments were carried out with a Malvern Microcal VP-ITC instrument.

Synthesis of building blocks

Synthesis of **1**

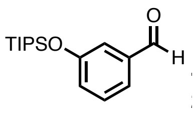

1 was made according to literature procedure found at
Juchum, M.; Günther, M.; Döring, E.; Sievers-Engler, A.; Lämmerhofer, M.; Laufer, S. Trisubstituted Imidazoles with a Rigidized Hinge Binding Motif Act As Single Digit nM Inhibitors of Clinically Relevant EGFR L858R/T790M and L858R/T790M/C797S Mutants: An Example of Target Hopping. *J. Med. Chem.* **2017**, *60*, 4636.

Synthesis of **2**

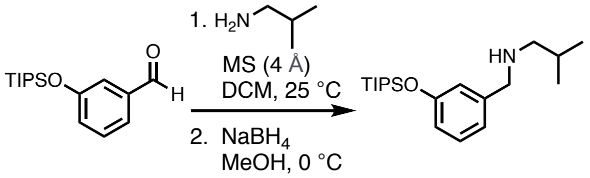

Isobutyl amine (0.92 mL, 0.68 g, 9.3 mmol, 1.1 eq) and molecular sieves (4 Å) were added to a solution of **1** (2.35 g, 8.44 mmol, 1.0 eq) in DCM (20 mL) at room temperature. The solution was stirred at room temperature until complete conversion of the aldehyde, which was monitored by ^{1}H NMR. The molecular sieves were removed and the solvent was evaporated *in vacuo* to obtain a colourless oil.

MeOH (20 mL) was added and the solution was cooled down to 0 °C, before sodium borohydride (351 mg, 9.28 mmol, 1.1 eq) was added. The reaction mixture was stirred at room temperature until the disappearance of the imine intermediate, as monitored by ^{1}H NMR. The solvent was removed *in vacuo* and the residue was dissolved in NaOH solution (1M in water, 80 mL). The solution was extracted with DCM (3 x 30 mL). The organic phase was dried ($MgSO_4$) and the solvent was evaporated *in vacuo*. The crude was purified by flash chromatography (SiO_2, 0-10% MeOH in DCM to yield the product as a pale yellow oil (2.60 g, 7.75 mmol, 92% yield).

^{1}H NMR (400 MHz, $CDCl_3$): δ_H 7.16 (t, *J* = 7.8 Hz, 1H), 6.89 – 6.83 (m, 2H), 6.76 (d, *J* = 8.2 Hz, 1H), 3.74 (s, 2H), 2.41 (d, *J* = 6.8 Hz, 2H), 1.75 (non, 6.6 Hz, 1H), 1.25 (m, 4H), 1.10 (d, *J* = 7.3 Hz, 18H), 0.90 (d, *J* = 6.6 Hz, 6H);
^{13}C NMR (101 MHz, $CDCl_3$): δ_C 156.3, 142.2, 129.3, 120.9, 119.8, 118.4, 57.3, 53.9, 28.5, 20.8, 18.1, 12.9;
FT-IR (ATR): ν_{max} /cm^{-1} 2944, 2866, 1602, 1586, 1485, 1462, 1442, 1385, 1276, 1166, 1154, 1115, 1073, 1004, 963, 919, 881, 818, 782, 680, 562, 509, 448;
HRMS (ES+): calcd for $C_{20}H_{37}NOSi + H^+$ is 336.2723, found 336.2714 (-2.7 ppm).

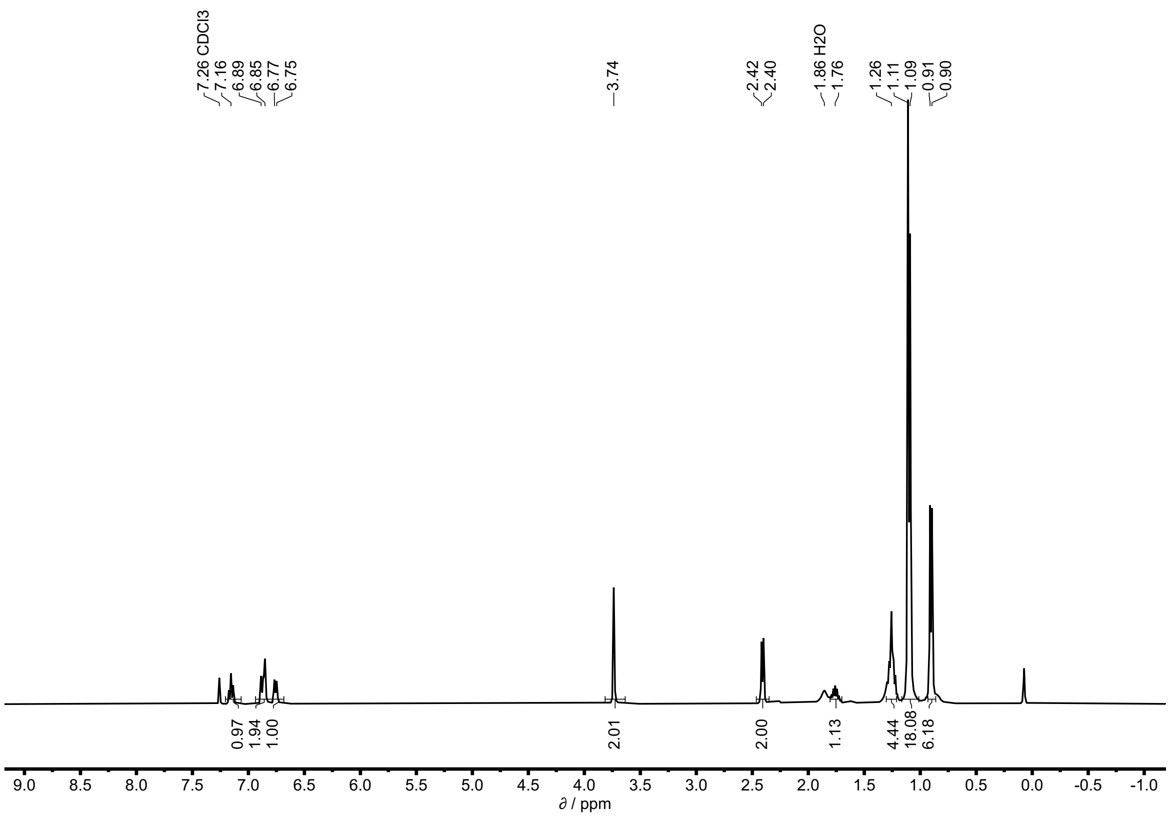

Figure S1. ^{1}H NMR spectrum (400 MHz, $CDCl_3$) of compound **2**.

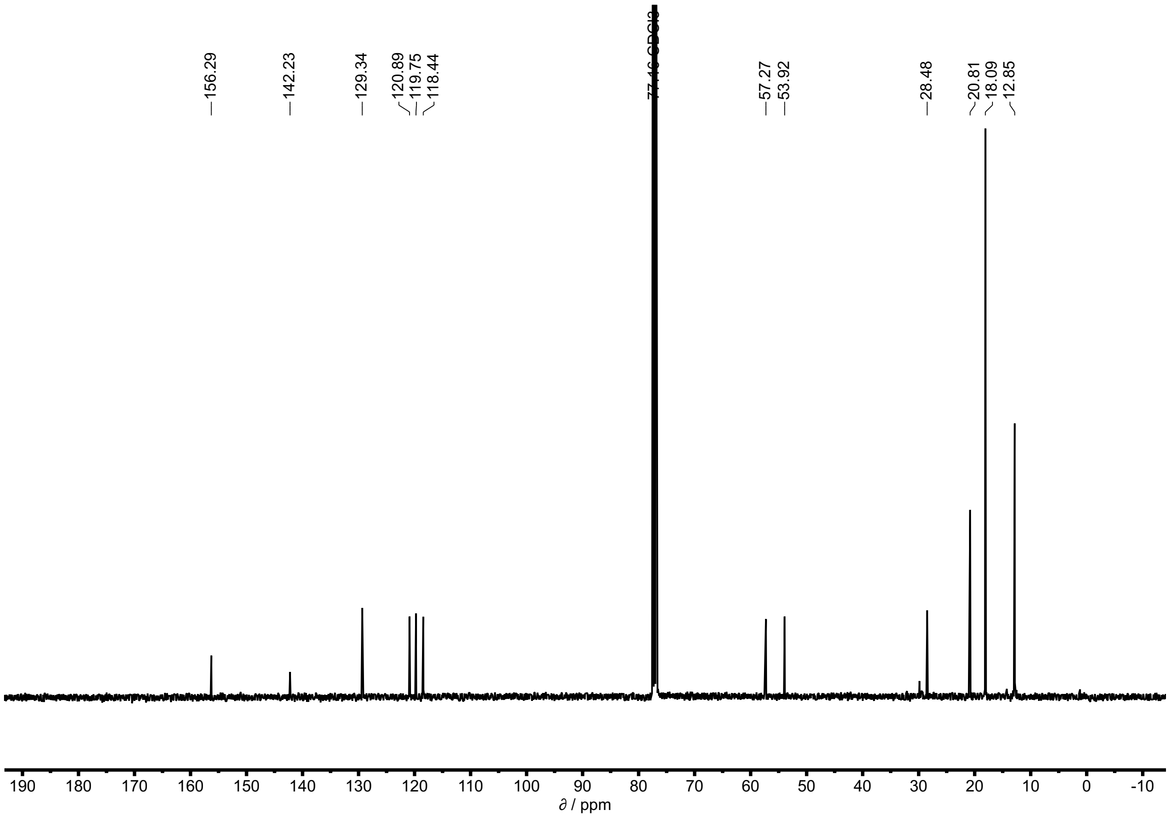

Figure S2. ^{13}C NMR spectrum (101 MHz, $CDCl_3$) of compound **2**.

Synthesis of **5**

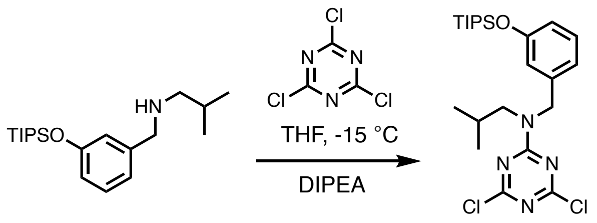

To a solution of cyanuric chloride (1.7 g, 9.2 mmol, 1.2 eq) in THF (25 mL) at -10 °C was added dropwise a solution of amine **2** (2.5 g, 7.5 mmol, 1.0 eq) in THF (5 mL), followed by DIPEA (2.6 mL, 1.94 g, 15.0 mmol, 2.0 eq). The solution was stirred at -10 °C for 1h. The solvent was evaporated *in vacuo*, and the residues were dissolved in EtOAc (50 mL). The solution was washed with citric acid solution (5% in water, 3 x 20 mL). The aqueous phase was extracted with EtOAc (1 x 20 mL). The combined organic phases were dried ($MgSO_4$) and the solvent was evaporated *in vacuo*. The crude was purified by flash chromatography (SiO_2, 0-30% gradient of EtOAc in 40-60 Pet. Ether). The product was obtained as a colourless oil (2.95 g, 6.1 mmol, 81% yield).

^{1}H NMR (400 MHz, $CDCl_3$): δ_H 7.18 (t, *J* = 7.8 Hz, 1H), 6.82-6.78 (m, 2H), 6.72 (t, *J* = 2.1 Hz, 1H), 4.81 (s, 2H), 3.39 (d, *J* = 7.6 Hz, 2H), 2.12 (non, *J* = 6.9 Hz, 1H), 1.20 (hep, *J* = 7.2 Hz, 3H), 1.07 (d, *J* = 7.3 Hz, 18H), 0.92 (d, *J* = 6.7 Hz, 6H);
^{13}C NMR (101 MHz, $CDCl_3$): δ_C 170.2, 165.7, 156.6, 137.2, 129.9, 120.5, 119.7, 119.1, 54.0, 50.8, 26.8, 20.2, 18.0, 12.8;
FT-IR (ATR): ν_{max} /cm^{-1} 2945, 2867, 1558, 1476, 1436, 1389, 1348, 1327, 1278, 1232, 1168, 1059, 1003, 982, 881, 845, 796, 682, 574;
HRMS (ES+): calcd for $C_{23}H_{36}Cl_2N_4OSi + H^+$ is 483.2114, found 483.2108 (-1.2 ppm).

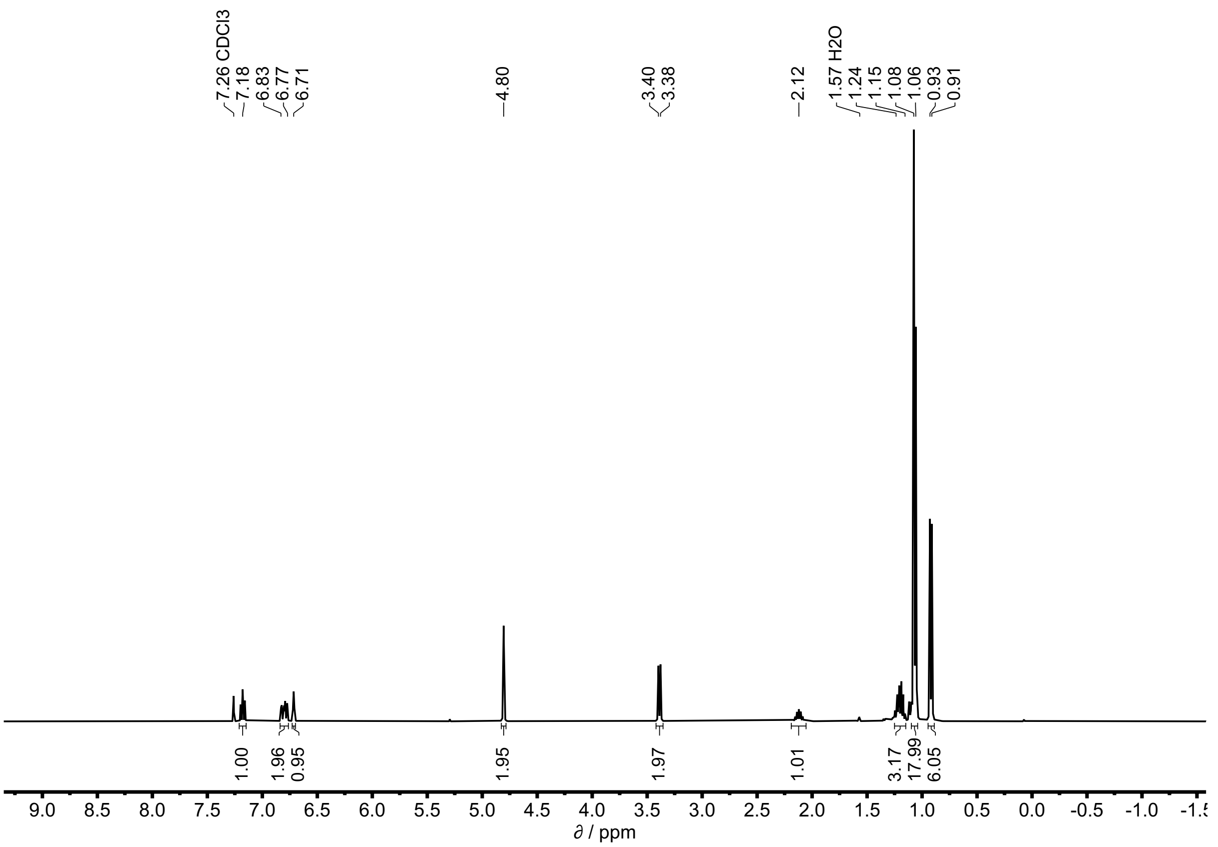

Figure S3. ^{1}H NMR spectrum (400 MHz, $CDCl_3$) of compound **5**.

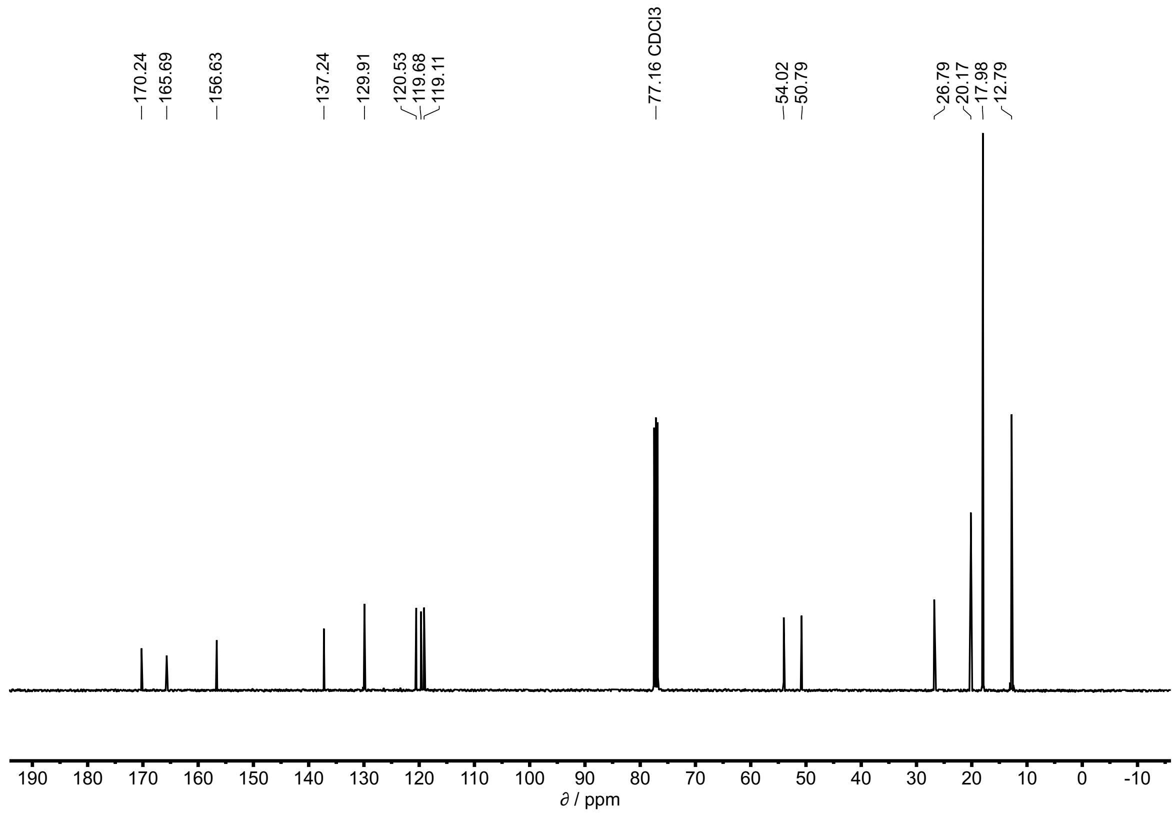

Figure S4. ^{13}C NMR spectrum (101 MHz, $CDCl_3$) of compound **5**.

Synthesis of **6**

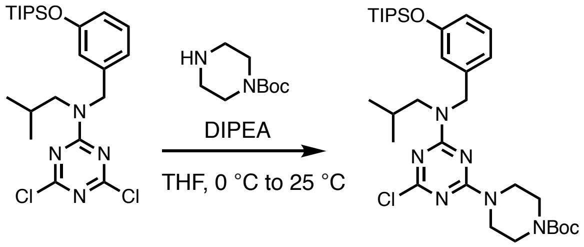

To a solution of **5** (1.00 g, 2.1 mmol, 1.0 eq) in THF (25 mL) at 0 °C was added 1-Boc-piperazine (385 mg, 2.1 mmol, 1.0 eq) and DIPEA (0.72 mL, 0.53 g, 4.1 mmol, 2.0 eq). The reaction mixture was stirred for 15 h at 22 °C. The solvent was removed *in vacuo* and the residue was dissolved in EtOAc (40 mL). The solution was washed with a solution of citric acid (5% in water, 3 x 15 mL). The aqueous wash was extracted with EtOAc (15 mL). The combined organic phases were dried ($MgSO_4$) and the solvent was evaporated *in vacuo*. The crude was purified by flash chromatography (SiO_2, 20-50% gradient of EtOAc in 40-60 Pet. Ether) to obtain a colourless viscous oil (1.20 g, 1.9 mmol, 92% yield).

The NMR spectra are consistent with the presence of two slowly exchanging rotamers in solution. Where rotamers are clearly distinguishable, both of the corresponding peaks are listed together. Where corresponding peaks are broad and overlap, they are listed as a broad peak with a chemical shift range.

^{1}H NMR (400 MHz, $CDCl_3$): δ_H 7.14 and 7.14 (t, *J* = 7.7 Hz, 1H, rotamers), 6.77 (m, 2H), 6.71 and 6.67 (s, 1H, rotamers), 4.78 and 4.72 (s, 2H), 3.84 – 3.57 (br m, 4H, rotamers), 3.50 – 3.37 (br m, 4H, rotamers), 3.37 and 3.26 (d, *J* = 7.5 Hz, 2H, rotamers), 2.07 (m, *J* = 6.3 Hz, 1H), 1.48 and 1.46 (s, 9H, rotamers), 1.18 (m, *J* = 7.4 Hz, 3H), 1.06 and 1.05 (d, *J* = 7.4 Hz, 18H), 0.90 and 0.89 (t, *J* = 6.3 Hz, 6H);
^{13}C NMR (101 MHz, $CDCl_3$): δ_C 169.7 and 169.6 (rotamers), 165.8, 164.7 and 164.6 (rotamers), 156.5 and 156.4 (rotamers), 154.8, 139.2 and 139.1 (rotamers), 129.6, 120.5 and 120.0 (rotamers), 119.1 and 119.0 (rotamers), 118.9 and 118.4 (rotamers), 80.3 and 80.3 (rotamers), 53.8 and 53.7 (rotamers), 50.5 and 50.3 (rotamers), 43.4, 43.3, 28.6, 27.3 and 27.1 (rotamers), 20.57 and 20.2 (rotamers), 18.0, 12.8;
FT-IR (ATR): ν_{max} /cm^{-1} 2947, 2866, 1691, 1603, 1562, 1486, 1410, 1365, 1311, 1273, 1236, 1170, 1106, 1044, 977, 882, 868, 829, 811, 798, 777, 705, 689, 640, 577, 527, 460;
HRMS (ES+): calcd for $C_{32}H_{53}ClN_6O_3Si + H^+$ is 633.3715, found 633.3701 (-2.2 ppm).

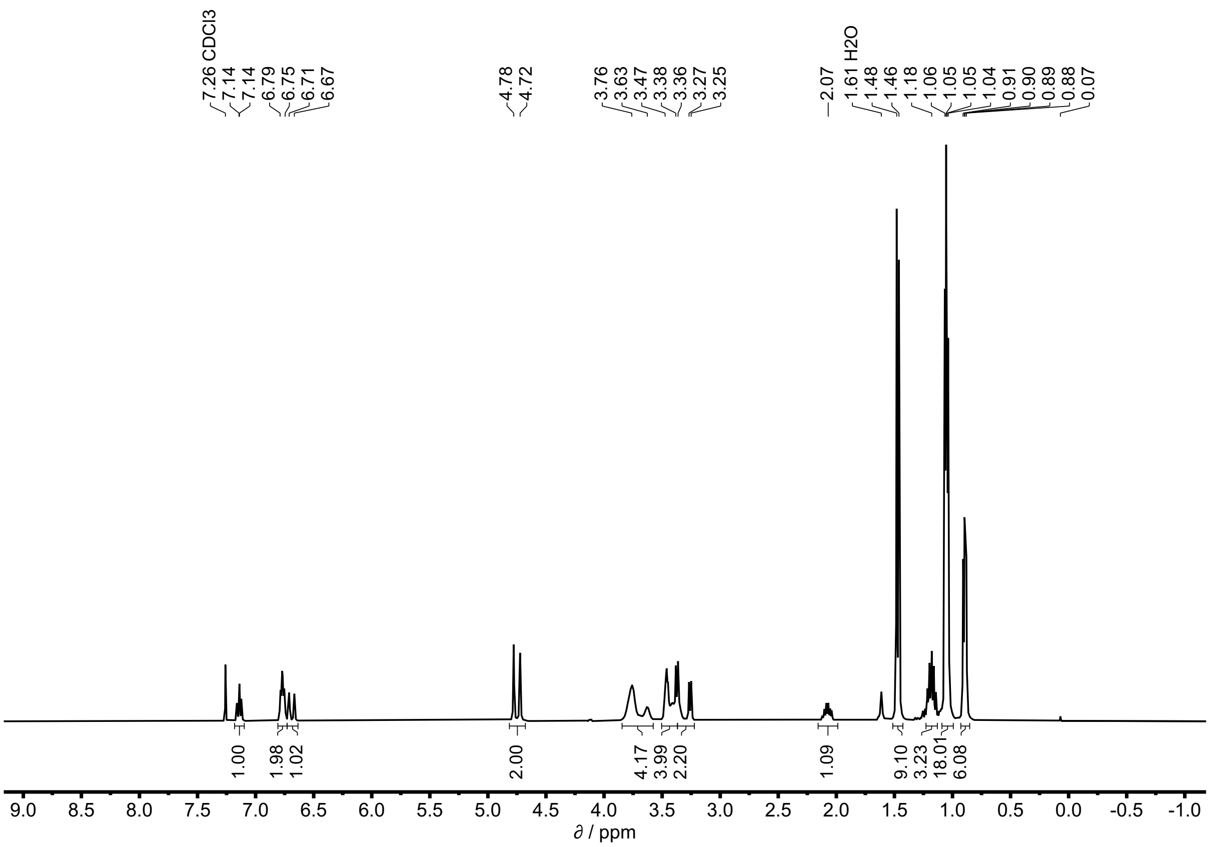

Figure S5. ^{1}H NMR spectrum (400 MHz, $CDCl_3$) of compound **6**.

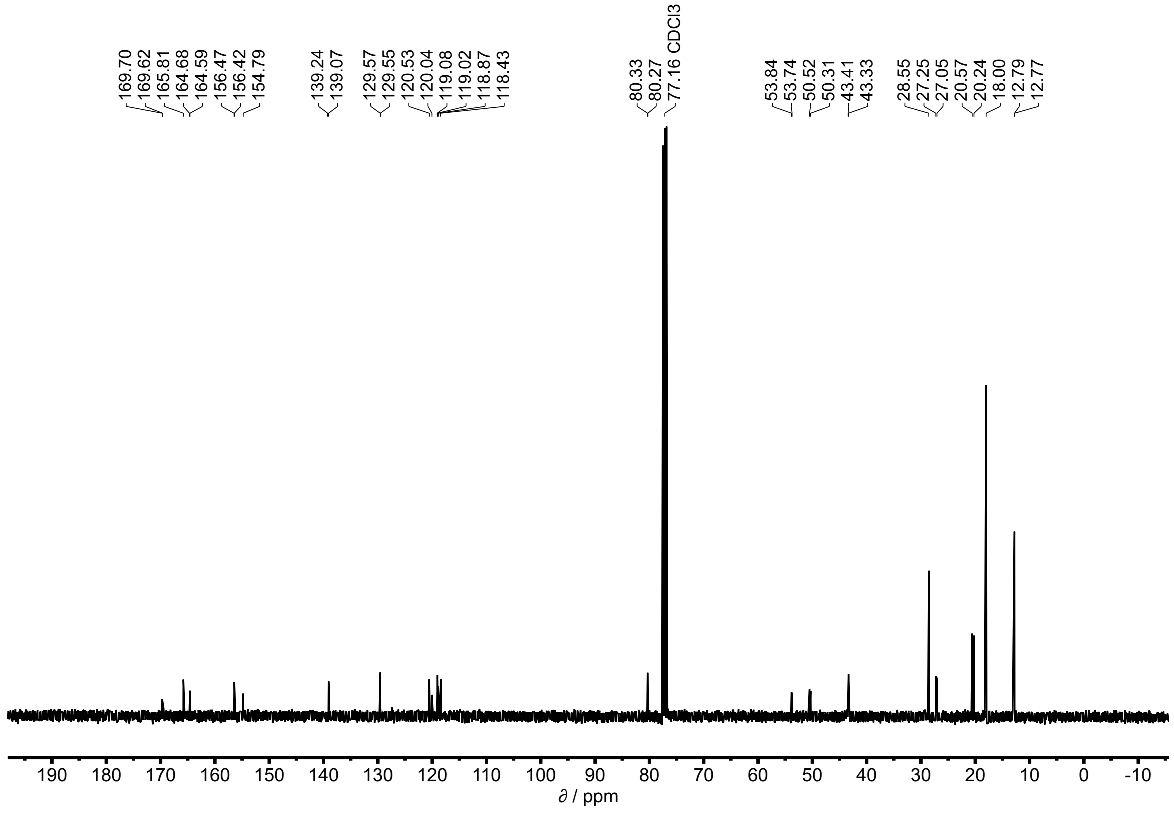

Figure S6. ^{13}C NMR spectrum (101 MHz, $CDCl_3$) of compound **6**.

Synthesis of **7**

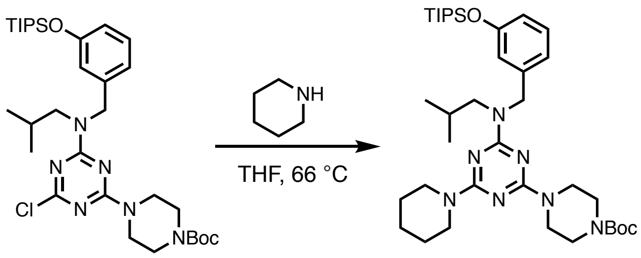

A solution of **6** (500 mg, 0.790 mmol, 1.0 eq) and piperidine (312 µL, 269 mg, 3.16 mmol, 4.0 eq) in THF (15 mL) was heated under reflux for 15 h. The solvent was removed *in vacuo* and the residue was dissolved in EtOAc (40 mL). The solution was washed with a solution of citric acid (5% in water, 3 x 15 mL). The aqueous wash was extracted with EtOAc (15 mL). The combined organic phases were dried ($MgSO_4$) and the solvent was evaporated *in vacuo*. The crude was purified by flash chromatography (SiO_2, 0-20% gradient of EtOAc in 40-60 Pet. Ether) to obtain the product as a colourless viscous oil (535 mg, 0.784 mmol, 99% yield).

^{1}H NMR (400 MHz, $CDCl_3$): δ_H 7.11 (t, *J* = 7.5 Hz, 1H), 6.80 (m, 1H), 6.74 -6.69 (m, 2H), 4.76 and 4.75 (s, 2H, rotamers), 3.76-3.62 (m, 8H, rotamers), 3.47-3.33 (m, 4H, rotamers), 3.28 (m, 2H, rotamers), 2.05 (m, 1H), 1.67-1.42 (m, 15H, overlaps with HDO), 1.17 (m, 3H), 1.04 (d, *J* = 7.2 Hz, 18H), 0.87 (d, *J* = 6.7 Hz, 6H);
^{13}C NMR (101 MHz, $CDCl_3$): δ_C 166.4, 165.7, 165.2, 156.2, 155.0, 141.2, 129.2, 120.4 and 120.2, 118.8 and 118.7, 118.3, 79.8, 53.7 and 53.6, 50.2 and 50.1, 44.3, 44.2, 43.2, 43.1 (1 pair of rotameric signals among the previous 4 peaks), 28.6, 27.6, 25.9, 25.2, 20.7, 18.1, 12.8;
FT-IR (ATR): ν_{max} /cm^{-1} 2930, 2865, 1699, 1602, 1587, 1529, 1481, 1415, 1364, 1275, 1230, 1164, 1101, 1074, 997, 882, 826, 806, 777, 683, 442;
HRMS (ES+): calcd for $C_{37}H_{63}N_7O_3Si + H^+$ is 682.4840, found 682.4836 (-0.6 ppm).

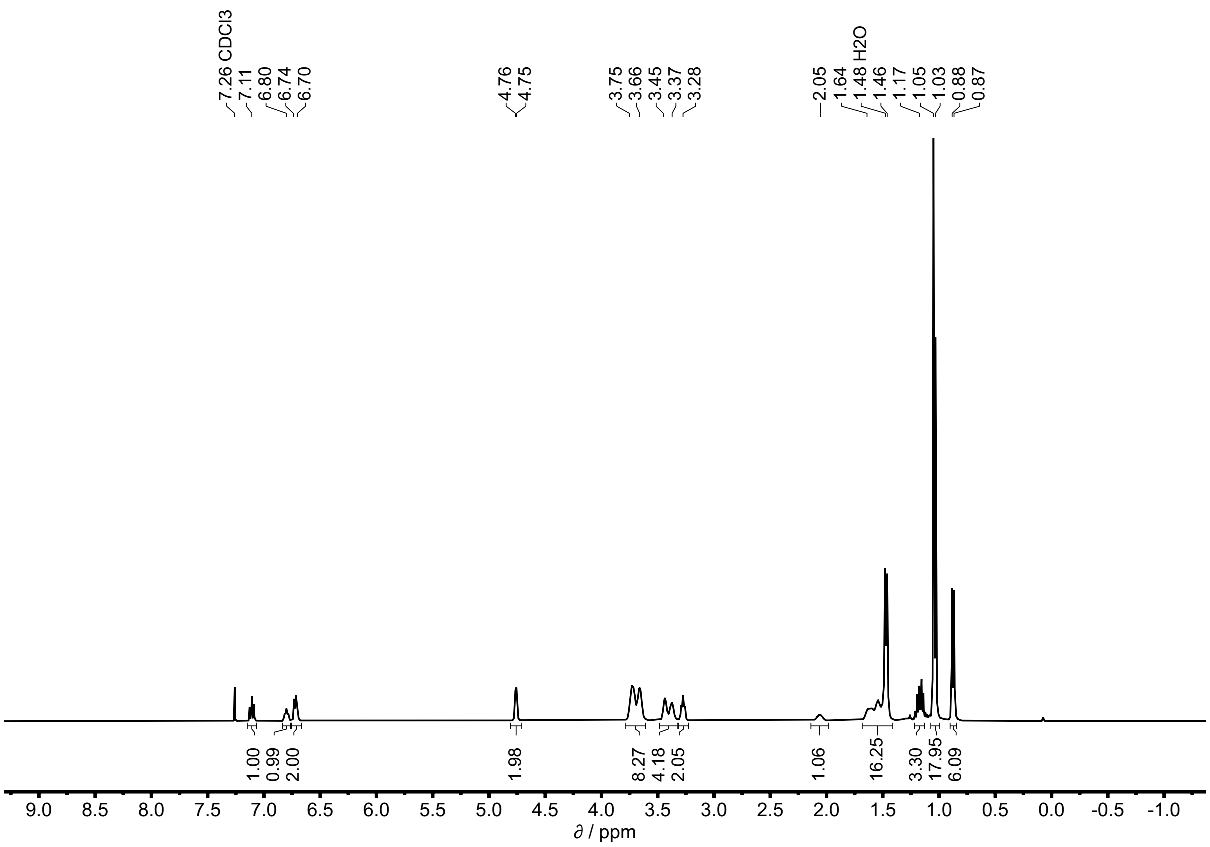

Figure S7. ^{1}H NMR spectrum (400 MHz, $CDCl_3$) of compound **7**.

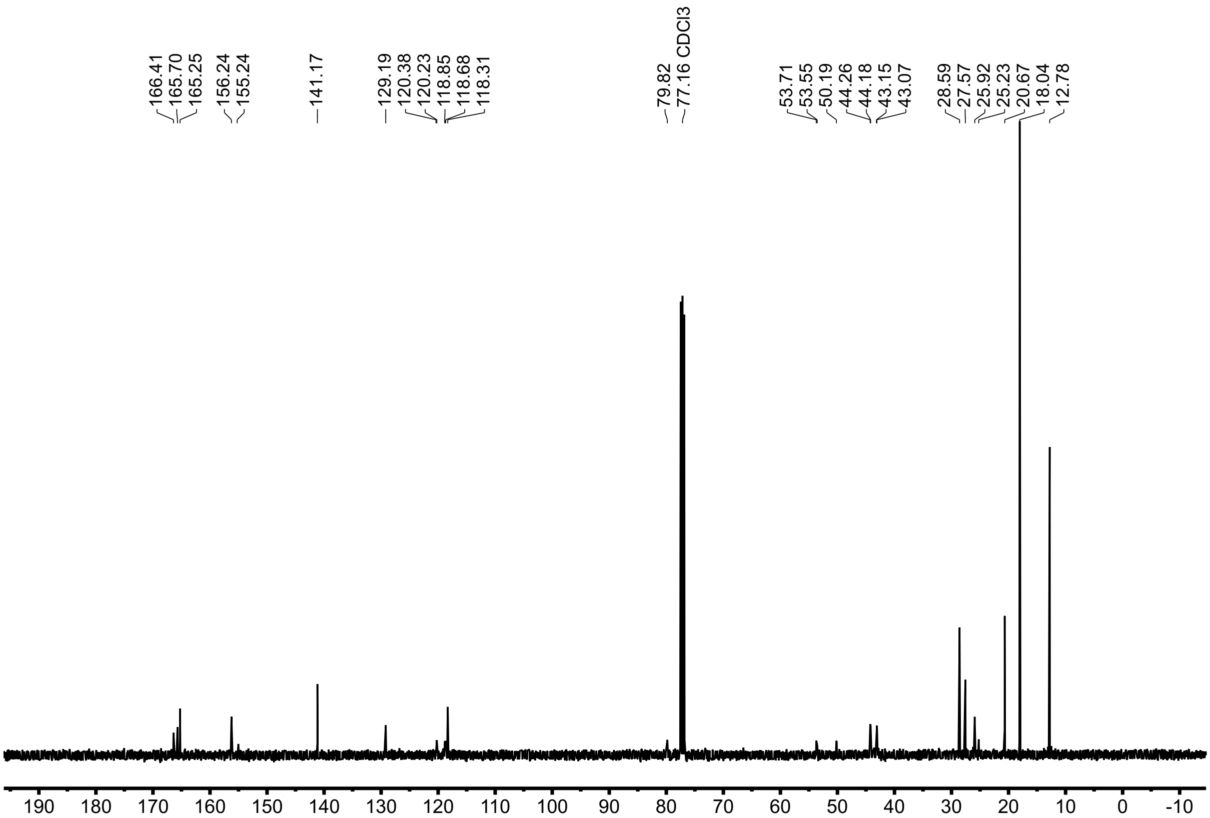

Figure S8. ^{13}C NMR spectrum (101 MHz, $CDCl_3$) of compound **7**.

Synthesis of **8**

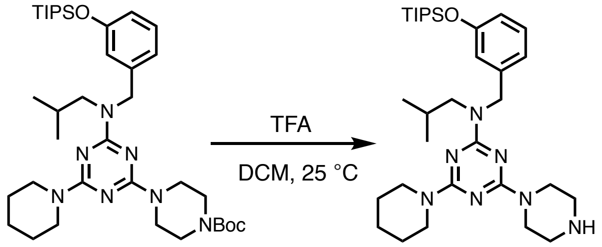

Trifluoroacetic acid (1.6 mL, 2.4 g, 21 mmol, 27 eq) was added dropwise to a solution of **7** (535 mg, 0.784 mmol, 1.0 eq) in DCM (20 mL) at 0 °C. The reaction mixture was stirred at 22 °C until complete conversion as monitored by LCMS. The reaction mixture was diluted with DCM (15 mL) and it was washed with NaOH solution (1 M in water, 3 x 15 mL). The aqueous phase was extracted with DCM (15 mL). The combined organic phases were dried ($MgSO_4$) and the solvent was removed *in vacuo*. The crude was purified by flash chromatography (SiO_2, 0-15% gradient of MeOH in DCM) to obtain the product as a colourless viscous oil (447 mg, 0.768 mmol, 98% yield).

^{1}H NMR (400 MHz, $CDCl_3$): δ_H 7.11 (t, *J* = 8.1 Hz, 1H), 6.81 (d, *J* = 7.6 Hz, 1H), 6.75-6.70 (m, 2H), 4.77 (s, 2H), 3.83-3.60 (m, 8H), 3.28 (d, *J* = 7.2 Hz, 2H), 2.87 and 2.81 (t, *J* = 4.9 Hz, 4H), 2.07 (non, *J* = 6.9 Hz, 1H), 1.93 (br s, 1H), 1.68 – 1.45 (m, 6H), 1.25 – 1.11 (m, 3H), 1.05 (d, *J* = 7.2 Hz, 18H), 0.88 (d, *J* = 6.7 Hz, 6H);
^{13}C NMR (101 MHz, $CDCl_3$): δ_C 166.37, 165.59, 165.25, 156.16, 141.23, 129.09, 120.33 and 120.26 (rotamers), 118.80 and 118.73 (rotamers), 118.21, 53.54 and 53.48 (rotamers), 50.06, 46.18 and 46.13 (rotamers), 44.40 and 44.34 and 44.18 and 44.10 (two rotameric pairs), 27.52, 25.88, 25.21 and 25.17 (roatmers), 20.63, 18.00, 12.74;
FT-IR (ATR): ν_{max} /cm^{-1} 2933, 2865, 1528, 1481, 1438, 1370, 1346, 1305, 1273, 1254, 1238, 1204, 1152, 1098, 985, 908, 882, 853, 807, 779, 733, 684, 645;
HRMS (ES+): calcd for $C_{32}H_{55}N_7OSi + H^+$ 582.4316, found 582.4326 (+1.7 ppm).

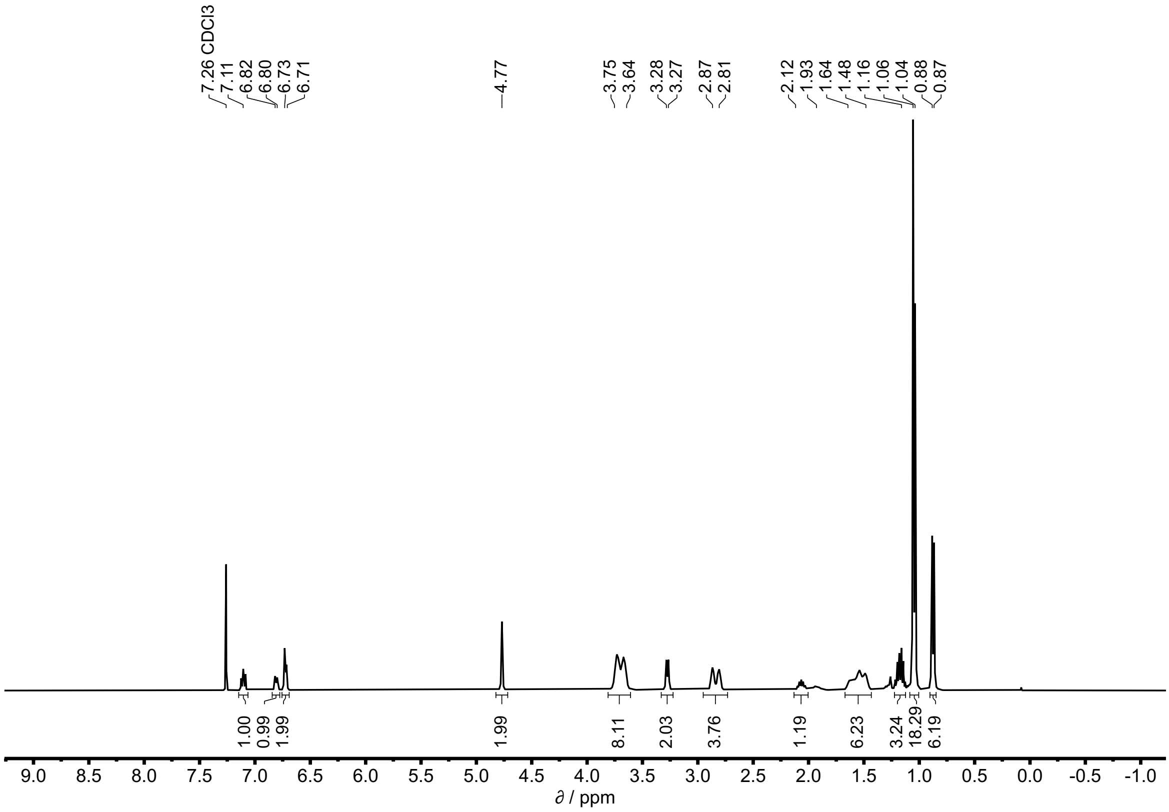

Figure S9. ^{1}H NMR spectrum (400 MHz, $CDCl_3$) of compound **8**.

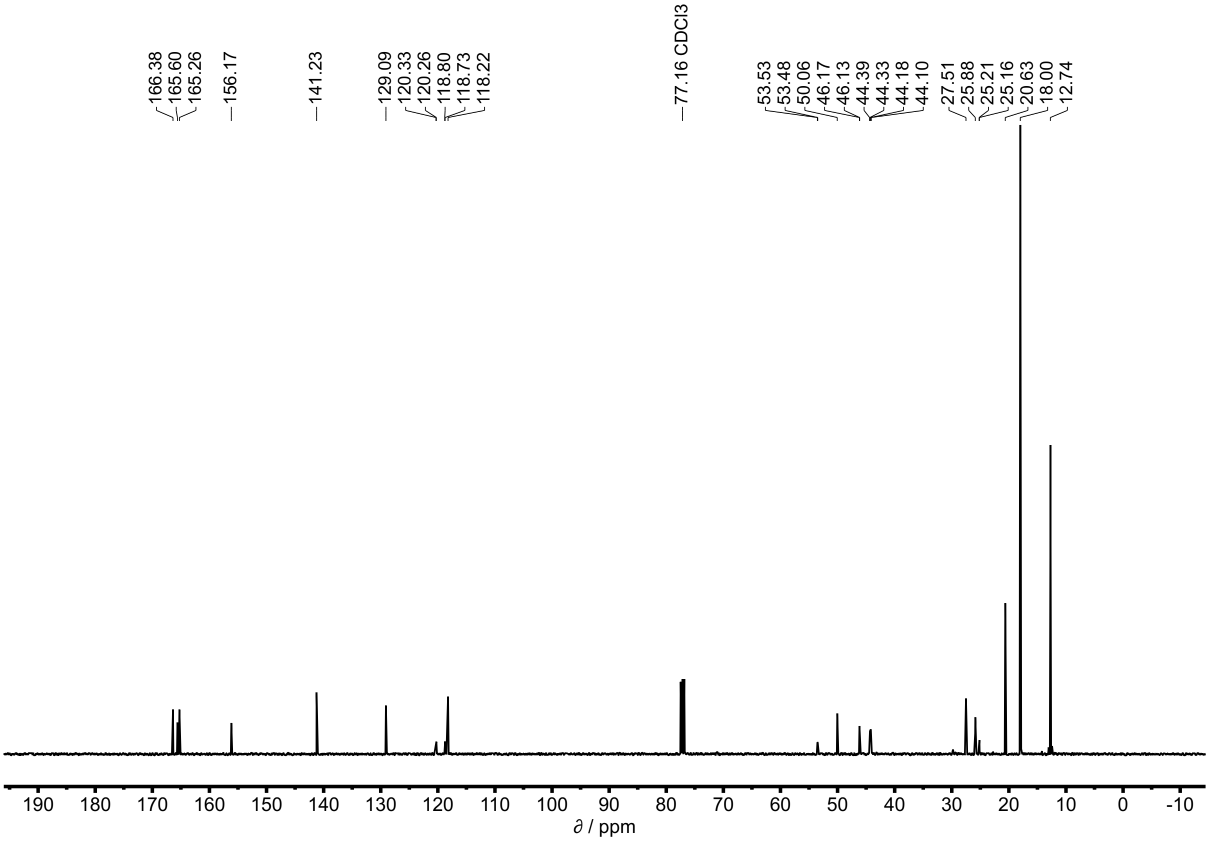

Figure S10. ^{13}C NMR spectrum (101 MHz, $CDCl_3$) of compound **8**.

Synthesis of **3**

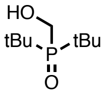

Compound **3** was synthesised as described in Iadevaia, G.; Stross, A. E.; Neumann, A.; Hunter, C. A. Mix and match backbones for the formation of H-bonded duplexes. *Chem. Sci.*, **2016**, *7*, 1760.

Synthesis of **4**

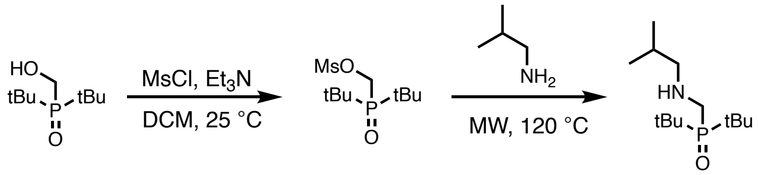

To a solution of **3** (5.5 g, 29 mmol, 1.0 eq) and triethylamine (6.0 mL, 4.3 g, 43 mmol, 1.5 eq) in DCM (100 mL) at 0 °C was added mesyl chloride (3.3 mL, 4.9 g, 43 mmol, 1.5 eq) dropwise over a period of 20 mins. The reaction mixture was stirred at 22 °C for 15 h, after which is was diluted with DCM (100 mL), washed with water (2 x 100 mL) and brine (100 mL) and then dried ($MgSO_4$). The solvent was removed *in vacuo*, and the crude was used in the next step without further purification.

The crude obtained in the previous step was dissolved in isobutylamine (20 mL, 14.7 g, 201 mmol, 6.9 equiv) and the reaction mixture was stirred at 120 °C in a microwave reactor for 2h. The excess of isobutylamine was removed *in vacuo* and the residue was dissolved in EtOAc (150 mL). The solution was washed with a sat. solution of Na_2CO_3 (3 x 30 mL), dried ($MgSO_4$) and evaporated *in vacuo*. The crude was purified by flash chromatography (SiO_2, 0–15% gradient of MeOH in DCM) to yield the product as a transparent oil (4.94 g, 20.0 mmol, 69% yield over two steps).

^{1}H NMR (400 MHz, CDCl$_3$): δ_H 2.92 (d, $^2J_{PH}$ = 6.4 Hz, 2H), 2.39 (d, J = 6.7 Hz, 2H), 1.67 (non, J = 6.7 Hz, 1H), 1.25 (d, $^3J_{PH}$ = 13.1 Hz, 18H), 0.86 (d, J = 6.7 Hz, 6H);
^{13}C NMR (101 MHz, CDCl$_3$): δ_C 60.7 (d, $^3J_{PC}$ = 11.7 Hz), 43.0 (d, $^1J_{PC}$ = 62.2 Hz), 35.4 (d, $^1J_{PC}$ = 57.7 Hz), 28.4, 26.9, 20.8;
^{31}P NMR (162 MHz, CDCl$_3$): δ_P 58.14;
FT-IR (ATR): ν_{max} /cm^{-1} 3401, 2951, 2870, 2809, 1469, 1389, 1367, 1201, 1143, 1020, 935, 816, 780, 644, 596, 516, 484, 455;
HRMS (ES+): calcd for $C_{13}H_{30}NOP + H^+$ is 248.2143, found 248.2150 (+2.8 ppm).

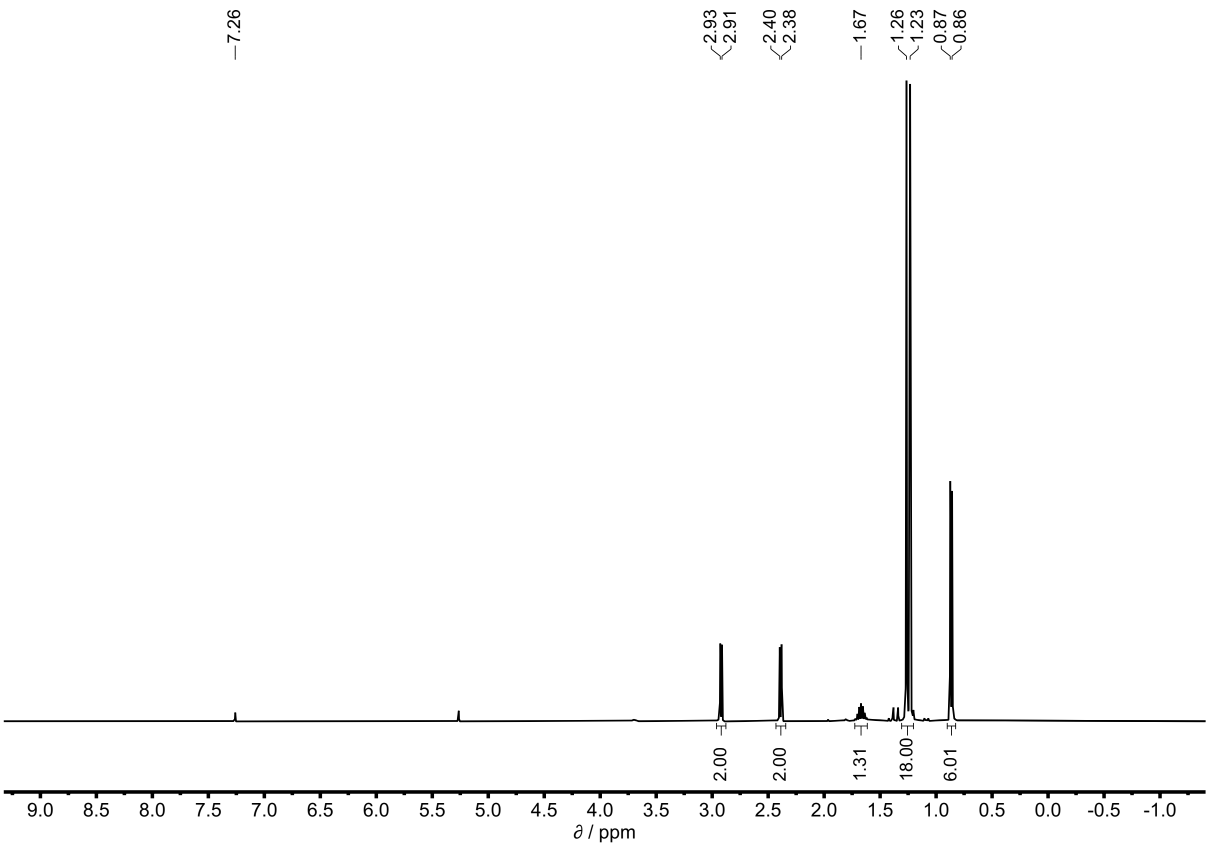

Figure S11. ^{1}H NMR spectrum (400 MHz, $CDCl_3$) of compound **4**.

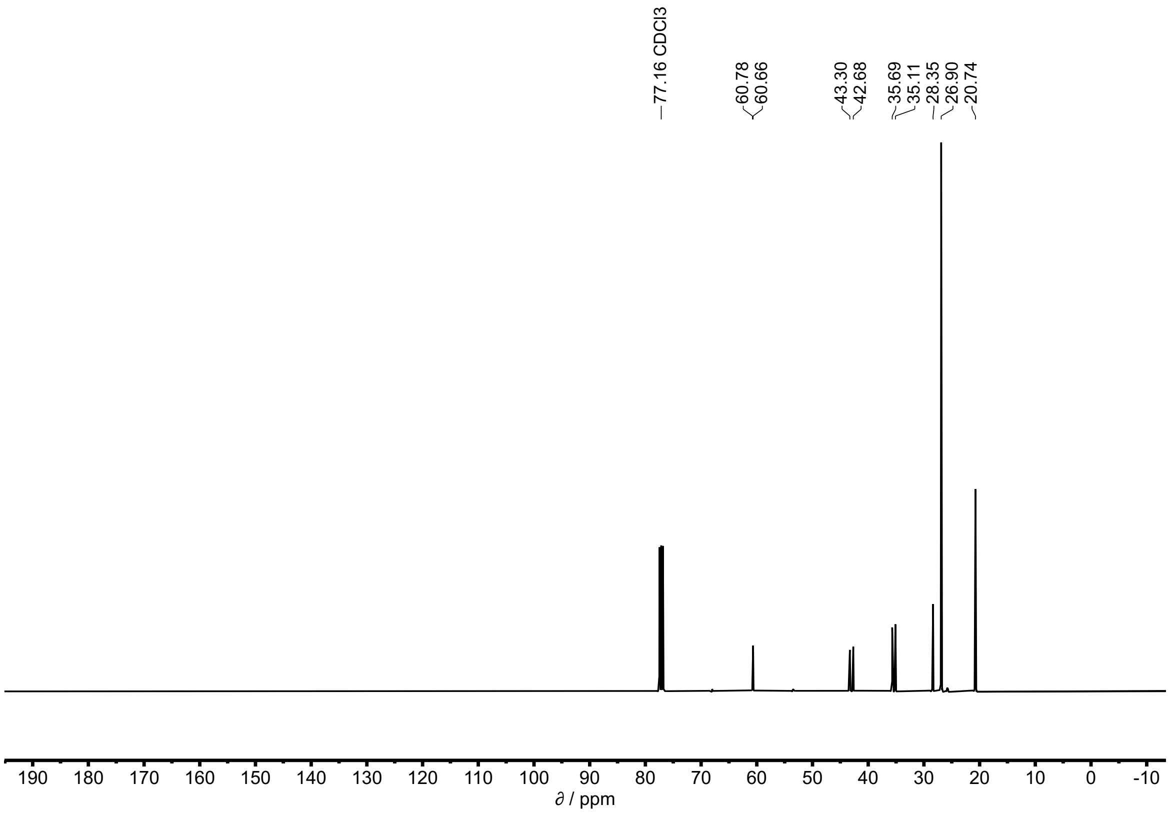

Figure S12. ^{13}C NMR spectrum (101 MHz, $CDCl_3$) of compound **4.**

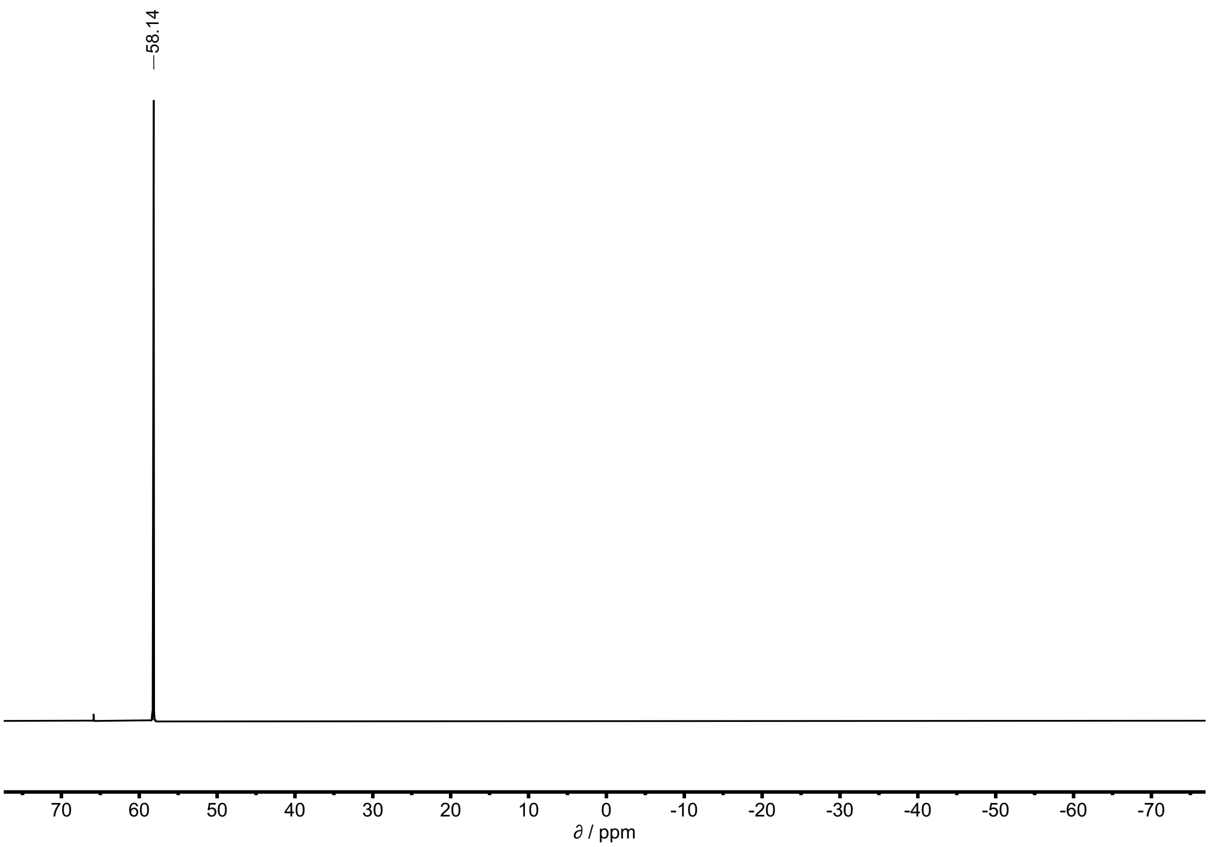

Figure S13. ^{31}P NMR spectrum (162 MHz, $CDCl_3$) of compound **4**.

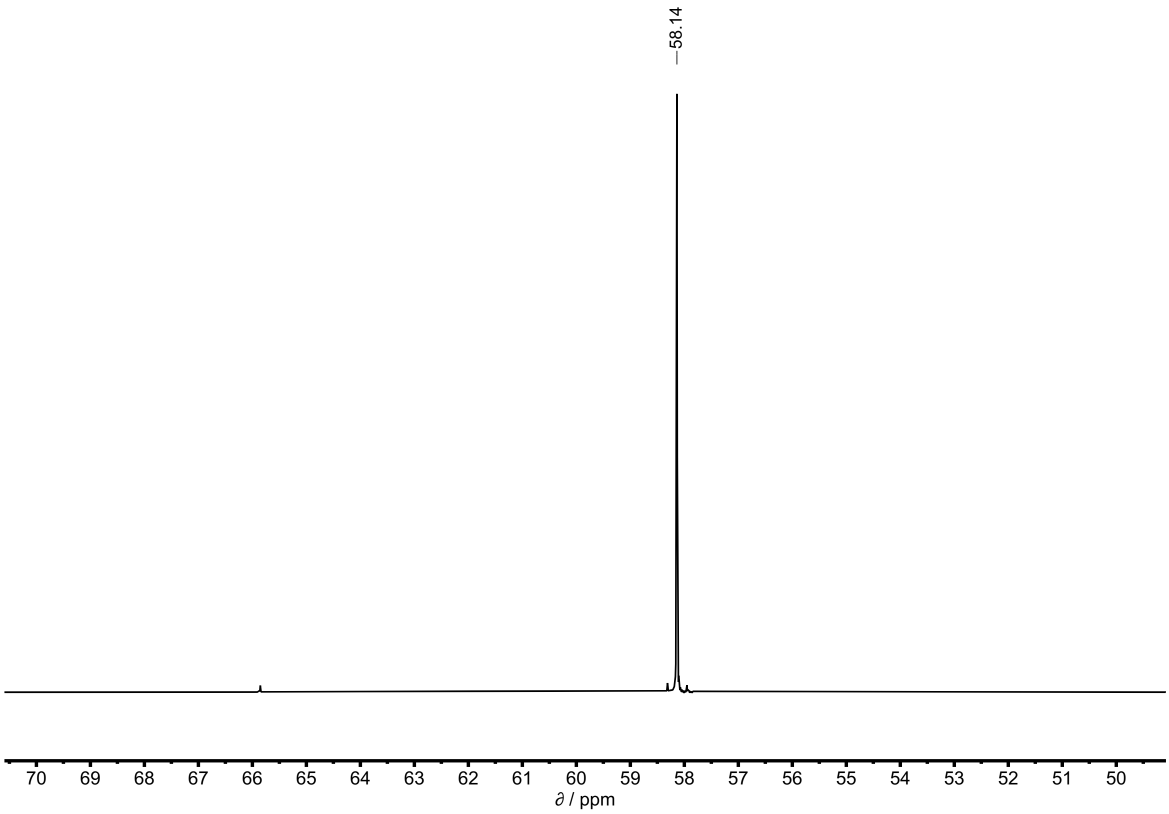

Figure S14. ^{31}P NMR spectrum (162 MHz, $CDCl_3$) of compound **4**.

Synthesis of **9**

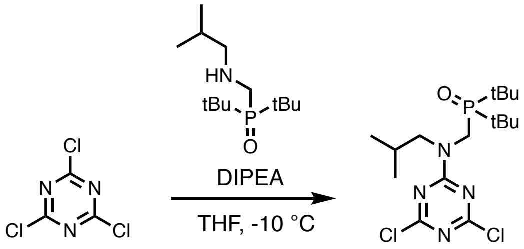

To a solution of cyanuric chloride (410 mg, 2.22 mmol, 1.1 eq) in THF (10 mL) at -10 °C was added dropwise a solution of amine **4** (500 mg, 2.02 mmol, 1.0 eq) in THF (5 mL), followed by DIPEA (704 μL, 522 mg, 4.04 mmol, 2.0 eq). The solution was stirred at -10 °C for 1h. The solvent was evaporated *in vacuo*, and the residues were dissolved in EtOAc (40 mL). The solution was washed with citric acid solution (5% in water, 3 x 15 mL). The aqueous wash was extracted with EtOAc (20 mL). The combined organic phases were dried ($MgSO_4$) and the solvent was evaporated *in vacuo*. The crude was purified by flash chromatography (SiO_2, 80-100% gradient of EtOAc in 40-60 Pet. Ether). The product was obtained as a white foam (750 mg, 1.90 mmol, 93% yield).

^{1}H NMR (400 MHz, $CDCl_3$): δ_H 4.30 (d, $^2J_{PH}$ = 3.6 Hz, 2H), 3.97 (d, J = 7.6 Hz, 2H), 2.19 (non, J = 6.9 Hz, 1H), 1.29 (d, $^3J_{PH}$ = 13.4 Hz, 18H), 0.92 (d, J = 6.7 Hz, 6H);
^{13}C NMR (101 MHz, $CDCl_3$): δ_C 170.4 and 170.0 (rotamers), 165.2, 54.3, 38.7 (d, $^1J_{PC}$ = 53.3 Hz), 36.3 (d, $^1J_{PC}$ = 56.7 Hz), 26.8, 26.3, 20.2;
^{31}P NMR (162 MHz, $CDCl_3$): δ_P 58.52;
FT-IR (ATR): ν_{max} /cm^{-1} 2962, 2873, 1731, 1557, 1475, 1442, 1350, 1327, 1235, 1198, 1165, 1126, 1053, 971, 910, 846, 832, 812, 796, 731, 702, 655, 561, 511, 450;
HRMS (ES+): calcd for $C_{16}H_{29}Cl_2N_4OP + H^+$ is 395.1534, found 395.1549 (+3.8 ppm).

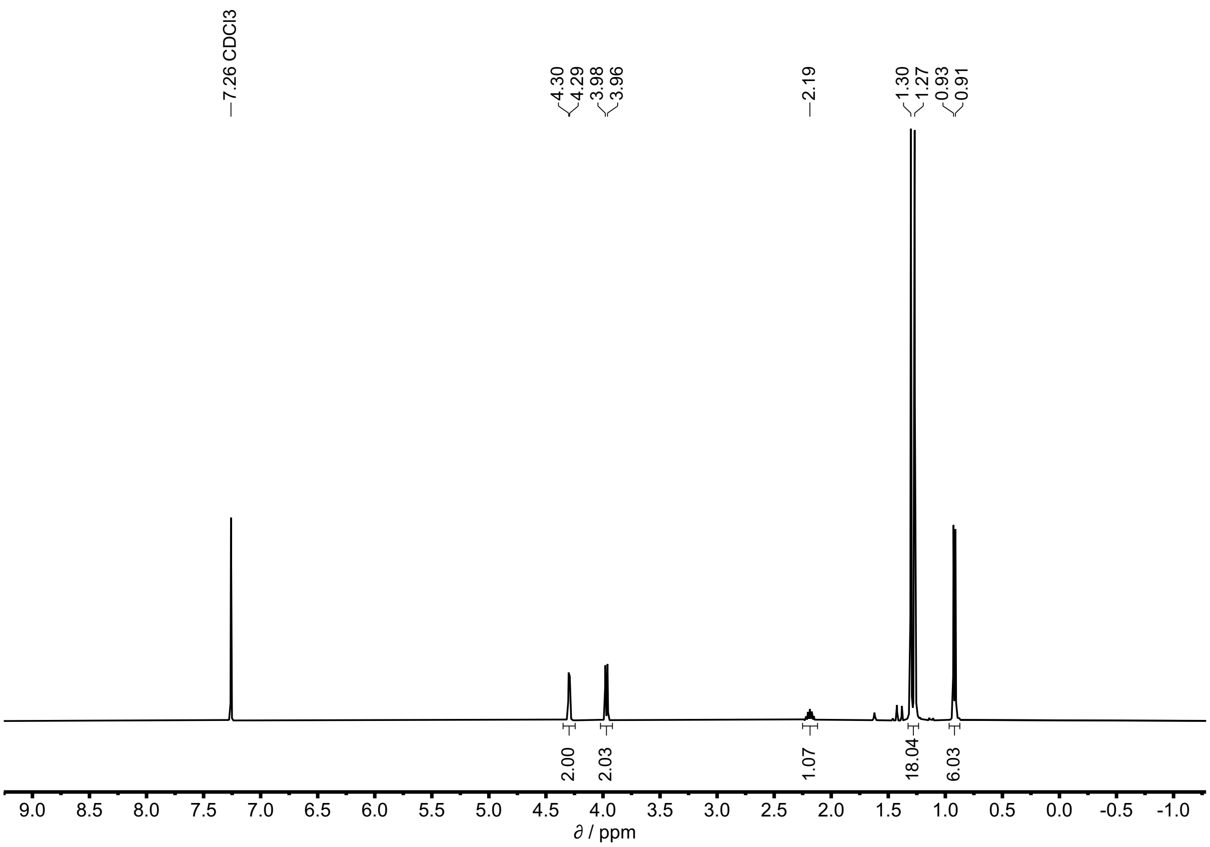

Figure S15. ^{1}H NMR spectrum (400 MHz, $CDCl_3$) of compound **9**.

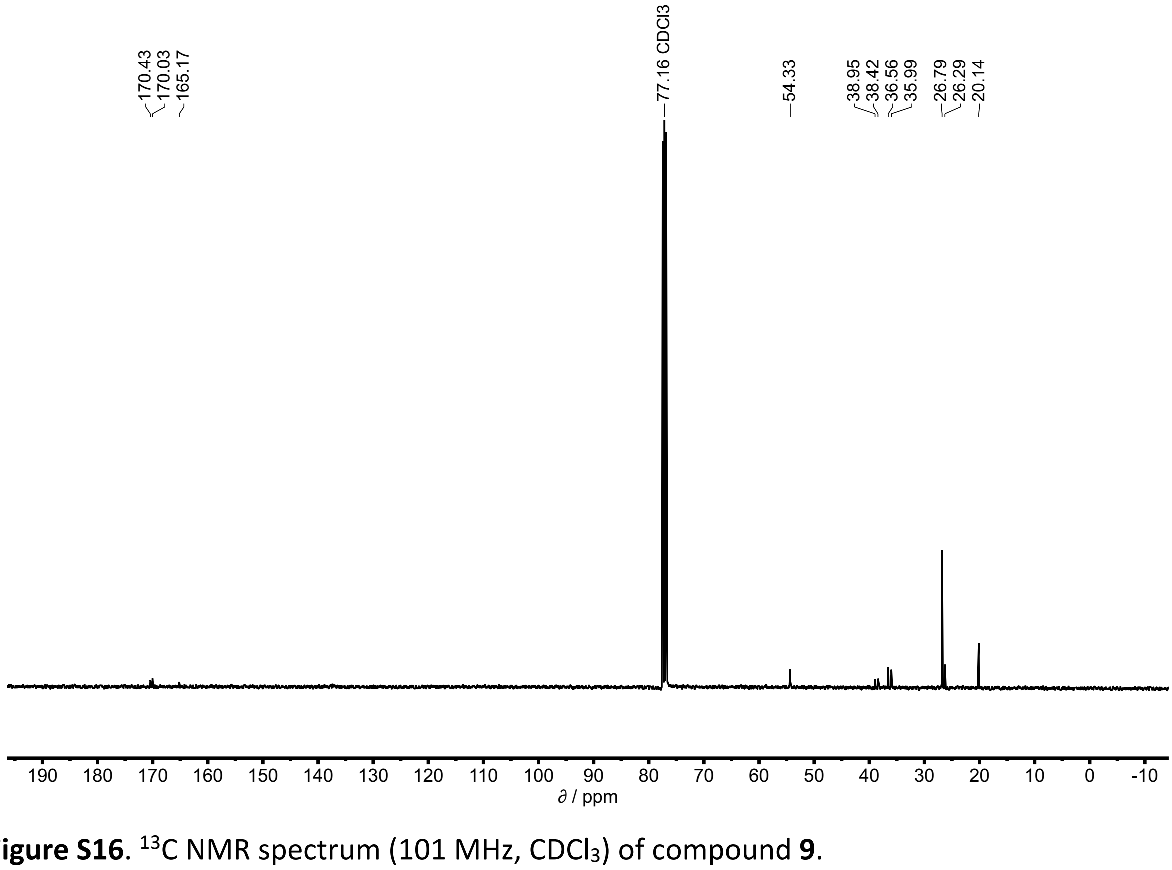

Figure S16. ^{13}C NMR spectrum (101 MHz, $CDCl_3$) of compound **9**.

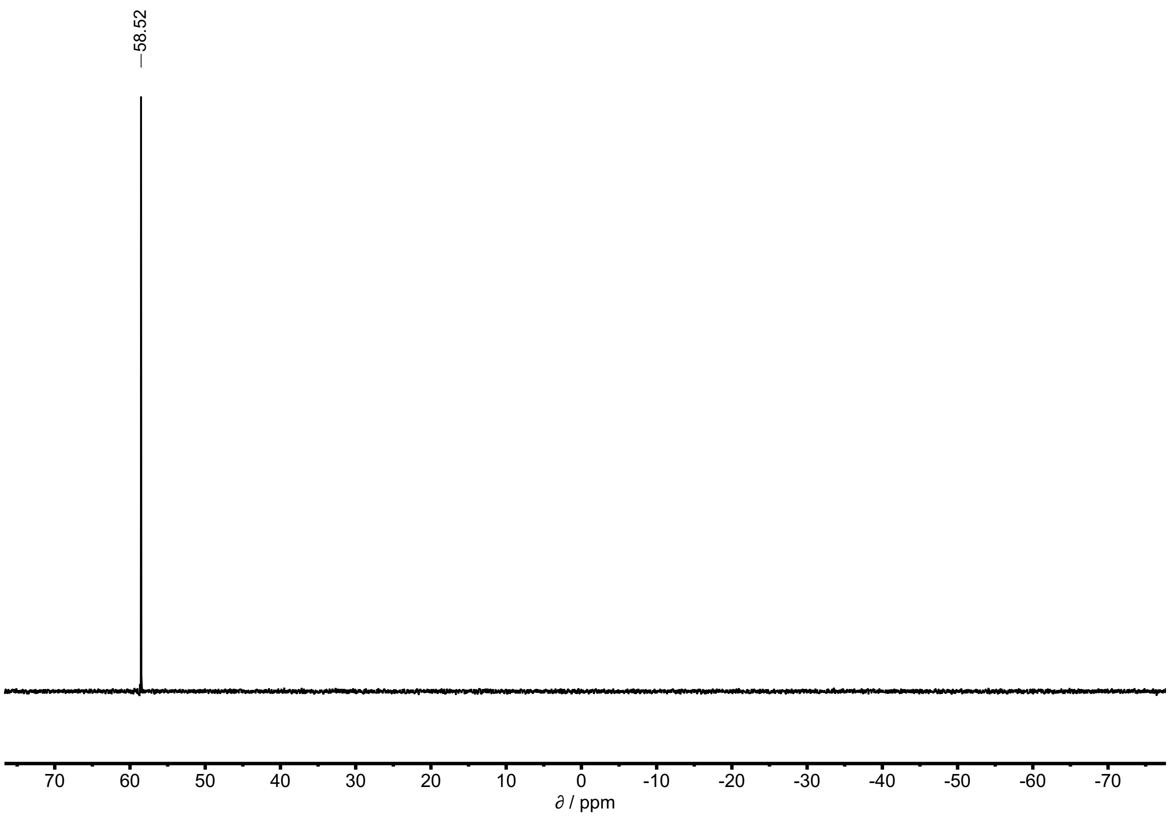

Figure S17. ^{31}P NMR spectrum (162 MHz, $CDCl_3$) of compound **9**.

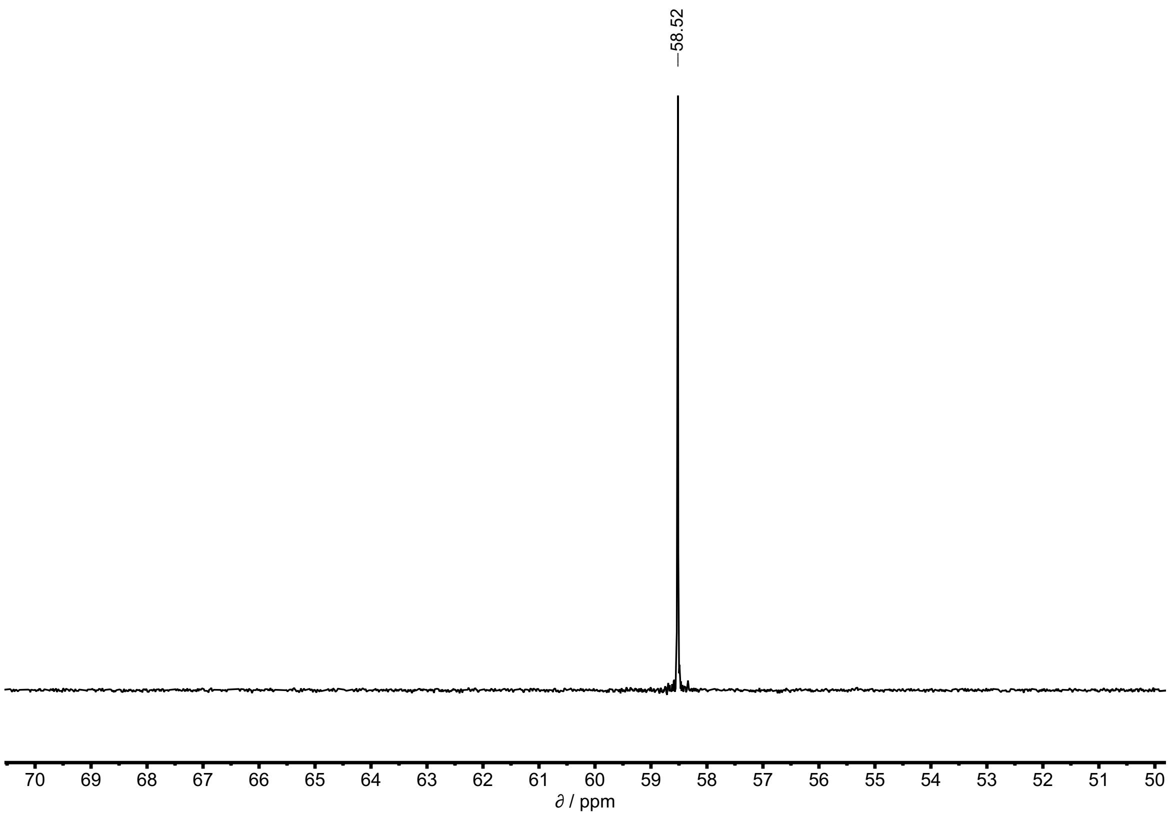

Figure S18. ^{31}P NMR spectrum (162 MHz, $CDCl_3$) of compound **9**.

Synthesis of **10**

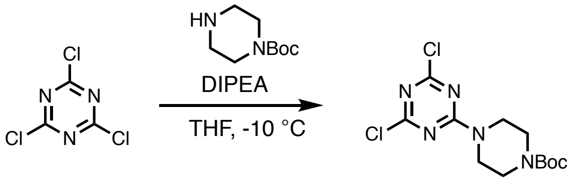

To a solution of cyanuric chloride (1.56 g, 8.46 mmol, 1.2 eq) in THF (25 mL) at -10 °C was added 1-Boc piperazine (1.31 g, 7.05 mmol, 1.0 eq) and DIPEA (2.5 mL, 1.8 g, 7.1 mmol, 2.0 eq) and the solution was stirred at -10 °C for 1h. The solvent was evaporated *in vacuo*, the residues were dissolved in EtOAc (40 mL) and the solution was washed with citric acid solution (5% in water, 3 x 15 mL). The aqueous wash was extracted with EtOAc (1 x 15 mL). The combined organic phases were dried ($MgSO_4$) and the solvent was evaporated *in vacuo*. The crude was purified by flash chromatography (SiO_2, 0-20% gradient of EtOAc in 40-60 Pet. Ether). The product was obtained as a white powder (1.66 g, 4.97 mmol, 70% yield).

^{1}H NMR (400 MHz, $CDCl_3$): δ_H 3.86 (t, *J* = 5.2 Hz, 4H), 3.51 (t, *J* = 5.2 Hz, 4H), 1.48 (s, 9H);
^{13}C NMR (101 MHz, $CDCl_3$): δ_C 170.63, 164.35, 154.55, 80.83, 44.13, 28.50;
FT-IR (ATR): ν_{max} /cm^{-1} 2973, 2927, 1696, 1574, 1470, 1419, 1362, 1348, 1327, 1282, 1265, 1232, 1156, 1133, 991, 837, 793, 771, 531;
HRMS (ES+): calcd for $C_{12}H_{17}Cl_2N_5O_2 + H^+$ is 334.0838, found 334.0835 (-0.9 ppm).

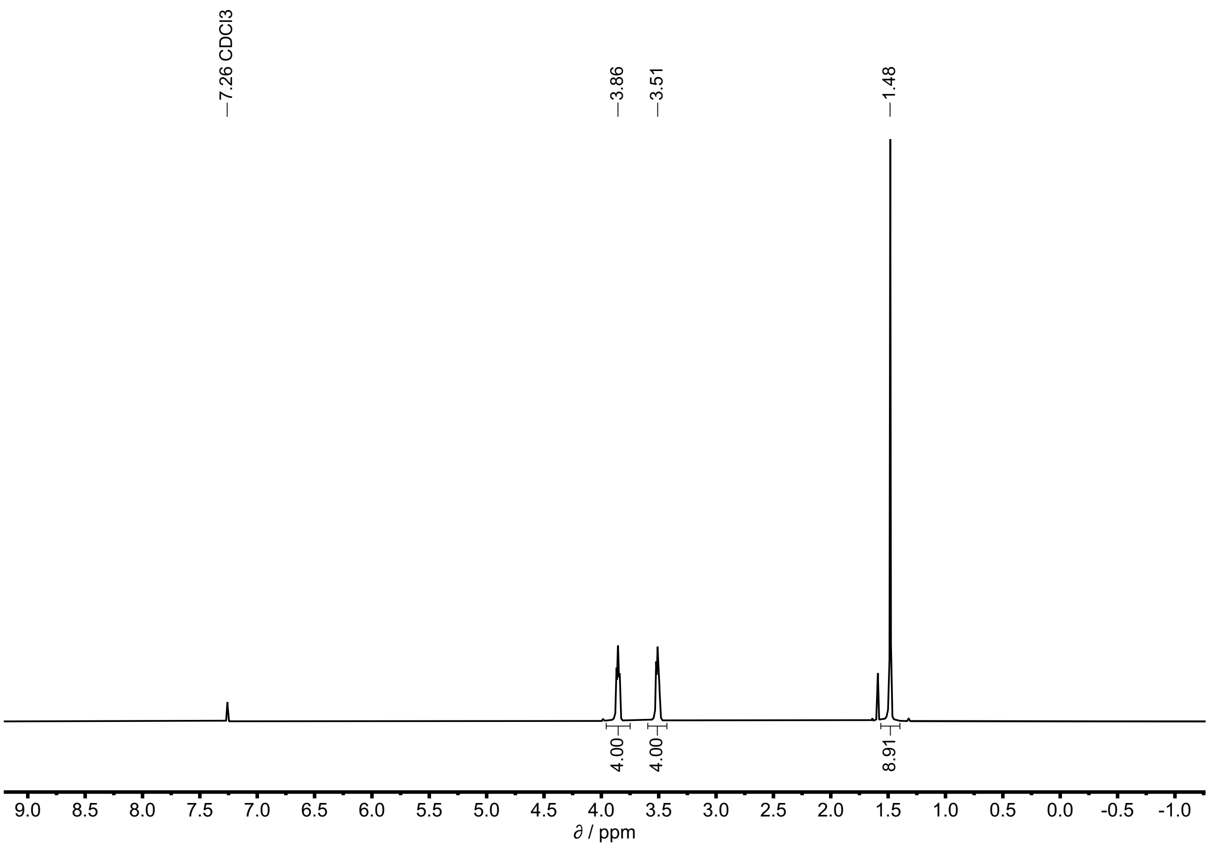

Figure S19. ^{1}H NMR spectrum (400 MHz, $CDCl_3$) of compound **10.**

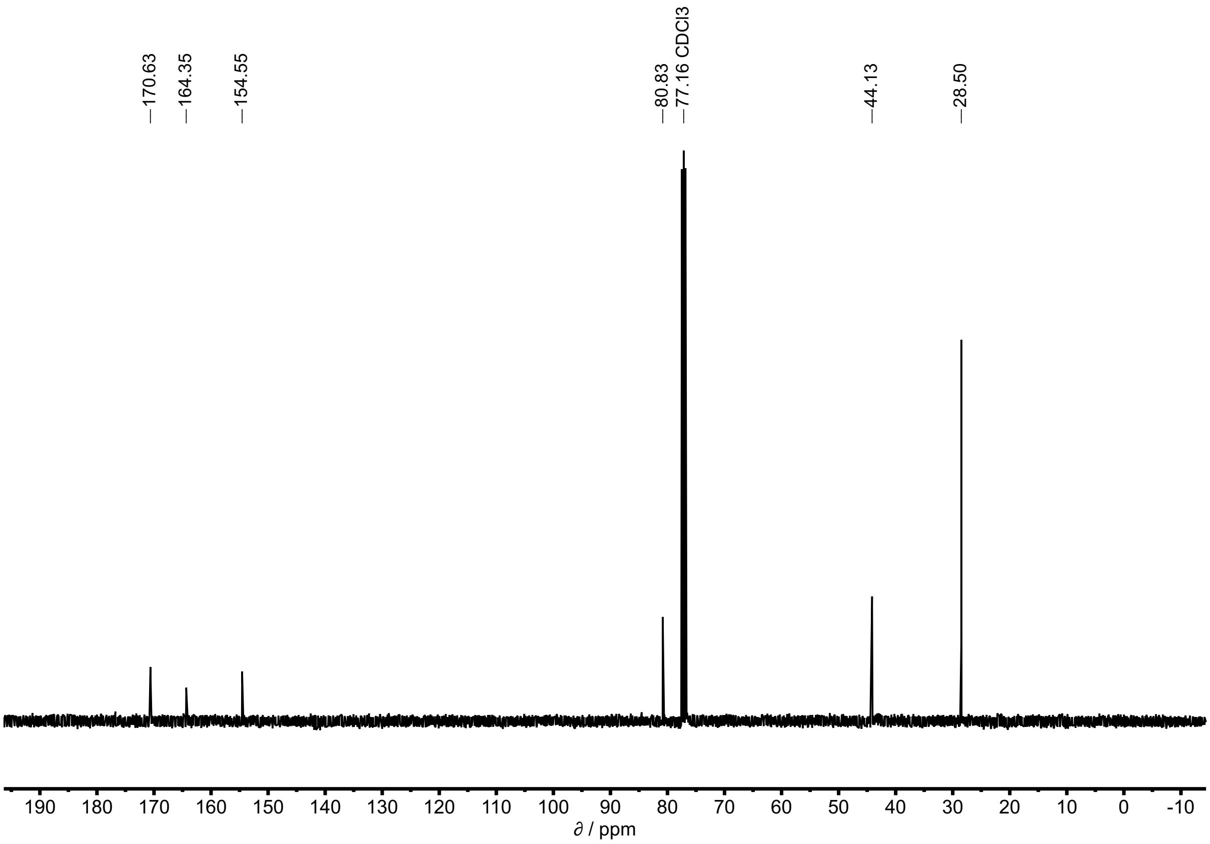

Figure S20. ^{13}C NMR spectrum (101 MHz, $CDCl_3$) of compound **10**.

Synthesis of **11**

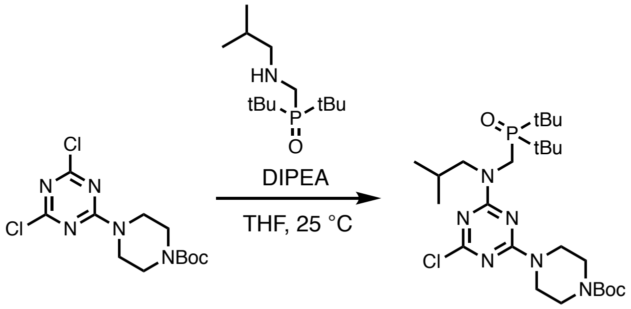

To a solution of **10** (338 mg, 1.01 mmol, 1.0 eq) in THF (10 mL) at 0 °C was added dropwise a solution of amine **4** (250 mg, 1.01 mmol, 1.0 eq) in THF (5 mL) followed by DIPEA (352 µL, 261 mg, 2.02 mmol, 2.0 eq). The reaction mixture was stirred for 15 h at 22 °C. The solvent was removed *in vacuo* and the residue was dissolved in EtOAc (35 mL). The solution was washed with a solution of citric acid (5% in water, 3 x 15 mL). The aqueous wash was extracted with EtOAc (15 mL). The combined organic phases were dried ($MgSO_4$) and the solvent was removed *in vacuo*. The crude was purified by flash chromatography (SiO_2, 80-100% gradient of EtOAc in 40-60 Pet. Ether) to obtain the product as a white solid (462 mg, 0.848 mmol, 84% yield).

^{1}H NMR (400 MHz, $CDCl_3$): δ_H 4.30 (m, 2H), 3.90 and 3.85 (d, *J* = 7.4 Hz, 2H, rotamers), 3.82 – 3.69 (m, 4H), 3.46 (t, *J* = 5.2 Hz, 4H), 2.17 (non, *J* = 6.8 Hz, 1H), 1.47 (s, 9H), 1.27 (d, $^3J_{PH}$ = 13.2 Hz, 18H), 0.89 (d, *J* = 6.7 Hz, 6H);
^{13}C NMR (101 MHz, $CDCl_3$): δ_C 169.2, 165.0, 164.5, 154.8, 80.35, 53.9 and 53.4 (rotamers), 43.4 (2 carbon atoms), 38.4 (d, $^1J_{CP}$ = 55.6 Hz), 36.1 (d, $^1J_{CP}$ = 55.9 Hz), 28.6, 26.8, 26.4, 20.57 and 20.19 (rotamers);
^{31}P NMR (162 MHz, $CDCl_3$): δ_P 58.60, 58.16;
FT-IR (ATR): ν_{max} /cm^{-1} 2959, 2932, 2869, 1704, 1568, 1487, 1439, 1418, 1364, 1307, 1265, 1236, 1212, 1162, 1136, 1000, 981, 829, 812, 798, 505, 448;
HRMS (ES+): calcd for $C_{25}H_{46}ClN_6O_3P + H^+$ is 545.3136, found 545.3136 (0.0 ppm).

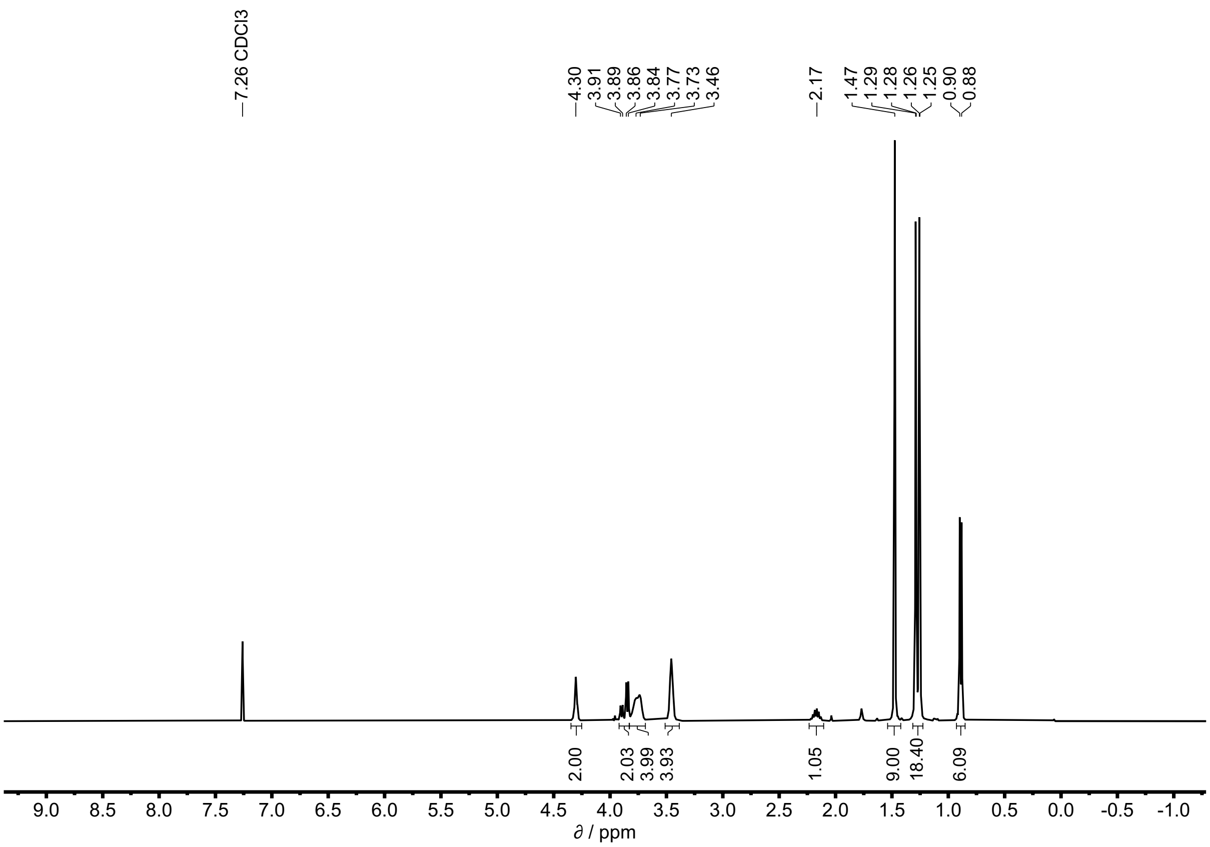

Figure S21. ^{1}H NMR spectrum (400 MHz, $CDCl_3$) of compound **11**.

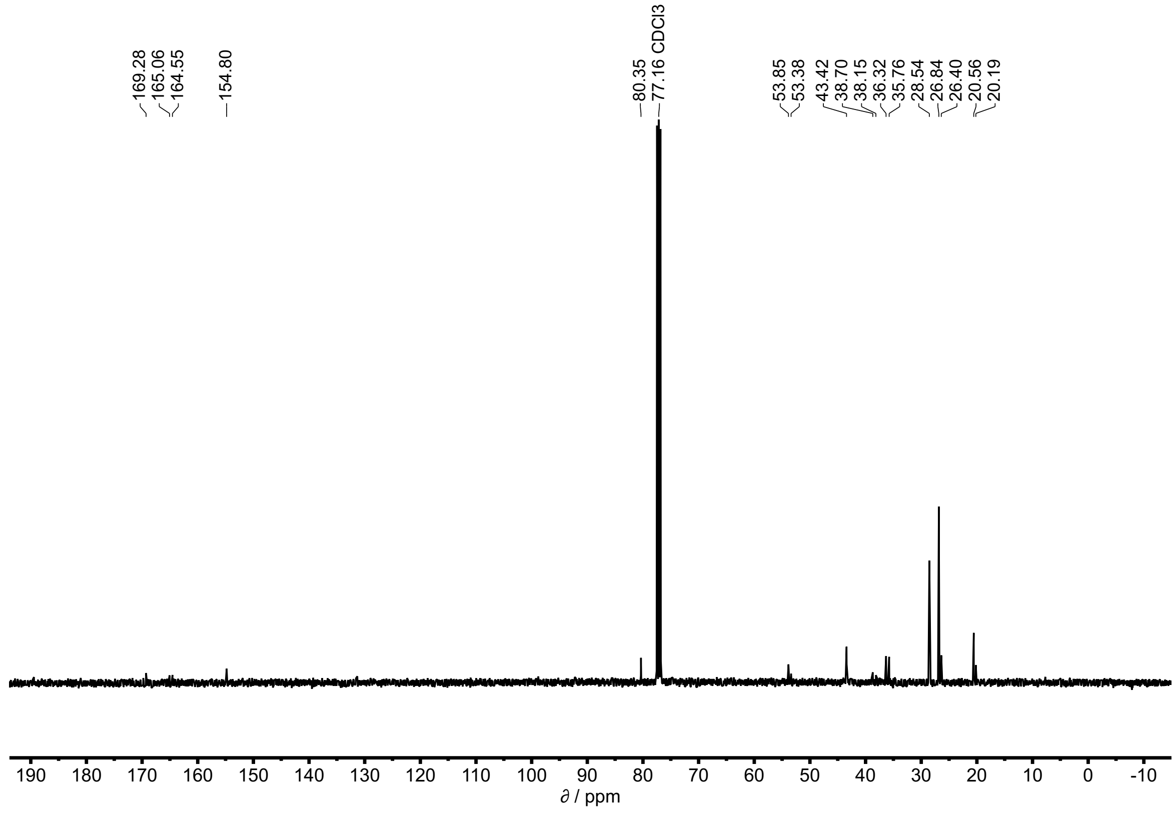

Figure S22. ^{13}C NMR spectrum (101 MHz, $CDCl_3$) of compound **11**.

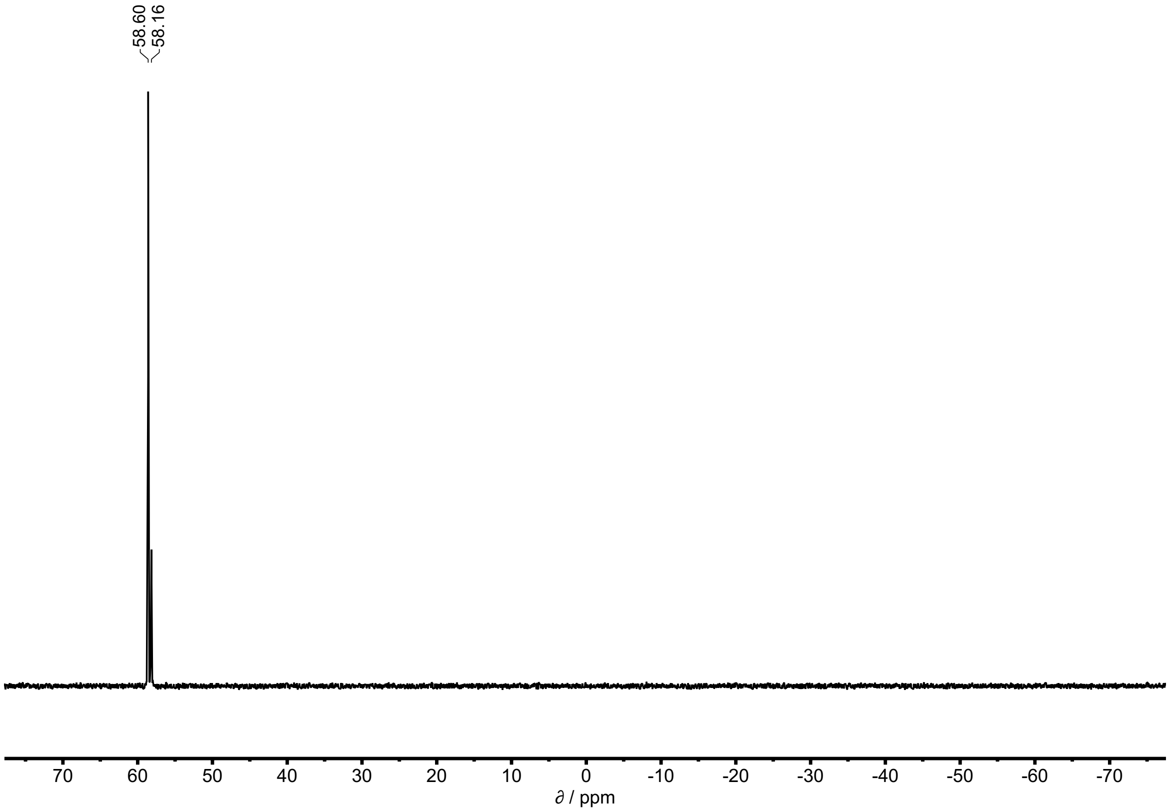

Figure S23. ^{31}P NMR spectrum (162 MHz, $CDCl_3$) of compound **11**.

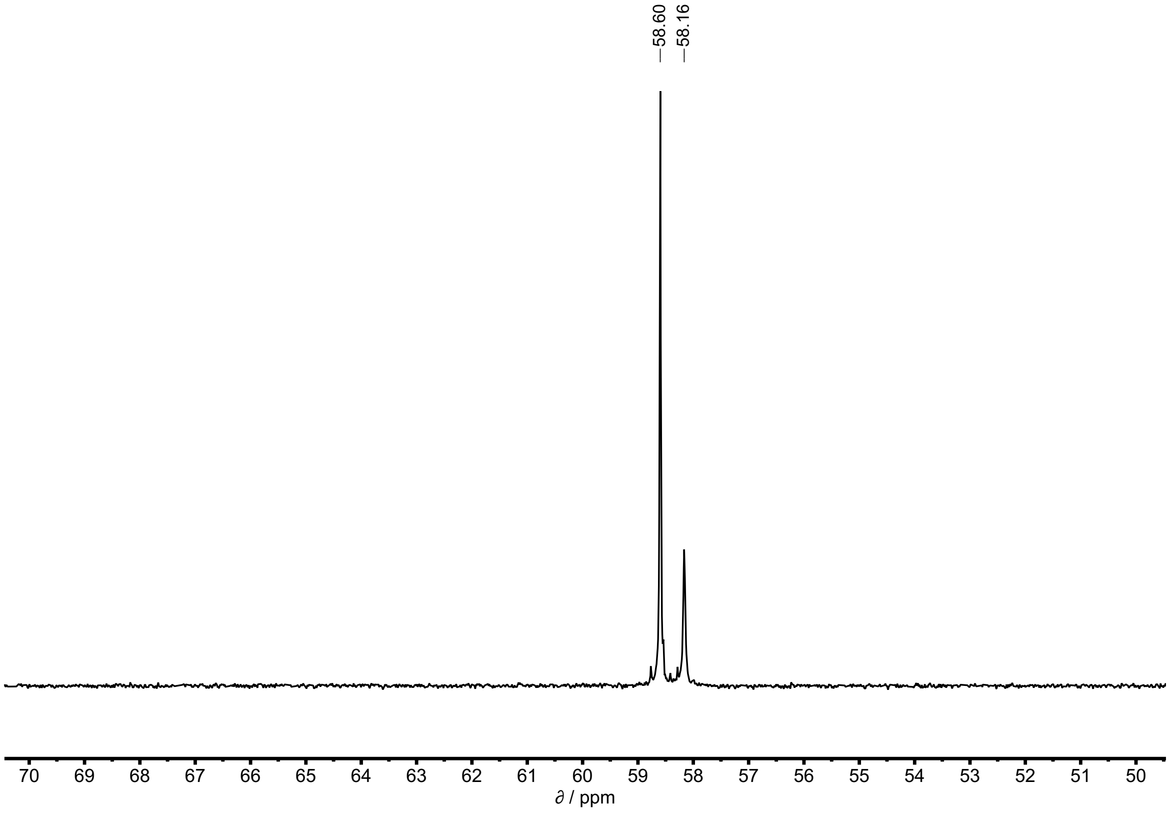

Figure S24. ^{31}P NMR spectrum (162 MHz, $CDCl_3$) of compound **11**.

Synthesis of **12**

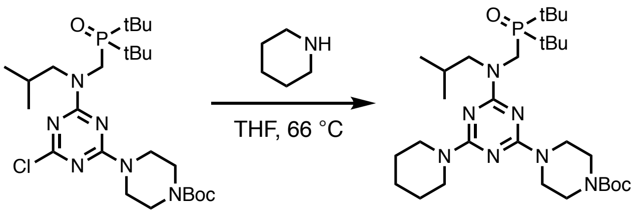

A solution of **11** (500 mg, 0.917 mmol, 1.0 eq) and piperidine (362 µL, 312 mg, 3.67 mmol, 4.0 eq) in THF (15 mL) was heated under reflux for 15 h. The solvent was removed *in vacuo* and the residue was dissolved in EtOAc (40 mL). The solution was washed with a solution of citric acid (5% in water, 3 x 15 mL). The aqueous wash was extracted with EtOAc (15 mL). The combined organic phases were dried ($MgSO_4$) and the solvent was evaporated *in vacuo*. The crude was purified by flash chromatography (SiO_2, 0-8% gradient of MeOH in DCM) to obtain the product as a transparent oil (517 mg, 0.871 mmol, 95% yield).

^{1}H NMR (400 MHz, $CDCl_3$): δ_H 4.38 (s, 2H), 3.82 (d, *J* = 7.4 Hz, 2H), 3.71 (m, 8H), 3.42 (t, *J* = 5.2 Hz, 4H), 2.19 (m, 1H), 1.63 (m, *J* = 6.0 Hz, 2H), 1.53 (m, 4H), 1.47 (s, 9H), 1.27 (d, $^3J_{PH}$ = 12.9 Hz, 18H), 0.87 (d, *J* = 6.7 Hz, 6H);
^{13}C NMR (101 MHz, $CDCl_3$): 165.6 and 165.5, 165.5 and 165.2, 165.0 and 164.7, 155.0, 78.0 (br), 53.1, 44.2, 43.1, 38.2 (d, $^1J_{CP}$ = 59.6 Hz), 35.9 (d, $^1J_{CP}$ = 55.6 Hz), 28.6, 26.9, 26.6, 25.9, 25.1, 20.7;
^{31}P NMR (162 MHz, $CDCl_3$): δ_P 58.72, 58.64;
FT-IR (ATR): ν_{max} /cm^{-1} 2929, 2865, 2854, 1695, 1528, 1480, 1428, 1364, 1301, 1230, 1160, 1143, 1107, 1021, 997, 832, 805, 649, 509, 449;
HRMS (ES+): calcd for $C_{30}H_{56}N_7O_3P + H^+$ is 594.4261, found 594.4257 (-0.7ppm).

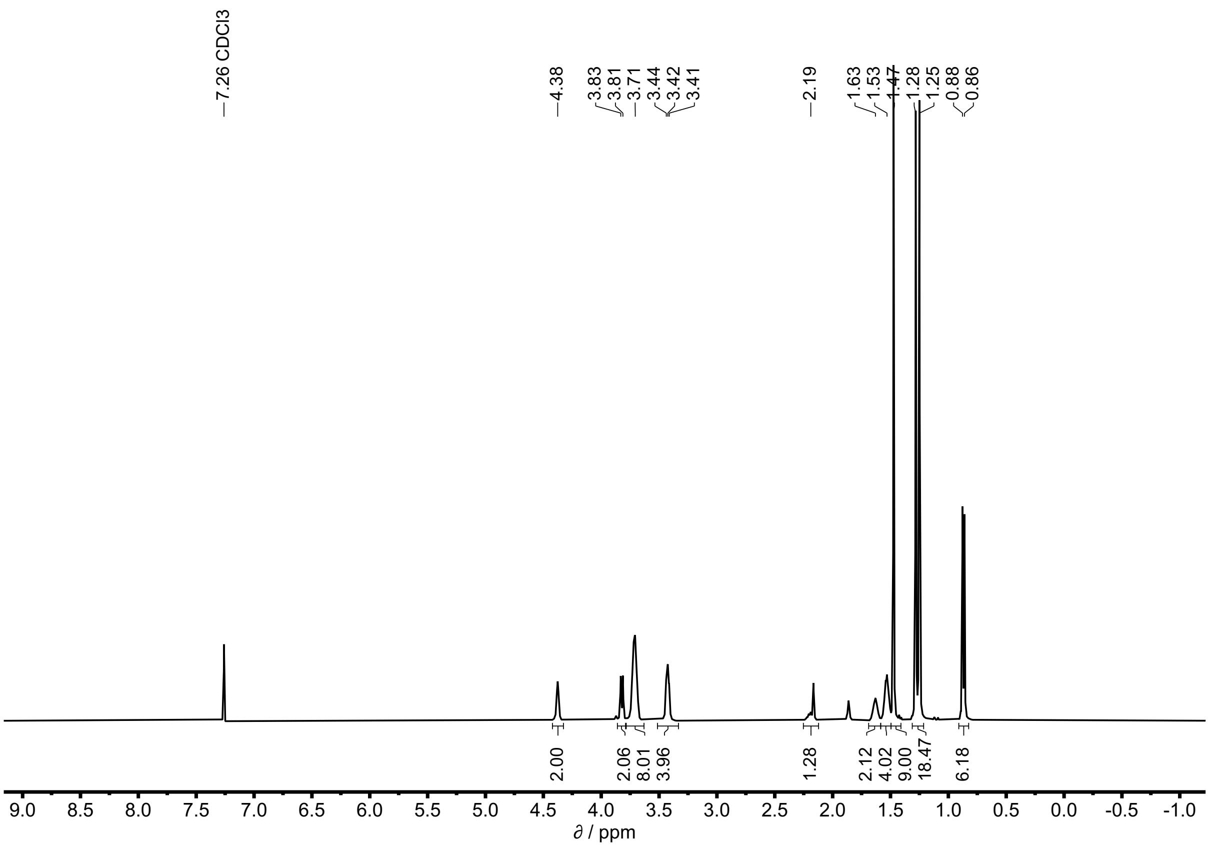

Figure S25. ^{1}H NMR spectrum (400 MHz, $CDCl_3$) of compound **12.**

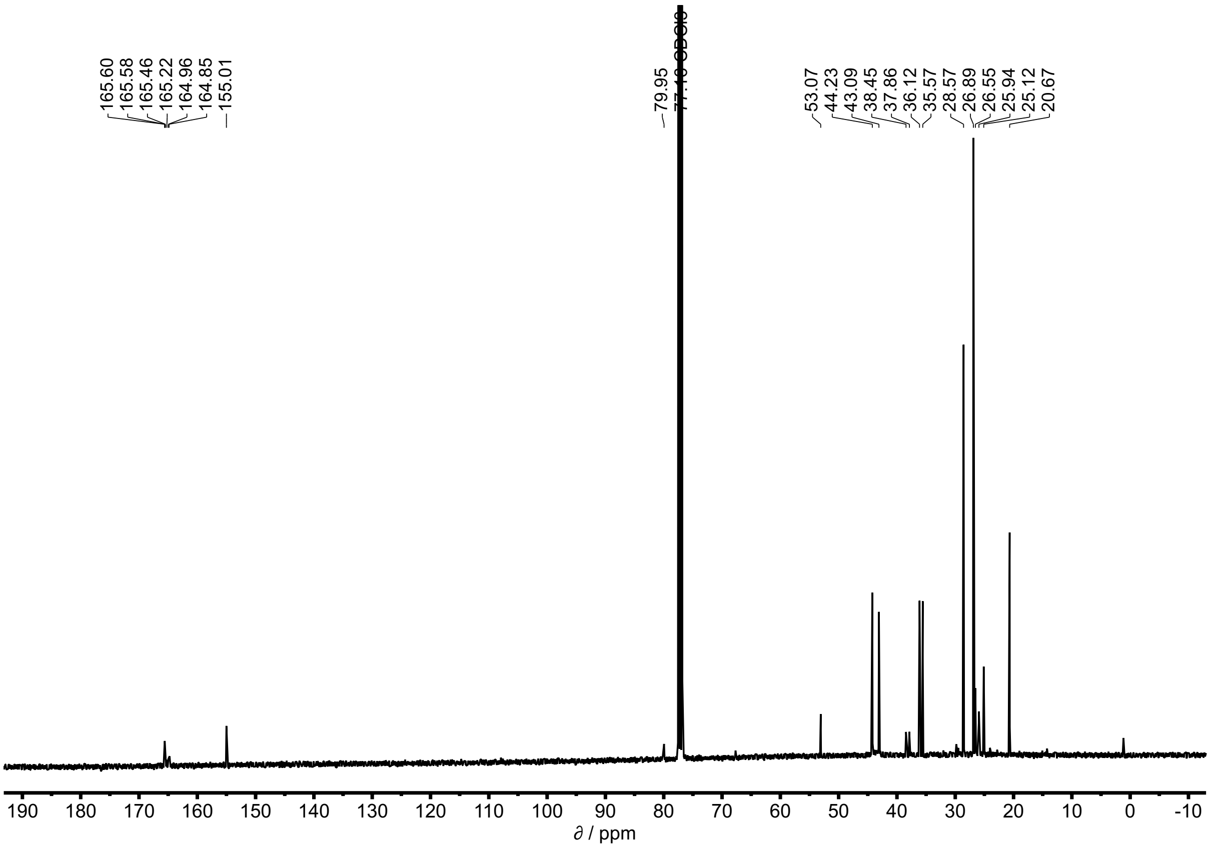

Figure S26. ^{13}C NMR spectrum (101 MHz, $CDCl_3$) of compound **12**.

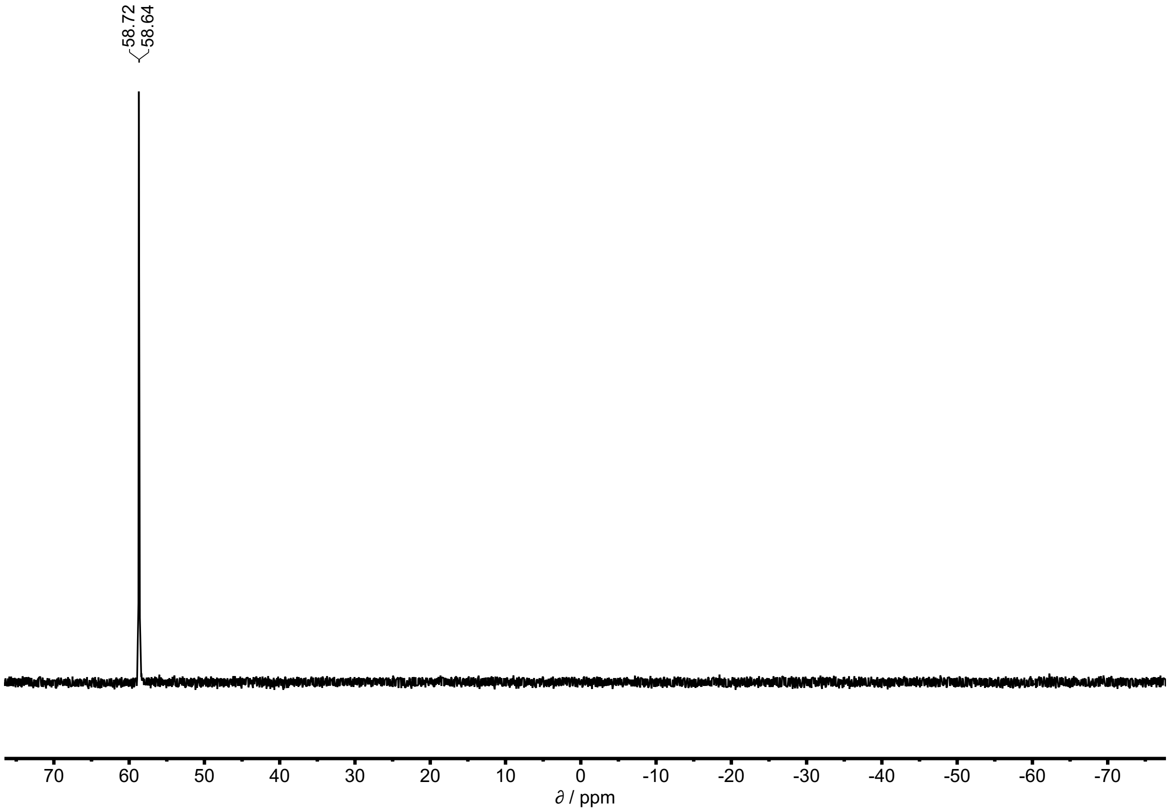

Figure S27. ^{31}P NMR spectrum (162 MHz, $CDCl_3$) of compound **12**.

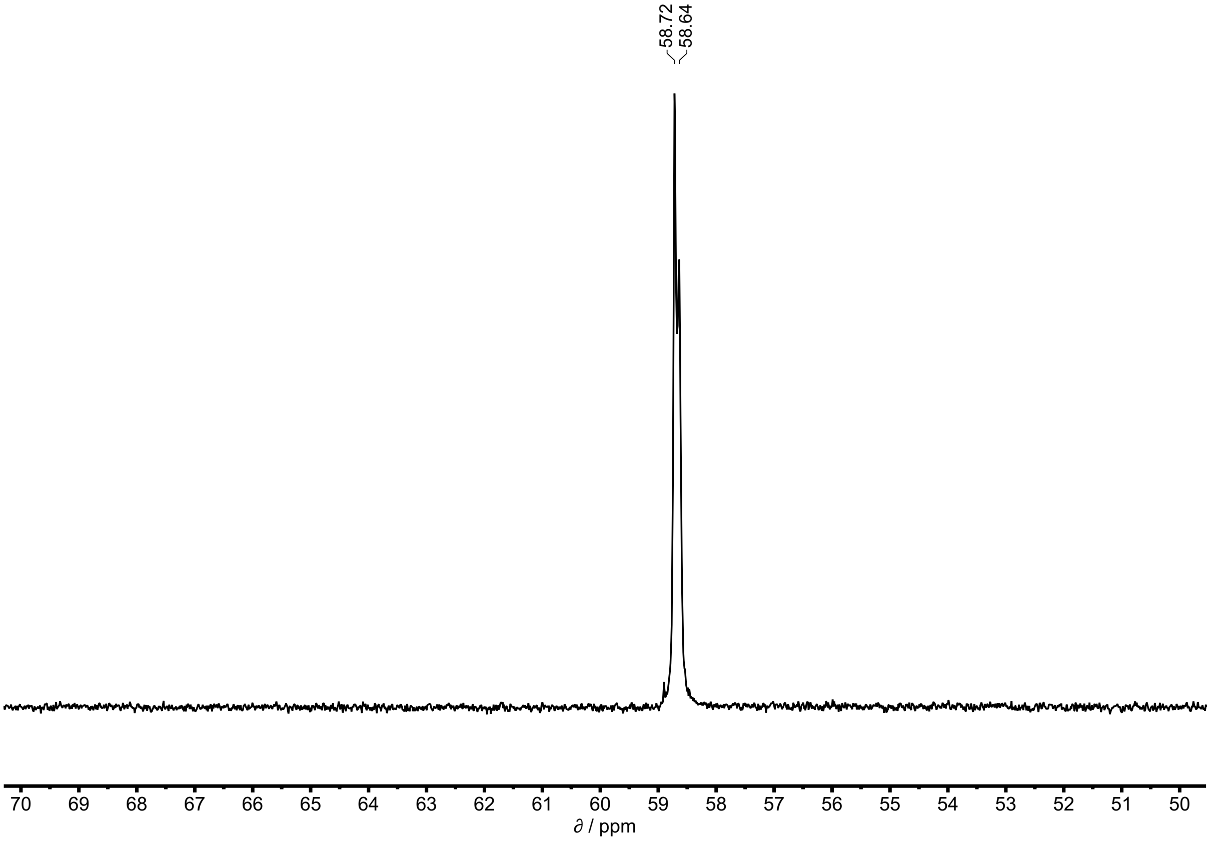

Figure S28. ^{31}P NMR spectrum (162 MHz, $CDCl_3$) of compound **12**.

Synthesis of **13**

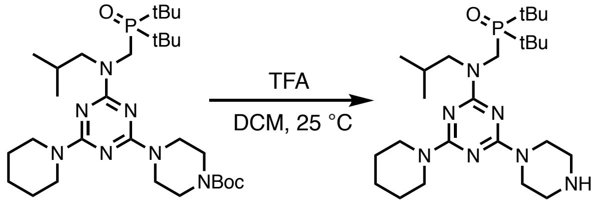

Trifluoroacetic acid (1.0 mL, 1.5 g, 13 mmol, 19 eq) was added dropwise to a solution of **12** (400 mg, 0.673 mmol, 1.0 eq) in DCM (20 mL) at 0 °C. The reaction mixture was stirred at 22 °C until complete conversion as monitored by LCMS. The reaction mixture was diluted with DCM (15 mL) and it was washed with NaOH solution (1 M in water, 3 x 15 mL). The aqueous wash was extracted with DCM (15 mL). The combined organic phases were dried ($MgSO_4$) and the solvent was removed *in vacuo*. The crude was purified by flash chromatography (SiO_2, 0-25% gradient of MeOH in DCM) to obtain the product as a colourless viscous oil (330 mg, 0.668 mmol, 99% yield).

Not all triazine carbons are visible in the ^{13}C NMR spectrum.

^{1}H NMR (400 MHz, $CDCl_3$): δ_H 4.38 (br d, *J* = 2.9 Hz, 2H), 3.81 (d, *J* = 7.3 Hz, 2H), 3.71 (m, 8H), 2.86 (t, *J* = 5.1 Hz, 4H), 2.19 (non, *J* = 6.9 Hz, 1H), 2.06 (br s, 1H, overlaps with HDO), 1.62 (m, 2H), 1.52 (m, 4H), 1.26 (d, $^3J_{PH}$ = 12.9 Hz, 18H), 0.87 (d, *J* = 6.7 Hz, 6H);
^{13}C NMR (101 MHz, $CDCl_3$): δ_C 165.6, 53.1, 46.1, 44.4, 44.2, 38.18 (d, $^1J_{PC}$ = 59.6 Hz), 35.9 (d, $^1J_{PC}$ = 55.4 Hz), 26.9, 26.6, 26.0 (br, rotamers), 25.2, 20.7;
^{31}P NMR (162 MHz, $CDCl_3$): δ_P 58.83;
FT-IR (ATR): ν_{max} /cm^{-1} 2931, 2851, 1525, 1479, 1428, 1367, 1298, 1235, 1206, 1136, 1092, 1020, 997, 832, 805, 648, 487, 448;
HRMS (ES+): calcd for $C_{25}H_{48}N_7OP$ + $2H^+$ is 247.6901, found 247.6902 (+0.2ppm).

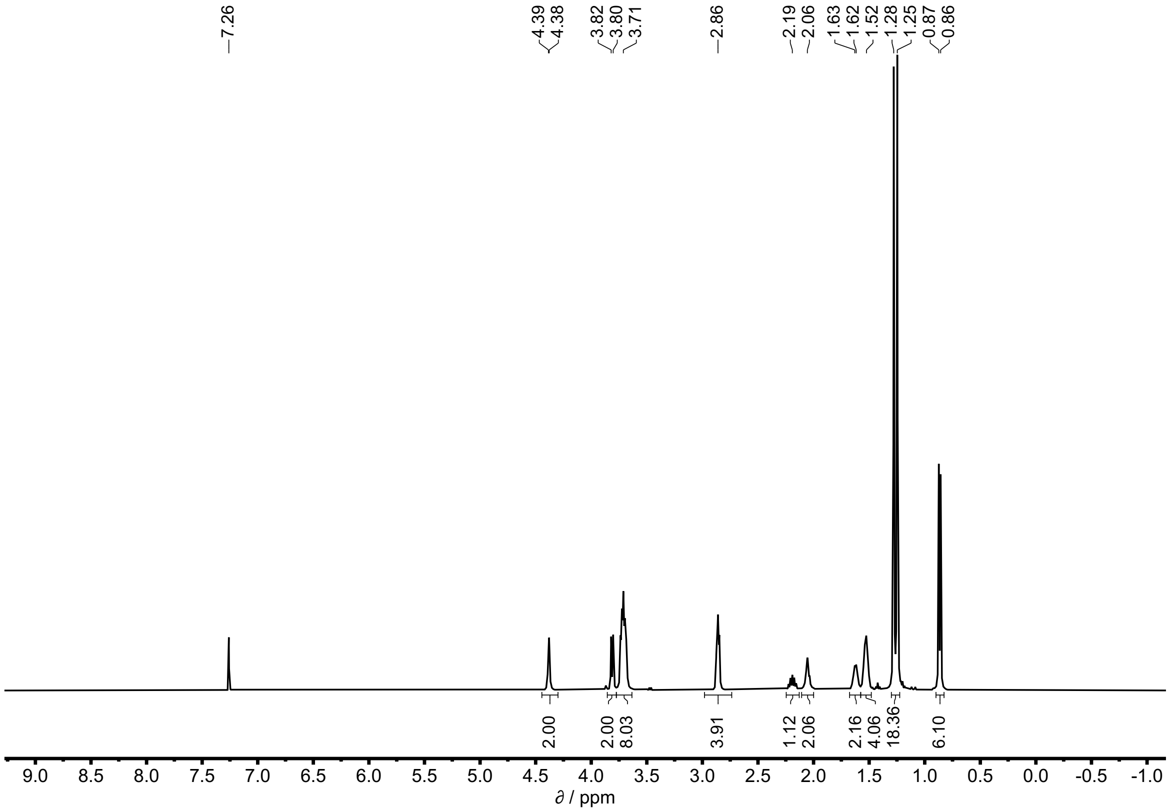

Figure S29. ^{1}H NMR spectrum (400 MHz, $CDCl_3$) of compound **13.**

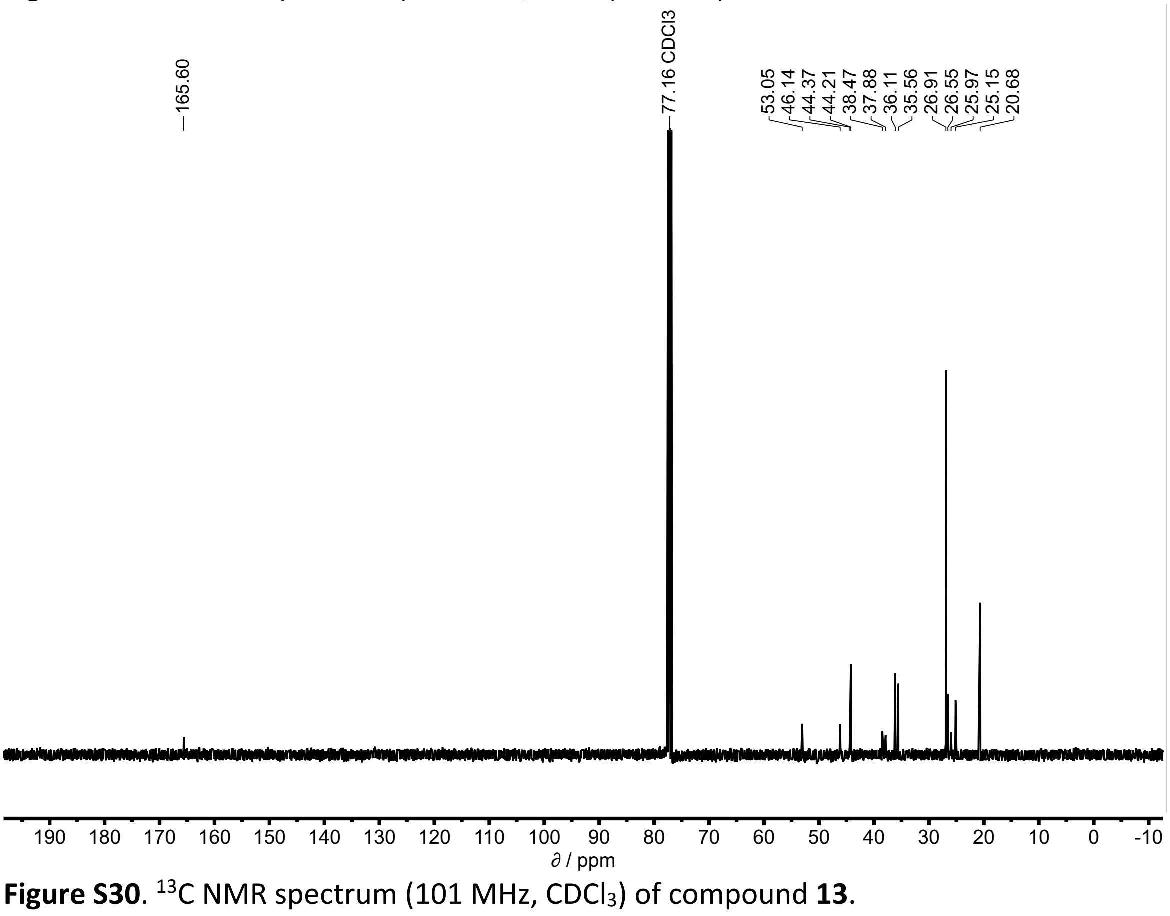

Figure S30. ^{13}C NMR spectrum (101 MHz, $CDCl_3$) of compound **13**.

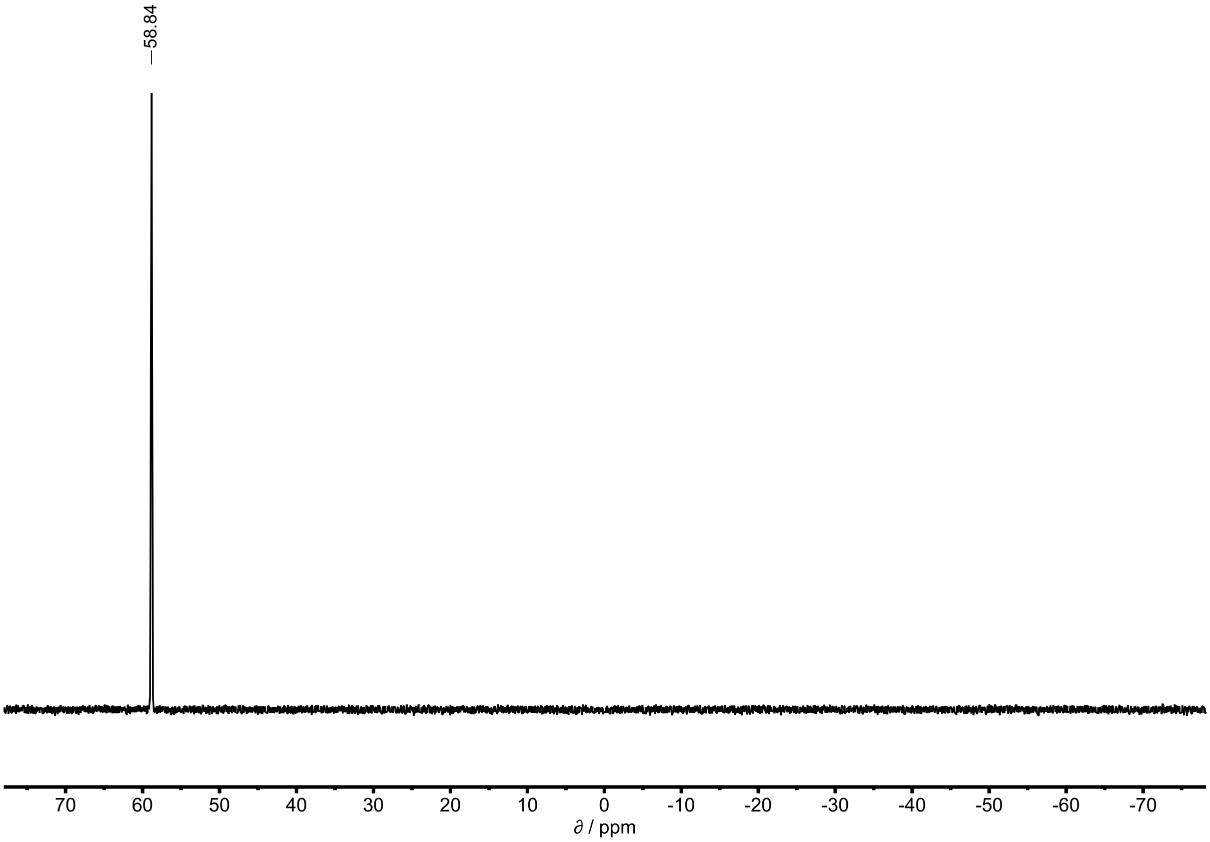

Figure S31. ^{31}P NMR spectrum (162 MHz, $CDCl_3$) of compound **13**.

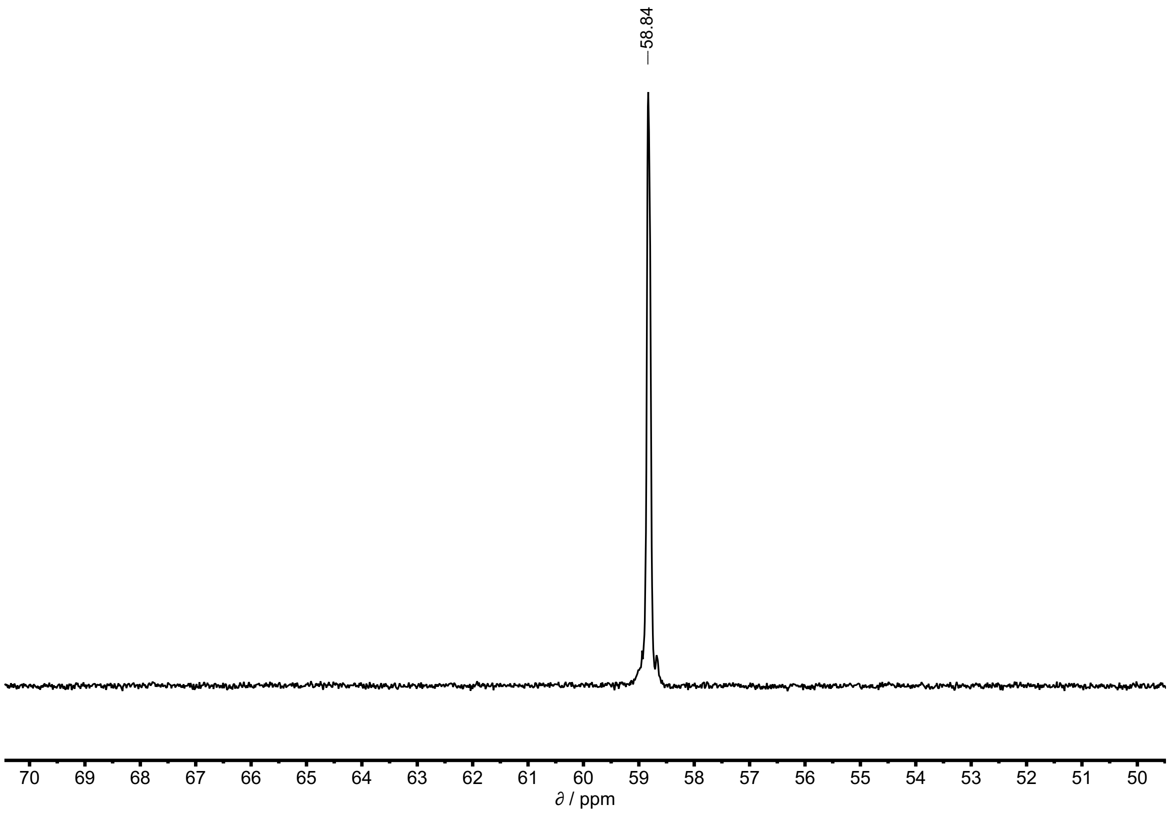

Figure S32. ^{31}P NMR spectrum (162 MHz, $CDCl_3$) of compound **13**.

Synthesis of reference compound

Synthesis of **14**

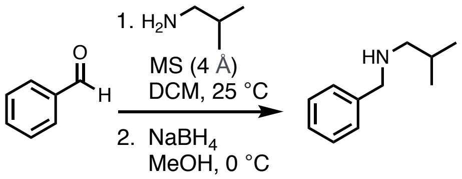

Isobutyl amine (3.1 mL, 2.28 g, 31.2 mmol, 1.1 eq) and molecular sieves (4 Å) were added to a solution of benzaldehyde (3.00 g, 28.3 mmol, 1.0 eq) in DCM (20 mL) at room temperature. The solution was stirred at room temperature until complete conversion of the aldehyde, which was monitored by ^{1}H NMR. The molecular sieves were removed and the solvent was evaporated *in vacuo* to obtain a colourless oil.

MeOH (20 mL) was added and the solution was cooled down to 0 °C, before sodium borohydride (1.2 g, 31.7 mmol, 1.1 eq) was added. The reaction mixture was stirred at room temperature until the disappearance of the imine intermediate, as monitored by ^{1}H NMR. The solvent was removed *in vacuo* and the residue was dissolved in NaOH solution (1M in water, 80 mL). The solution was extracted with DCM (3 x 30 mL). The organic phase was dried ($MgSO_4$) and the solvent was evaporated *in vacuo.* The crude was purified by flash chromatography (SiO_2, 0-10% gradient of MeOH in DCM) to yield the product as a transparent oil (3.92 g, 24.0 mmol, 85% yield).

^{1}H NMR (400 MHz, $CDCl_3$): δ_H 7.33 (m, 4H), 7.29 – 7.21 (m, 1H), 3.79 (s, 2H), 2.45 (d, *J* = 6.7 Hz, 2H), 1.77 (non, *J* = 6.7 Hz, 1H), 1.29 (s, 1H), 0.92 (d, *J* = 6.7 Hz, 6H);
^{13}C NMR (101 MHz, $CDCl_3$): δ_C 140.9, 128.5, 128.2, 126.9, 57.7, 54.3, 28.5, 20.8;
FT-IR (ATR): ν_{max} /cm^{-1} 3027, 2953, 2870, 2808, 1495, 1453, 1385, 1364, 1114, 1075, 1028, 731, 696, 598, 459;
HRMS (ES+): calcd for $C_{11}H_{17}N + H^+$ is 164.1439, found 164.1434 (-3.0 ppm).

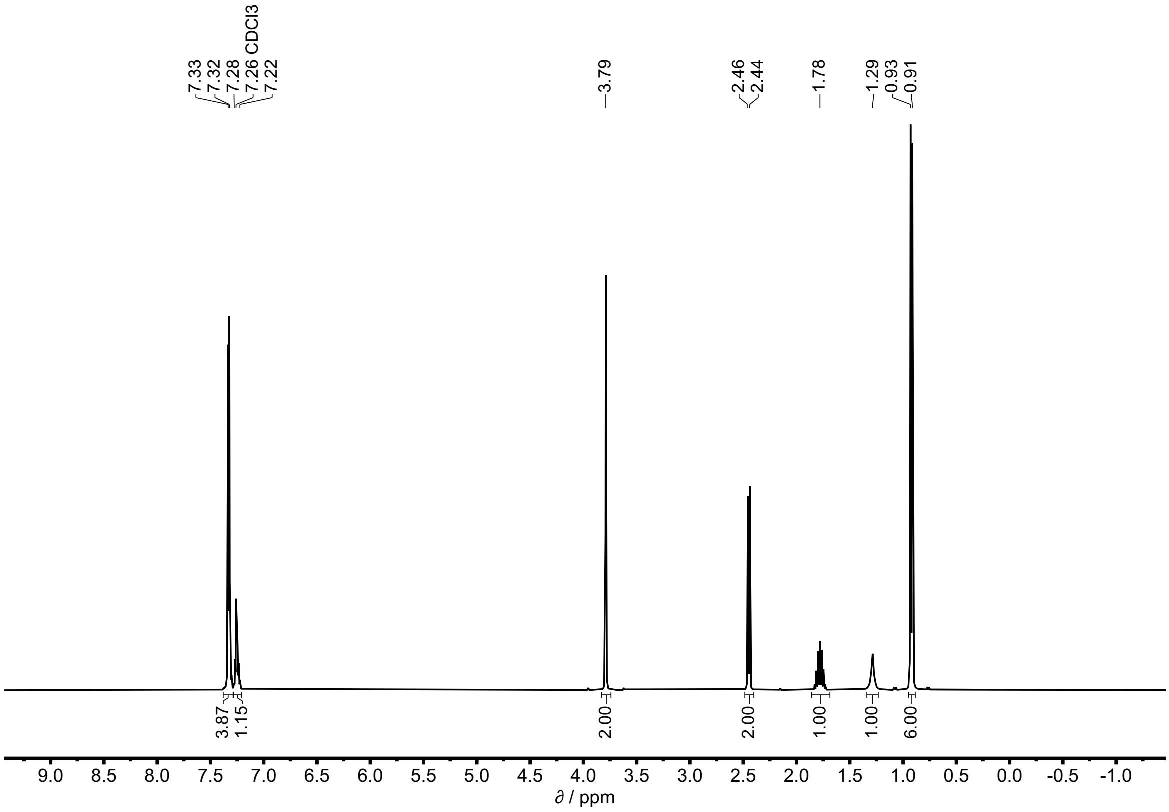

Figure S33. ^{1}H NMR spectrum (400 MHz, $CDCl_3$) of compound **14**.

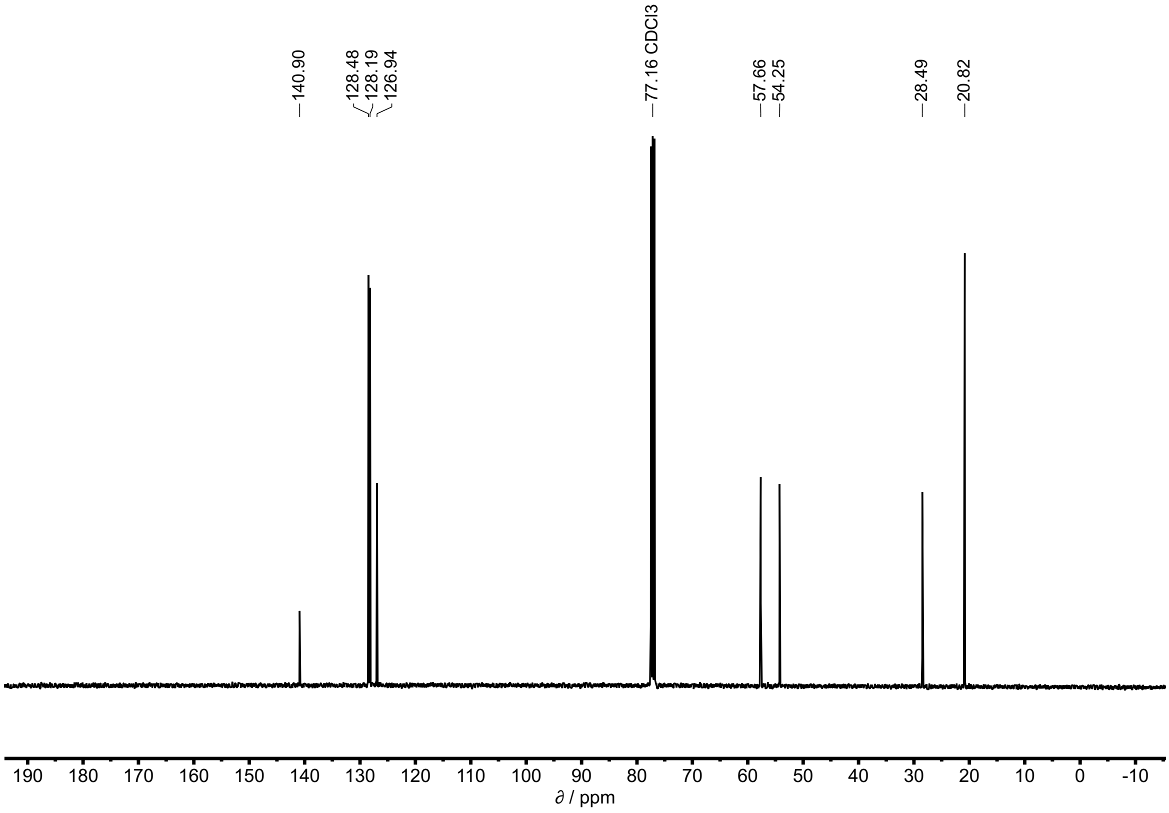

Figure S34. ^{13}C NMR spectrum (101 MHz, $CDCl_3$) of compound **14**.

Synthesis of **15**

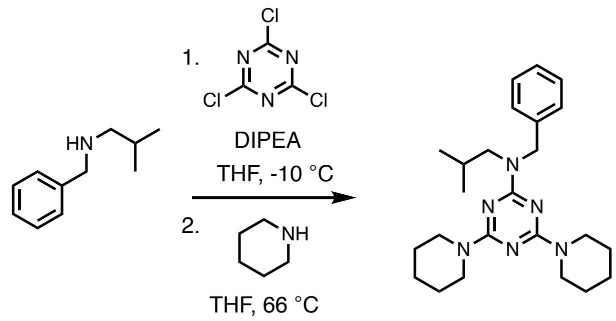

To a solution of cyanuric chloride (226 mg, 1.23 mmol, 1.1 eq) in THF (25 mL) at -10 °C was added dropwise a solution of amine **14** (182 mg, 1.12 mmol, 1.0 eq) in THF (10 mL), followed by DIPEA (0.39 mL, 289 mg, 2.24 mmol, 2.0 eq). The solution was stirred at -10 °C for 1h. The solvent was evaporated *in vacuo*, and the residues were dissolved in EtOAc (30 mL). The solution was washed with citric acid solution (5% in water, 3 x 15 mL). The aqueous phase was extracted with EtOAc (1 x 20 mL). The combined organic phases were dried ($MgSO_4$) and the solvent was evaporated *in vacuo* to yield the crude (325.2 mg).
A fraction of the thus obtained crude (120 mg, 0.385 mmol assuming full purity, 1.0 eq) was dissolved in dry THF (10 mL) and piperidine (152 µL, 131 mg, 1.54 mmol, 4.0 eq) was added. The reaction mixture was heated under reflux for 15 h. The solvent was evaporated *in vacuo*, and the residues were dissolved in EtOAc (30 mL). The solution was washed with citric acid solution (5% in water, 3 x 15 mL). The aqueous phase was extracted with EtOAc (1 x 20 mL). The combined organic phases were dried ($MgSO_4$) and the solvent was evaporated *in vacuo.* The crude was purified by flash chromatography (SiO_2, 0-3% gradient of EtOAc in 40-60 Pet. Ether). The product was obtained as a colourless oil (71.6 mg, 0.175 mmol, 89% yield over two steps).

^{1}H NMR (400 MHz, CDCl$_3$): δ_H 7.27 (m, 4H), 7.25 – 7.19 (m, 1H), 4.85 (s, 2H), 3.77-3.65 (m, 8H), 3.32 (d, *J* = 7.3 Hz, 2H), 2.10 (non, *J* = 6.8 Hz, 1H), 1.68 – 1.46 (m, 12H), 0.89 (d, *J* = 6.7 Hz, 6H);
^{13}C NMR (101 MHz, CDCl$_3$): δ_C 166.5, 165.5, 140.0, 128.3, 127.9, 126.7, 53.5, 50.3, 44.2, 27.5, 26.0, 25.3, 20.7;
FT-IR (ATR): ν_{max} /cm^{-1} 2929, 2851, 1525, 1478, 1459, 1423, 1385, 1372, 1343, 1295, 1271, 1232, 1209, 1129, 1096, 1023, 989, 975, 953, 907, 852, 805, 729, 697;
HRMS (ES+): calcd for $C_{24}H_{36}N_6 + H^+$ is 409.3080, found 4409.3094 (+3.4 ppm).

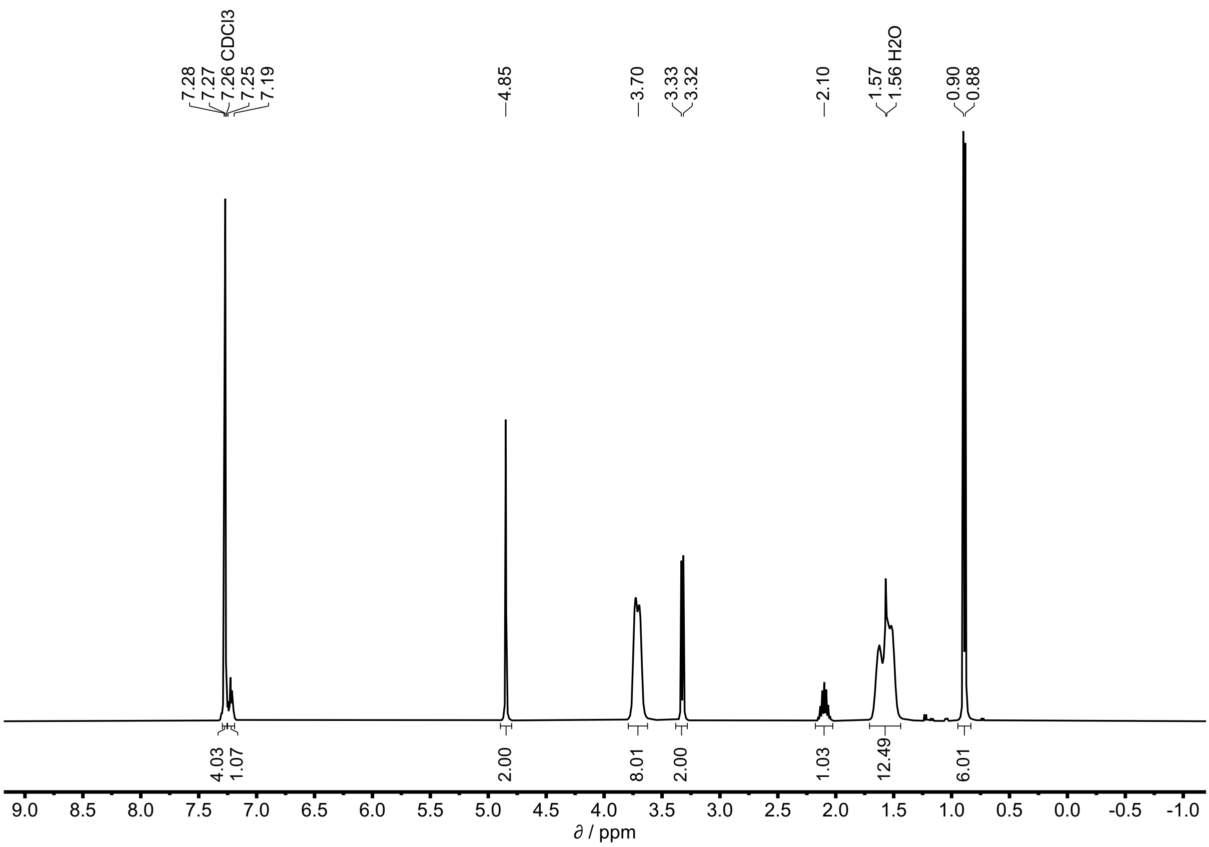

Figure S35. ^{1}H NMR spectrum (400 MHz, $CDCl_3$) of compound **15**.

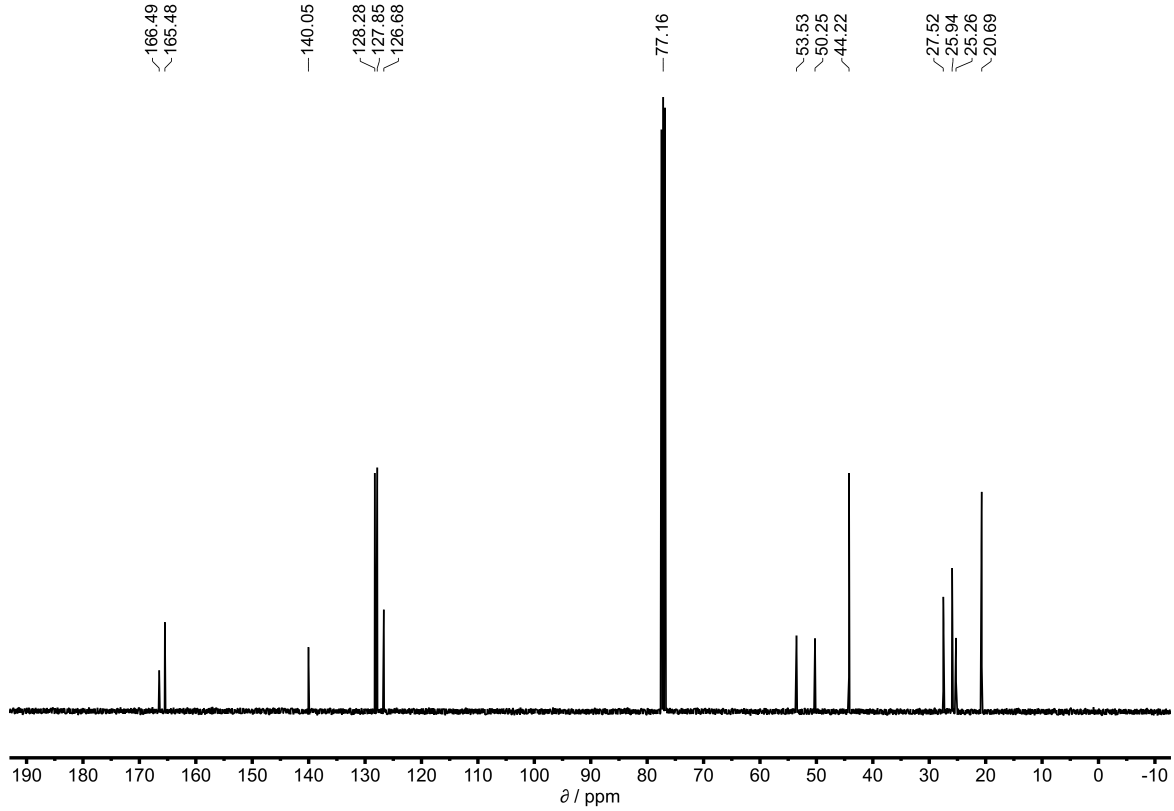

Figure S36. ^{13}C NMR spectrum (101 MHz, $CDCl_3$) of compound **15**.

Synthesis of 1-mers

Synthesis of **D**

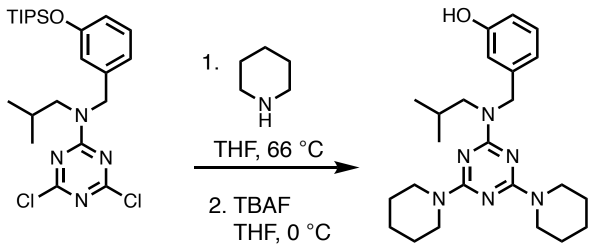

A solution of **5** (900 mg, 1.86 mmol, 1.0 eq) and piperidine (735 µL, 634 mg, 7.45 mmol, 4.0 eq) in THF (20 mL) was heated under reflux for 15 h. The solvent was evaporated *in vacuo* and the residue was dissolved in EtOAc (30 mL). The solution was washed with a solution of citric acid (5% in water, 3 x 15 mL). The aqueous wash was extracted with EtOAc (15 mL). The combined organic phases were dried ($MgSO_4$) and the solvent was evaporated *in vacuo* to yield a colourless oil.
To a solution of the obtained oil in THF (15 mL) at 0 °C was added dropwise a solution of TBAF (1M in THF, 3.72 mL, 3.72 mmol, 2.0 eq), and the reaction mixture was stirred at 22 °C until complete conversion of the starting material, as monitored by LCMS. The reaction mixture was diluted with ethyl EtOAc (40 mL) and washed with sat. aqueous ammonium chloride solution (2 x 15 mL). The organic phase was dried ($MgSO_4$) and evaporated *in vacuo*. The crude was purified by flash chromatography (SiO_2, 0-20% gradient of EtOAc in 40-60 Pet. Ether). The product was afforded as a white solid (710 mg, 1.67 mmol, 90% yield over 2 steps).

^{1}H NMR (400 MHz, $CDCl_3$): δ_H 7.12 (t, *J* = 7.8 Hz, 1H), 6.81 (d, *J* = 7.6 Hz, 1H), 6.69 (br s, 1H), 6.65 (m, 1H), 4.90 (s, 1H), 4.78 (s, 2H), 3.70 (m, 8H), 3.30 (d, *J* = 7.2 Hz, 2H), 2.07 (non, *J* = 6.9 Hz, 1H), 1.67-1.45 (m, 12H, overlaps with HDO), 0.87 (d, *J* = 6.7 Hz, 6H);
^{13}C NMR (101 MHz, $CDCl_3$): δ_C 166.5, 165.5, 155.8, 142.0, 129.5, 120.2, 114.5, 113.7, 53.5, 50.0, 44.2, 27.5, 25.9, 25.2, 20.7;
FT-IR (ATR): ν_{max} /cm^{-1} 2931, 2852, 1590, 1524, 1484, 1459, 1441, 1385, 1372, 1344, 1299, 1272, 1233, 1206, 1129, 1097, 1024, 980, 954, 853, 806, 754, 697;
HRMS (ES+): calcd for $C_{24}H_{36}N_6O + H^+$ is 425.3029, found 425.3038 (2.1 ppm).

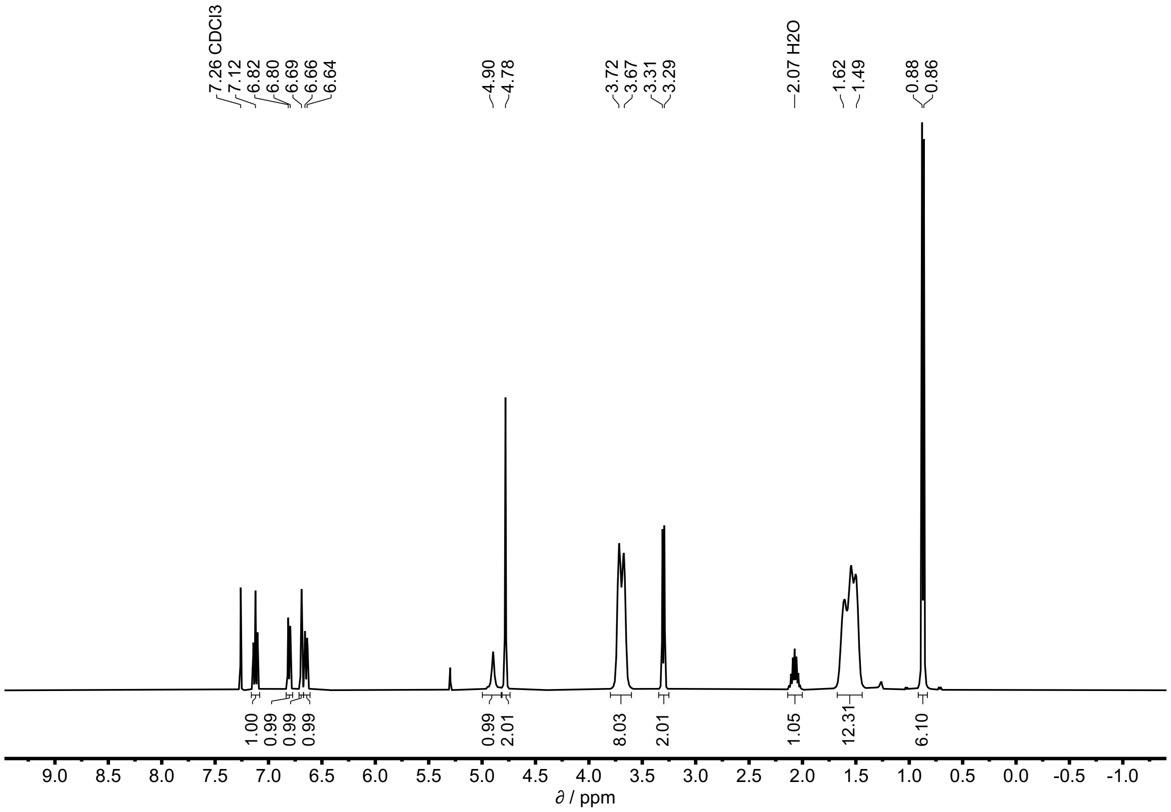

Figure S37. ^{1}H NMR spectrum (400 MHz, $CDCl_3$) of compound **D**.

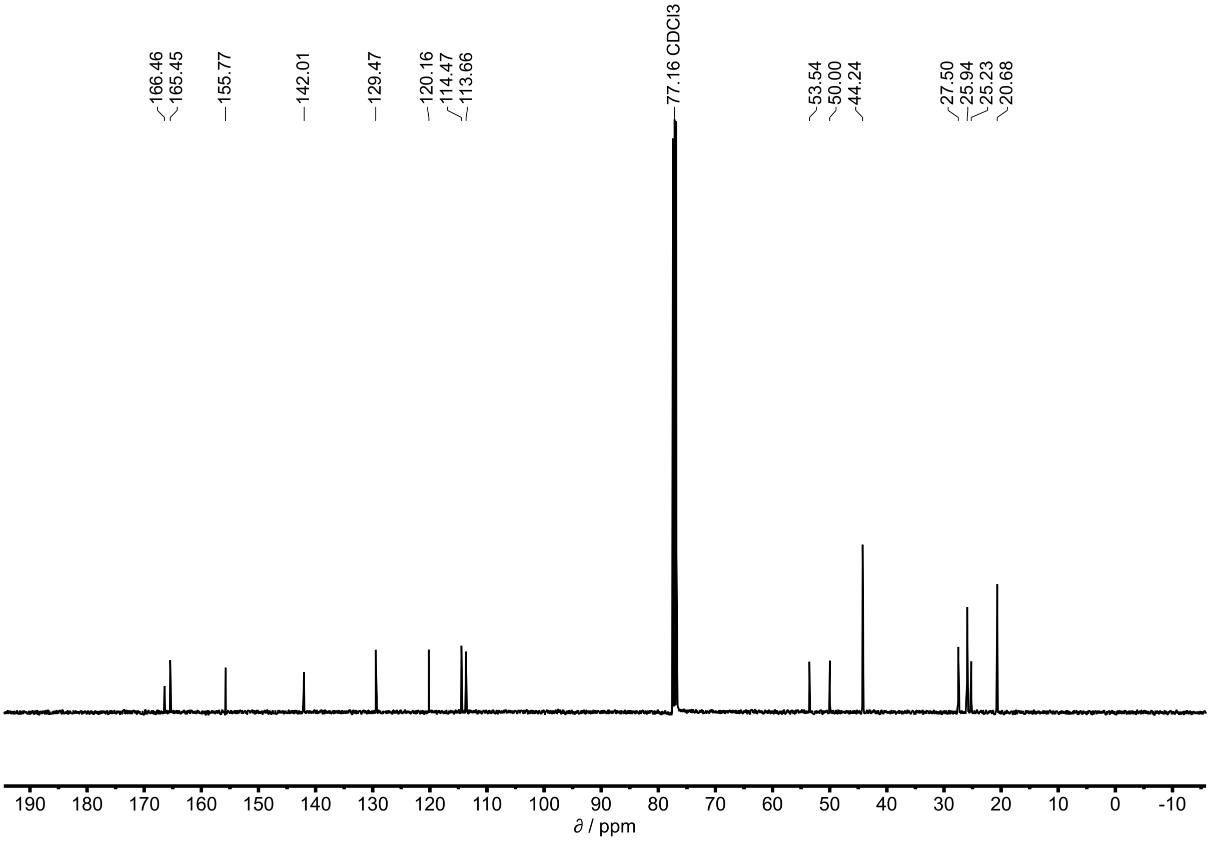

Figure S38. ^{13}C NMR spectrum (101 MHz, $CDCl_3$) of compound **D**.

Synthesis of **A**

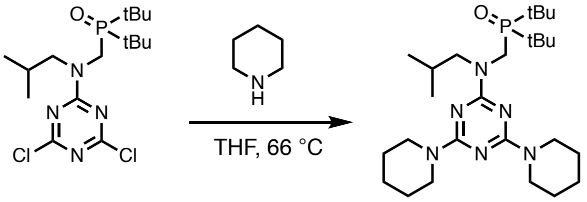

A solution of **9** (100 mg, 0.253 mmol, 1.0 eq) and piperidine (100 µL, 86.2 mg, 1.01 mmol, 4.0 eq) in THF (10 mL) was heated under reflux for 15 h. The solvent was evaporated *in vacuo* and the residue was dissolved in EtOAc (30 mL). The solution was washed with a solution of citric acid (5% in water, 3 x 15 mL). The aqueous wash was extracted with EtOAc (15 mL). The combined organic phases were then washed with 1M NaOH solution (3 x 10 mL). The organic phase was dried ($MgSO_4$) and the solvent was evaporated *in vacuo*. The crude was purified by flash chromatography (SiO_2, 0-20% gradient of MeOH in DCM). The product was obtained as a colourless oil (120 mg, 0.244 mmol, 96% yield).

^{1}H NMR (400 MHz, CDCl$_3$): δ_H 4.39 (d, $^2J_{PH}$ = 2.8 Hz, 2H), 3.81 (d, J = 7.3 Hz, 2H), 3.69 (t, J = 5.3 Hz, 8H), 2.20 (non, J = 6.8 Hz, 1H), 1.61 (m, 4H), 1.52 (m, 8H), 1.26 (d, $^3J_{PH}$ = 12.9 Hz, 18H), 0.86 (d, J = 6.8 Hz, 6H);
^{13}C NMR (101 MHz, CDCl$_3$): δ_C 165.7, 165.6, 53.0, 44.2, 38.2 (d, $^1J_{PC}$ = 59.4 Hz), 35.8 (d, $^1J_{PC}$ = 56.7 Hz), 26.9, 26.6, 26.0 (br), 25.2, 20.7;
^{31}P NMR (162 MHz, CDCl$_3$): δ_P 58.63;
FT-IR (ATR): ν_{max} /cm^{-1} 2931, 2853,1526, 1479, 1459, 1430, 1388, 1370, 1346, 1298, 1271, 1245, 1232, 1207, 1149, 1131, 1093, 1021, 990, 933, 852, 834, 806, 749, 729, 662, 648, 582, 512, 488, 449;
HRMS (ES+): calcd for $C_{26}H_{49}N_6OP + 2H^+$ is 493.3778, found 493.3791 (+2.6ppm).

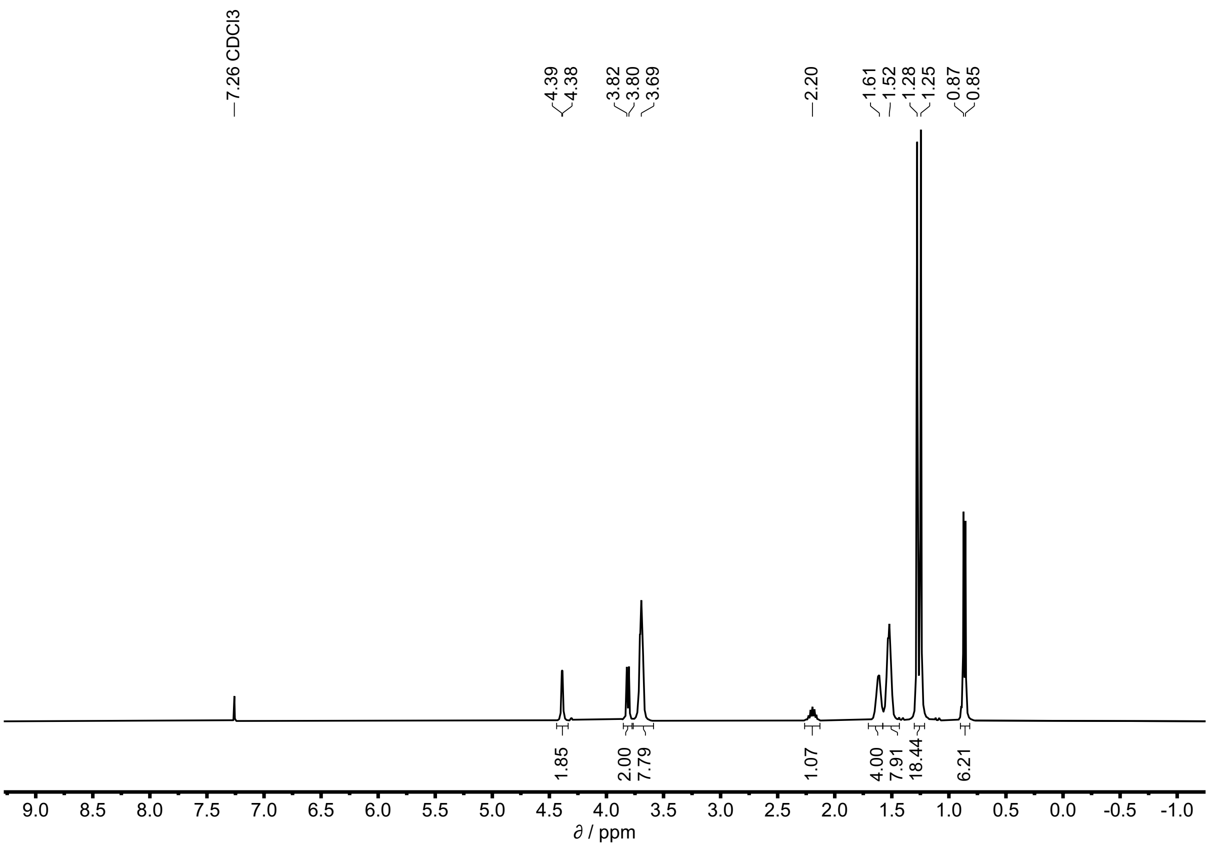

Figure S39. ^{1}H NMR spectrum (400 MHz, $CDCl_3$) of compound **A**.

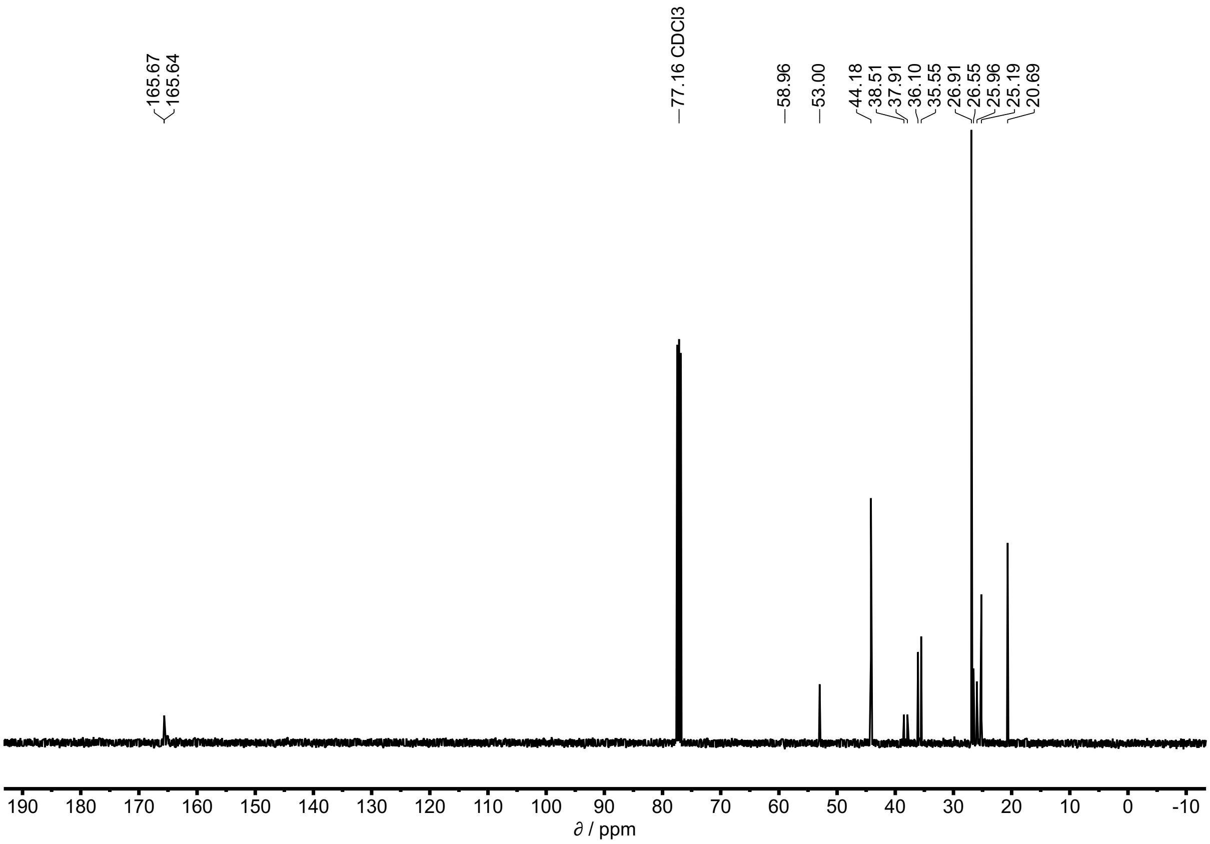

Figure S40. ^{13}C NMR spectrum (101 MHz, $CDCl_3$) of compound **A**.

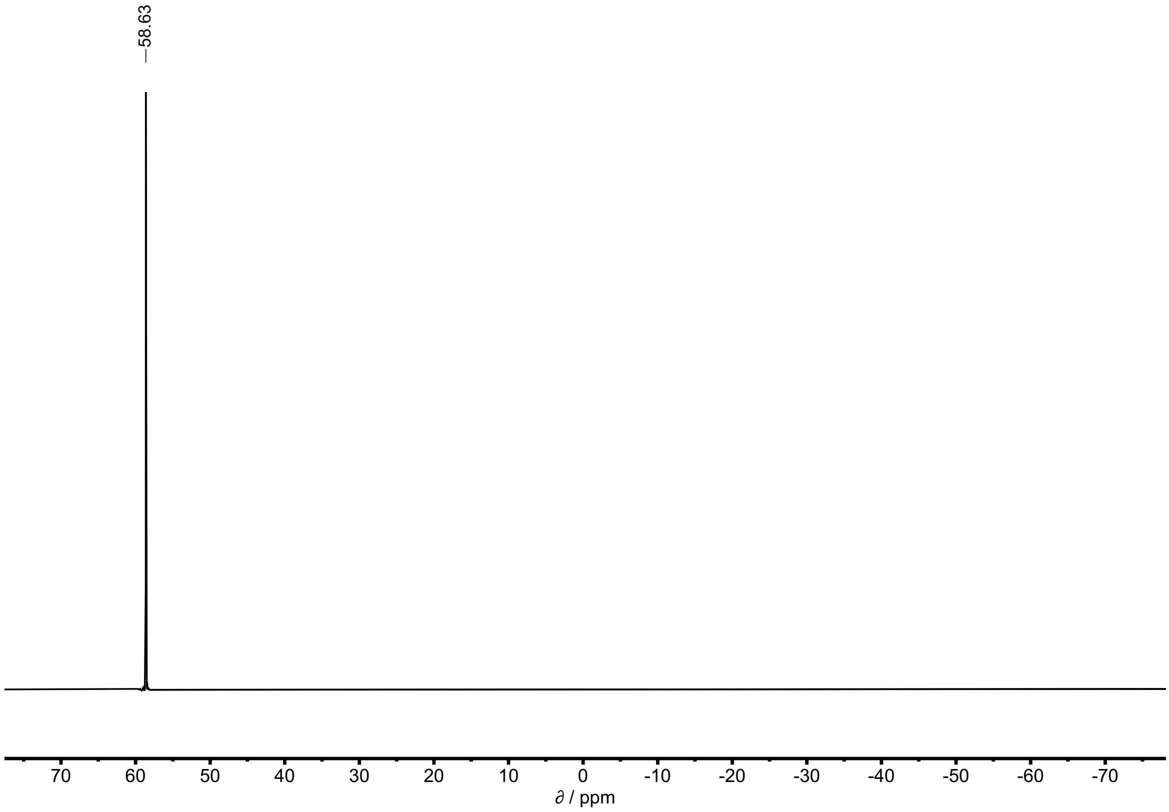

Figure S41. ^{31}P NMR spectrum (162 MHz, $CDCl_3$) of compound **A**.

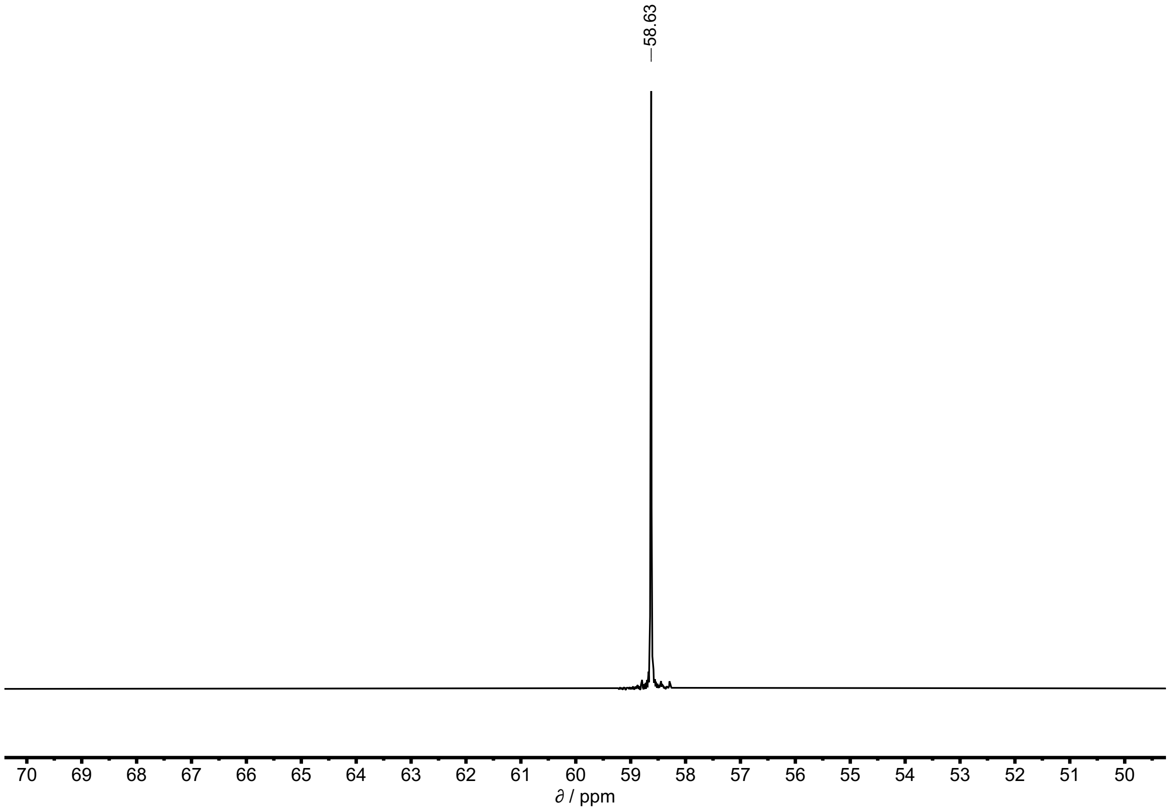

Figure S42. ^{31}P NMR spectrum (162 MHz, $CDCl_3$) of compound **A**.

Synthesis of 2-mers

Synthesis of **DD**

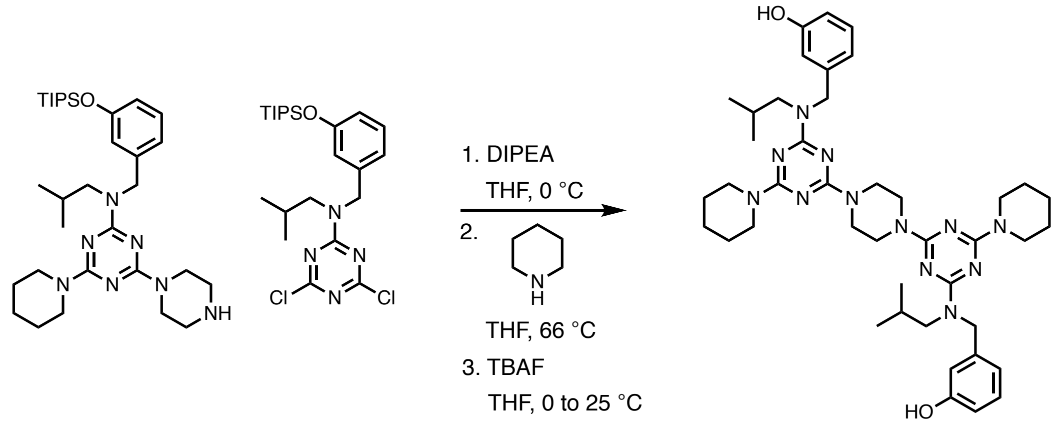

To a solution of **5** (200 mg, 0.414 mmol, 1.0 eq) and **8** (241 mg, 0.414 mmol, 1.0 eq) in THF (20 mL) was added DIPEA (143 μL, 106 mg, 0.82 mmol, 2.0 eq), and the reaction mixture was stirred for 15 h at 22 °C. The solvent was removed *in vacuo* and the residue was dissolved in EtOAc (40 mL). The solution was washed with a solution of citric acid (5% in water, 3 x 15 mL). The aqueous wash was extracted with EtOAc (15 mL). The combined organic phases were dried ($MgSO_4$) and the solvent was evaporated *in vacuo* to yield a transparent oil.

To a solution of the thus obtained oil in THF (20 mL) was added piperidine (162 μL, 140 mg, 1.64 mmol, 4.0 eq) and the solution was heated under reflux for 15 h. The solvent was removed *in vacuo* and the residue was dissolved in EtOAc (40 mL). The solution was washed with a solution of citric acid (5% in water, 3 x 15 mL). The aqueous wash was extracted with EtOAc (15 mL). The combined organic phases were dried ($MgSO_4$) and the solvent was evaporated *in vacuo* to yield a transparent oil.

To a solution of the thus obtained oil in THF (15 mL) at 0 °C was added dropwise a solution of TBAF (1M in THF, 0.90 mL, 0.90 mmol, 2.2 eq), and the reaction mixture was stirred at 22 °C until complete conversion of the starting material, as monitored by LCMS. The reaction mixture was diluted with EtOAc (30 mL) and washed with sat. aqueous ammonium chloride solution (2 x 10 mL). The organic phase was dried ($MgSO_4$) and evaporated *in vacuo*. The crude was purified by flash chromatography (SiO_2, 0-5% gradient of MeOH in DCM). The product was afforded as a white solid (254 mg, 0.332 mmol, 80% yield over 3 steps).

^{1}H NMR (400 MHz, $CDCl_3$): δ_H 7.12 (t, *J* = 8.0 Hz, 2H), 6.80 (d, *J* = 8.2 Hz, 2H), 6.68 (s, 2H), 6.63 (d, *J* = 8.5 Hz, 2H), 5.22 (br s, 2H), 4.79 (m, 4H), 3.72 (m, 16H), 3.32 (d, *J* = 7.2 Hz, 4H), 2.08 (m, 2H), 1.55 (m, 12H), 0.88 (d, *J* = 6.6 Hz, 12H);
^{13}C NMR (101 MHz, $CDCl_3$): δ_C 166.4, 165.7, 165.2, 155.8, 141.7, 129.5, 120.0, 114.4, 113.7, 53.6, 50.0, 44.3, 43.3, 27.5, 25.9, 25.2, 20.7;
FT-IR (ATR): ν_{max} /cm^{-1} 2931, 2853, 1590, 1524, 1483, 1434, 1386, 1368, 1298, 1265, 1238, 1205, 1182, 1134, 997, 806, 754, 735;
HRMS (ES+): calcd for $C_{42}H_{60}N_{12}O_2 + H^+$ is 765.5040, found 765.5024 (-2.1ppm).

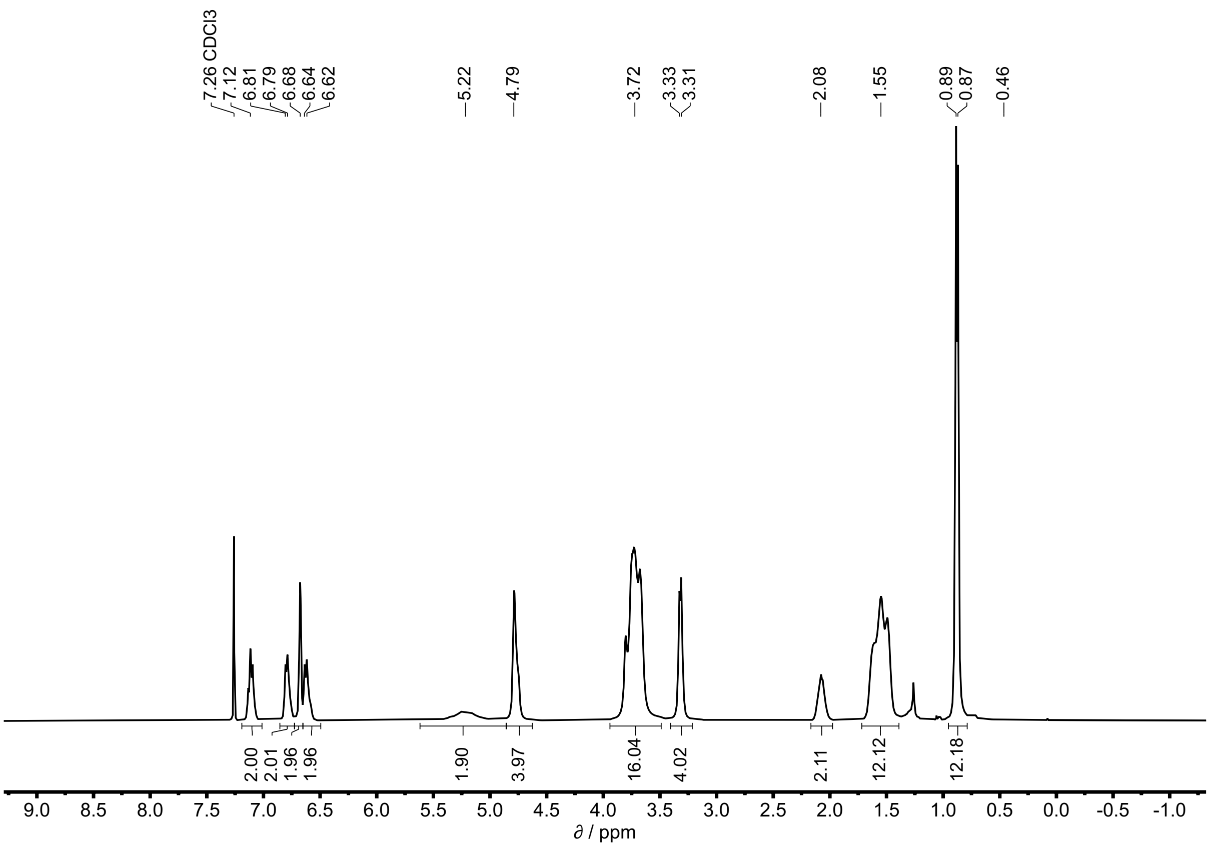

Figure S43. ^{1}H NMR spectrum (400 MHz, $CDCl_3$) of compound **DD**.

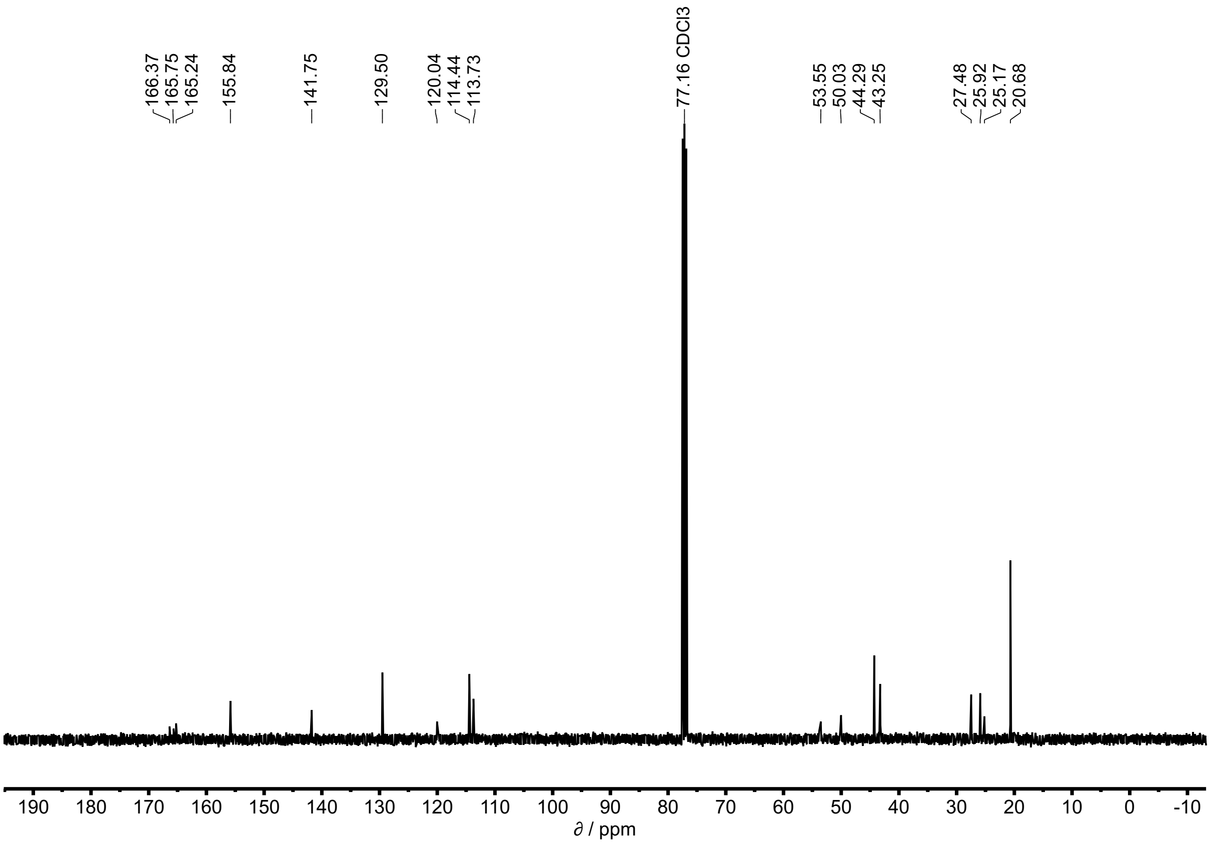

Figure S44. ^{13}C NMR spectrum (101 MHz, $CDCl_3$) of compound **DD**.

Synthesis of **AA**

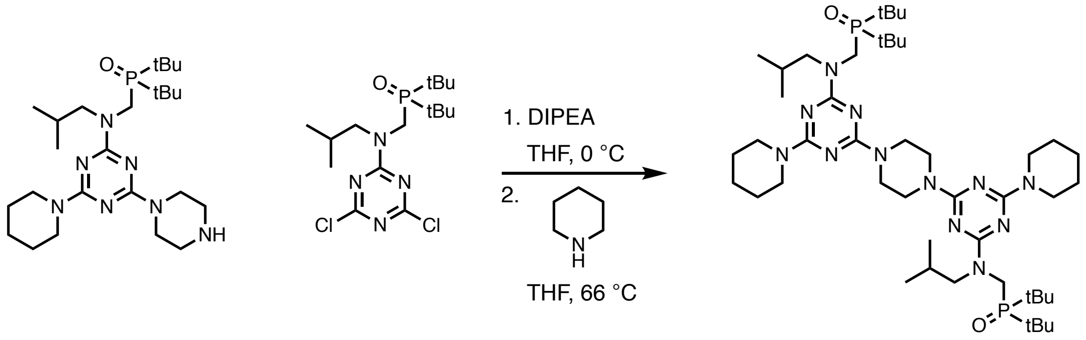

To a solution of **9** (100 mg, 0.253 mmol, 1.0 eq) and **13** (125 mg, 0.253 mmol, 1.0 eq) in THF (20 mL) was added DIPEA (88 µL, 65 mg, 0.51 mmol, 2.0 eq), and the reaction mixture was stirred for 15 h at 22 °C. The solvent was removed *in vacuo* and the residue was dissolved in EtOAc (40 mL). The solution was washed with a solution of citric acid (5% in water, 3 x 15 mL). The aqueous wash was extracted with EtOAc (15 mL). The combined organic phases were dried ($MgSO_4$) and the solvent was evaporated *in vacuo* to yield a white powder.

To a solution of the thus obtained white powder in THF (20 mL) was added piperidine (100 µL, 86 mg, 1.0 mmol, 4.0 eq) and the solution was heated under reflux for 15 h. The solvent was removed *in vacuo* and the residue was dissolved in EtOAc (40 mL). The solution was washed with a solution of citric acid (5% in water, 3 x 15 mL). The aqueous wash was extracted with EtOAc (15 mL). The combined organic phases were then washed with 1M NaOH solution (3 x 10 mL). The organic phase was dried ($MgSO_4$) and the solvent was evaporated *in vacuo* to yield a transparent oil. The crude was purified by flash chromatography (SiO_2, 0-20% gradient of MeOH in DCM). The product was afforded as a colourless oil (200 mg, 0.222 mmol, 88% yield over 2 steps).

^{1}H NMR (400 MHz, $CDCl_3$): δ_H 4.39 (br s, 4H, rotamers), 3.84 (m, 4H), 3.80-3.67 (m, 16H), 2.21 (non, J = 6.8 Hz, 2H), 1.68-1.49 (m, 12H, overlaps with HDO), 1.27 (d, $^3J_{PH}$ = 12.9 Hz, 36H), 0.88 (d, J = 6.7 Hz, 12H);

^{13}C NMR (101 MHz, $CDCl_3$): δ_C 165.6 (3 carbon atoms), 53.1, 44.2, 43.2, 38.2 (d, $^1J_{PC}$ = 60.8 Hz), 35.9 (d, $^1J_{PC}$ = 56.7 Hz), 26.9, 26.6, 26.0, 25.2, 20.7;

^{31}P NMR (162 MHz, $CDCl_3$): δ_P 58.76, 58.72, 58.58;

FT-IR (ATR): ν_{max} /cm^{-1} 2931, 2853, 1524, 1477, 1429, 1367, 1297, 1265, 1206, 1144, 1096, 1023, 996, 834, 806, 749, 662, 509, 450;

HRMS (ES+): calcd for $C_{46}H_{86}N_{12}O_2P_2 + 2H^+$ is 451.3313, found 451.3305 (-0.9ppm).

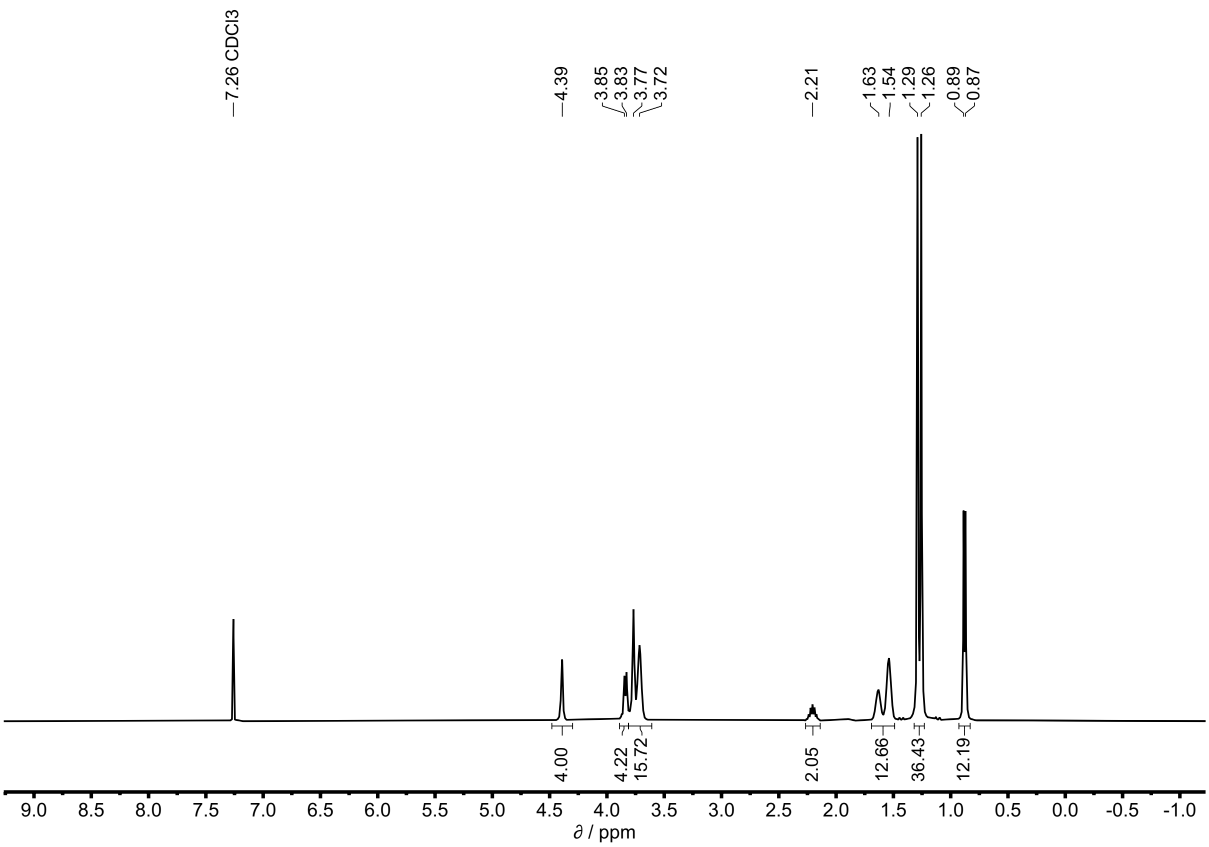

Figure S45. ^{1}H NMR spectrum (400 MHz, $CDCl_3$) of compound **AA**.

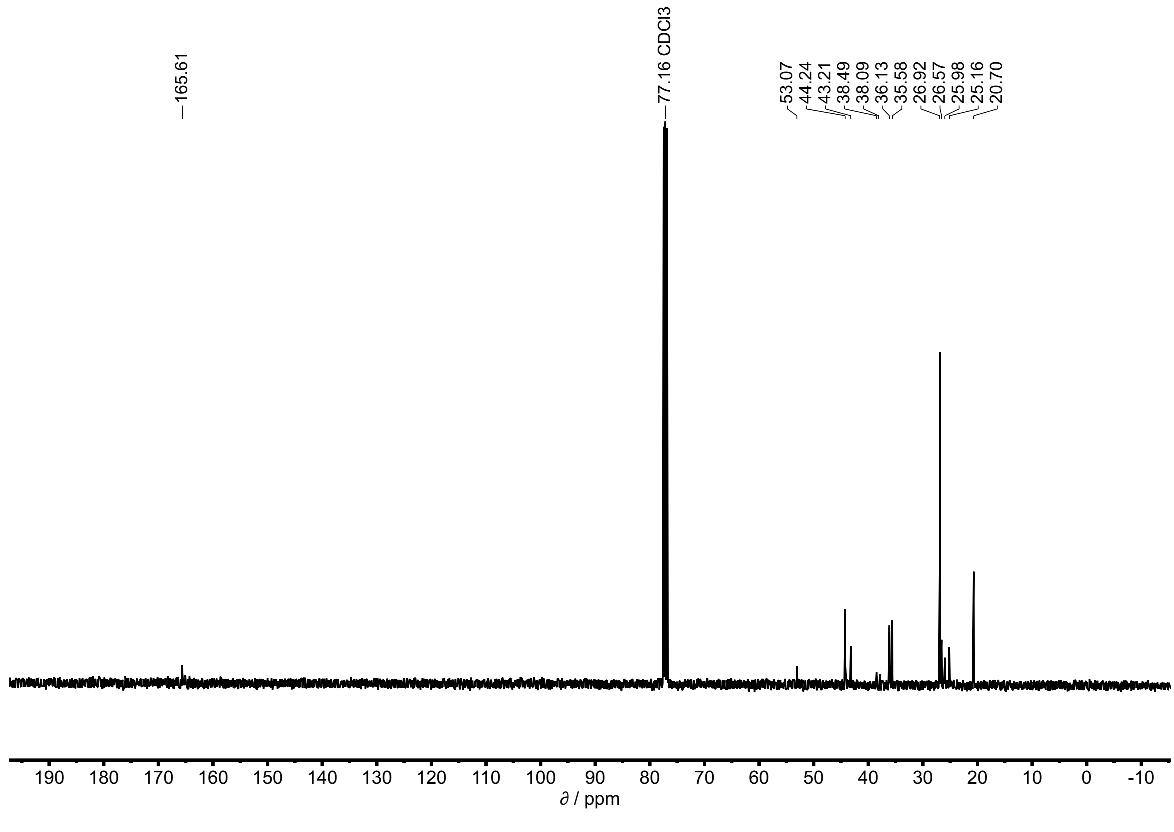

Figure S46. ^{13}C NMR spectrum (101 MHz, $CDCl_3$) of compound **AA**.

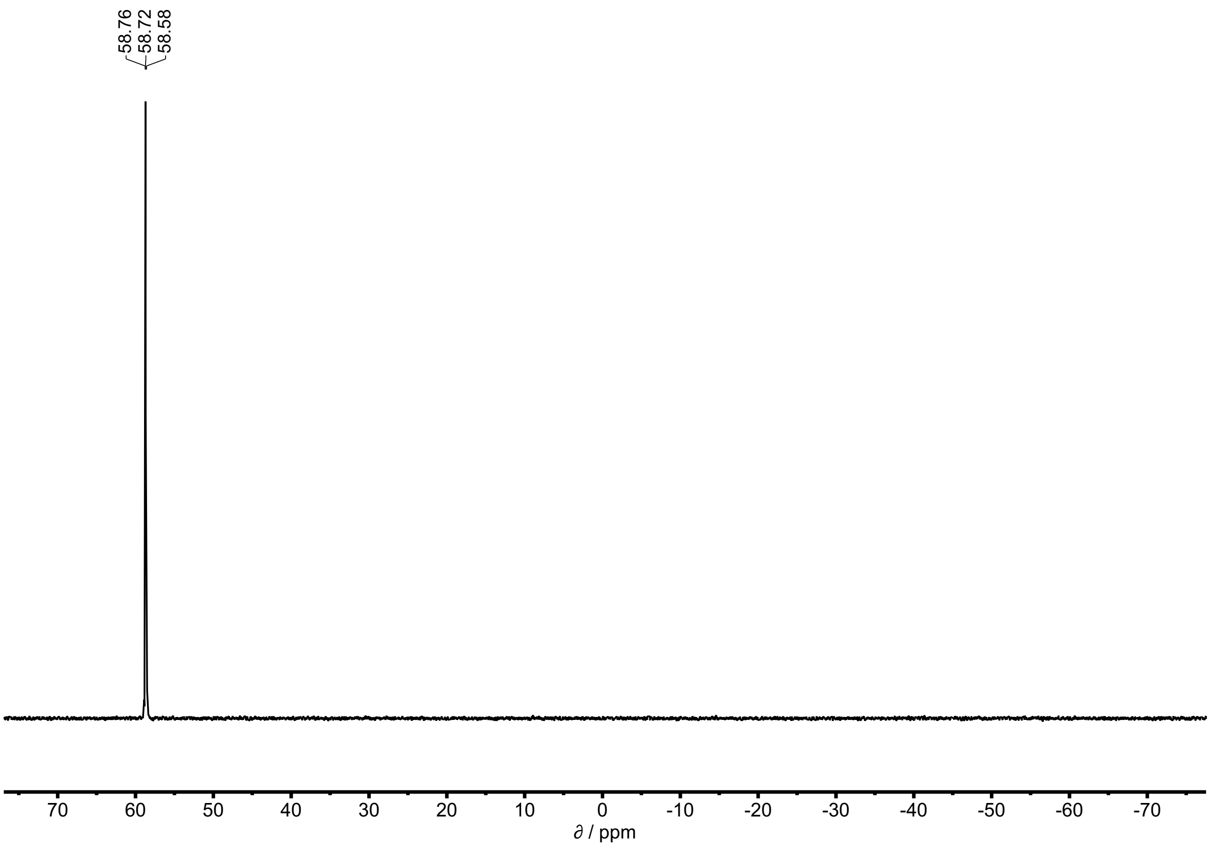

Figure S47. ^{31}P NMR spectrum (162 MHz, $CDCl_3$) of compound **AA**.

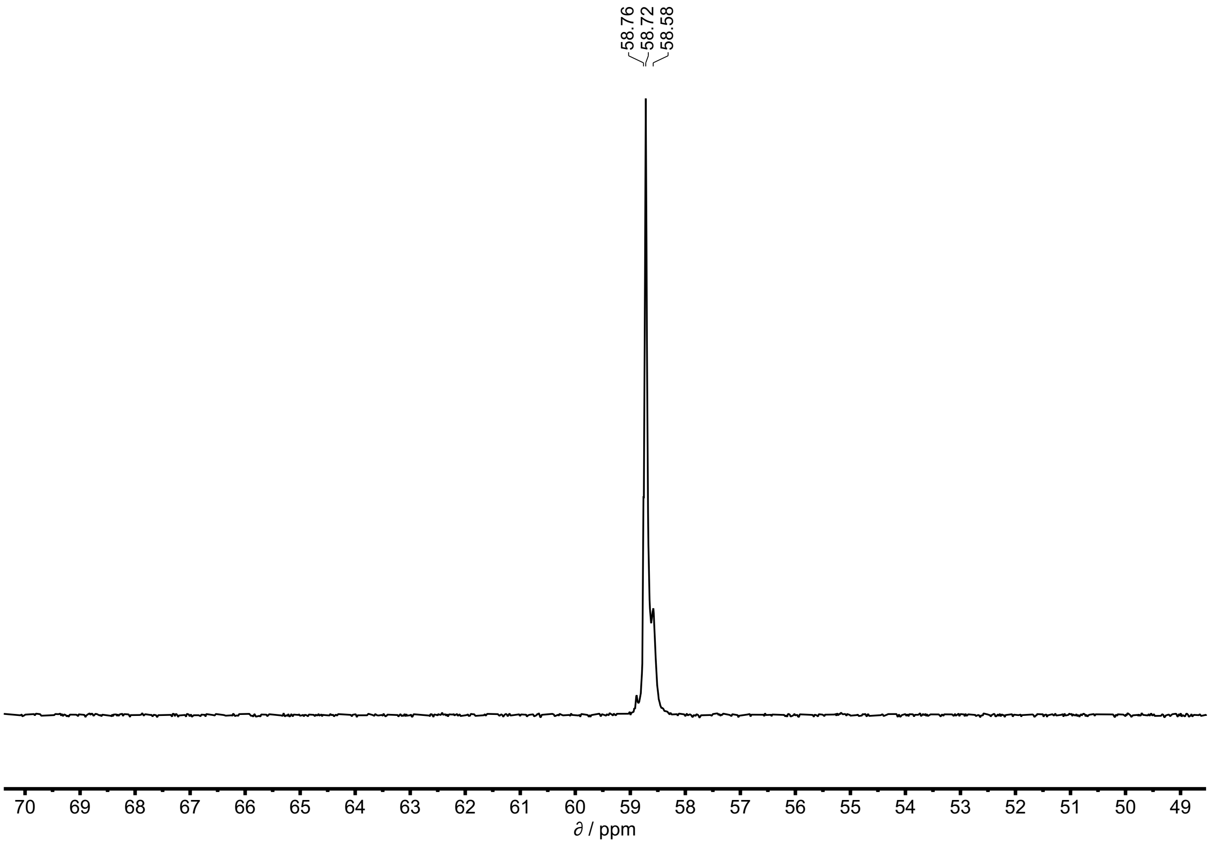

Figure S48. ^{31}P NMR spectrum (162 MHz, $CDCl_3$) of compound **AA**.

Synthesis of **AD**

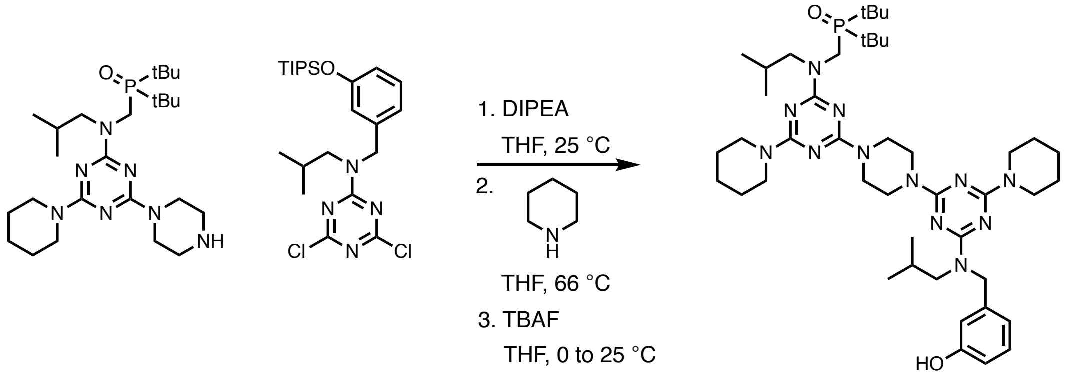

To a solution of **5** (40 mg, 0.083 mmol, 1.0 eq) and **13** (40 mg, 0.081 mmol, 1.0 eq) in THF (10 mL) was added DIPEA (42 µL, 31 mg, 0.24 mmol, 3.0 eq) and the reaction mixture was stirred at 22 °C for 15h. The solvent was removed *in vacuo* and the residue was dissolved in EtOAc (30 mL). The solution was washed with a solution of citric acid (5% in water, 3 x 10 mL). The aqueous wash was extracted with EtOAc (10 mL). The combined organic phases were dried ($MgSO_4$) and the solvent was evaporated *in vacuo*.
To a solution of the thus obtained oil in THF (10 mL) was added piperidine (33 µL, 28 mg, 0.33 mmol, 4.0 eq) and the solution was heated under reflux for 15 h. The solvent was removed *in vacuo* and the residue was dissolved in EtOAc (30 mL). The solution was washed with a solution of citric acid (5% in water, 3 x 10 mL). The aqueous wash was extracted with EtOAc (10 mL). The combined organic phases were dried ($MgSO_4$) and the solvent was evaporated *in vacuo* to yield an oil.
To a solution of the thus obtained oil in THF (15 mL) at 0 °C was added dropwise a solution of TBAF (1M in THF, 90 µL, 0.090 mmol, 1.1 eq), and the reaction mixture was stirred at 22 °C for 15 h. The reaction mixture was diluted with EtOAc (30 mL) and washed with sat. aqueous ammonium chloride solution (2 x 10 mL). The organic phase was dried ($MgSO_4$) and evaporated *in vacuo*. The crude was purified by flash chromatography (SiO_2, 0-100% gradient of EtOAc in 40-60 Pet. Ether). The product was afforded as a white solid (55 mg, 0.066 mmol, 82% yield over 3 steps).

^{1}H NMR (400 MHz, $CDCl_3$): δ_H ^{1}H NMR (400 MHz, $CDCl_3$) δ 8.09 (br s, 1H), 7.09 (t, *J* = 7.8 Hz, 1H), 6.77 (m, 3H), 4.79 (s, 2H), 4.44 (s, 2H), 3.72 (m, 18H), 3.30 (d, *J* = 7.2 Hz, 2H), 2.21 (m, 1H), 2.08 (m, 1H), 1.69 – 1.41 (m, 12H, overlaps with HDO), 1.29 (d, $^{3}J_{HP}$ = 13.0 Hz, 18H), 0.86 (m, 12H);
^{13}C NMR (101 MHz, $CDCl_3$): δ_C 166.4, 165.8 (br), 165.7 (br), 165.3, 157.3 (br), 141.3, 129.3, 119.1 and 118.9, 114.8, 114.0, 53.3, 53.1, 50.1, 44.2, 43.2, 38.3 (d, $^{1}J_{CP}$ = 85.9 Hz), 35.9 (d, $^{1}J_{CP}$ = 89.1 Hz), 27.4, 26.9, 26.6, 26.0 (br), 25.2, 25.2, 20.7, 20.7;
^{31}P NMR (162 MHz, $CDCl_3$): δ_P 60.58, 60.38;
FT-IR (ATR): ν_{max} /cm^{-1} 2933, 2852, 1529, 1481, 1433, 1368, 1266, 1206, 1133, 1096, 1026, 997, 807, 753;
HRMS (ES+): calcd for $C_{44}H_{73}N_{12}O_2P + H^+$ is 833.5795, found 833.5760 (-4.2ppm).

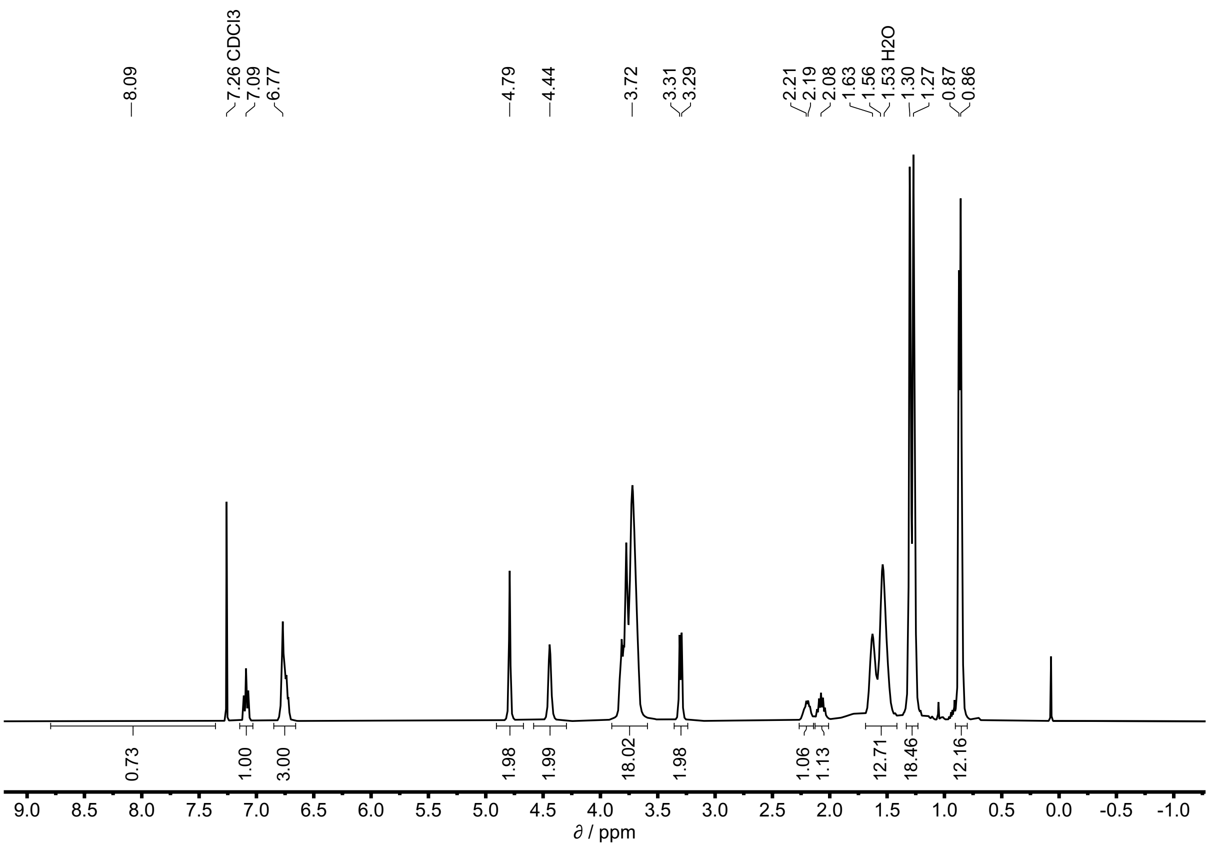

Figure S49. ^{1}H NMR spectrum (400 MHz, $CDCl_3$) of compound **AD.**

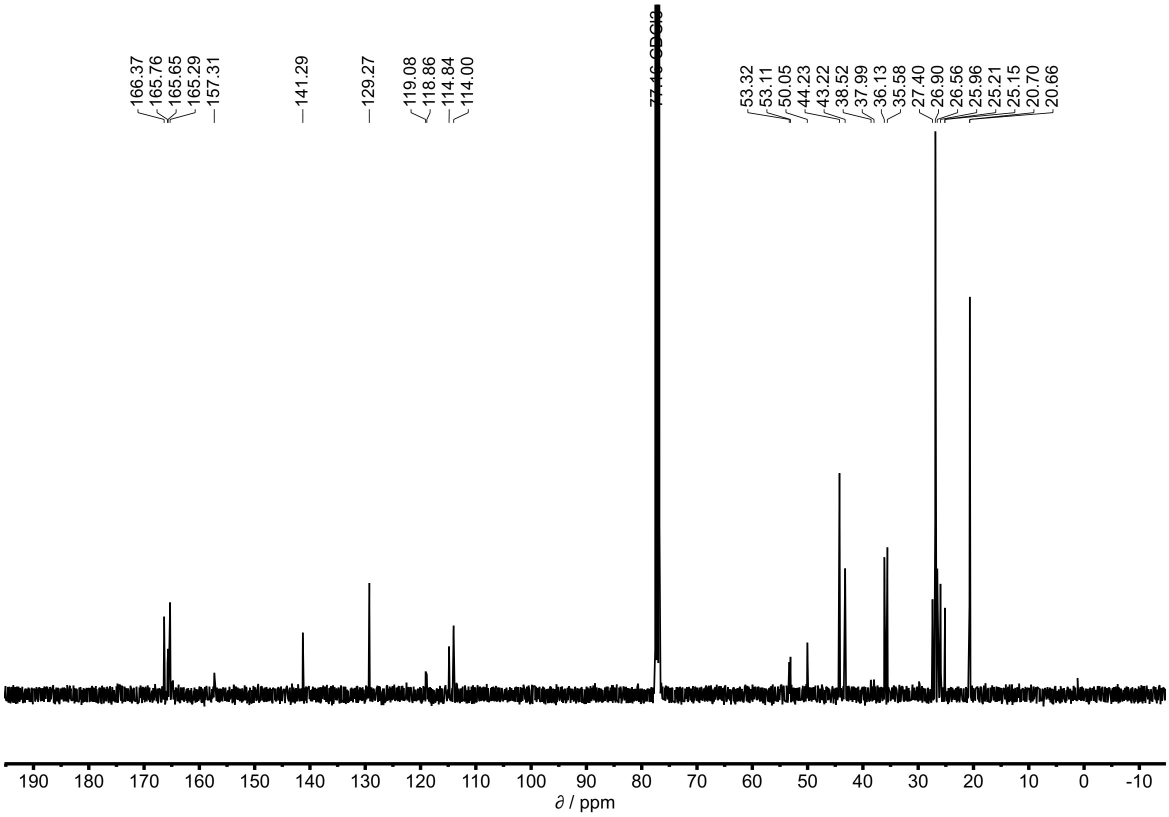

Figure S50. ^{13}C NMR spectrum (101 MHz, $CDCl_3$) of compound **AD**.

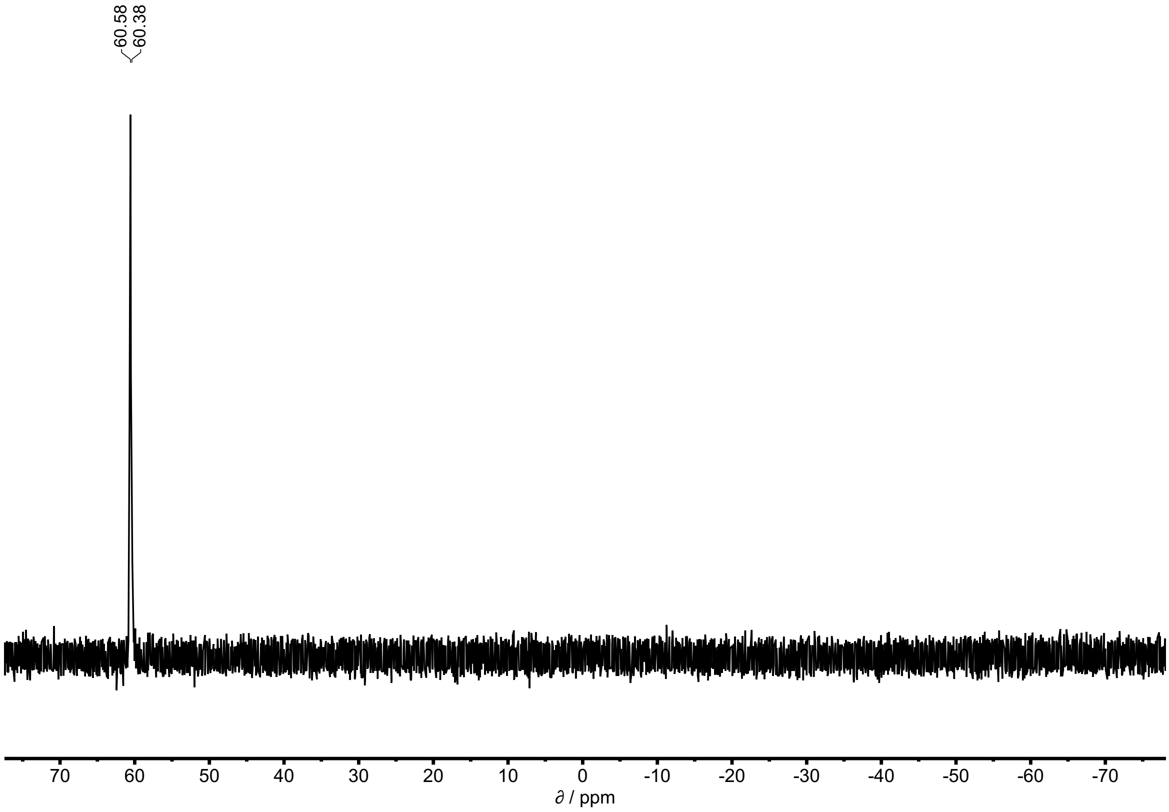

Figure S51. ^{31}P NMR spectrum (162 MHz, $CDCl_3$) of compound **AD**.

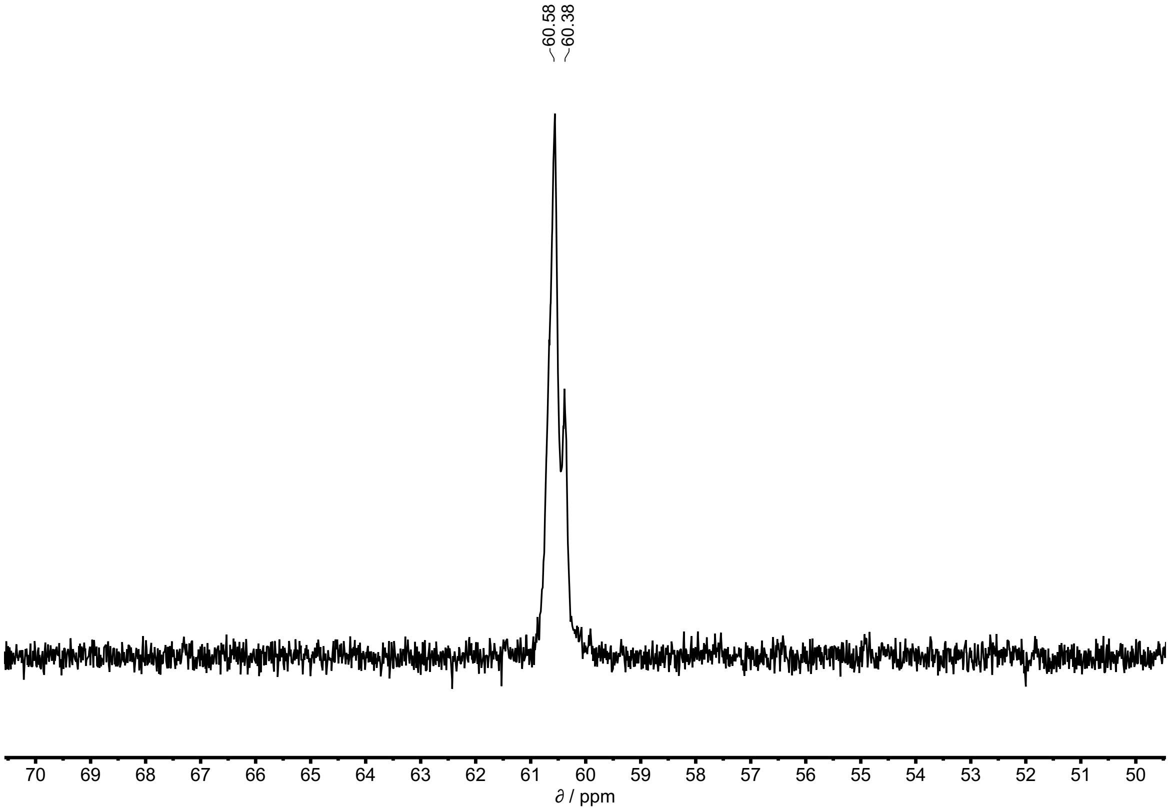

Figure S52. ^{31}P NMR spectrum (162 MHz, $CDCl_3$) of compound **AD**.

Synthesis of 3-mers

Due to the presence of multiple slowly interconverting rotamers, the peaks in the ^{1}H NMR spectrum are often very broad and show unresolved coupling. The number of signals in the ^{13}C NMR spectrum of the trimers are not always what would be expected based on symmetry and free rotation around single bonds. This is due to pseudo-symmetry (reduces the number of signals) and the presence of slowly interconverting rotamers (increases the number of signals). Peaks that are related to one another either by pseudosymmetry or by slow rotation around C—N bonds are separated by 'and'.

Synthesis of **DDD**

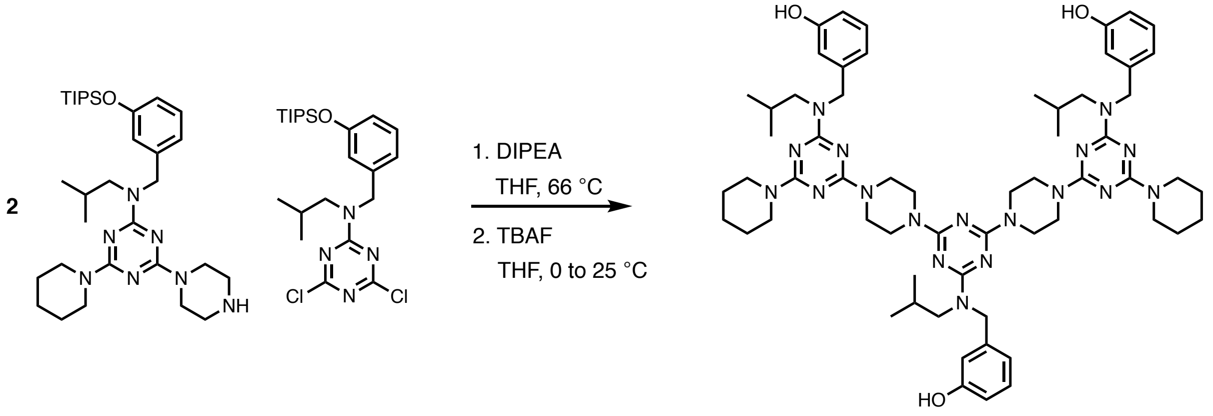

To a solution of **8** (200 mg, 0.344 mmol, 4.0 eq) and **5** (42.5 mg, 0.0859 mmol, 1.0 eq) in THF (10 mL) was added DIPEA (120 µL, 89 mg, 0.689 mmol, 8.0 eq) and the reaction mixture was heated under reflux for 63 h. The solvent was removed *in vacuo* and the residue was dissolved in EtOAc (40 mL). The solution was washed with a solution of citric acid (5% in water, 3 x 15 mL). The aqueous wash was extracted with EtOAc (15 mL). The combined organic phases were dried ($MgSO_4$) and the solvent was evaporated *in vacuo*. The crude was purified by flash chromatography (SiO_2, 0-25% gradient of EtOAc in 40-60 Pet. Ether) to yield the TIPS-protected trimer (81 mg).

The thus obtained TIPS-protected trimer (81 mg, 0.052 mmol assuming 100% purity, 1.0 eq) was dissolved in THF (7 mL) and TBAF (1M in THF, 0.31 mL, 0.31 mmol, 6 eq) was added to the solution. The reaction mixture was stirred at 22 °C for 15h. The reaction mixture was diluted with EtOAc (20 mL) and washed with sat. aqueous ammonium chloride solution (2 x 10 mL). The organic phase was dried ($MgSO_4$) and evaporated *in vacuo*. The crude was purified by flash chromatography (SiO_2, 0-60% gradient of EtOAc in 40-60 Pet. Ether) to yield the product as a white solid (47 mg, 0.043 mmol, 49% yield over 2 steps).

^{1}H NMR (500 MHz, $CDCl_3$): δ_H 7.10 (m, 3H), 6.78 (m, 3H), 6.67 (s, 3H), 6.59 (m, 3H), 4.78 (m, 6H), 3.74 (m, 24H), 3.32 (d, *J* = 6.2 Hz, 6H), 2.08 (m, 3H), 1.55 (m, 12H, overlaps with HDO), 0.88 (m, 18H);

^{13}C NMR (126 MHz, $CDCl_3$): δ_C 166.4, 166.3 (br) and 166.2, 165.7 and 165.6, 165.5 (br), 165.2 and 165.2, 156.0 (br), 141.6 and 141.5, 129.5, 119.9, and 119.8 and 119.6, 114.5, 113.7 (br), 53.5, 50.0, 44.3, 43.2, 27.5, 25.9 and 25.9, 25.2 and 25.1, 20.7;

FT-IR (ATR): ν_{max} /cm^{-1} 3349, 2929, 2853, 1590, 1519, 1479, 1426, 1386, 1367, 1297, 1256, 1239, 1204, 1180, 1153, 1098, 1027, 996, 955, 908, 853, 806, 781, 732, 696, 648;

HRMS (ES+): calcd for $C_{60}H_{84}N_{18}O_3 + H^+$ is 1105.7052, found 1105.7052 (0.0ppm).

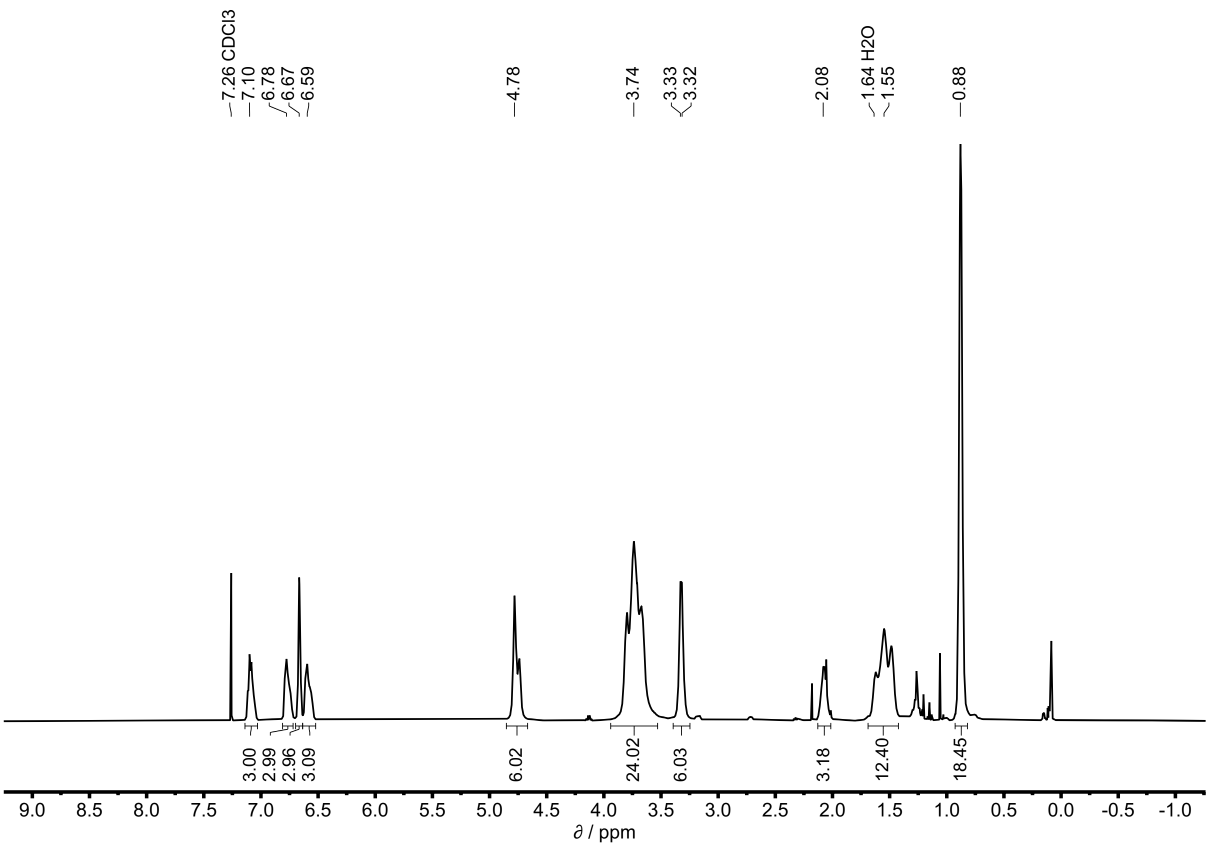

Figure S53. ^{1}H NMR spectrum (500 MHz, $CDCl_3$) of compound **DDD.**

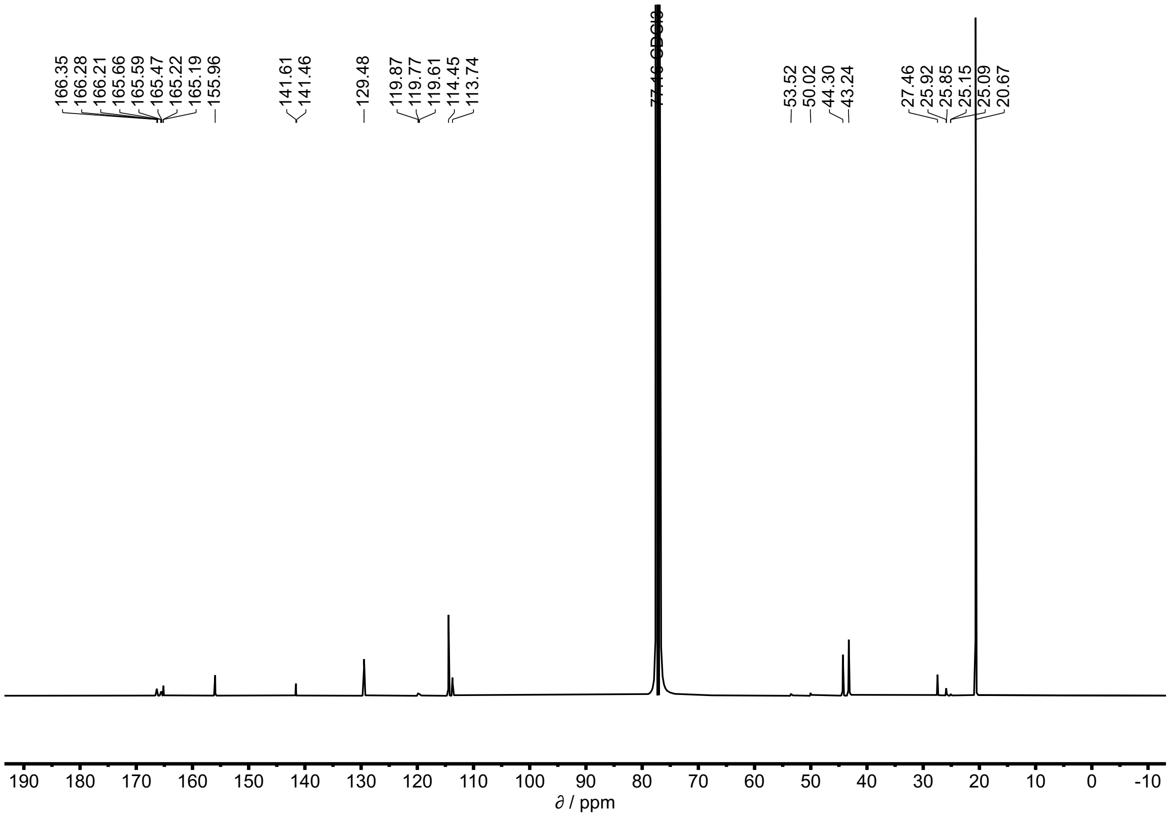

Figure S54. ^{13}C NMR spectrum (126 MHz, $CDCl_3$) of compound **DDD**.

Synthesis of **DAD**

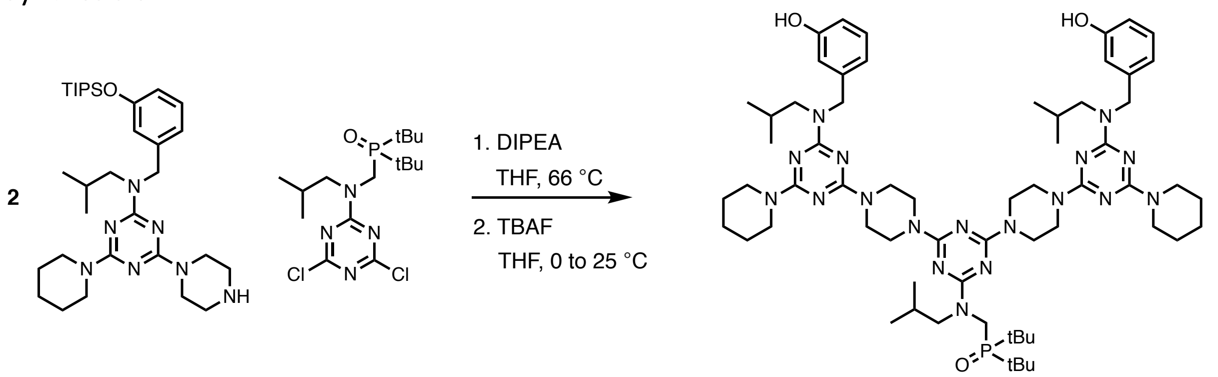

To a solution of **8** (229 mg, 0.394 mmol, 3.1 eq) and **9** (51 mg, 0.129 mmol, 1.0 eq) in THF (10 mL) was added DIPEA (132 µL, 97.9 mg, 0.759 mmol, 5.9 eq) and the reaction mixture was heated under reflux for 24 h. The solvent was removed *in vacuo* and the residue was dissolved in EtOAc (40 mL). The solution was washed with a solution of citric acid (5% in water, 3 x 15 mL). The aqueous wash was extracted with EtOAc (15 mL). The combined organic phases were dried ($MgSO_4$) and the solvent was evaporated *in vacuo*. The crude was purified by flash chromatography (SiO_2, 0-70% gradient of EtOAc in DCM) to yield the TIPS-protected trimer. The thus obtained product was dissolved in THF (10 mL) and TBAF (1M in THF, 0.379 mL, 0.379 mmol, 3.0 eq) was added to the solution. The reaction mixture was stirred at 22 °C for 40 h. The reaction mixture was diluted with EtOAc (30 mL) and washed with sat. aqueous ammonium chloride solution (2 x 10 mL). The organic phase was dried ($MgSO_4$) and evaporated *in vacuo*. The crude was purified by flash chromatography (SiO_2, 0-6% gradient of MeOH in DCM) to yield the product as a white solid (50 mg, 0.043 mmol, 34% yield over 2 steps).

^{1}H NMR (400 MHz, CDCl$_3$): δ_H ^{1}H NMR (400 MHz, CDCl$_3$) δ 7.52 (br s, 2H), 7.09 (t, *J* = 7.7 Hz, 2H), 6.74 (m, 6H), 4.78 (s, 4H), 4.46 (s, 2H), 3.72 (m, 26H), 3.31 (d, *J* = 7.2 Hz, 4H), 2.20 (m, 1H), 2.108 (m, 2H), 1.68 – 1.44 (m, 12H, overlaps with HDO), 1.29 (d, *J* = 12.1 Hz, 18H), 0.86 (m, 18H);
^{13}C NMR (101 MHz, CDCl$_3$): δ_C 166.3, 165.7, 165.6, 165.3, 156.9 (br), 141.4, 129.3, 119.3 and 119.1, 114.7, 114.0, 53.4, 53.2, 50.1, 44.3, 43.2, 38.3 (d, $^1J_{PC}$ = 59.6 Hz), 35.9 (d, $^1J_{PC}$ = 55.6 Hz), 27.4, 26.9, 26.6, 25.9 (br), 25.2 (br), 20.7, 20.6;
^{31}P NMR (162 MHz, CDCl$_3$): δ_P 61.41, 61.31;
FT-IR (ATR): ν_{max} /cm^{-1} 2953, 2931, 2852, 1527, 1480, 1432, 1386, 1367, 1298, 1258, 1241, 1205, 1133, 997, 807, 755;
HRMS (ES+): calcd for $C_{62}H_{97}N_{18}O_3P + H^+$ is 1173.7807, found 1173.7803 (-0.3ppm).

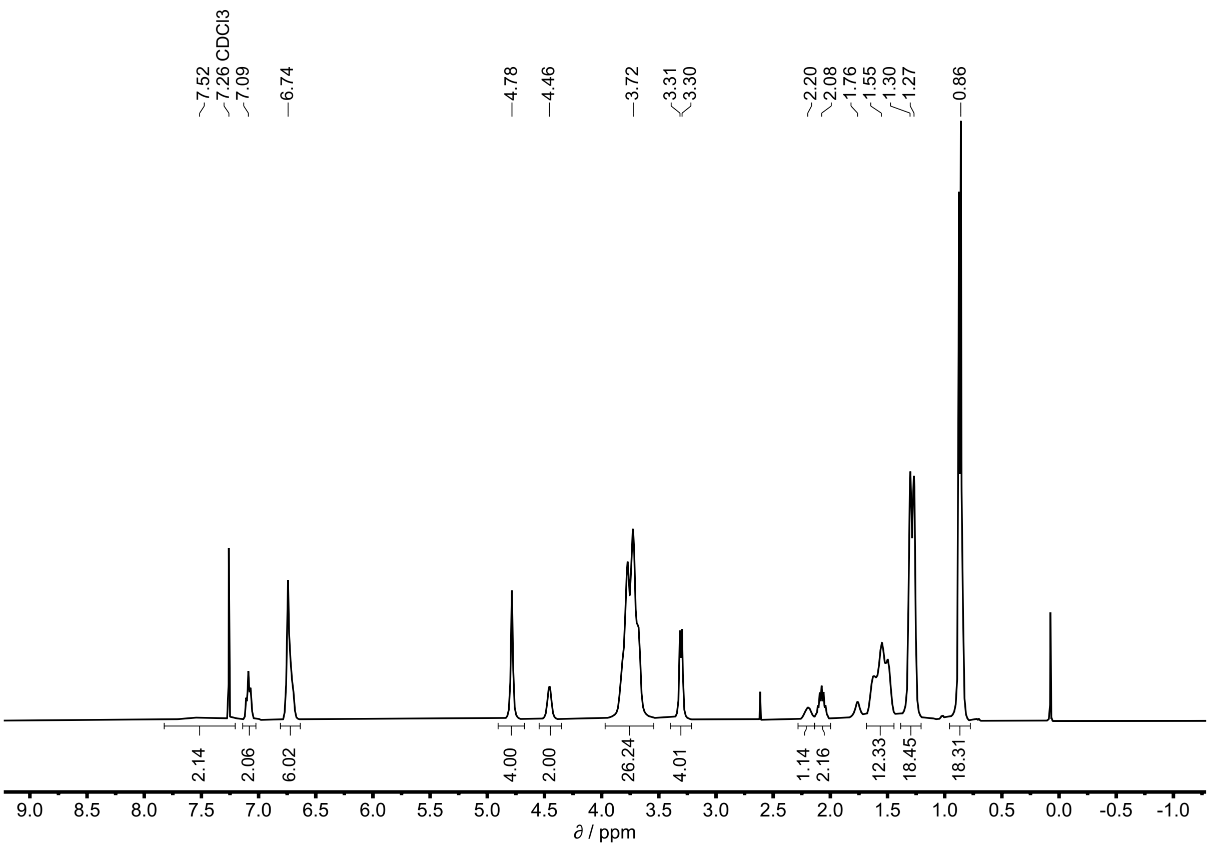

Figure S55. ^{1}H NMR spectrum (400 MHz, $CDCl_3$) of compound **DAD.**

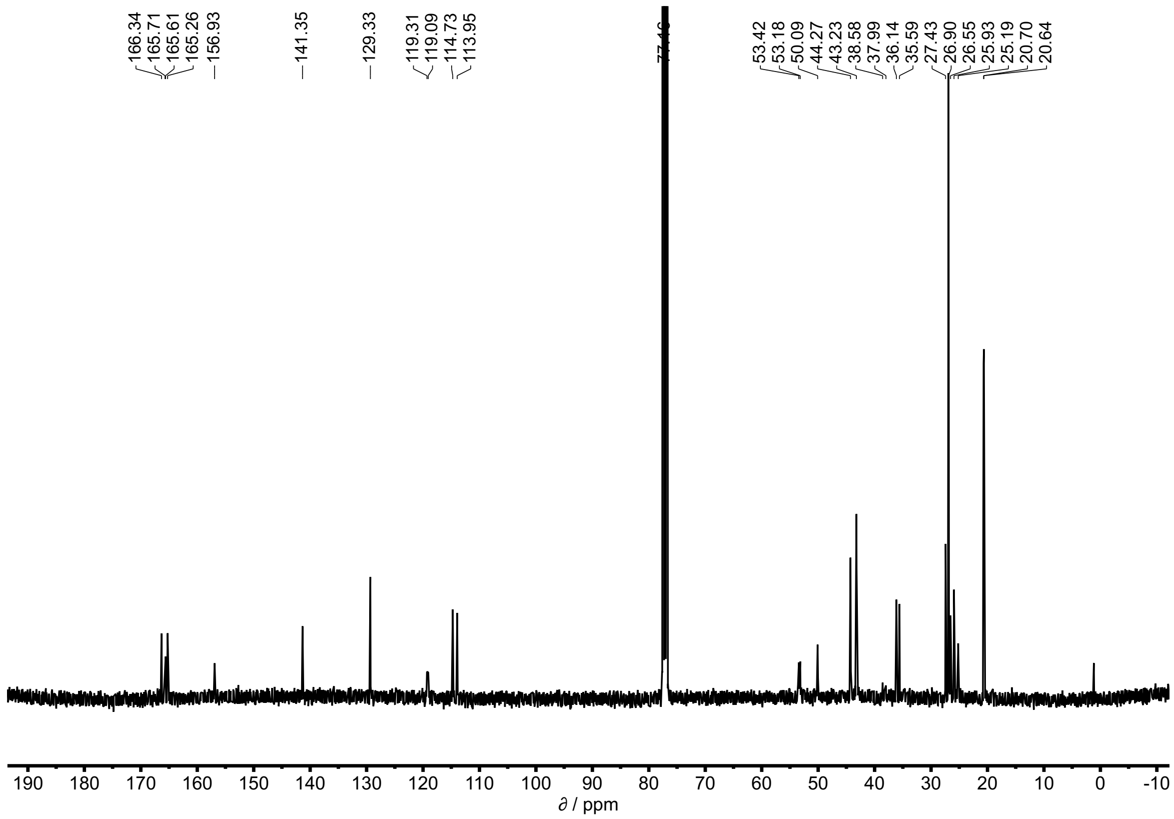

Figure S56. ^{13}C NMR spectrum (101 MHz, $CDCl_3$) of compound **DAD**.

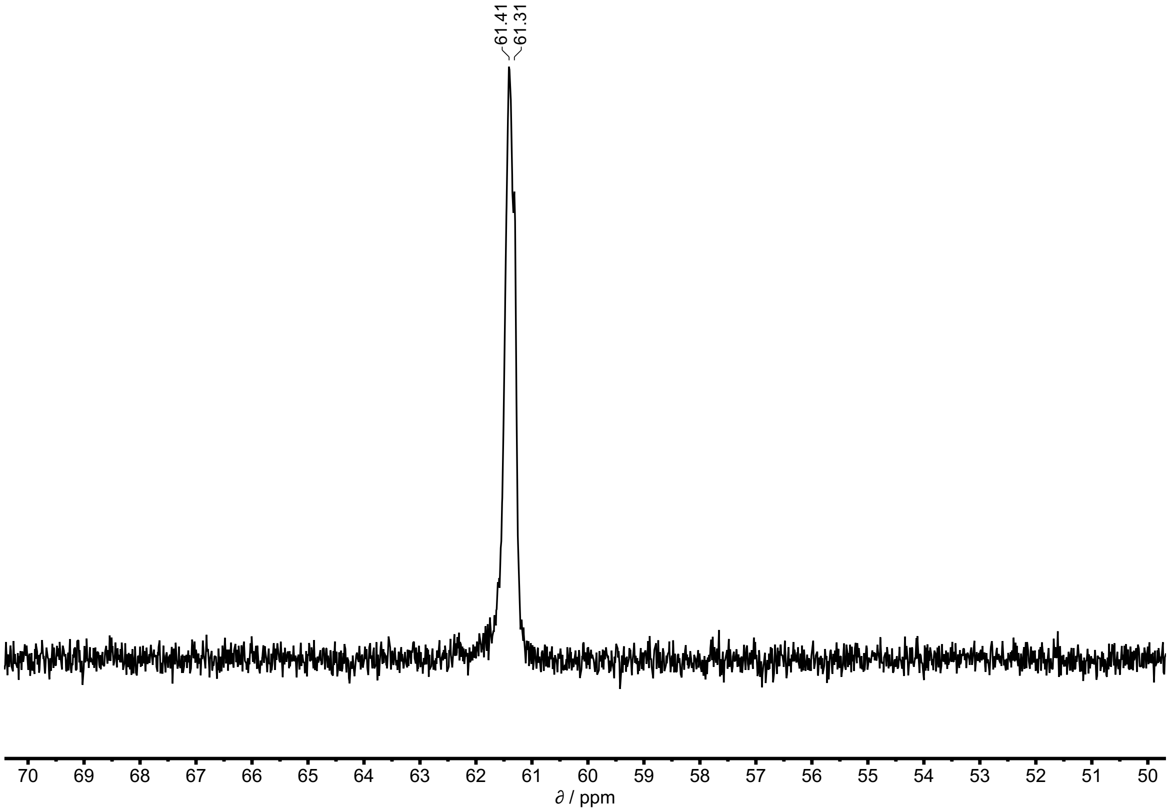

Figure S57. ^{31}P NMR spectrum (162 MHz, $CDCl_3$) of compound **DAD**.

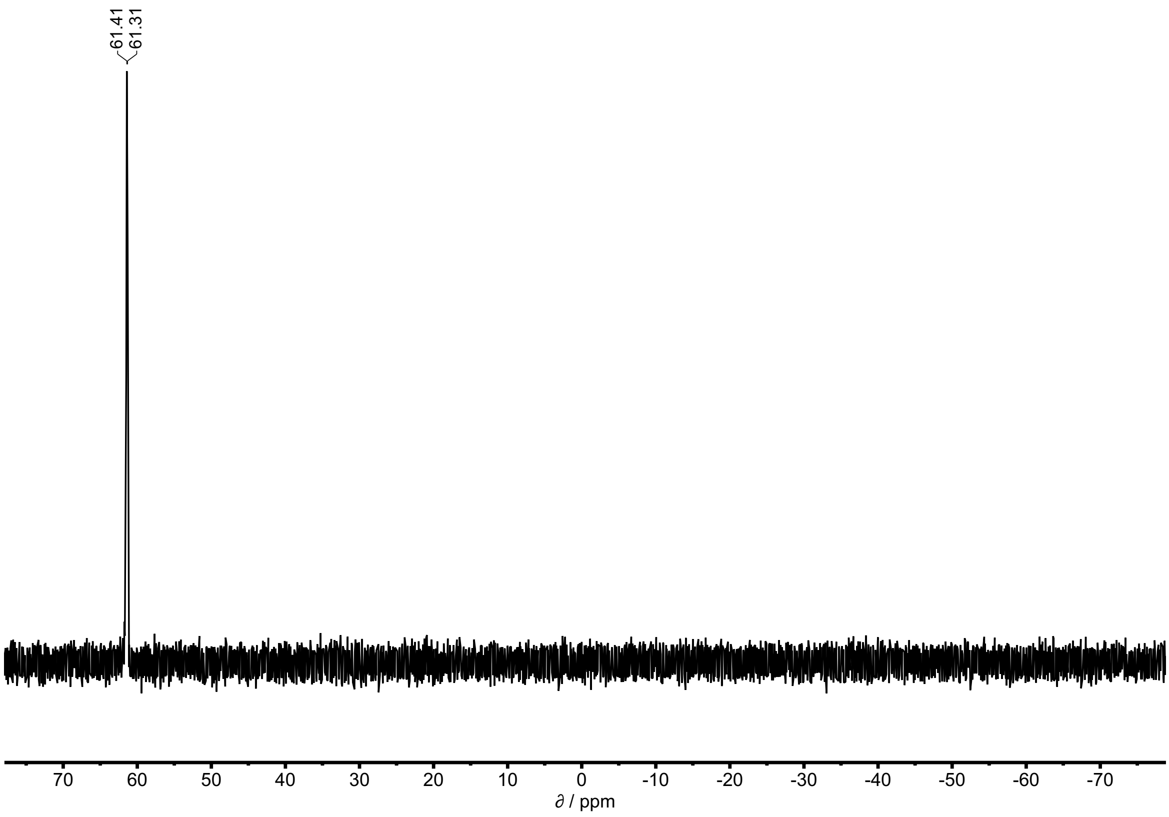

Figure S58. ^{31}P NMR spectrum (162 MHz, $CDCl_3$) of compound **DAD**.

Synthesis of **AAA**

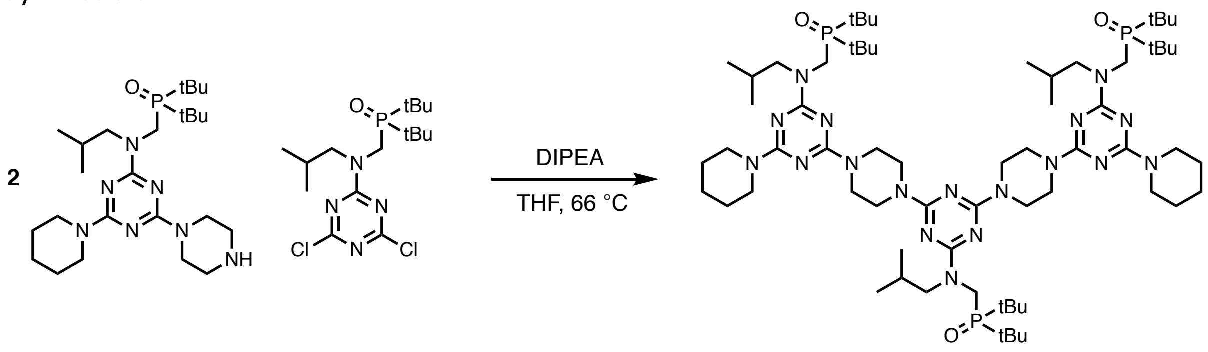

To a solution of **13** (162 mg, 0.328 mmol, 2.5 eq) and **9** (52 mg, 0.132 mmol, 1.0 eq) in THF (10 mL) was added DIPEA (100 µL, 74 mg, 0.574 mmol, 4.3 eq) and the reaction mixture was heated under reflux for 24 h. The solvent was removed *in vacuo* and the residue was dissolved in EtOAc (40 mL). The solution was washed with a solution of citric acid (5% in water, 3 x 15 mL). The aqueous wash was extracted with EtOAc (15 mL). The combined organic phases were then washed with 1M NaOH solution (3 x 10 mL). The organic phase was dried ($MgSO_4$) and the solvent was evaporated *in vacuo*. The crude was purified by flash chromatography (SiO_2, 0-13% gradient of MeOH in DCM). The product was afforded as a colourless oil (164 mg, 0.125 mmol, 95% yield).

^{1}H NMR (500 MHz, $CDCl_3$): δ_H 4.40 (m, 6H), 3.78 (m, 30H), 2.20 (m, 3H), 1.64 (m, 4H), 1.54 (m, 8H), 1.28 (m, 54H), 0.89 (m, 18H);
^{13}C NMR (126 MHz, $CDCl_3$): δ_C 165.6, 165.4, 165.2, 165.0, 164.8, 53.1 (br), 44.2, 43.2 and 43.2, 38.2 (d, $^1J_{PC}$ = 60 MHz), 35.9 and 35.9 (both are d, $^1J_{PC}$ = 55Hz), 26.9, 26.6, 25.9 (br), 25.1, 20.7;
^{31}P NMR (162 MHz, $CDCl_3$): δ_P 59.11, 59.04, 58.91;
FT-IR (ATR): ν_{max} /cm^{-1} 2953, 2931, 2868, 1524, 1476, 1428, 1388, 1366, 1348, 1296, 1255, 1205, 1175, 1143, 1094, 1023, 996, 932, 833, 805, 728, 643, 506, 450;
HRMS (ES+): calcd for $C_{66}H_{123}N_{18}O_3P_3 + H^+$ is 1309.9317, found 1309.9312 (-0.4 ppm).

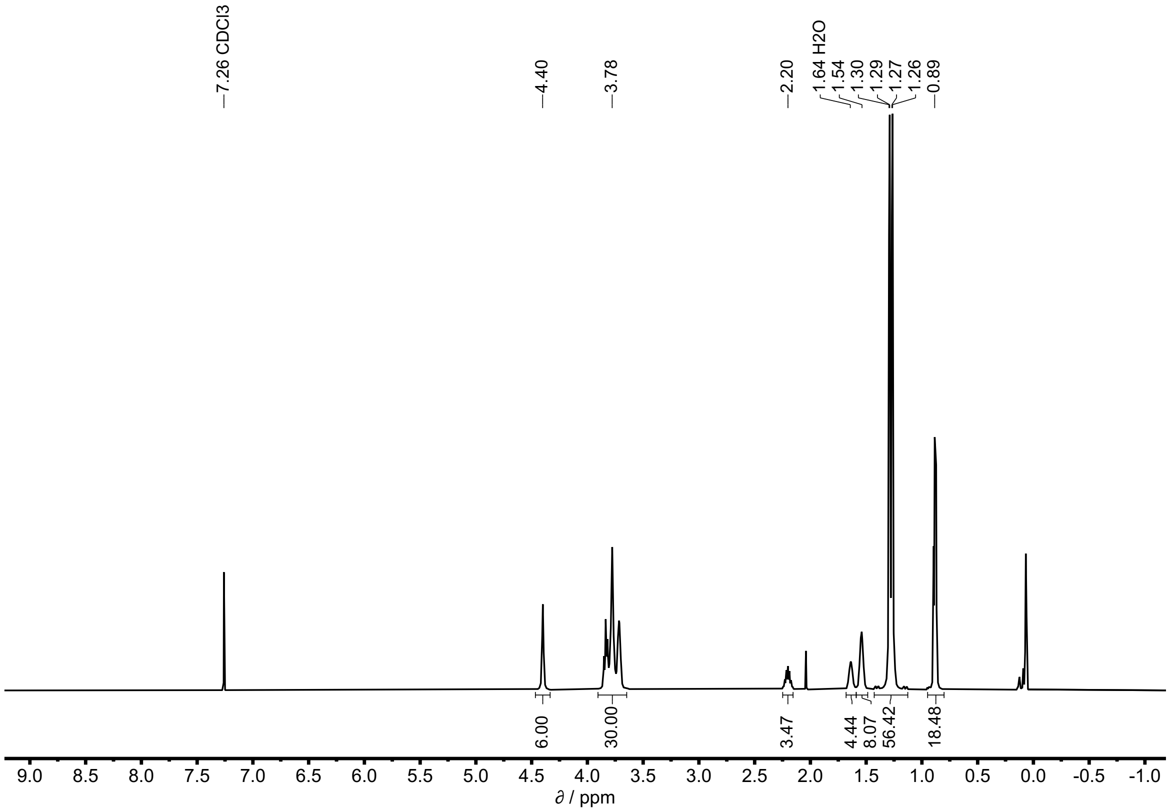

Figure S59. ^{1}H NMR spectrum (500 MHz, $CDCl_3$) of compound **AAA.**

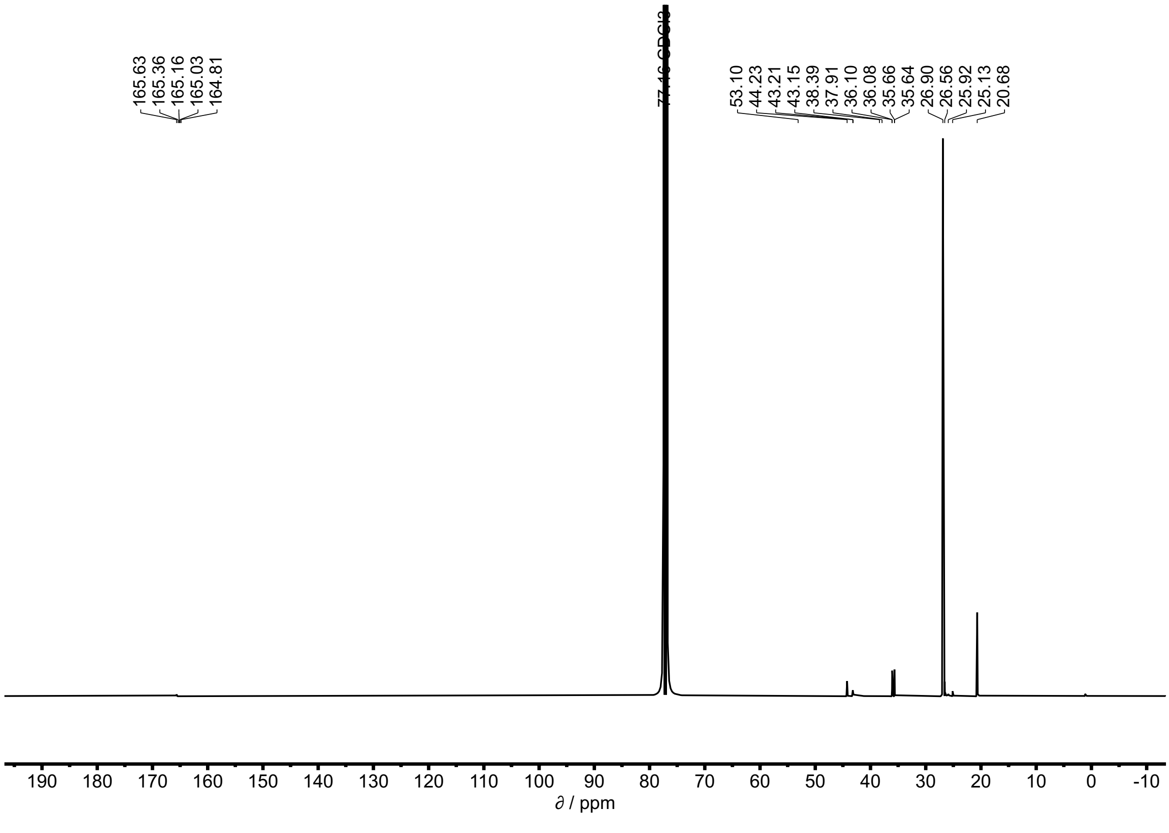

Figure S60. ^{13}C NMR spectrum (126 MHz, $CDCl_3$) of compound **AAA**.

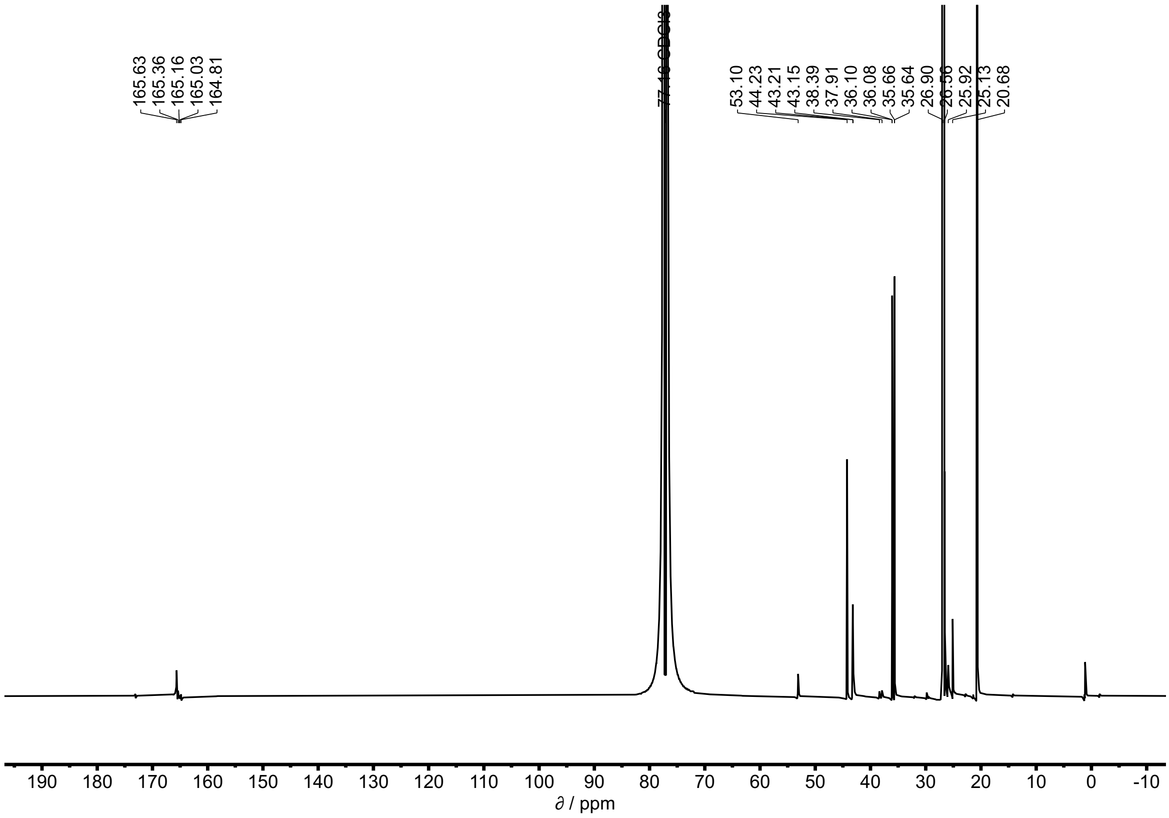

Figure S61. ^{13}C NMR spectrum (126 MHz, $CDCl_3$) of compound **AAA**.

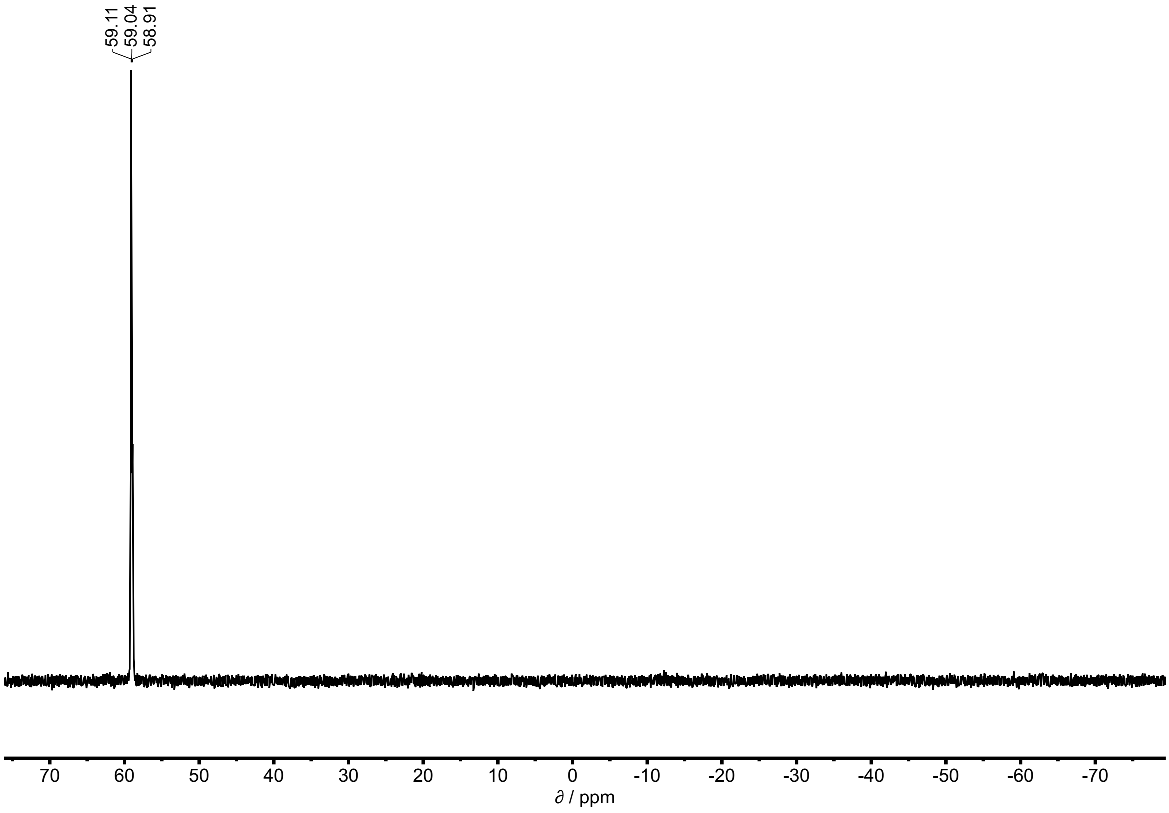

Figure S62. ^{31}P NMR spectrum (162 MHz, $CDCl_3$) of compound **AAA**.

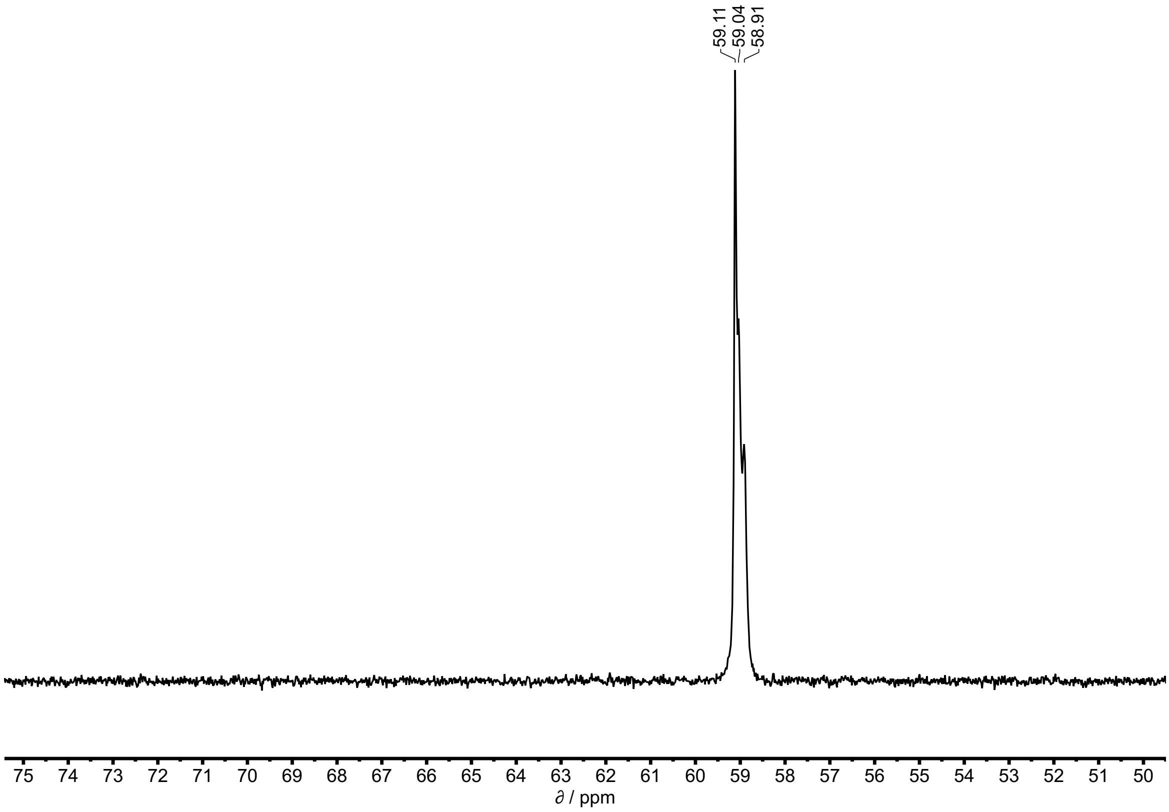

Figure S63. ^{31}P NMR spectrum (162 MHz, $CDCl_3$) of compound **AAA**.

Synthesis of **ADD and ADA**

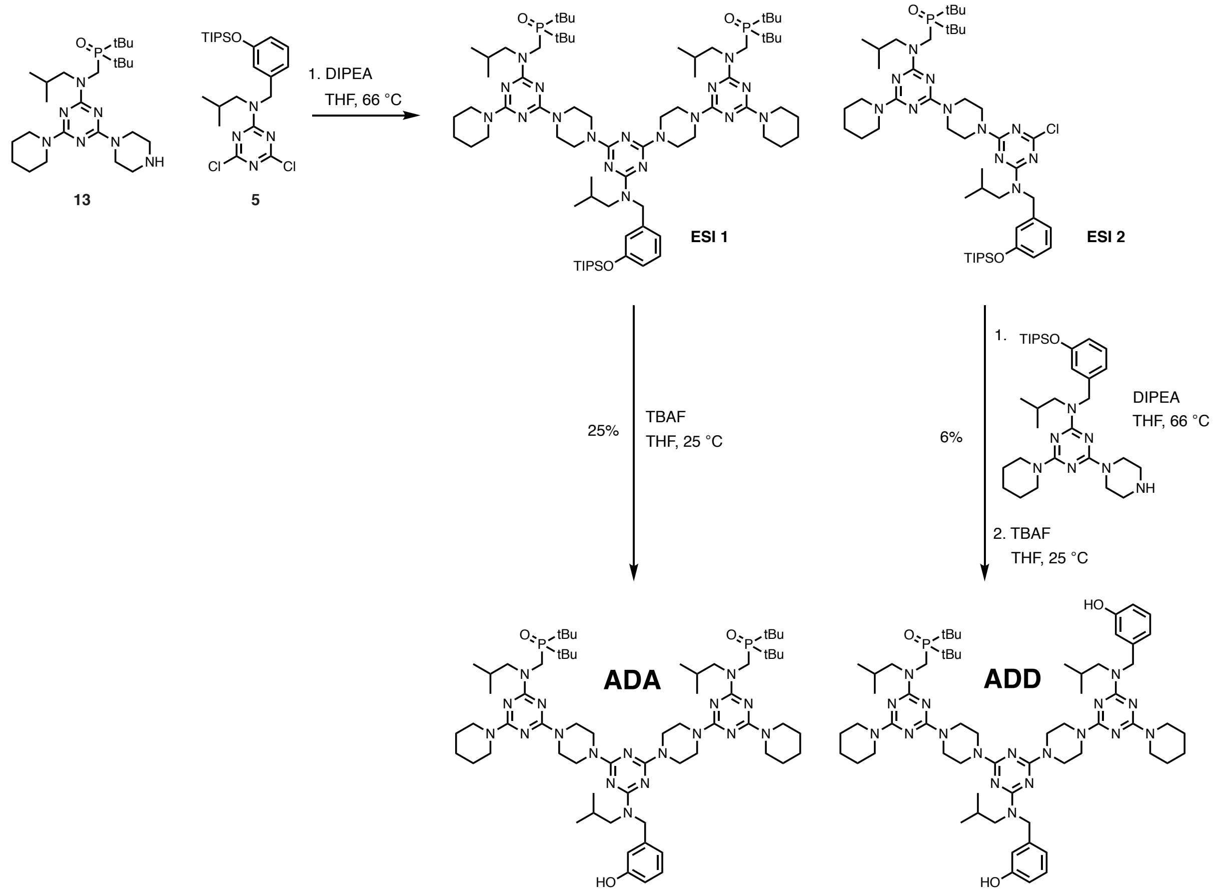

To a solution of **13** (146 mg, 0.296 mmol, 3.0 eq) and **5** (48 mg, 0.099 mmol, 1.0 eq) in THF (10 mL) was added DIPEA (140 µL, 104 mg, 0.816 mmol, 8.2 eq) and the reaction mixture was heated under reflux for 40 h. The solvent was removed *in vacuo* and the residue was dissolved in EtOAc (40 mL). The solution was washed with a solution of citric acid (5% in water, 3 x 15 mL). The aqueous wash was extracted with EtOAc (15 mL). The combined organic phases were dried ($MgSO_4$) and the solvent was evaporated *in vacuo*. The crude was purified by flash chromatography (SiO_2, 0-10% gradient of MeOH in DCM) to afford **ESI 1** (81 mg) and **ESI 2** (47 mg).

Synthesis of **ADA**

ESI 1 obtained above (81 mg, 0.058 mmol, 1.0 eq) was dissolved in THF (5 mL) and TBAF (1M in THF, 0.115 mL, 0.115 mmol, 2.0 eq) was added to the solution. The reaction mixture was stirred at 22 °C for 24h. The reaction mixture was diluted with EtOAc (20 mL) and washed with sat. aqueous ammonium chloride solution (2 x 10 mL). The organic phase was dried ($MgSO_4$) and evaporated *in vacuo*. The crude was purified by flash chromatography (SiO_2, 0-7% gradient of MeOH in DCM) to yield the product as a white solid (30.8 mg, 0.025 mmol, 25% yield over 2 steps).

^{1}H NMR (500 MHz, $CDCl_3$): δ_H 9.44 (br s, 1H), 7.08 (t, *J* = 7.5 Hz, 1H), 6.80 (m, 2H), 6.71 (d, *J* = 8.0 Hz, 1H), 4.81 (s, 2H), 4.43 (br s, 4H), 3.78 (m, 28H), 3.31 (d, *J* = 7.5 Hz, 2H), 2.21 (m, 2H), 2.08 (m, 1H), 1.64 (br m, 4H), 1.52 (br m, 8H), 1.28 (m, 36H), 0.86 (m, 18H);
^{13}C NMR (126 MHz, $CDCl_3$): δ_C 166.3, 165.6 (br), 165.5, 165.3, 165.0, 164.8, 157.9, 140.8, 129.2, 118.4, 115.0, 114.2, 53.1 (br), 50.0, 44.2, 43.2, 38.3 (d, $^1J_{CP}$ = 59 Hz), 35.9 (d, $^1J_{CP}$ = 55 Hz), 27.3, 26.9, 26.6, 25.9 (br), 25.1, 20.7, 20.7;
^{31}P NMR (162 MHz, $CDCl_3$): δ_P 60.12, 59.92, 59.70;
FT-IR (ATR): ν_{max} /cm^{-1} 2950, 2927, 2856, 1530, 1481, 1432, 1387, 1367, 1298, 1258, 1205, 1178, 1137, 1094, 997, 807;
HRMS (ES+): calcd for $C_{64}H_{110}N_{18}O_3P_2 + H^+$ is 1241.8562, found 1241.8546 (-1.3ppm).

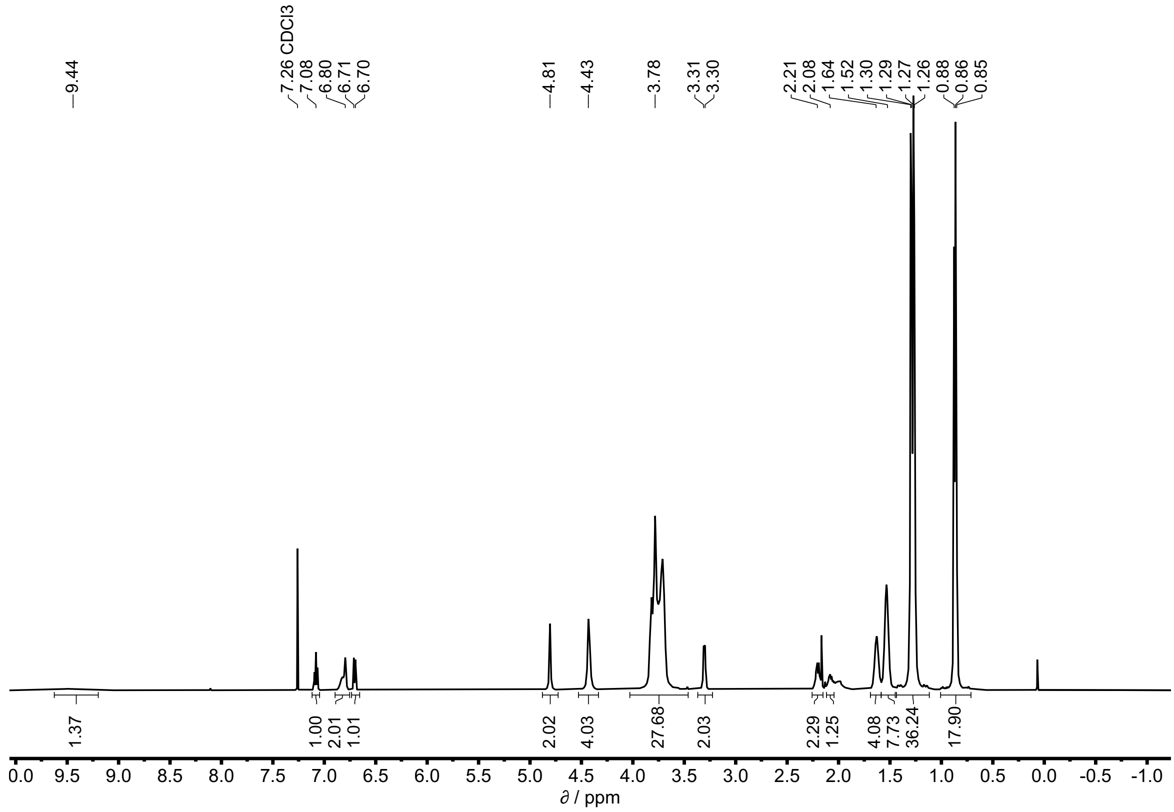

Figure S64. ^{1}H NMR spectrum (500 MHz, $CDCl_3$) of compound **ADA.**

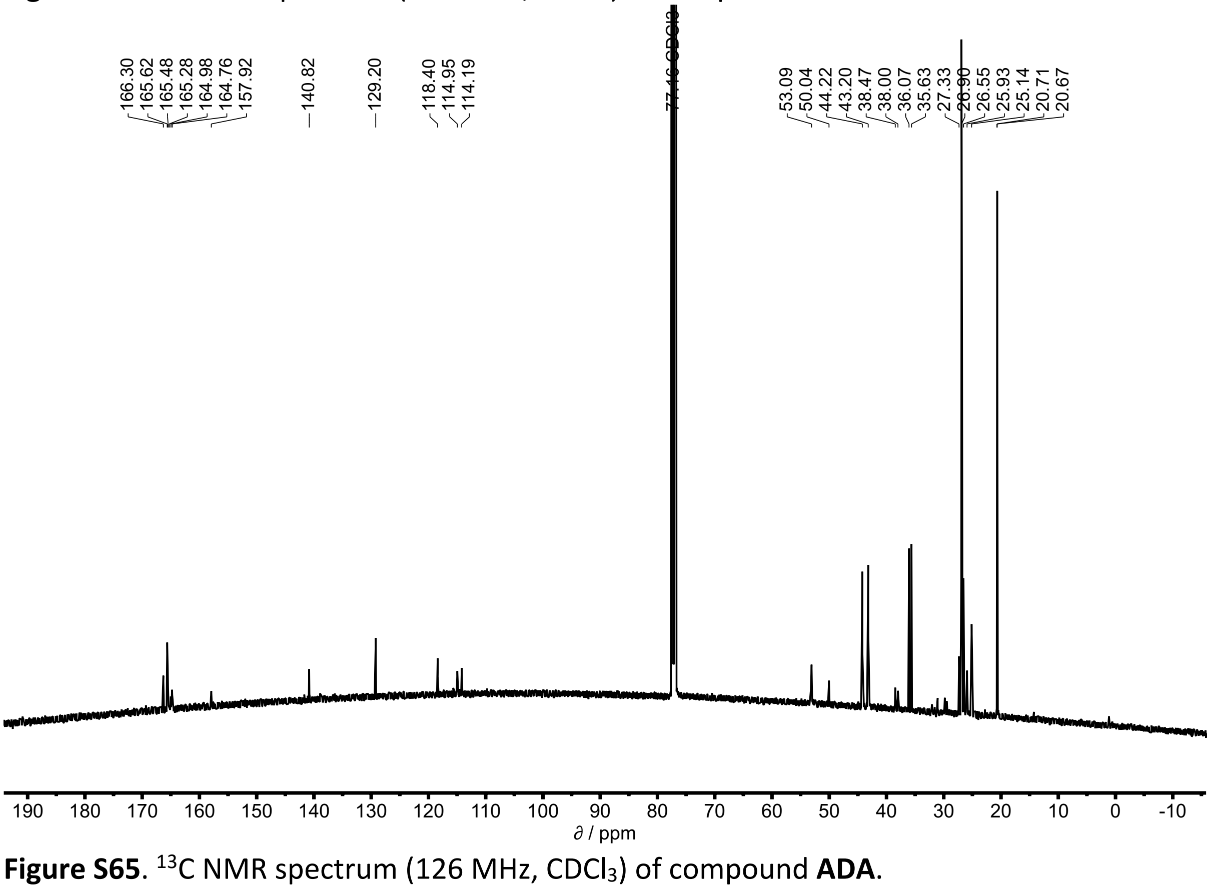

Figure S65. ^{13}C NMR spectrum (126 MHz, $CDCl_3$) of compound **ADA**.

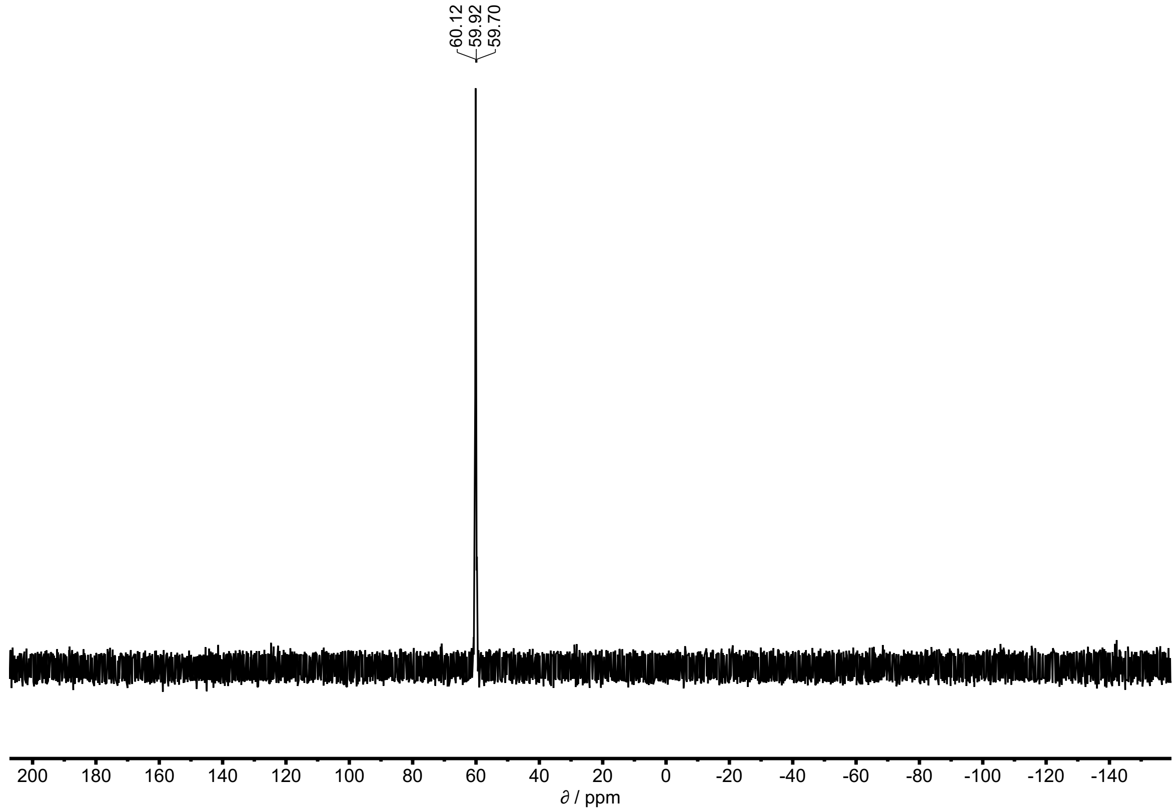

Figure S66. ^{31}P NMR spectrum (162 MHz, $CDCl_3$) of compound **ADA**.

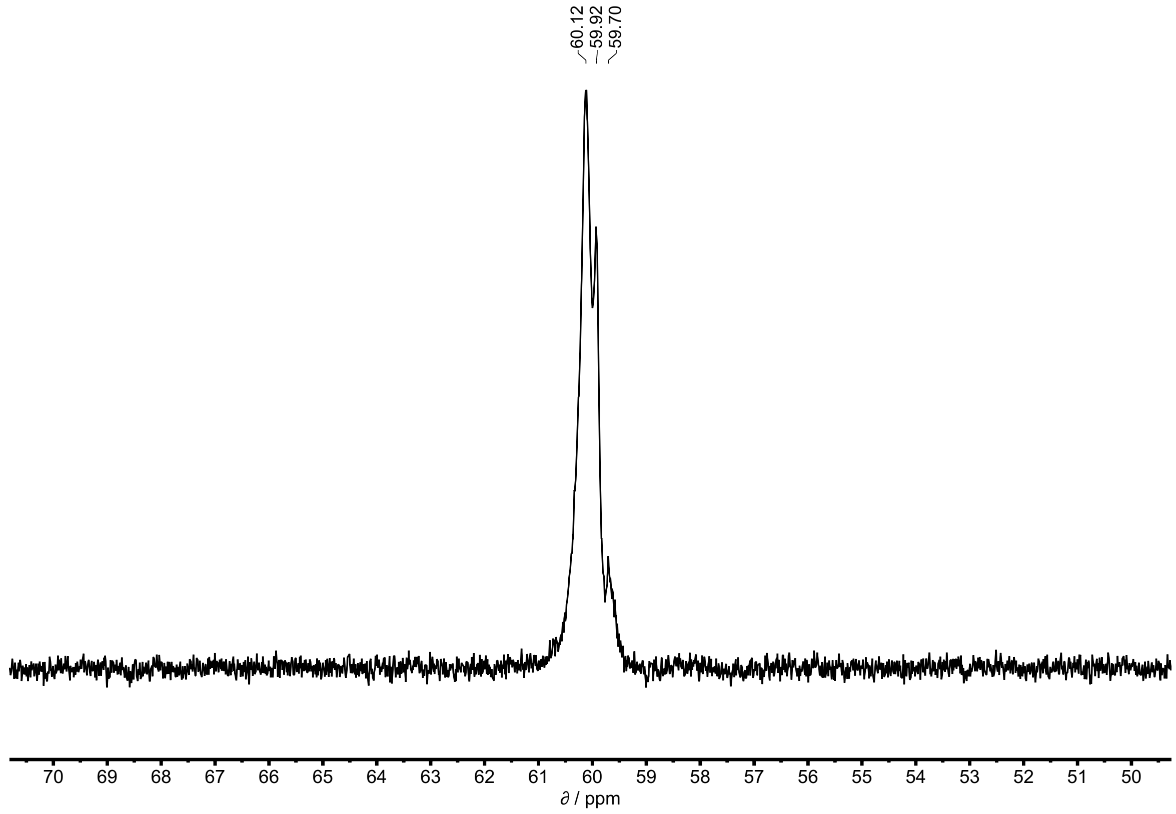

Figure S67. ^{31}P NMR spectrum (162 MHz, $CDCl_3$) of compound **ADA**.

Synthesis of **ADD**

ESI 2 (47 mg, 0.050 mmol, 1.0 eq) in THF (10 mL) was added to **8** (58 g, 0.101 mmol, 2.0 eq) in THF (5 mL) and DIPEA (18 µL, 13 mg, 0.101 mmol, 2.0 eq) and the reaction mixture was heated under reflux for 63 h. The solvent was removed *in vacuo* and the residue was dissolved in EtOAc (30 mL). The solution was washed with a solution of citric acid (5% in water, 3 x 10 mL). The aqueous wash was extracted with EtOAc (15 mL). The combined organic phases were dried ($MgSO_4$) and the solvent was evaporated *in vacuo*. The crude was purified by flash chromatography (SiO_2, 0-3% gradient of MeOH in DCM).

The thus obtained intermediate was dissolved in THF (3 mL) and TBAF (1M in THF, 110 µL, 0.110 mmol, 2.2 eq assuming quantitative conversion in the second step) was added to the solution. The reaction mixture was stirred at 22 °C for 15h. The reaction mixture was diluted with EtOAc (15 mL) and washed with sat. aqueous ammonium chloride solution (2 x 5 mL). The organic phase was dried ($MgSO_4$) and evaporated *in vacuo*. The crude was purified by flash chromatography (SiO_2, 0-6% gradient of MeOH in DCM) to yield the product as a white solid (7.4 mg, 0.0063 mmol, 6% yield over 3 steps).

^{1}H NMR (500 MHz, $CDCl_3$): δ_H 7.09 (br m, 2H), 6.74 (m, 6H), 4.79 (m, 4H), 4.44 (s, 2H), 4.10 – 3.07 (m, 30H), 2.18 (m, 1H), 2.08 (m, 2H), 1.74 – 1.44 (m, 12H, overlapping with HDO), 1.27 (m, 18H), 0.88 (m, 18H);

^{13}C NMR (126 MHz, $CDCl_3$): δ_C ^{13}C NMR (126 MHz, $CDCl_3$) δ 166.3, 165.6, 165.3, 165.0, 164.8, 156.6, 141.6 and 141.3, 129.4, 119.3 (br), 114.6, 113.9, 53.5, 53.2, 50.1, 44.3, 43.2, 38.3 (d, $^{1}J_{CP}$ = 75.6 Hz), 35.9 (d, $^{1}J_{CP}$ = 55.4 Hz), 29.8, 29.2, 27.5, 26.9, 26.6, 26.0, 25.1, 22.8, 20.7;

^{31}P NMR (162 MHz, $CDCl_3$): δ_P 61.08, 60.94;

FT-IR (ATR): ν_{max} /cm^{-1} 2955, 2929, 2853, 1523, 1477, 1427, 1386, 1367, 1297, 1256, 1204, 1179, 1131, 1093, 1026, 996, 954, 908, 852, 837, 805, 753, 733, 700, 666, 648, 504 449;

HRMS (ES+): calcd for $C_{62}H_{97}N_{18}O_3P + H^+$ is 1173.7807, found 1173.7803 (-0.3ppm).

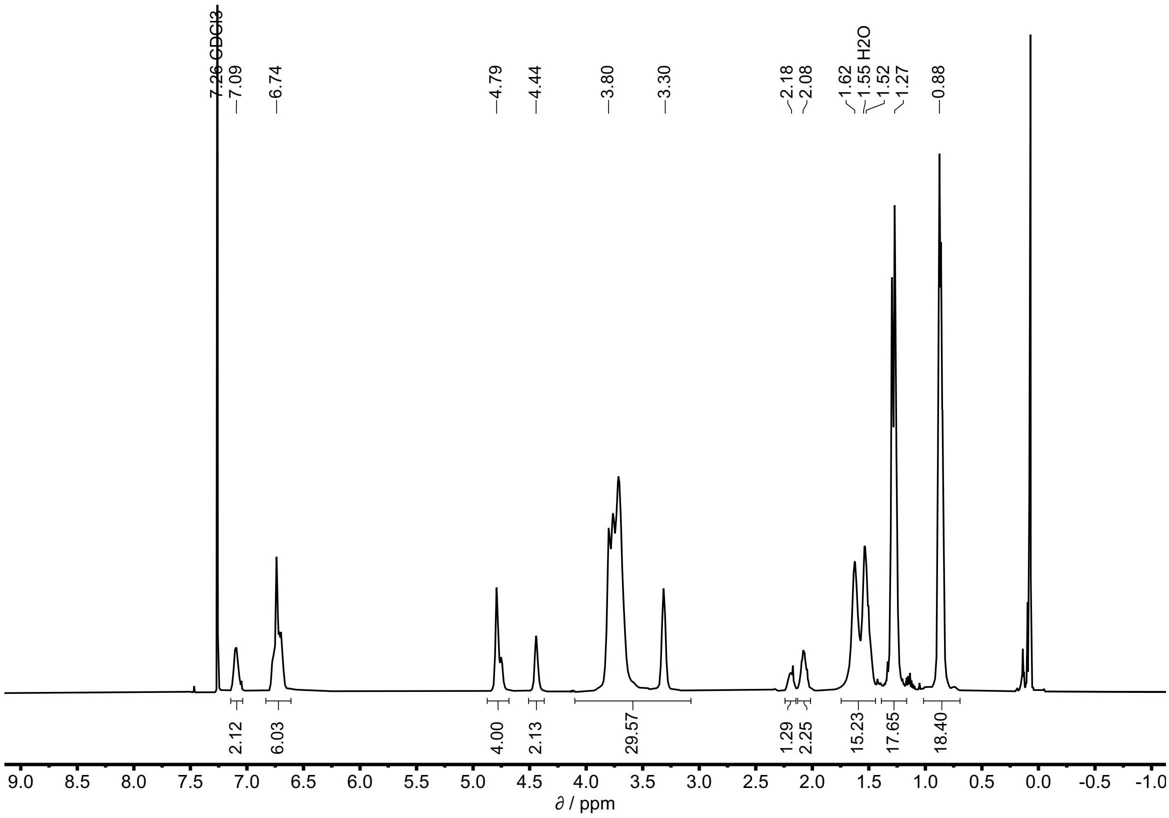

Figure S68. ^{1}H NMR spectrum (500 MHz, $CDCl_3$) of compound **ADD.**

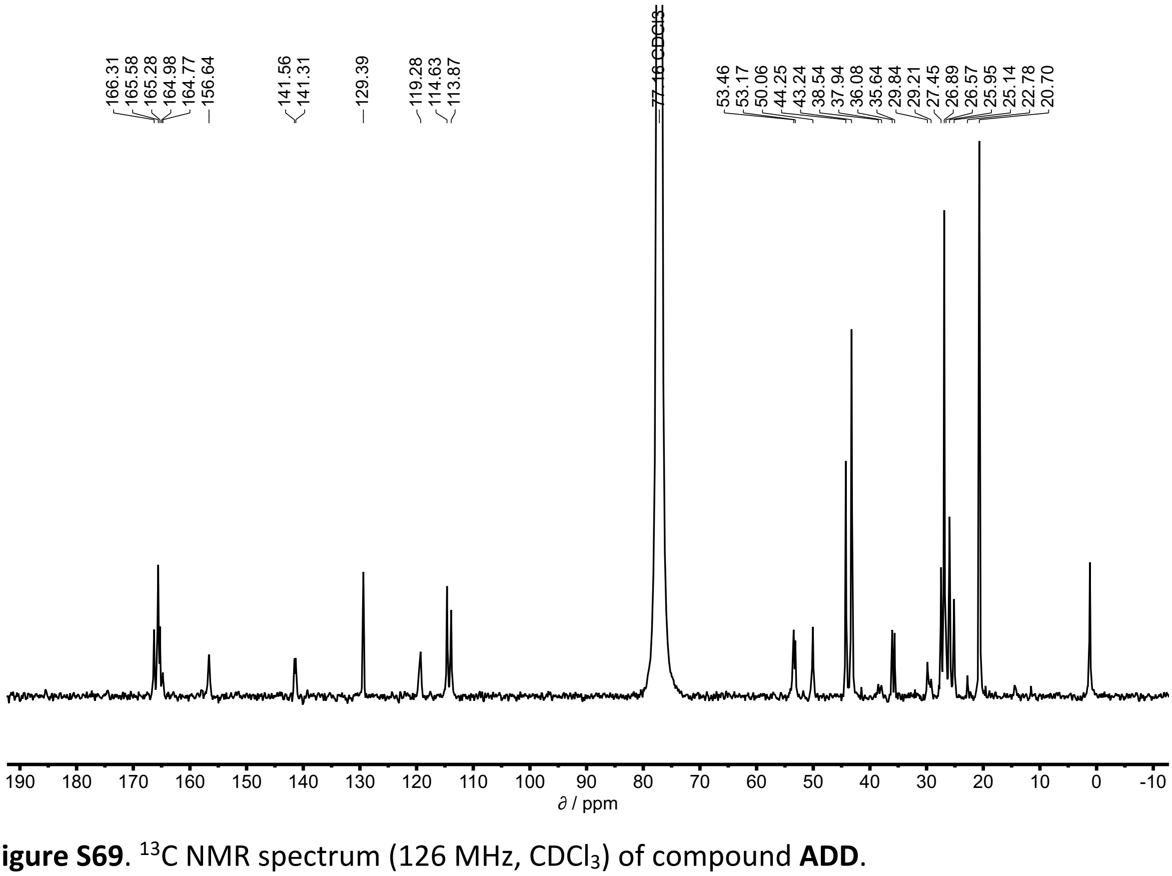

Figure S69. ^{13}C NMR spectrum (126 MHz, $CDCl_3$) of compound **ADD**.

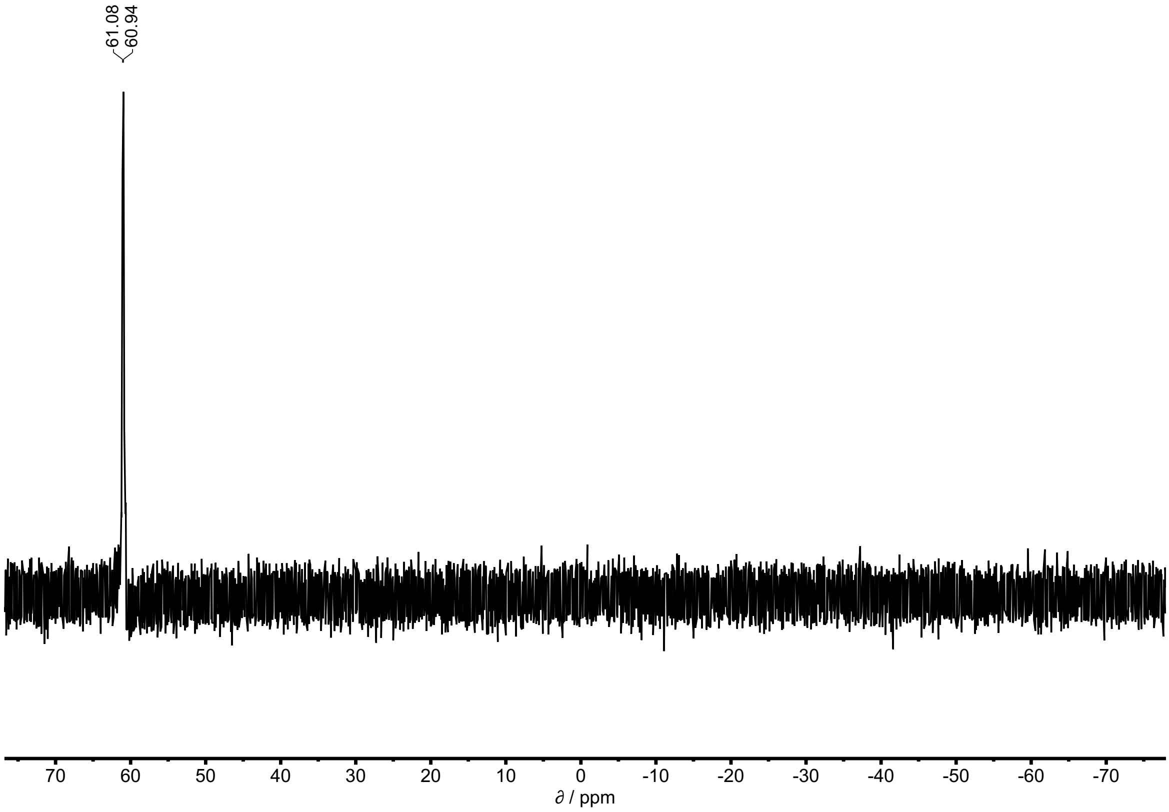

Figure S70. ^{31}P NMR spectrum (162 MHz, $CDCl_3$) of compound **DDA**.

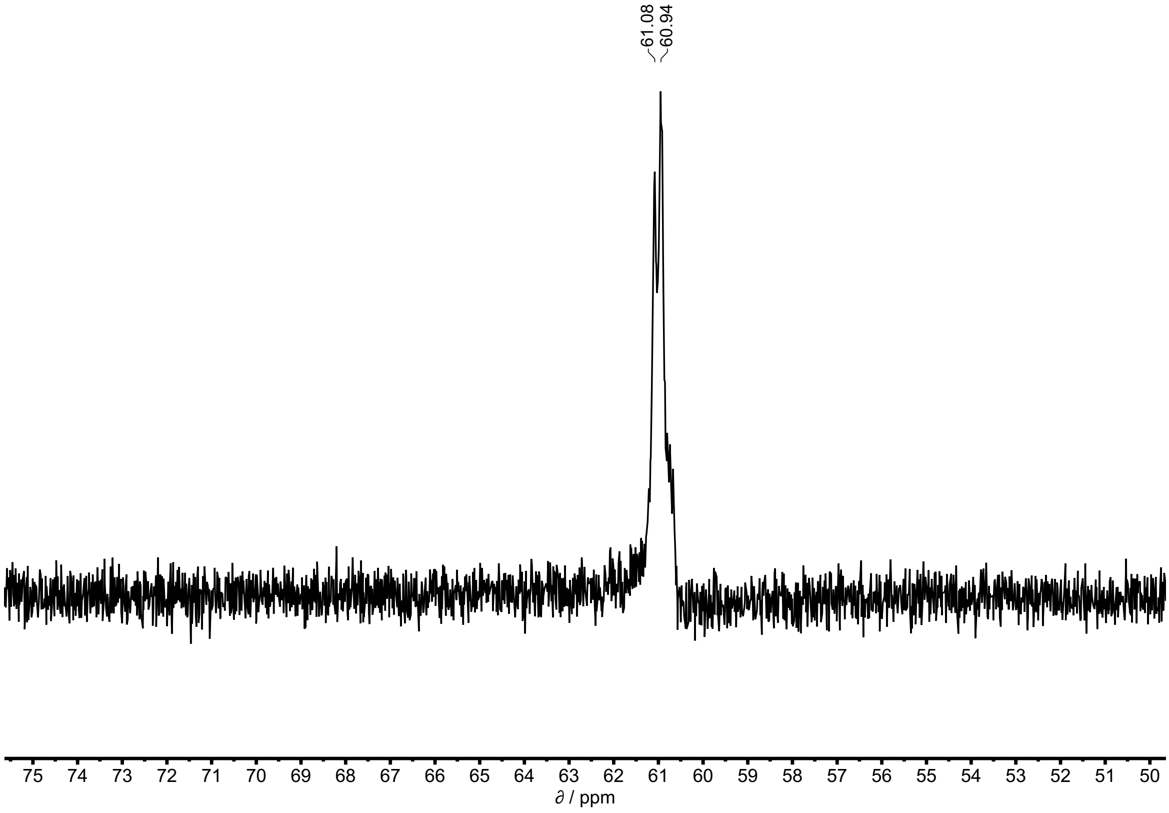

Figure S71. ^{31}P NMR spectrum (162 MHz, $CDCl_3$) of compound **DDA**.

Synthesis of **AAD**

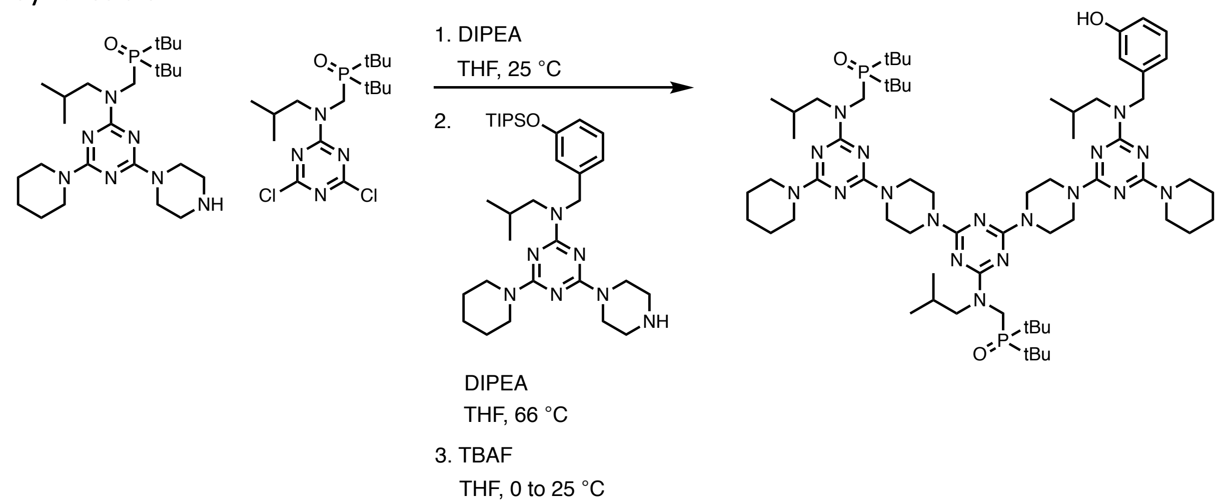

To a solution of **13** (100 mg, 0.203 mmol, 1.0 eq) and **9** (80 mg, 0.203 mmol, 1.0 eq) in THF (10 mL) was added DIPEA (71 µL, 53 mg, 0.406 mmol, 2.0 eq) and the reaction mixture was stirred at 22 °C for 40 h. The solvent was removed *in vacuo* and the residue was dissolved in EtOAc (40 mL). The solution was washed with a solution of citric acid (5% in water, 3 x 15 mL). The aqueous wash was extracted with EtOAc (15 mL). The combined organic phases were dried ($MgSO_4$) and the solvent was evaporated *in vacuo*.

To the thus obtained oil in THF (10 mL) was added **8** (187 mg, 0.321 mmol, 1.6 eq) in THF (10 mL) and DIPEA (178 µL, 132 mg, 1.02 mmol, 5.0 eq) and the reaction mixture was heated under reflux for 40 h. The solvent was removed *in vacuo* and the residue was dissolved in EtOAc (40 mL). The solution was washed with a solution of citric acid (5% in water, 3 x 15 mL). The aqueous wash was extracted with EtOAc (15 mL). The combined organic phases were dried ($MgSO_4$) and the solvent was evaporated *in vacuo*. The crude was purified by flash chromatography (SiO_2, 0-7% gradient of MeOH in DCM) to yield the TIPS-protected trimer (111 mg).

The thus obtained crude (111 mg, 0.0796 mmol assuming 100% purity, 1.0 eq) was dissolved in THF (5 mL) and TBAF (1M in THF, 0.159 mL, 0.159 mmol, 2.0 eq) was added to the solution at 0 °C. The reaction mixture was stirred at 22 °C for 15h. The reaction mixture was diluted with EtOAc (15 mL) and washed with sat. aqueous ammonium chloride solution (2 x 5 mL). The organic phase was dried ($MgSO_4$) and evaporated *in vacuo*. The crude was purified by flash chromatography (SiO_2, 0-7% gradient of MeOH in DCM) to yield the product as a white solid (54 mg, 0.044 mmol, 21% yield over 3 steps).

^{1}H NMR (500 MHz, CDCl$_3$): δ_H ^{1}H NMR (500 MHz, $CDCl_3$) δ 9.62 (br m, 1H), 7.07 (t, *J* = 7.7 Hz, 1H), 6.82 (m, 2H), 6.71 (d, *J* = 7.5 Hz, 1H), 4.79 (m, 2H), 4.43 (s, 4H), 3.77 (m, 28H), 3.30 (m, 2H), 2.20 (m, 2H), 2.08 (m, 1H), 1.67 – 1.46 (m, 12H), 1.29 (d, *J* = 13.0 Hz, 36H), 0.87 (m, 18H);
^{13}C NMR (126 MHz, CDCl$_3$): δ_C 166.3, 165.8, 165.7, 165.6, 165.5, 165.3 and 165.2, 165.1, 165.0, 164.7, 158.1 and 157.9, 141.0, 129.1, 118.4 and 118.3, 115.0, 114.1, 53.1, 50.1, 50.0, 44.2, 43.3, 43.2, 38.2 (d, $^1J_{CP}$ = 62Hz), 35.9 (d, $^1J_{CP}$ = 55 Hz), 35.8 (d, $^1J_{CP}$ = 55 Hz), 27.4, 27.3, 26.9, 26.5, 25.9, 25.9, 25.2, 25.1, 20.7, 20.7, 20.6;
^{31}P NMR (162 MHz, CDCl$_3$): δ_P 60.16;
FT-IR (ATR): ν_{max} /cm^{-1} 2953, 2930, 2852, 1523, 1475, 1425, 1386, 1366, 1296, 1255, 1239, 1204, 1176, 1132, 1096, 1023, 995, 931, 908, 853, 834, 806, 728, 669, 644, 582, 504, 448;
HRMS (ES+): calcd for $C_{64}H_{110}N_{18}O_3P_2$ + H^+ is 1241.8562, found 1241.8527 (-2.8ppm).

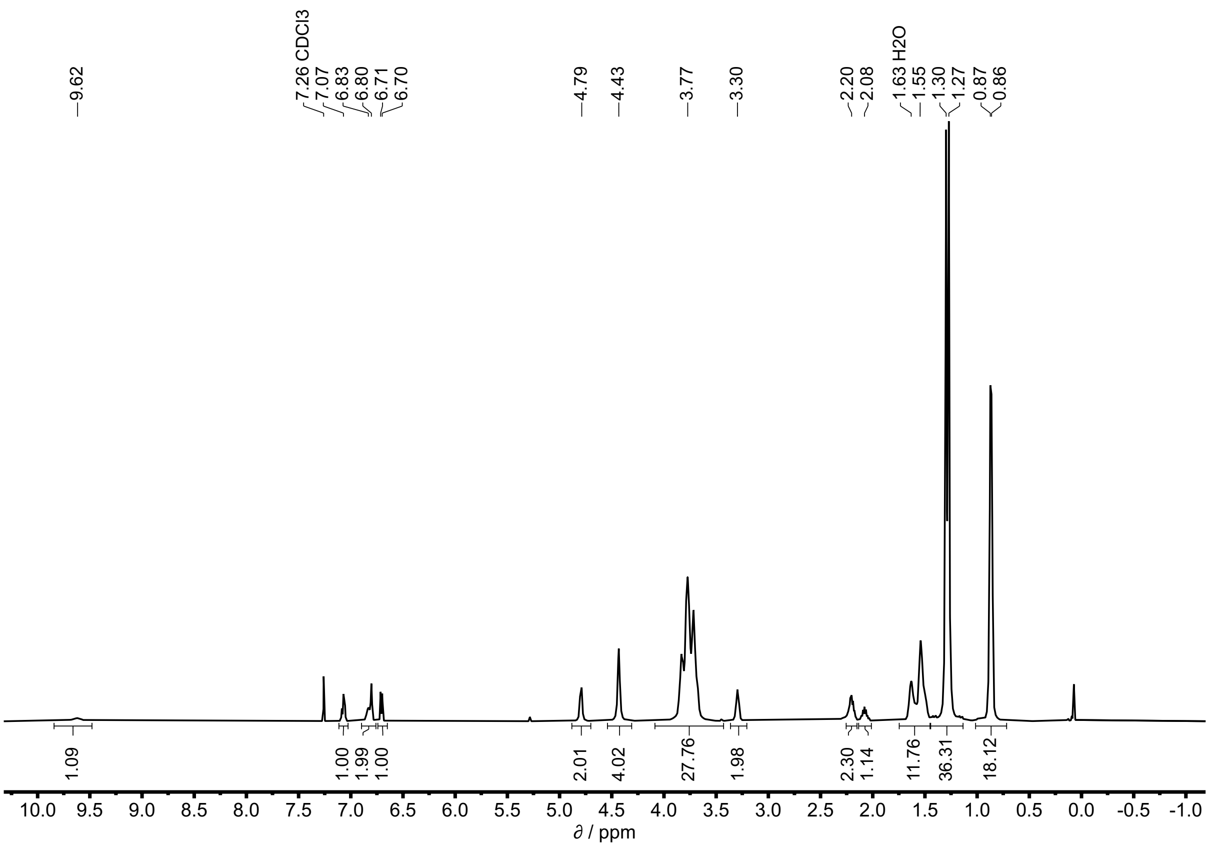

Figure S72. ^{1}H NMR spectrum (500 MHz, $CDCl_3$) of compound **AAD.**

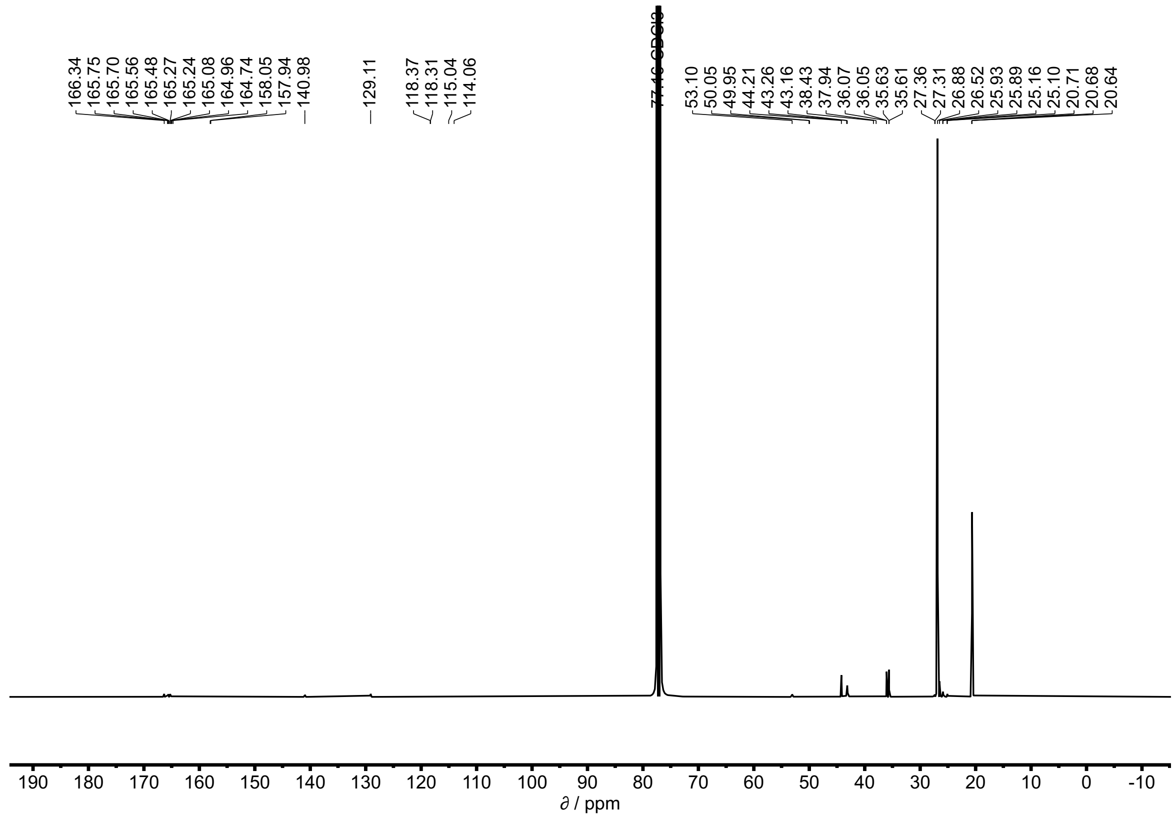

Figure S73. ^{13}C NMR spectrum (126 MHz, $CDCl_3$) of compound **AAD**.

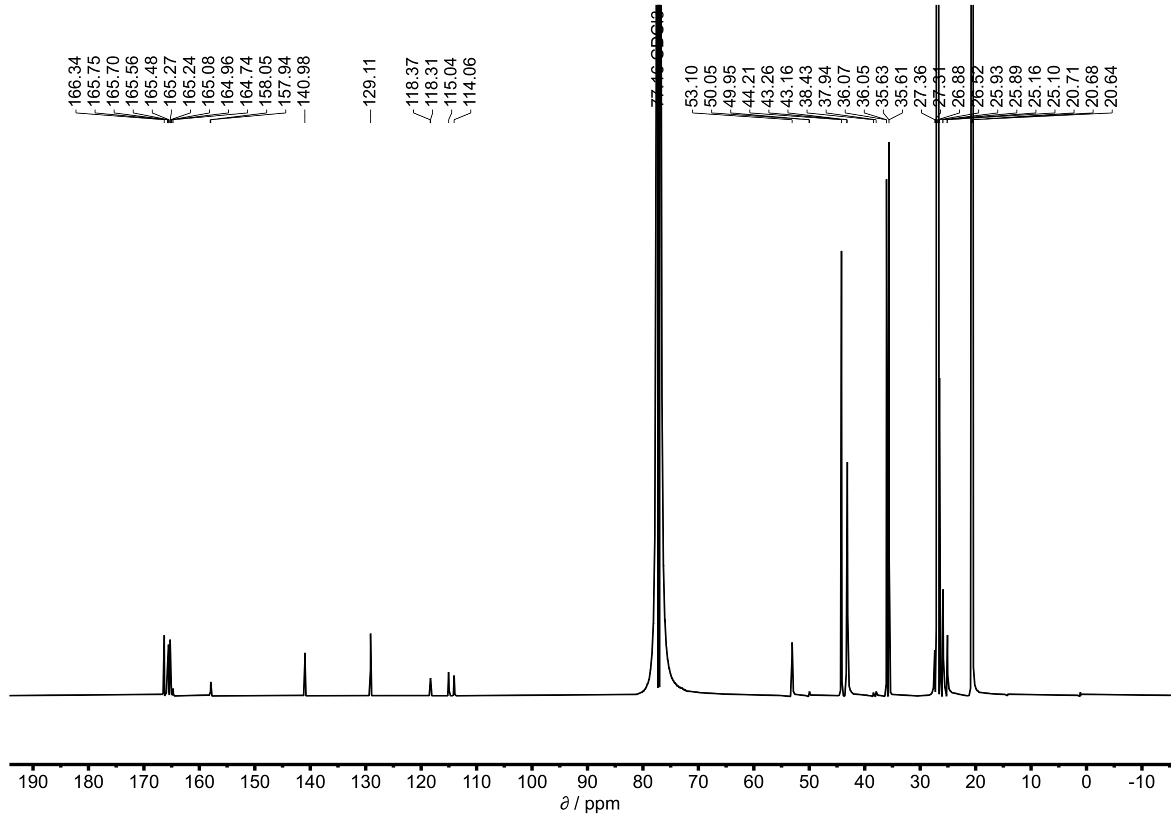

Figure S74. ^{13}C NMR spectrum (126 MHz, $CDCl_3$) of compound **AAD**.

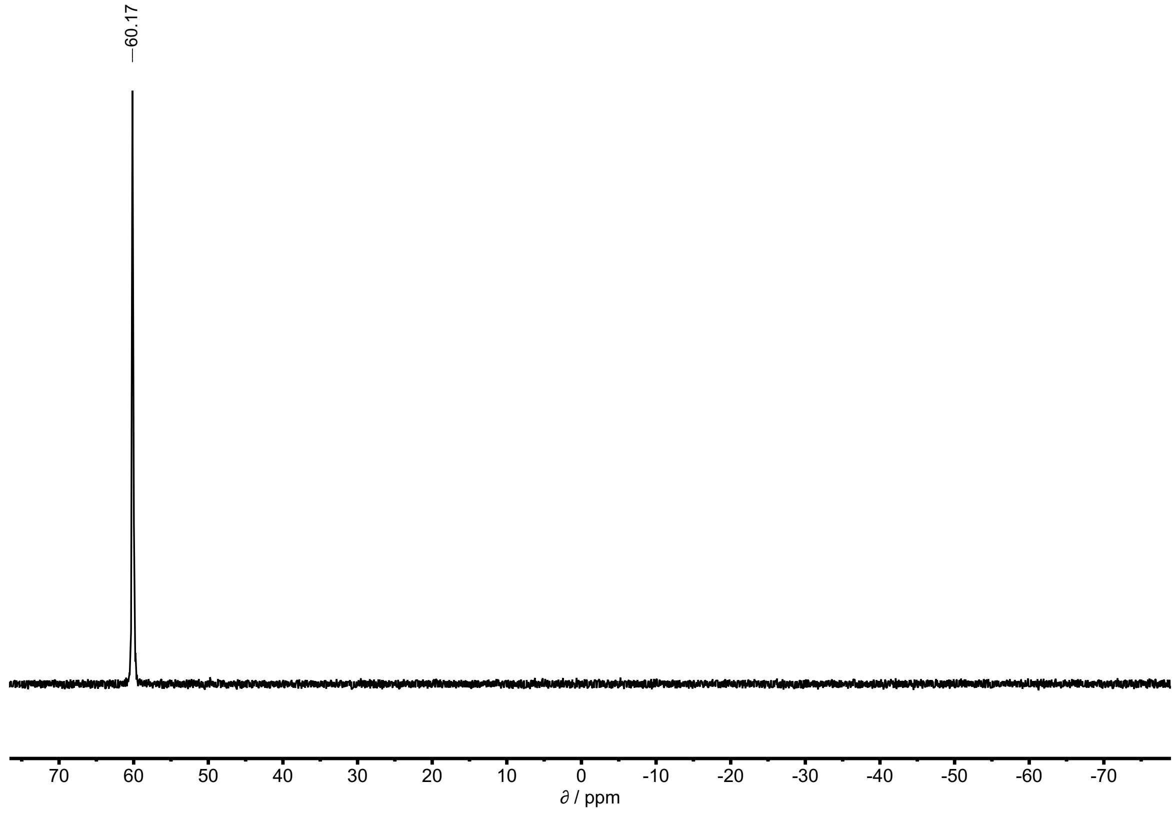

Figure S75. ^{31}P NMR spectrum (162 MHz, $CDCl_3$) of compound **AAD**.

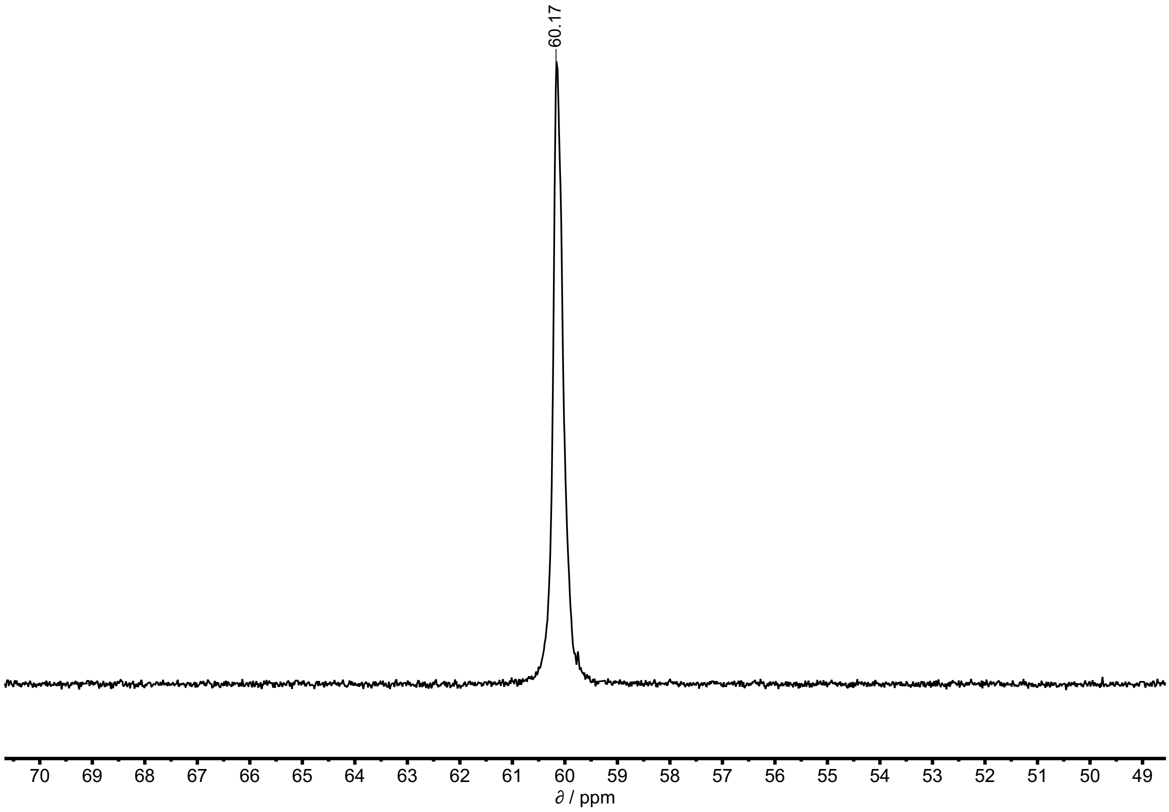

Figure S76. ^{31}P NMR spectrum (162 MHz, $CDCl_3$) of compound **AAD**.

Synthesis of 4-mers

Synthesis of **DDDD**

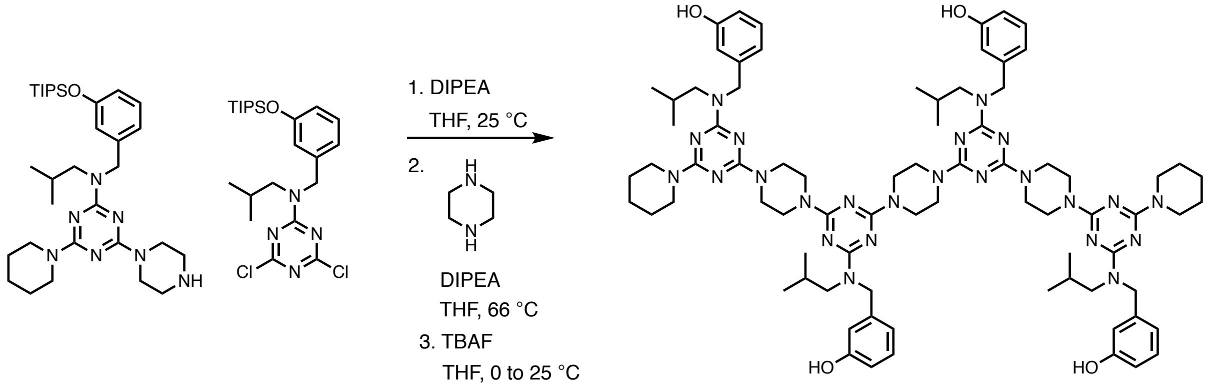

To a solution of **5** (100 mg, 0.207 mmol, 1.0 eq) and **8** (120 mg, 0.207 mmol, 1.0 eq) in THF (10 mL) was added DIPEA (72 μL, 54 mg, 0.414 mmol, 2.0 eq), and the reaction mixture was stirred for 15 h at 22 °C. The solvent was removed *in vacuo* and the residue was dissolved in EtOAc (0 mL). The solution was washed with a solution of citric acid (5% in water, 3 x 15 mL). The aqueous wash was extracted with EtOAc (15 mL). The combined organic phases were dried ($MgSO_4$) and the solvent was evaporated *in vacuo* to yield a transparent oil, which was purified by flash chromatography (SiO_2, 0-30% gradient of EtOAc in 40-60 Pet. Ether). The product of step 1 was obtained as a transparent oil (141 mg, 0.137 mmol, 66% yield).
To a solution of the thus obtained oil (141 mg, 0.137 mmol, 1.0 eq) in THF (10 mL) was added piperazine (5.9 mg, 0.069 mmol, 0.5 eq) and DIPEA (72 μL, 54 mg, 0.414 mmol, 6.0 eq) and the solution was heated under reflux for 39 h. The solvent was removed *in vacuo* and the residue was dissolved in EtOAc (40 mL). The solution was washed with a solution of citric acid (5% in water, 3 x 15 mL). The aqueous wash was extracted with EtOAc (15 mL). The combined organic phases were dried ($MgSO_4$) and the solvent was evaporated *in vacuo*. The crude was purified by flash chromatography (SiO_2, 0-30% gradient of ethyl EtOAc in 40-60 Pet. Ether) to yield the product of step 2 as a transparent oil (51.7 mg, 0.025 mmol, 36% yield).
To a solution of the thus obtained oil (51.7 mg, 0.025 mmol, 1.0 eq) in THF (3 mL) at 0 °C was added dropwise a solution of TBAF (1M in THF, 0.125 mL, 0.125 mmol, 5.0 eq), and the reaction mixture was stirred at 22 °C for 15 h. The reaction mixture was diluted with EtOAc (15 mL) and washed with sat. aqueous ammonium chloride solution (2 x 5 mL). The organic phase was dried ($MgSO_4$) and evaporated *in vacuo*. The crude was purified by flash chromatography (SiO_2, 0-50% gradient of EtOAc in 40-60 Pet Ether). The product was afforded as a white solid (11.3 mg, 0.0099 mmol, 39% yield).

^{1}H NMR (400 MHz, THF-*d*$_8$): δ_H 8.08 (m, 4H), 7.01 (m, 4H), 6.69 – 6.53 (m, 12H), 4.80 (br m, 8H), 3.75 (m, 32H), 3.31 (m, 8H), 2.13 (m, 4H), 1.68 – 1.42 (m, 12H), 0.88 (m, 24H);
^{13}C NMR (101 MHz, THF-*d*$_8$): δ_C 167.11 and 167.08, 166.17 (br), 165.85, 158.66, 141.71 and 141.55, 129.60 and 129.55, 118.92 (br), 114.61 (br), 114.36 and 114.31, 53.81, 50.37, 44.63, 43.75, 27.97 and 27.95, 26.54 (br), 25.79, 20.63 and 20.62;
FT-IR (ATR): ν_{max} /cm^{-1} 3291, 2953, 2928, 2852, 1590, 1524, 1479, 1429, 1386, 1367, 1298, 1256, 1205, 1179, 1155, 1098, 1031, 997, 806, 780, 747, 697;
HRMS (ES+): calcd for $C_{78}H_{108}N_{24}O_4 + H^+$ is 1445.9064, found 1445.9030 (-2.4ppm).

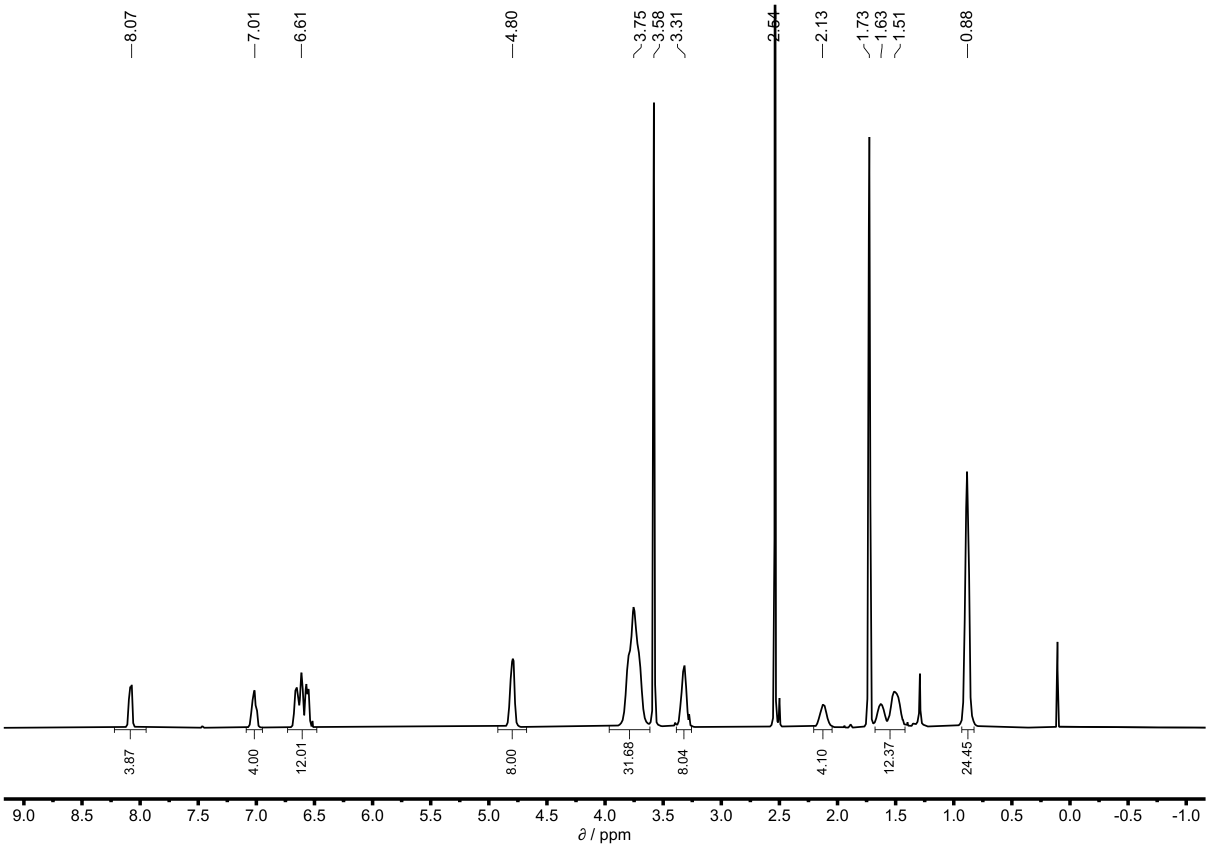

Figure S77. ^{1}H NMR spectrum (400 MHz, THF-d_8) of compound **DDDD.**

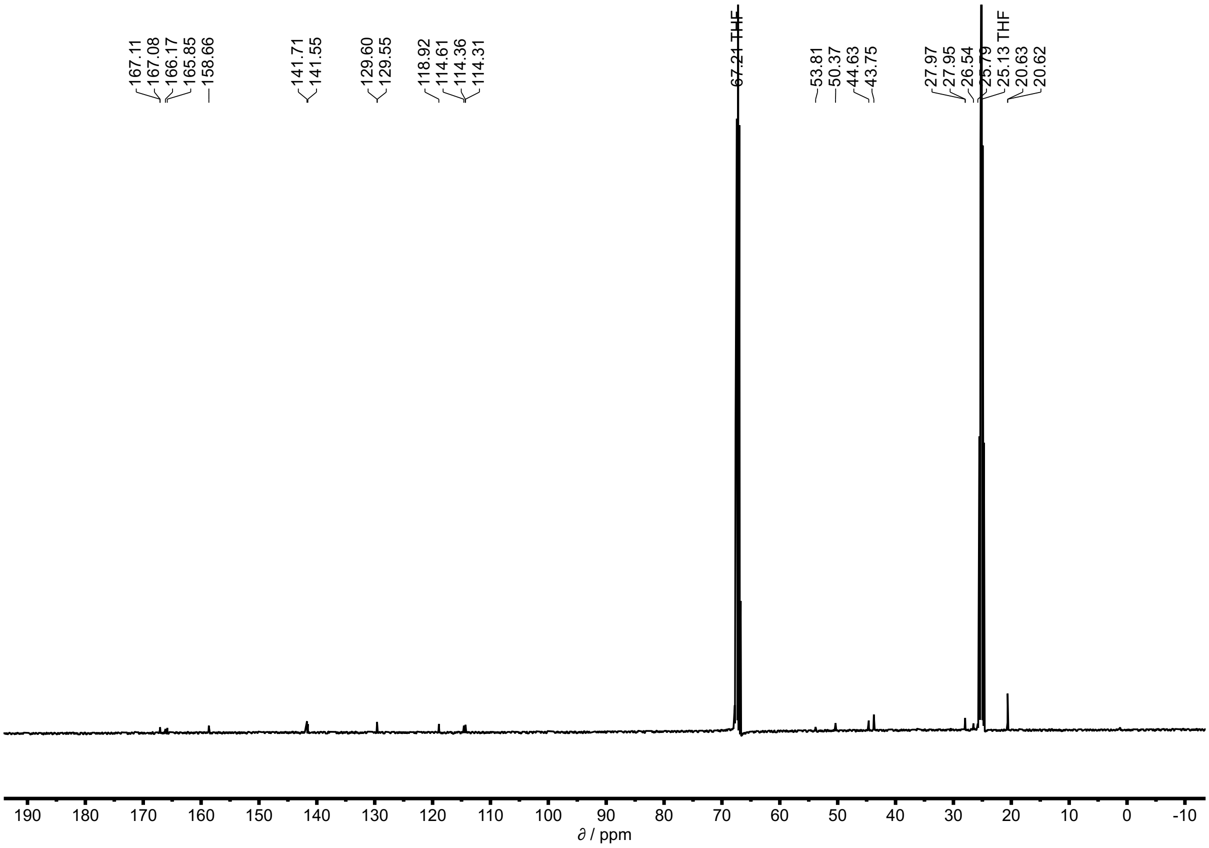

Figure S78. ^{13}C NMR spectrum (101 MHz, THF-d_8) of compound **DDDD**.

Synthesis of **AAAA**

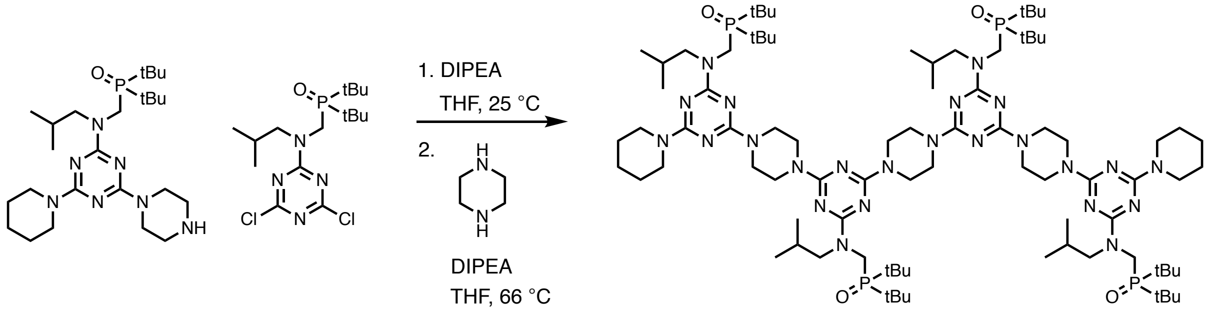

To a solution of **13** (125 mg, 0.253 mmol, 1.0 eq) and **9** (100 mg, 0.253 mmol, 1.0 eq) in THF (10 mL) was added DIPEA (88 µL, 65 mg, 0.51 mmol, 2.0 eq), and the reaction mixture was stirred for 15 h at 22 °C. The solvent was removed *in vacuo* and the residue was dissolved in EtOAc (0 mL). The solution was washed with a solution of citric acid (5% in water, 3 x 15 mL). The aqueous wash was extracted with EtOAc (15 mL). The combined organic phases were dried ($MgSO_4$) and the solvent was evaporated *in vacuo* to yield a transparent oil, which was purified by flash chromatography (SiO_2, 0-5% gradient of MeOH in DCM). The product of step 1 was obtained as a transparent oil (40.1 mg, 0.047 mmol, 19% yield).
To a solution of the thus obtained oil (40.1 mg, 0.047 mmol, 1.0 eq) in THF (10 mL) was added piperazine (2.0 mg, 0.024 mmol, 0.5 eq) and DIPEA (50 µL, 37 mg, 0.28 mmol, 6.1 eq) and the solution was heated under reflux for 39 h. The solvent was removed *in vacuo* and the residue was dissolved in EtOAc (20 mL). The solution was washed with a solution of citric acid (5% in water, 3 x 10 mL). The aqueous wash was extracted with EtOAc (10 mL). The combined organic phases were then washed with 1M NaOH solution (3 x 10 mL). The organic phase was dried ($MgSO_4$) and the solvent was evaporated *in vacuo*. The crude was purified by flash chromatography (SiO_2, 0-15% gradient of MeOH in DCM) to yield the product as a transparent oil (11.0 mg, 0.0064 mmol, 27% yield).

^{1}H NMR (400 MHz, $CDCl_3$): δ_H 4.40 (s, 8H), 3.90 – 3.68 (m, 40H), 2.21 (m, 4H), 1.67 – 1.49 (m, 12H), 1.28 (d, $^3J_{CP}$ = 13.1, 72H), 0.89 (m, 24H);
^{13}C NMR (126 MHz, $CDCl_3$): δ_C 165.6, 165.4, 165.2, 165.0, 164.8, 53.1 (br), 44.3, 43.2 (br), 38.2 (br d, $^1J_{CP}$ = 59.2 Hz), 35.9 and 35.9 (both d, $^1J_{CP}$ = 55.4 Hz), 26.9 (br), 26.6, 26.0 and 26.0, 25.1, 20.7;
^{31}P NMR (162 MHz, $CDCl_3$): δ_P 58.81 (broad);
FT-IR (ATR): ν_{max} /cm^{-1} 2954, 2923, 2853, 1528, 1479, 1430, 1388, 1367, 1294, 1257, 1206, 1139, 1093, 1019, 997, 832, 803, 732, 701, 646, 505, 449;
HRMS (ES+): calcd for $C_{86}H_{160}N_{24}O_4P_4 + H^+$ is 1718.2083, found 1718.2080 (-0.2ppm).

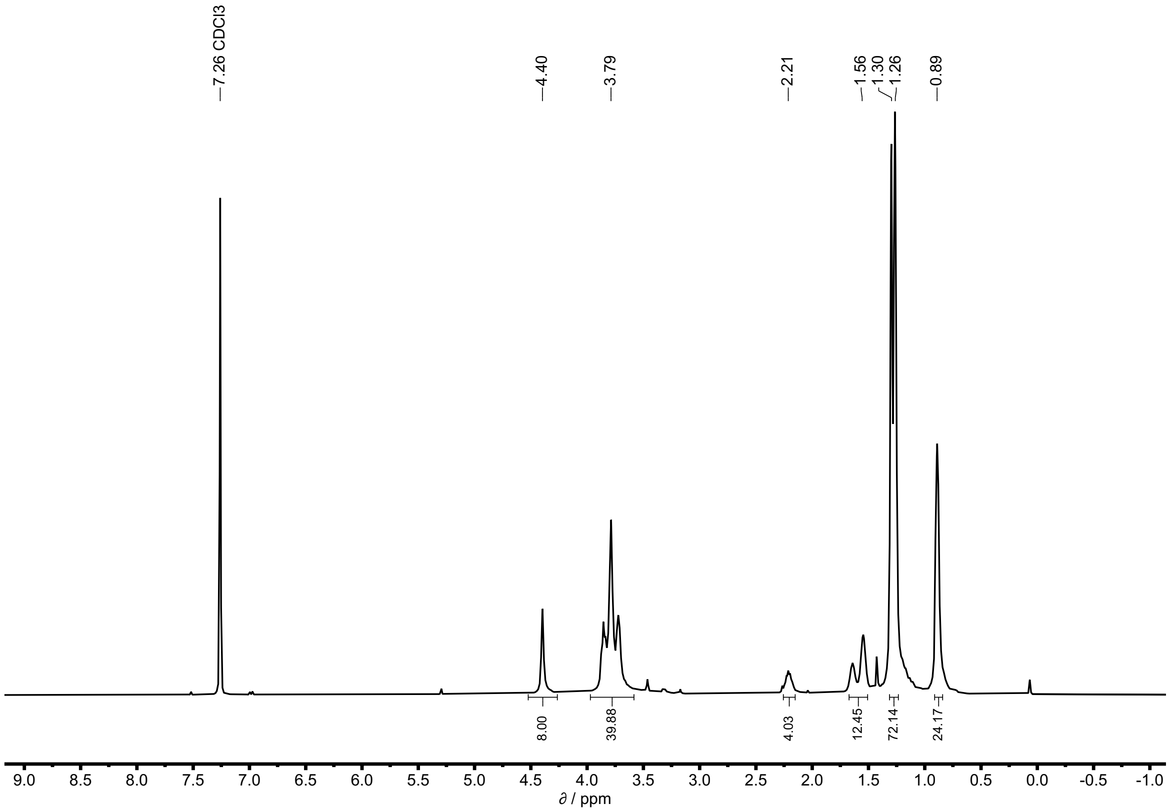

Figure S79. ^{1}H NMR spectrum (400 MHz, $CDCl_3$) of compound **AAAA.**

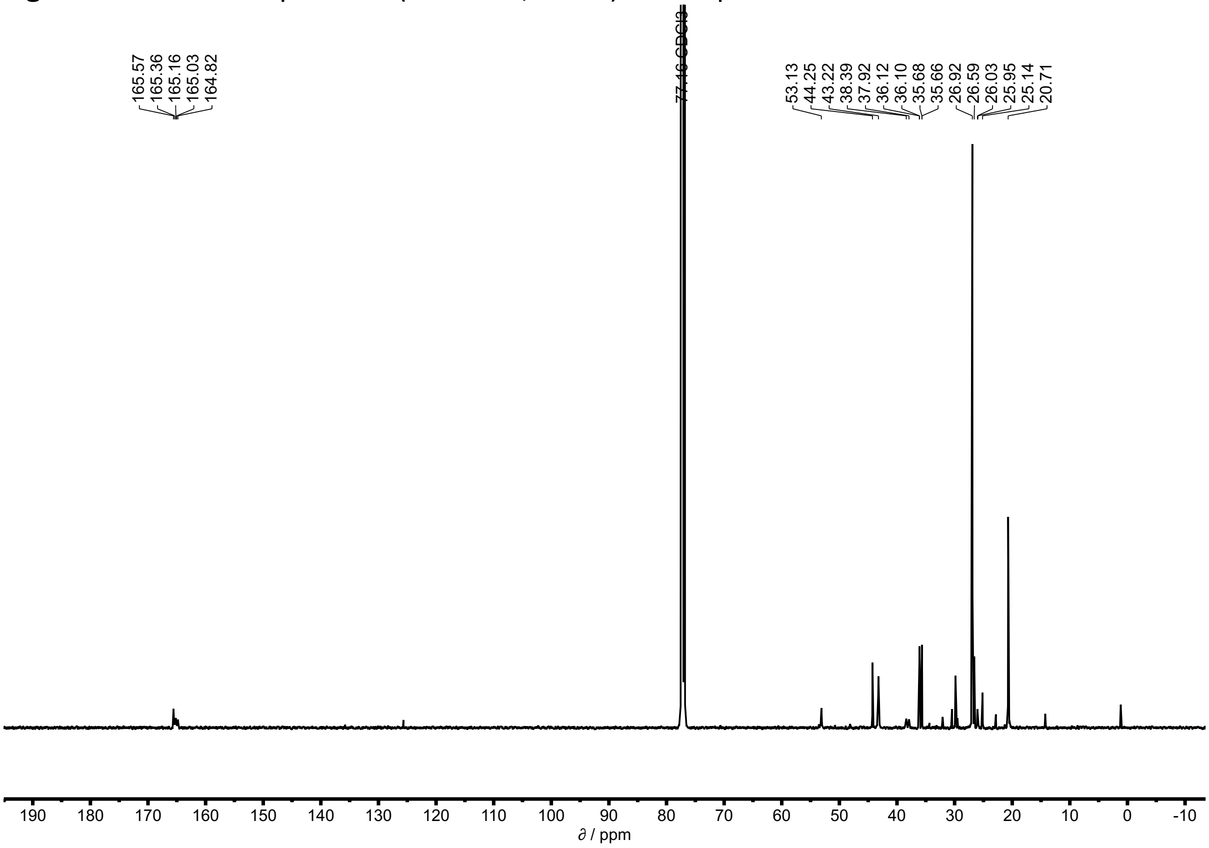

Figure S80. ^{13}C NMR spectrum (126 MHz, $CDCl_3$) of compound **AAAA**.

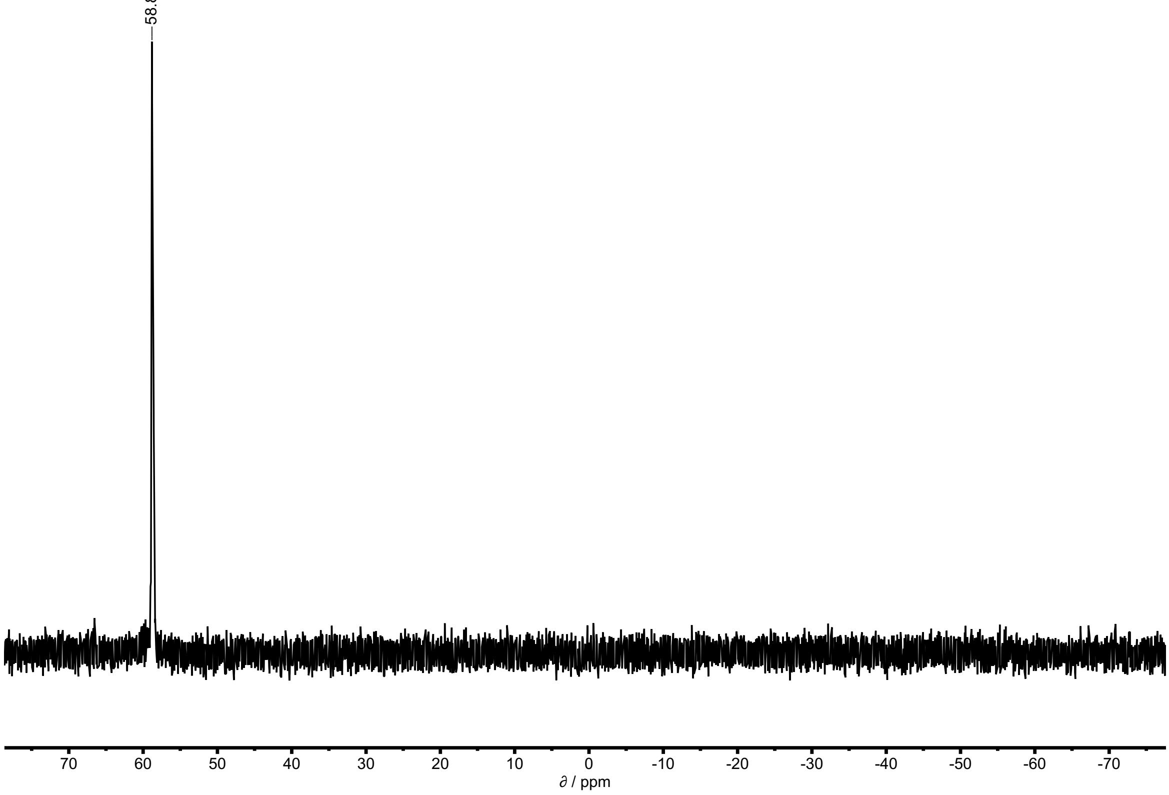

Figure S81. ^{31}P NMR spectrum (162 MHz, $CDCl_3$) of compound **AAAA**.

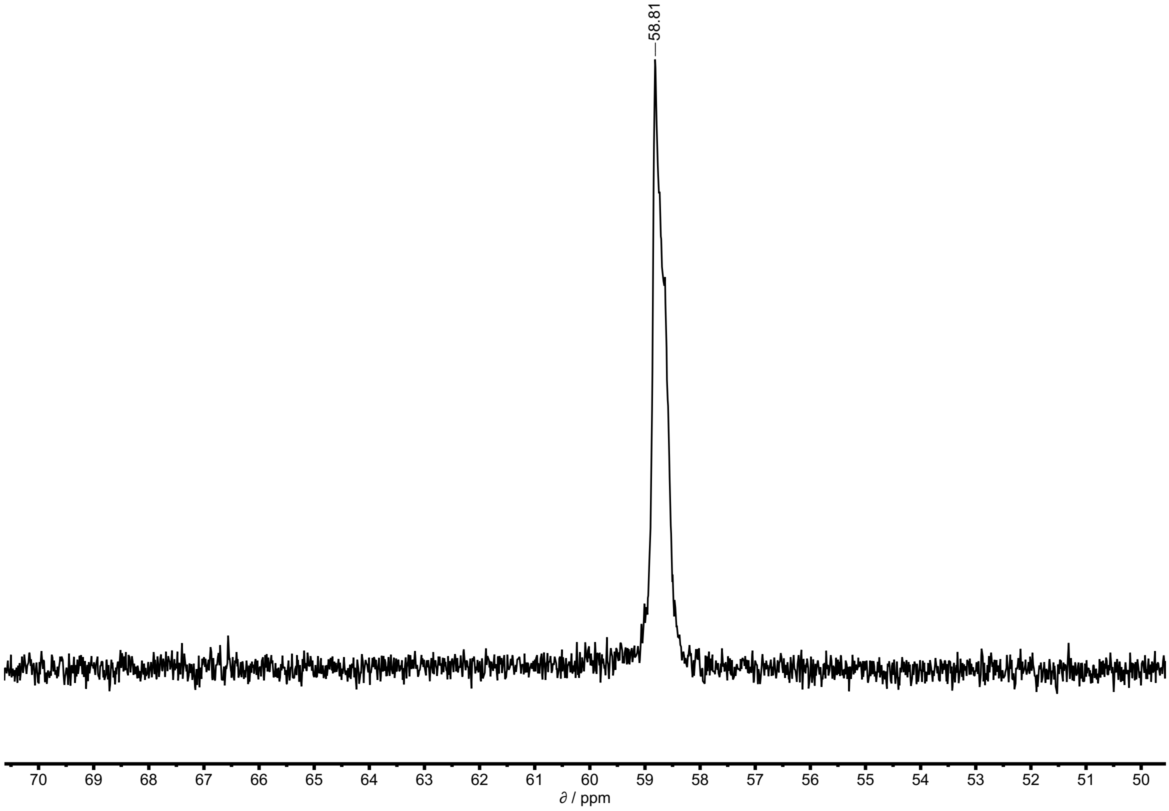

Figure S82. ^{31}P NMR spectrum (162 MHz, $CDCl_3$) of compound **AAAA**.

^{31}P NMR titration and dilution experiments

Titration of **D** into **A**

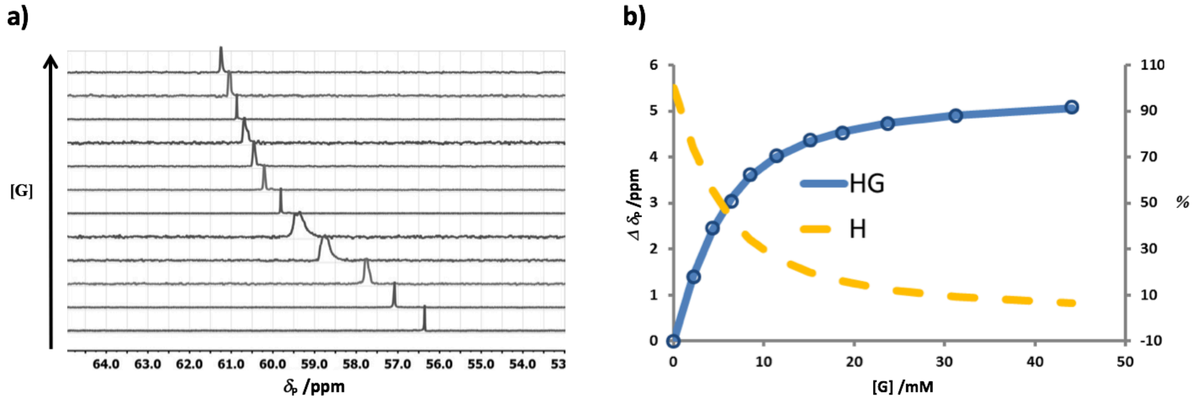

Figure S83. Titration experiment used to determine the binding constant between compounds **A** and **D**. a) The 202 MHz ^{31}P NMR spectra corresponding to the titration D into A in toluene-d_8 at 298 K. b) The line is the best fit of the weighted-average chemical shifts to a 1:1 binding isotherm with K = 361 M^{-1} and Δ∂ = 5.3 ppm.

Dilution of **AD**

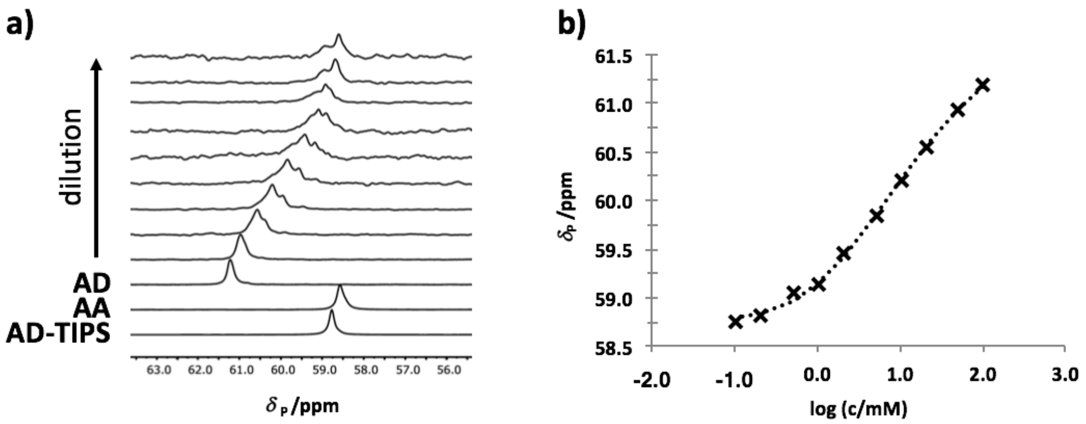

Figure S84. Dilution experiment used to determine the self-association constant of compound **AD**. a) The 202 MHz ^{31}P NMR spectra corresponding to the dilution of **AD** in $CDCl_3$ at 298 K. The spectra corresponding to **AA** and TIPS-protected **AD** are also shown for reference. b) The line is the best fit of the weighted-average chemical shifts to a 1:1 binding isotherm with K = 100 M^{-1} and Δ∂ = 3.2 ppm.

Dilution of **AAD**

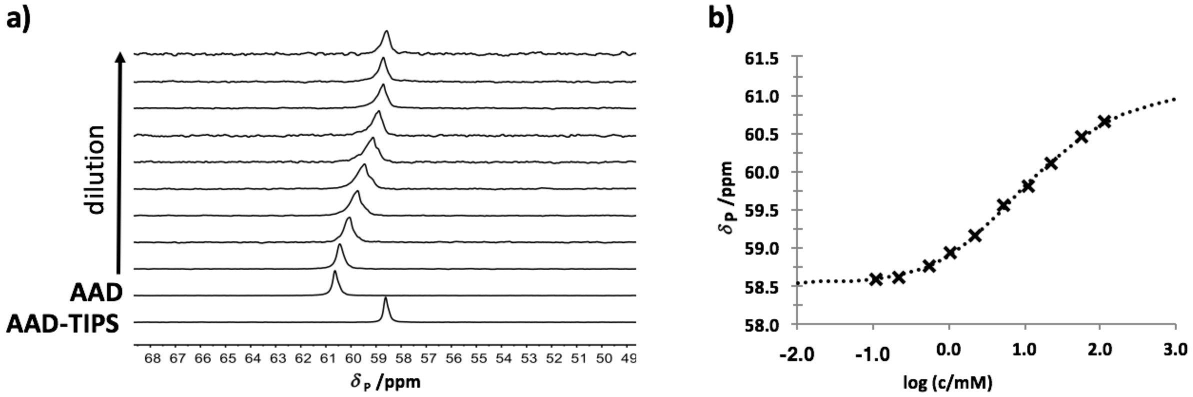

Figure S85. Dilution experiment used to determine the self-association constant of compound **AAD**. a) The 202 MHz ^{31}P NMR spectra corresponding to the dilution of **AAD** in $CDCl_3$ at 298 K. The spectrum corresponding to TIPS-protected **AAD** is also shown for reference. b) The line is the best fit of the weighted-average chemical shifts to a 1:1 binding isotherm with K = 100 M^{-1} and $\Delta\partial$ = 2.6 ppm.

Dilution of **ADD**

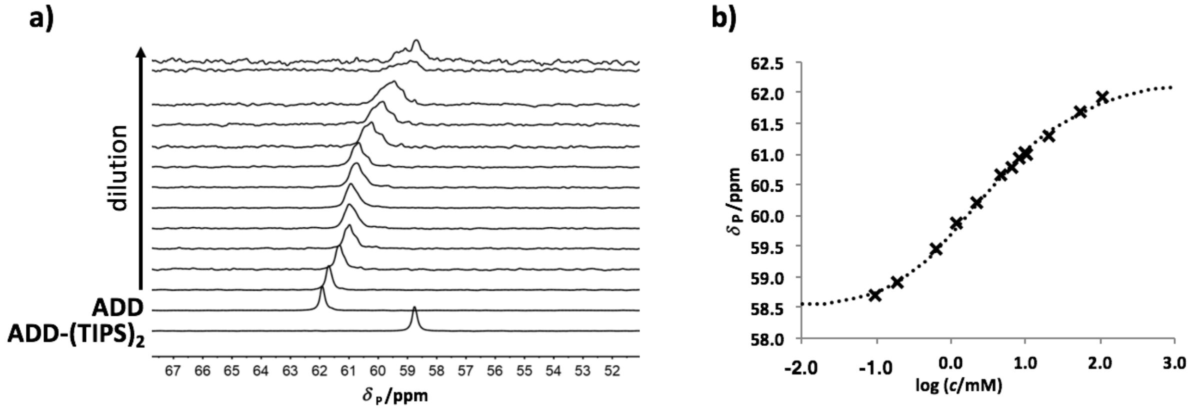

Figure S86. Dilution experiment used to determine the self-association constant of compound **ADD**. a) The 202 MHz ^{31}P NMR spectra corresponding to the dilution of **ADD** in $CDCl_3$ at 298 K. The spectrum corresponding to TIPS-protected **ADD** is also shown for reference. b) The line is the best fit of the weighted-average chemical shifts to a 1:1 binding isotherm with K = 310 M^{-1} and $\Delta\partial$ = 3.7 ppm.

Isothermal Titration Calorimetry

ITC Dilution Experiments

To study dimerization of the individual oligomers, the compound was dissolved in HPLC grade toluene with a concentration 10-100 times the expected dissociation constant and loaded into the injection syringe. Pure solvent was loaded into the sample cell of the microcalorimeter. The number of injections was between 40 and 60, and the volume of the injections was 5-7 µL. The thermogram peaks were integrated using Microcal Origin, and the resulting data were fit to a dimerization isotherm using purpose-written macros in Microsoft Excel. The macros use an iterative procedure to fit the experimental data to the following equations to determine the optimum values for the association constant (K) and the enthalpy of dimerization ($\Delta H°$).

$$[\mathrm{Gt}]_s = [\mathrm{G}]_s + 2[\mathrm{G{\bullet}G}]_s \qquad \text{(S1)}$$
$$[\mathrm{G{\bullet}G}]_s = K[\mathrm{G}]_s^2 \qquad \text{(S2)}$$

where $[\mathrm{Gt}]_s$ is the total concentration in the syringe
$[\mathrm{G}]_s$ is the concentration of monomeric species in the syringe
$[\mathrm{G{\bullet}G}]_s$ is the concentration of dimer in the syringe

$$[\mathrm{Gt}]_i = [\mathrm{G}]_i + 2[\mathrm{G{\bullet}G}]_i \qquad \text{(S3)}$$
$$[\mathrm{G{\bullet}G}]_i = K[\mathrm{G}]_i^2 \qquad \text{(S4)}$$

where $[\mathrm{Gt}]_i$ is the total concentration in the cell after the i-th injection
$[\mathrm{G}]_i$ is the concentration of monomeric species in the cell after the i-th injection
$[\mathrm{G{\bullet}G}]_i$ is the concentration of dimer in the cell after the i-th injection

The integrated molar heat of the i-th injection (Q_i) is given by

$$Q_i = Q_0 + 2\frac{V([\mathrm{G{\bullet}G}]_i - [\mathrm{G{\bullet}G}]_{i-1}) + V_i([\mathrm{G{\bullet}G}]_{i-1} - [\mathrm{G{\bullet}G}]_s)}{V_i[\mathrm{Gt}]_s}\Delta H° \qquad \text{(S5)}$$

where Q_0 is the baseline correction that is usually of the order 1 kJ mol^{-1}
V is the volume of the cell
V_i is the volume of the i-th injection

ITC Titration experiments

In a typical ITC titration experiment, one of the components of the complex (the host, H) was dissolved in HPLC grade toluene with a concentration 10-100 times the expected dissociation constant, and the solution was loaded into the sample cell of the microcalorimeter. A solution of the second component (the guest, G), which was 8-10 times more concentrated than the cell solution, was loaded into the injection syringe. The number of injections was between 40 and 60, and the volume of the injections was 5-7 µL. The thermogram peaks were integrated using Microcal Origin, and the resulting data were fit to a 1:1 binding isotherm using purpose-written macros in Microsoft Excel. Where required, an isotherm that also allowed for dimerization of both host and guest was used. The previously determined parameters that describe the thermodynamics of dimerization ($K_{H•H}$, $K_{G•G}$, $\Delta H°_{H•H}$ and $\Delta H°_{G•G}$) were used as fixed values, and an iterative procedure was used to fit the experimental data to the following equations to determine the optimum values for the host•guest association constant ($K_{H•G}$) and the enthalpy of binding ($\Delta H°_{H•G}$).

$$[\mathrm{Gt}]_s = [\mathrm{G}]_s + 2[\mathrm{G•G}]_s \quad \text{(S6)}$$
$$[\mathrm{G•G}]_s = K[\mathrm{G}]_s^2 \quad \text{(S7)}$$

where $[Gt]_s$ is the total concentration in the syringe
$[G]_s$ is the concentration of monomeric species in the syringe
$[G•G]_s$ is the concentration of dimer in the syringe

$$[\mathrm{Ht}]_i = [\mathrm{H}]_i + 2[\mathrm{H•H}]_i + [\mathrm{H•G}]_i \quad \text{(S8)}$$
$$[\mathrm{Gt}]_i = [\mathrm{G}]_i + 2[\mathrm{G•G}]_i + [\mathrm{H•G}]_i \quad \text{(S9)}$$
$$[\mathrm{H•H}]_i = K_{\mathrm{H•H}}[\mathrm{H}]_i^2 \quad \text{(S10)}$$
$$[\mathrm{G•G}]_i = K_{\mathrm{G•G}}[\mathrm{G}]_i^2 \quad \text{(S11)}$$
$$[\mathrm{H•G}]_i = K_{\mathrm{H•G}}[\mathrm{H}]_i\ [\mathrm{G}]_i \quad \text{(S12)}$$

where $[Ht]_i$ is the total concentration of host in the cell after the i-th injection
$[Gt]_i$ is the total concentration of guest in the cell after the i-th injection
$[H]_i$ is the concentration of unbound host in the cell after the i-th injection
$[G]_i$ is the concentration of unbound guest in the cell after the i-th injection
$[H•H]_i$ is the concentration of host dimer in the cell after the i-th injection
$[G•G]_i$ is the concentration of guest dimer in the cell after the i-th injection
$[H•G]_i$ is the concentration of the host•guest complex in the cell after the i-th injection

The integrated molar heat of the i-th injection (Q_i) is given by

$$Q_i = Q_0 + Q_{\mathrm{H•H}} + Q_{\mathrm{G•G}} + Q_{\mathrm{H•G}} \quad \text{(S13)}$$

where Q_0 is the baseline correction that is usually of the order 1 kJ mol^{-1}

$$Q_{\mathrm{H•H}} = 2\frac{V([\mathrm{H•H}]_i-[\mathrm{H•H}]_{i-1})+V_i[\mathrm{H•H}]_{i-1}}{V_i[\mathrm{Gt}]_s}\Delta H°_{\mathrm{H•H}} \quad \text{(S14)}$$
$$Q_{\mathrm{G•G}} = 2\frac{V([\mathrm{G•G}]_i-[\mathrm{G•G}]_{i-1})+V_i([\mathrm{G•G}]_{i-1}-[\mathrm{G•G}]_s)}{V_i[\mathrm{Gt}]_s}\Delta H°_{\mathrm{G•G}} \quad \text{(S15)}$$
$$Q_{\mathrm{H•G}} = \frac{V([\mathrm{H•G}]_i-[\mathrm{H•G}]_{i-1})+V_i[\mathrm{H•G}]_{i-1}}{V_i[\mathrm{Gt}]_s}\Delta H°_{\mathrm{H•G}} \quad \text{(S16)}$$

where V is the volume of the cell
V_i is the volume of the i-th injection

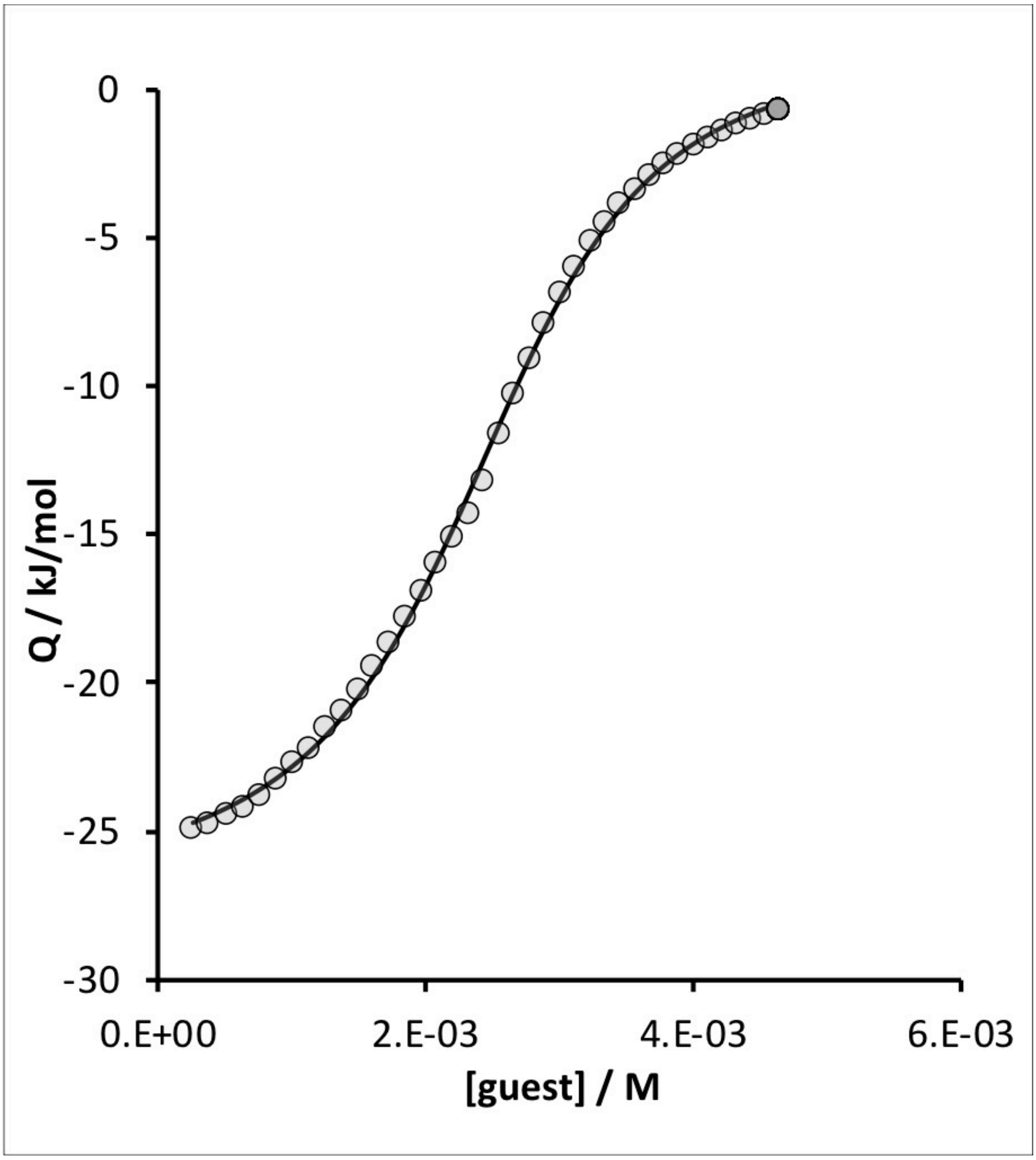

Figure S87. ITC titration of **AA** (26 mM in the syringe) into **DD** (2.6 mM in the cell) in toluene at 298 K. The line of best fit to a 1:1 isotherm corresponds to K = 4,200 M^{-1} and $\Delta H°$ = -27 kJ mol^{-1}.

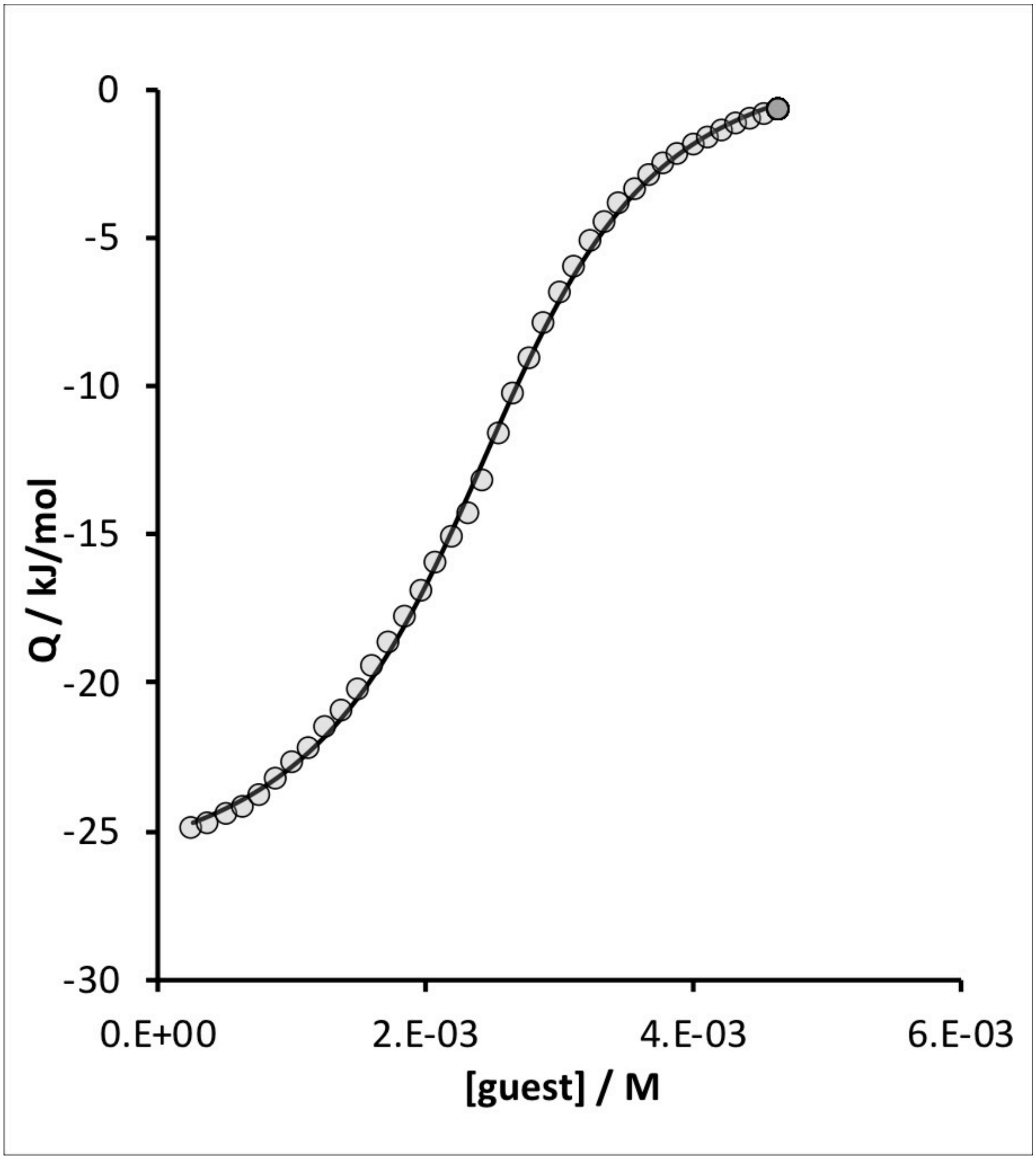

Figure S88. ITC titration of **DD** (26 mM in the syringe) into **AA** (2.6 mM in the cell) in toluene at 298 K. The line of best fit to a 1:1 isotherm corresponds to K = 4,800 M^{-1} and $\Delta H°$ = -31 kJ mol^{-1}.

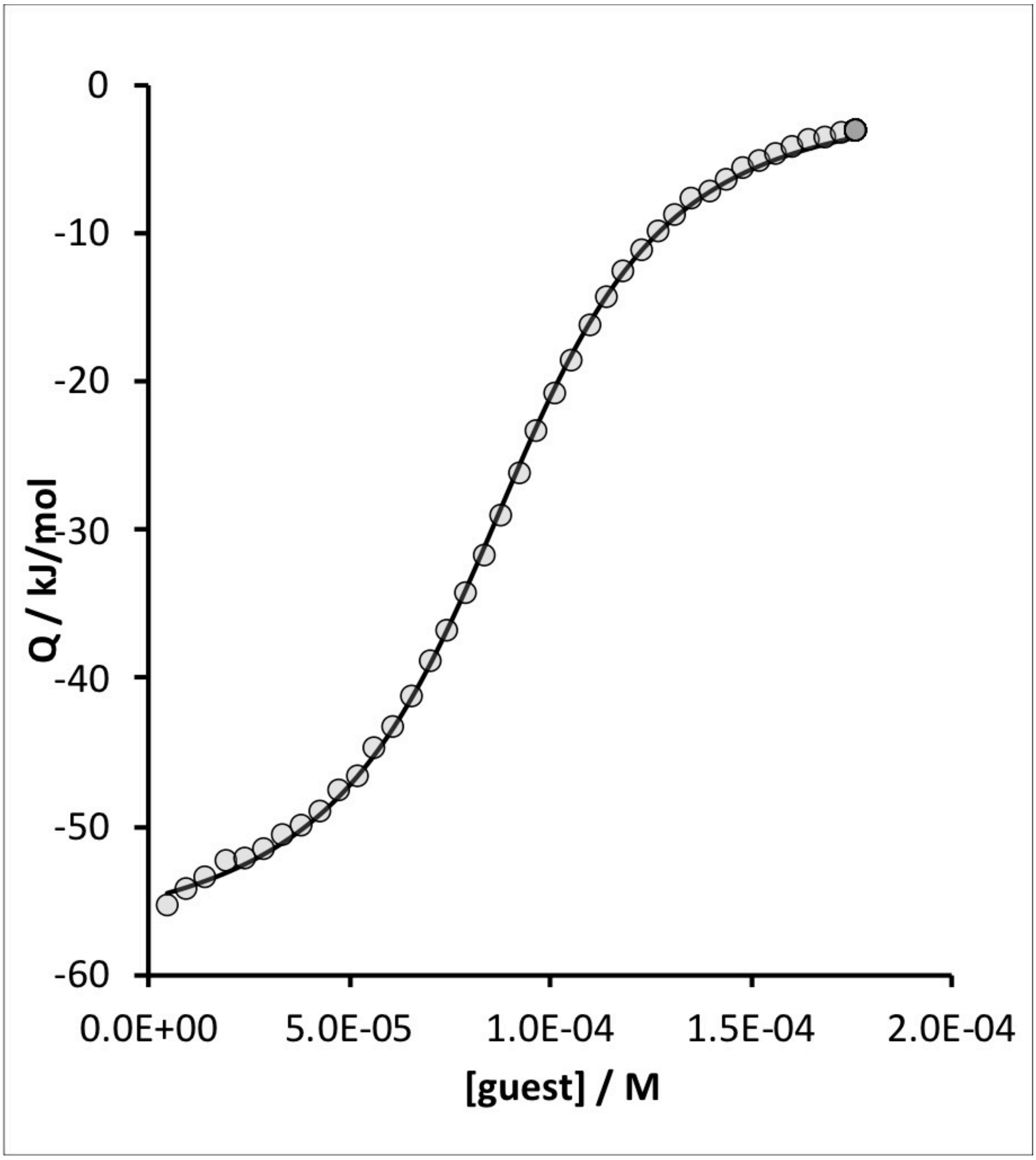

Figure S89. ITC titration of **AAA** (0.99 mM in the syringe) into **DDD** (0.10 mM in the cell) in toluene at 298 K. The line of best fit to a 1:1 isotherm corresponds to K = 150,000 M^{-1} and $\Delta H°$ = -57 kJ mol^{-1}.

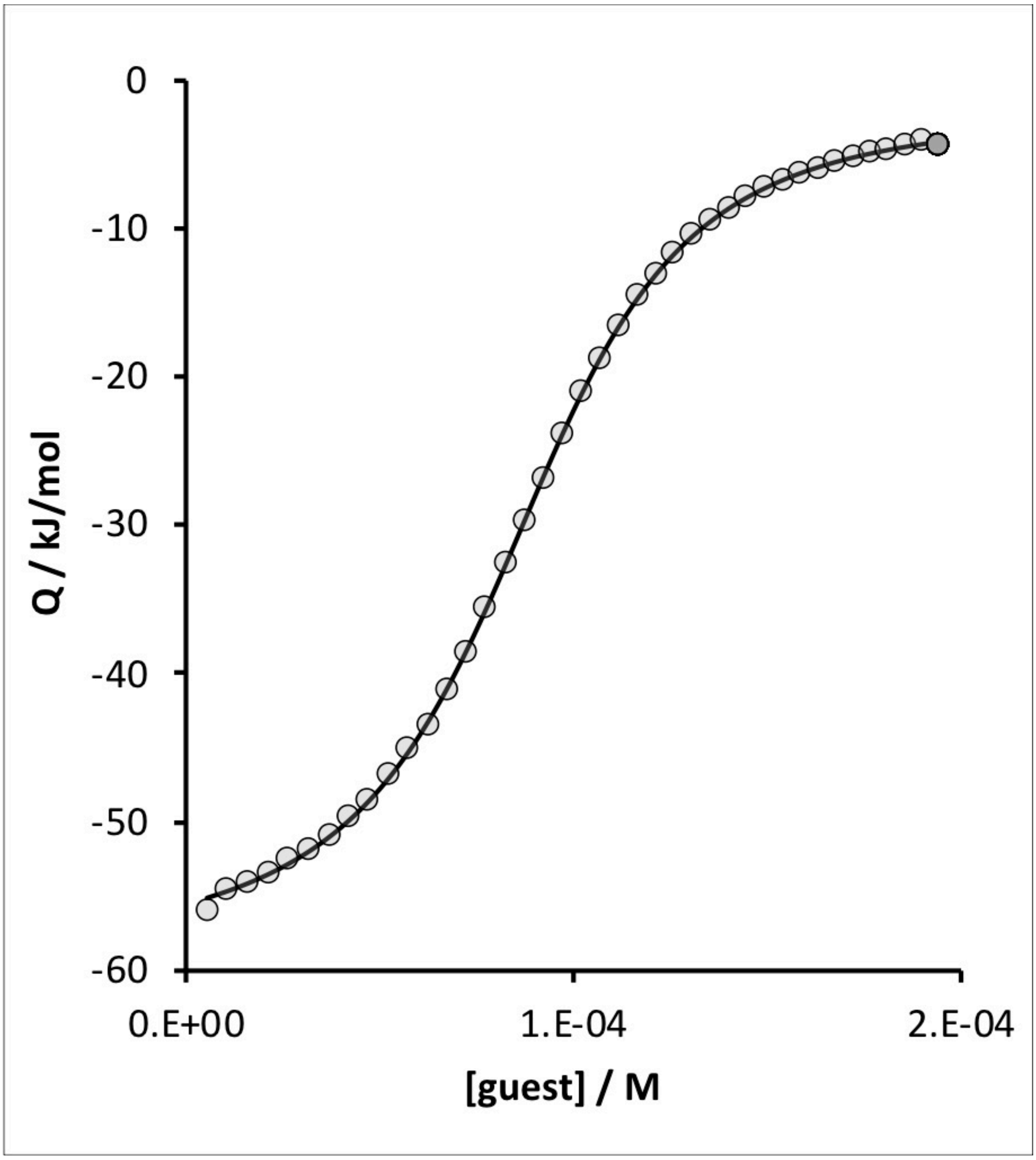

Figure S90. ITC titration of **DDD** (1.1 mM in the syringe) into **AAA** (0.10 mM in the cell) in toluene at 298 K. The line of best fit to a 1:1 isotherm corresponds to K = 140,000 M^{-1} and $\Delta H°$ = -57 kJ mol^{-1}.

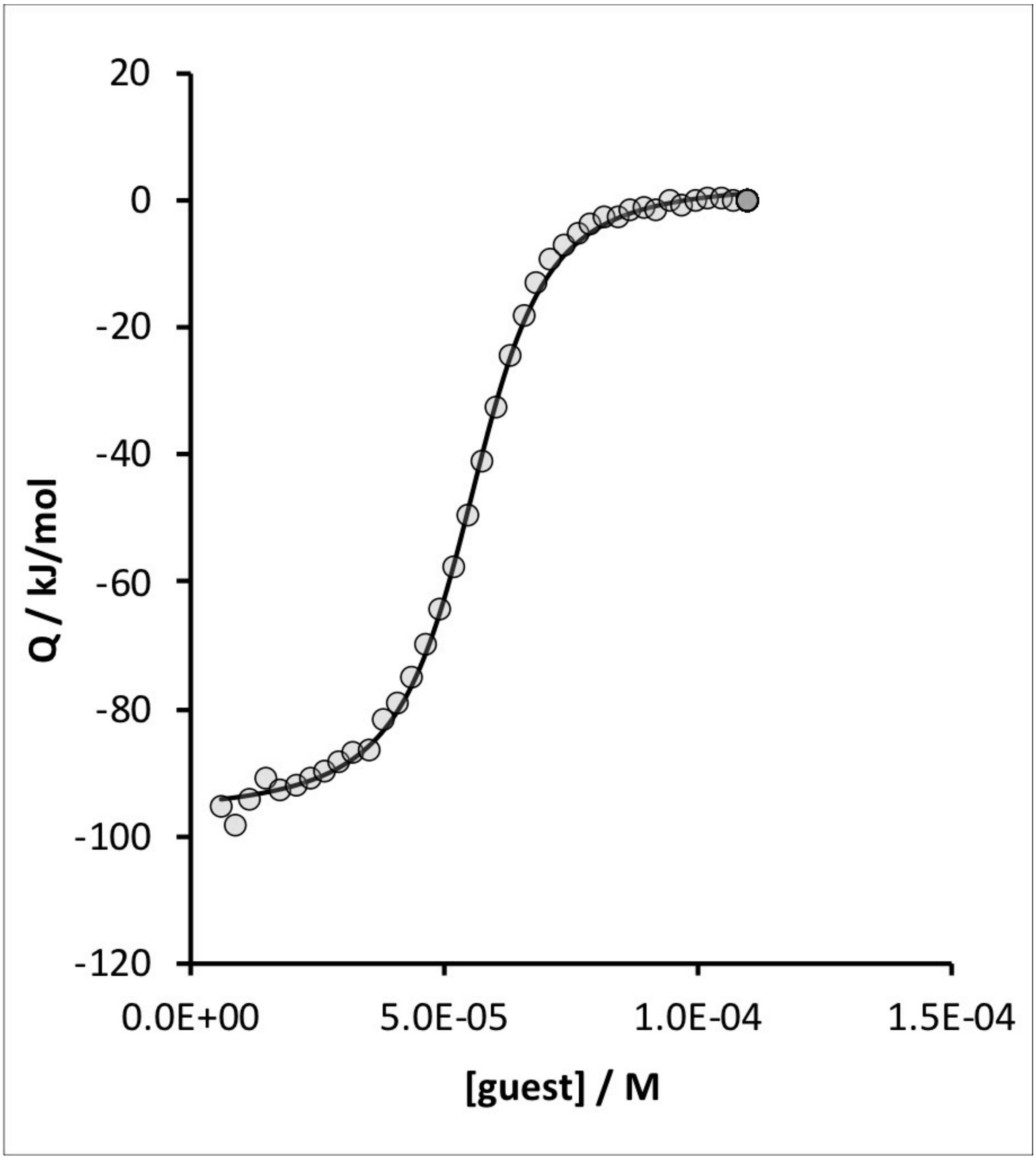

Figure S91. ITC titration of **AAAA** (0.62 mM in the syringe) into **DDDD** (0.060 mM in the cell) in toluene at 298 K. The line of best fit to a 1:1 isotherm corresponds to K = 750,000 M^{-1} and $\Delta H°$ = -100 kJ mol^{-1}.

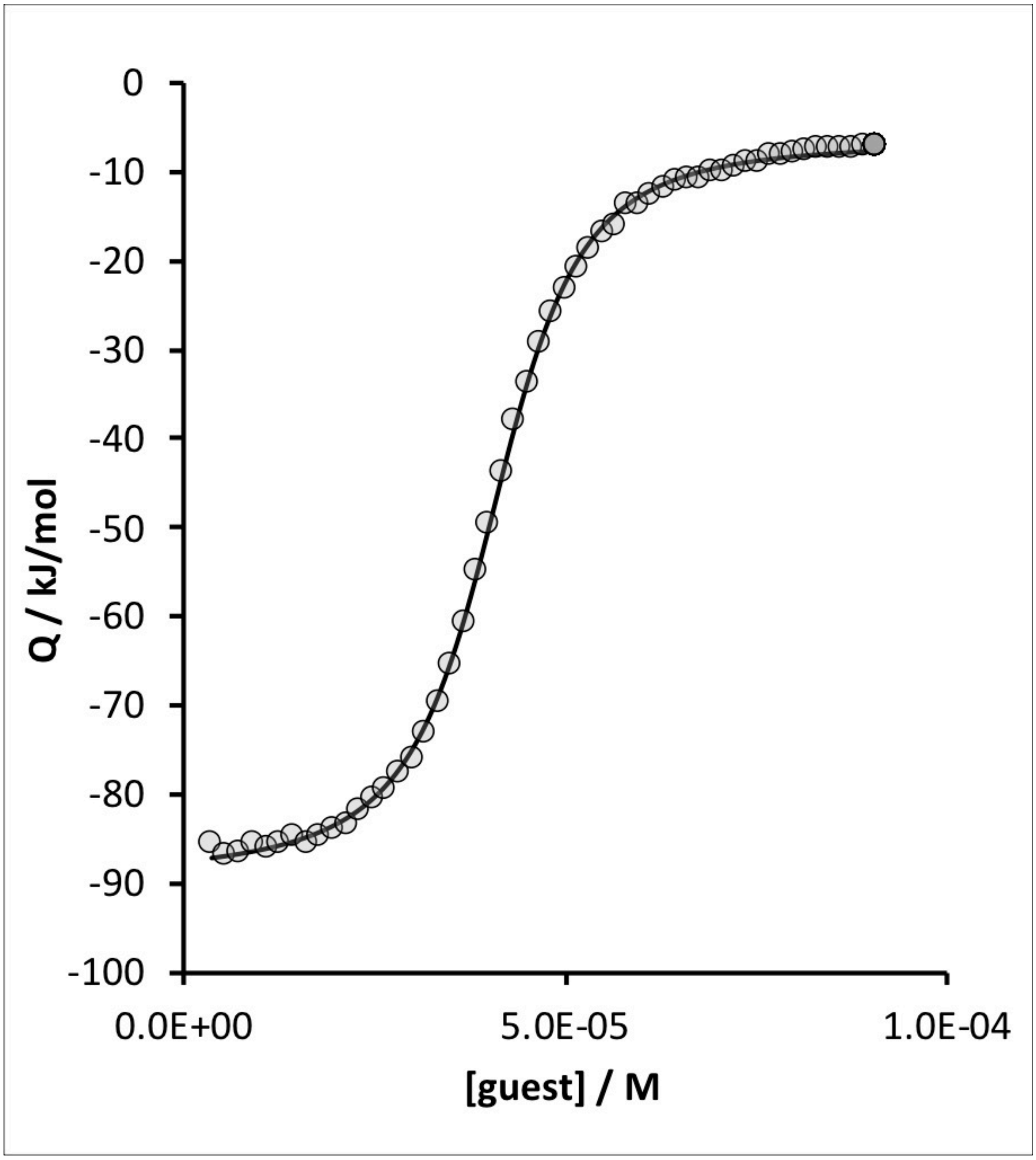

Figure S92. ITC titration of **DDDD** (0.52 mM in the syringe) into **AAAA** (0.045 mM in the cell) in toluene at 298 K. The line of best fit to a 1:1 isotherm corresponds to K = 930,000 M^{-1} and $\Delta H°$ = -83 kJ mol^{-1}.

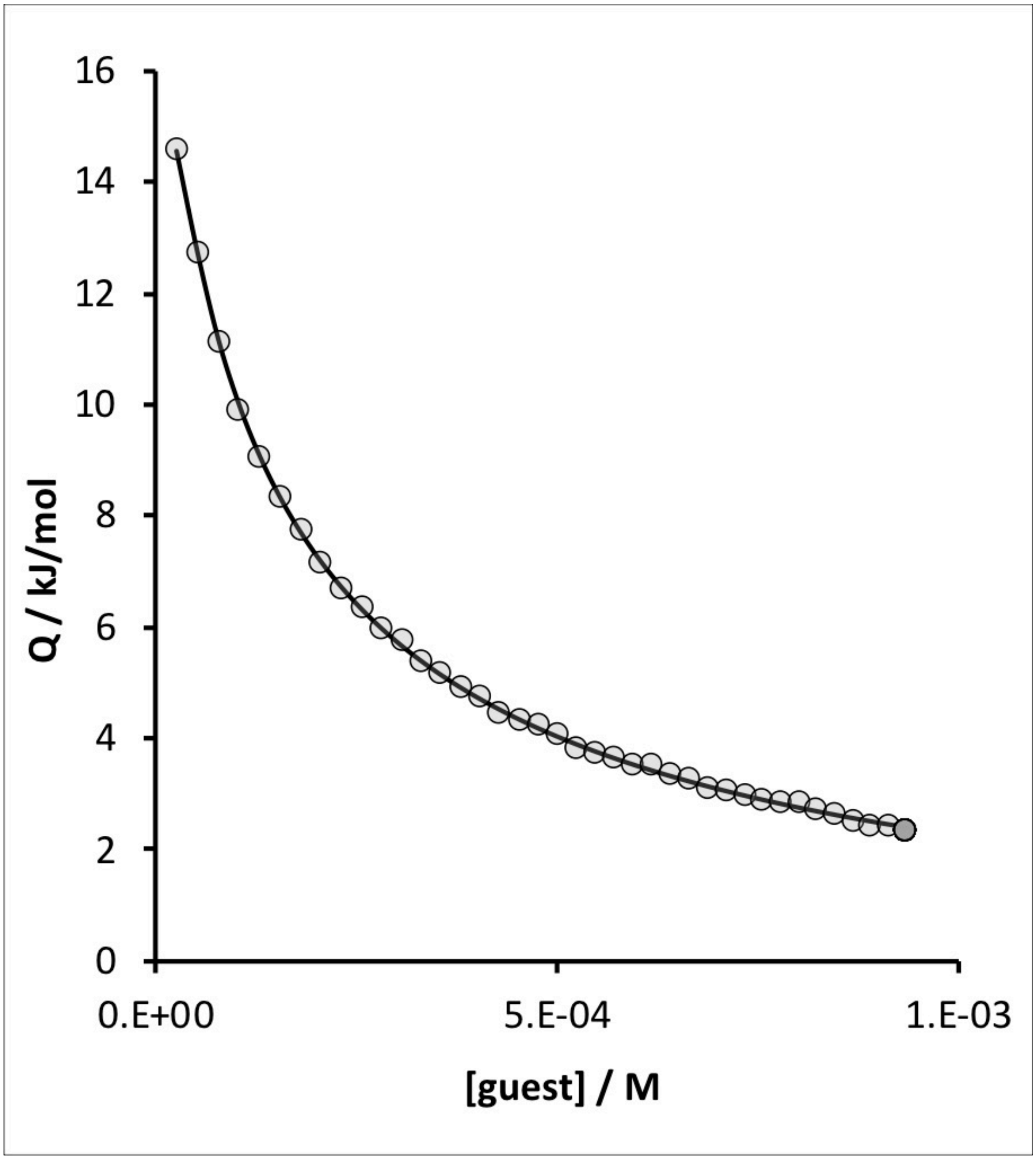

Figure S93. ITC dilution of **AD** (5.4 mM in the syringe) into toluene at 298 K. The line of best fit to a dimerisation isotherm corresponds to K = 1,600 M^{-1} and $\Delta H°$ = -40 kJ mol^{-1}.

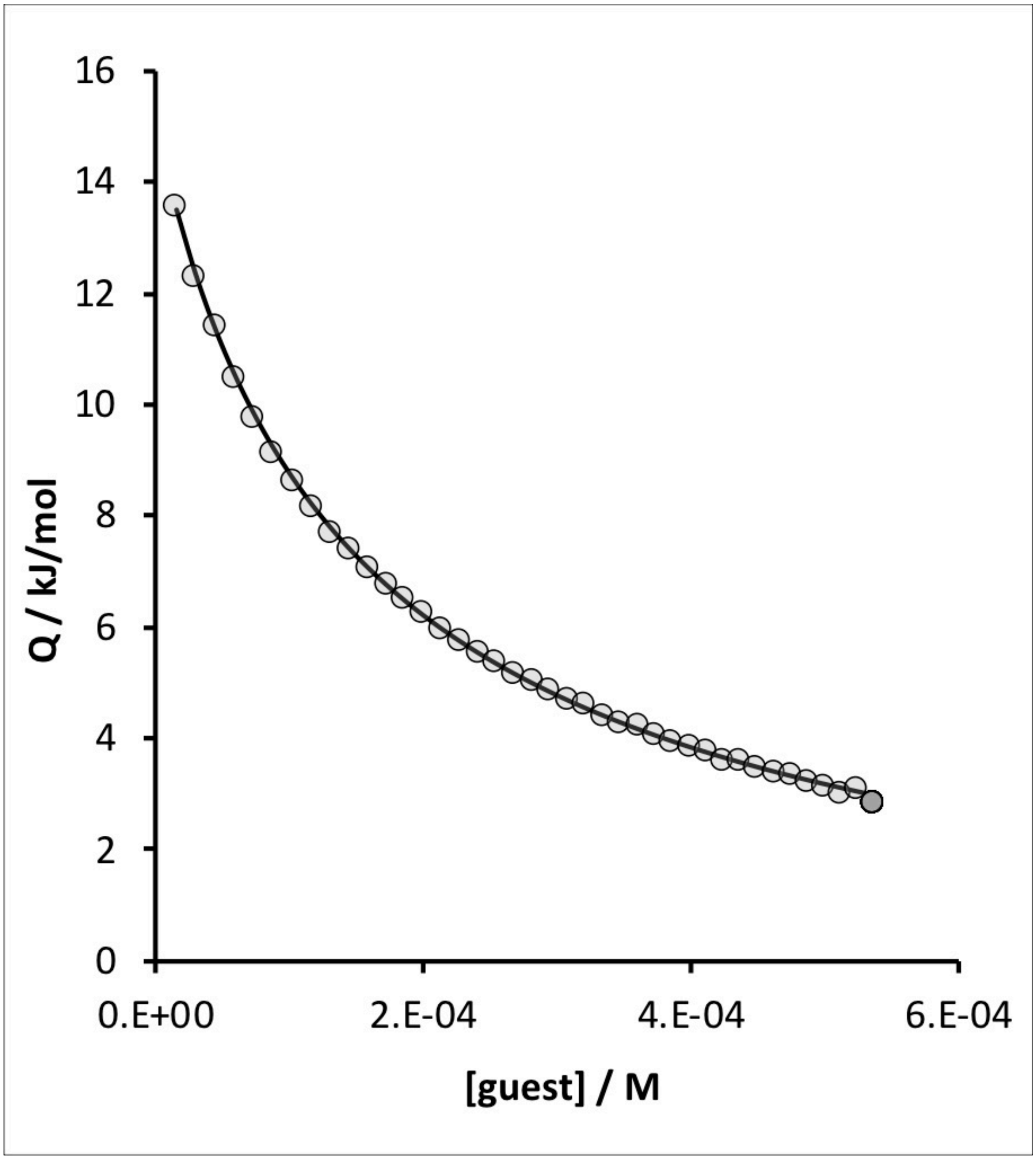

Figure S94. ITC dilution of **AAD** (3 mM in the syringe) into toluene at 298 K. The line of best fit to a dimerisation isotherm corresponds to K = 1,300 M^{-1} and $\Delta H°$ = -39 kJ mol^{-1}.

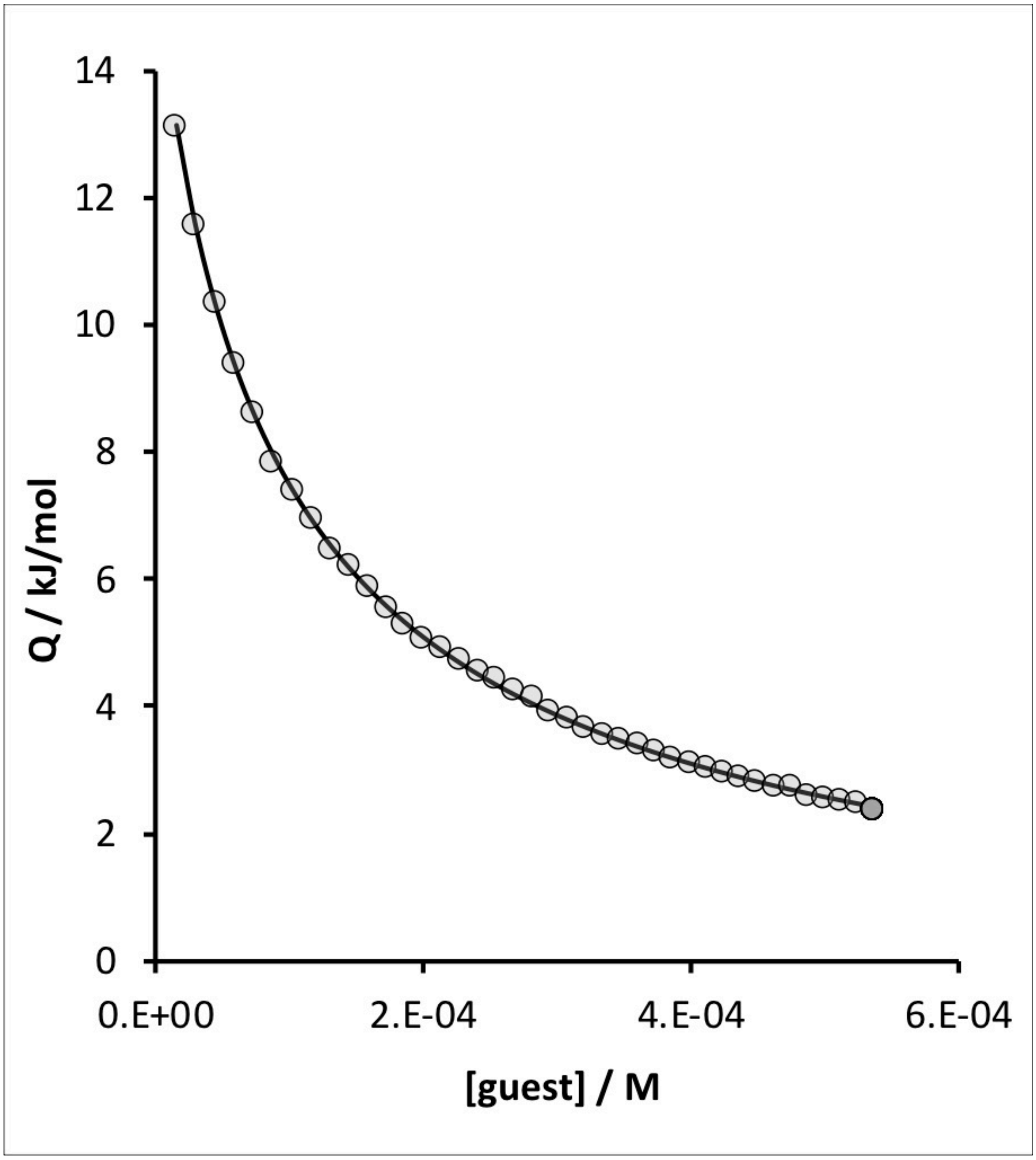

Figure S95. ITC dilution of **ADD** (3 mM in the syringe) into toluene at 298 K. The line of best fit to a dimerisation isotherm corresponds to K = 2,100 M^{-1} and $\Delta H°$ = -36 kJ mol^{-1}.

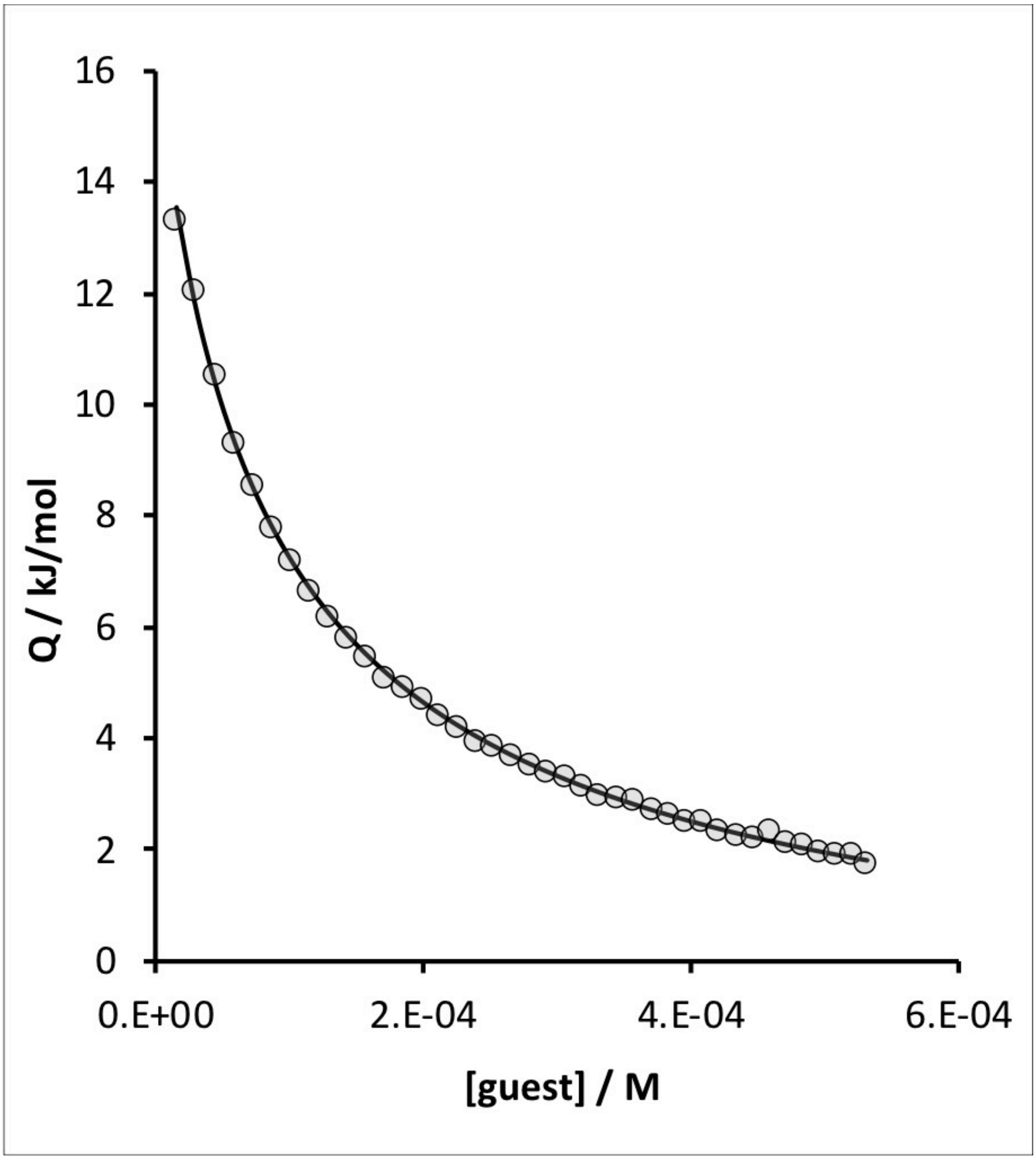

Figure S96. ITC dilution of **DAD** (3 mM in the syringe) into toluene at 298 K. The line of best fit to a dimerisation isotherm corresponds to K = 2,300 M^{-1} and $\Delta H°$ = -39 kJ mol^{-1}.

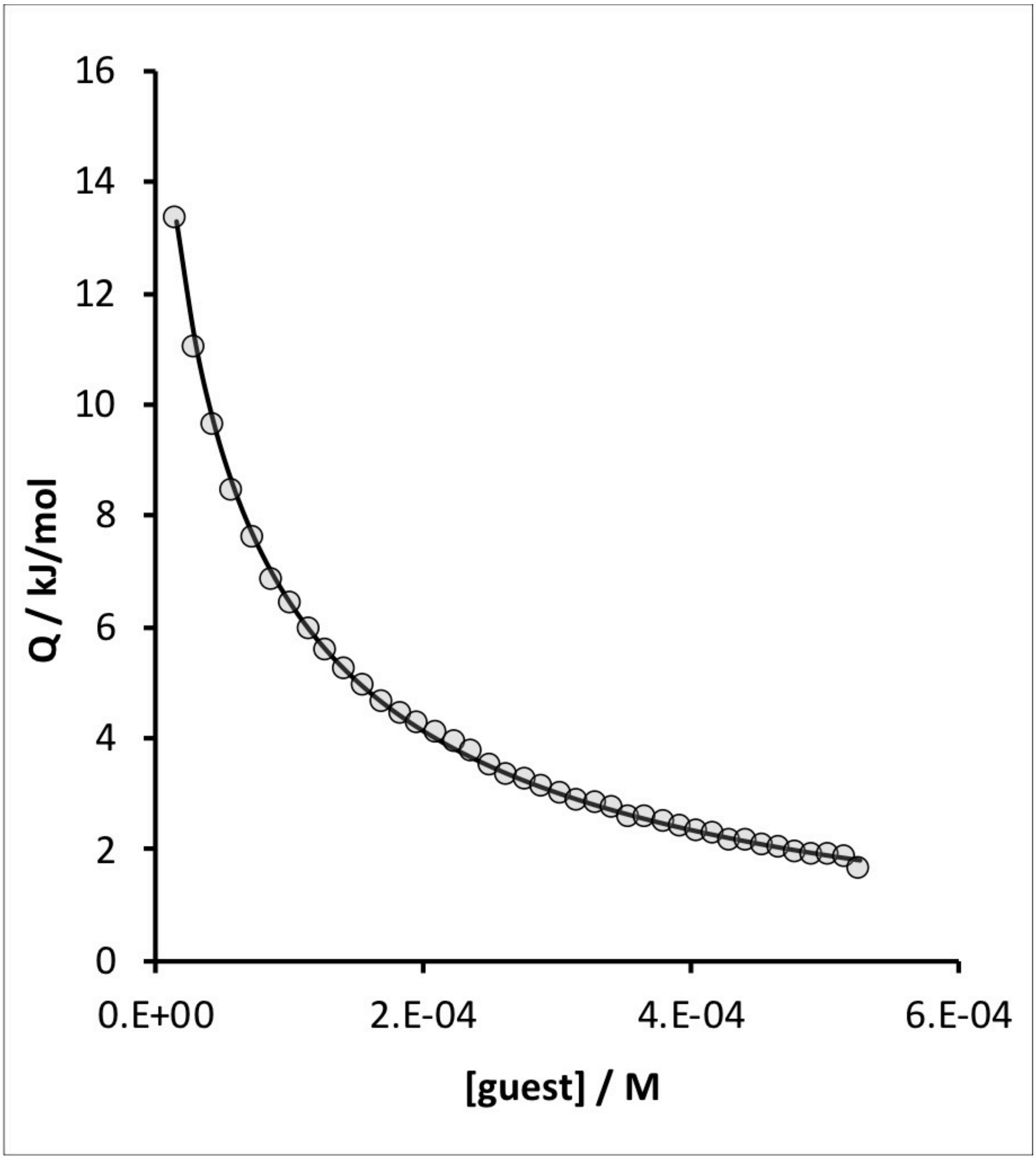

Figure S97. ITC dilution of **ADA** (3 mM in the syringe) into toluene at 298 K. The line of best fit to a dimerisation isotherm corresponds to K = 3,400 M^{-1} and $\Delta H°$ = -36 kJ mol^{-1}.

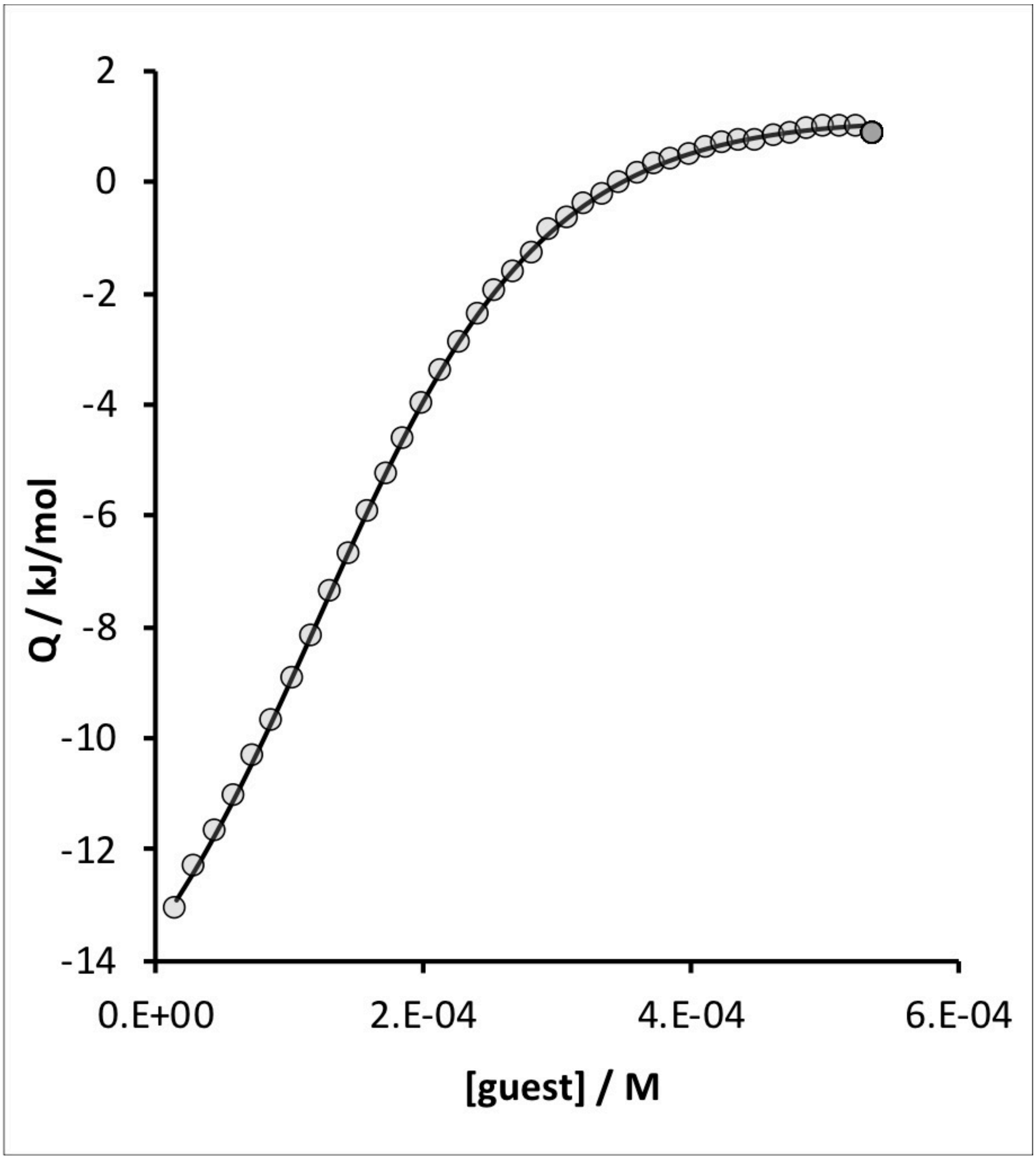

Figure S98. ITC titration of **ADD** (3.0 mM in the syringe) into **AAD** (0.30 mM in the cell) in toluene at 298 K. The line of best fit to an isotherm that allows for formation of the 1:1 complex as well as dimerization of both host and guest corresponds to K = 17,000 M^{-1} and $\Delta H°$ = -46 kJ mol^{-1}.

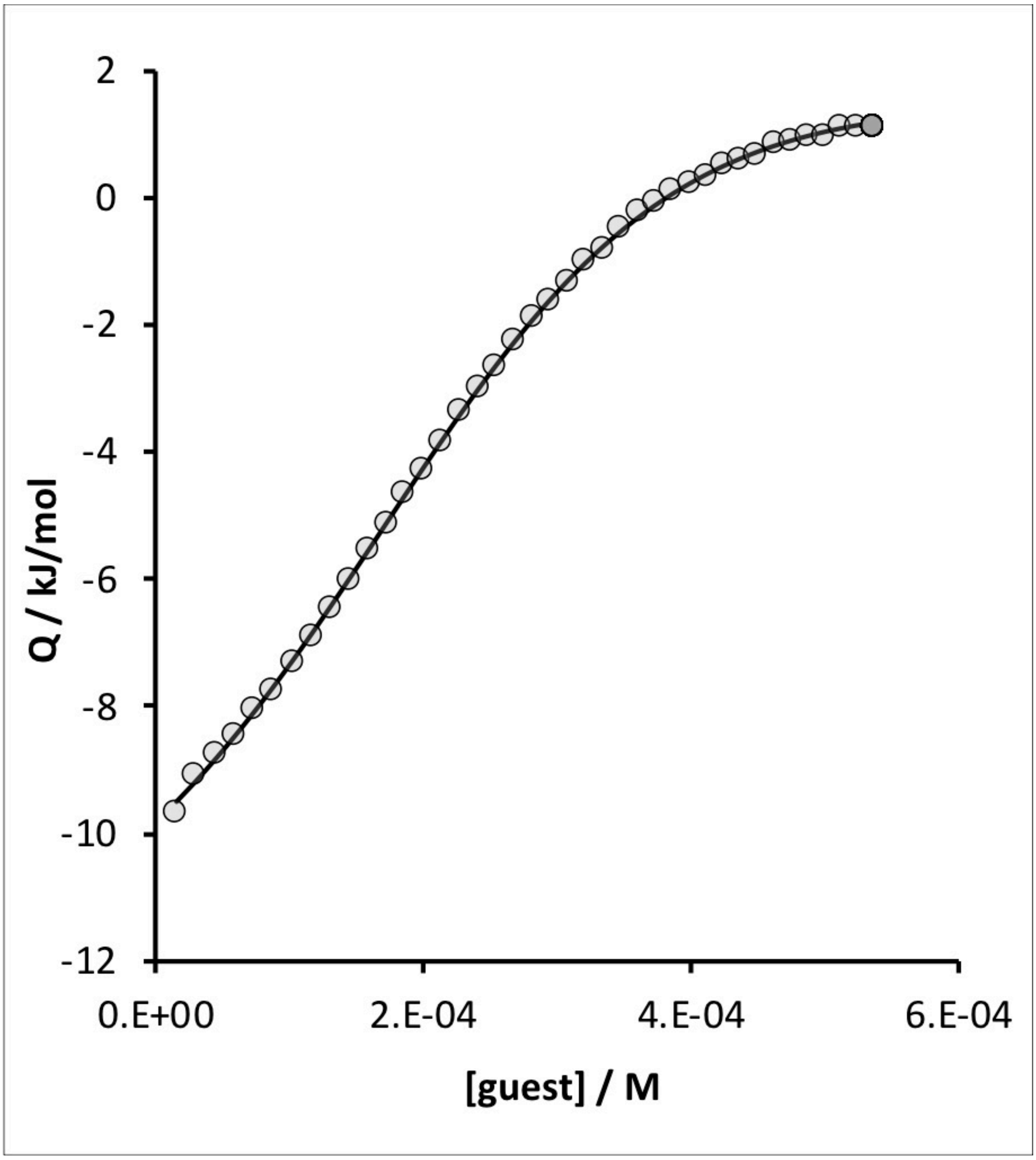

Figure S99. ITC titration of **AAD** (3.0 mM in the syringe) into **ADD** (0.30 mM in the cell) in toluene at 298 K. The line of best fit to an isotherm that allows for formation of the 1:1 complex as well as dimerization of both host and guest corresponds to K = 14,000 M^{-1} and $\Delta H°$ = -44 kJ mol^{-1}.

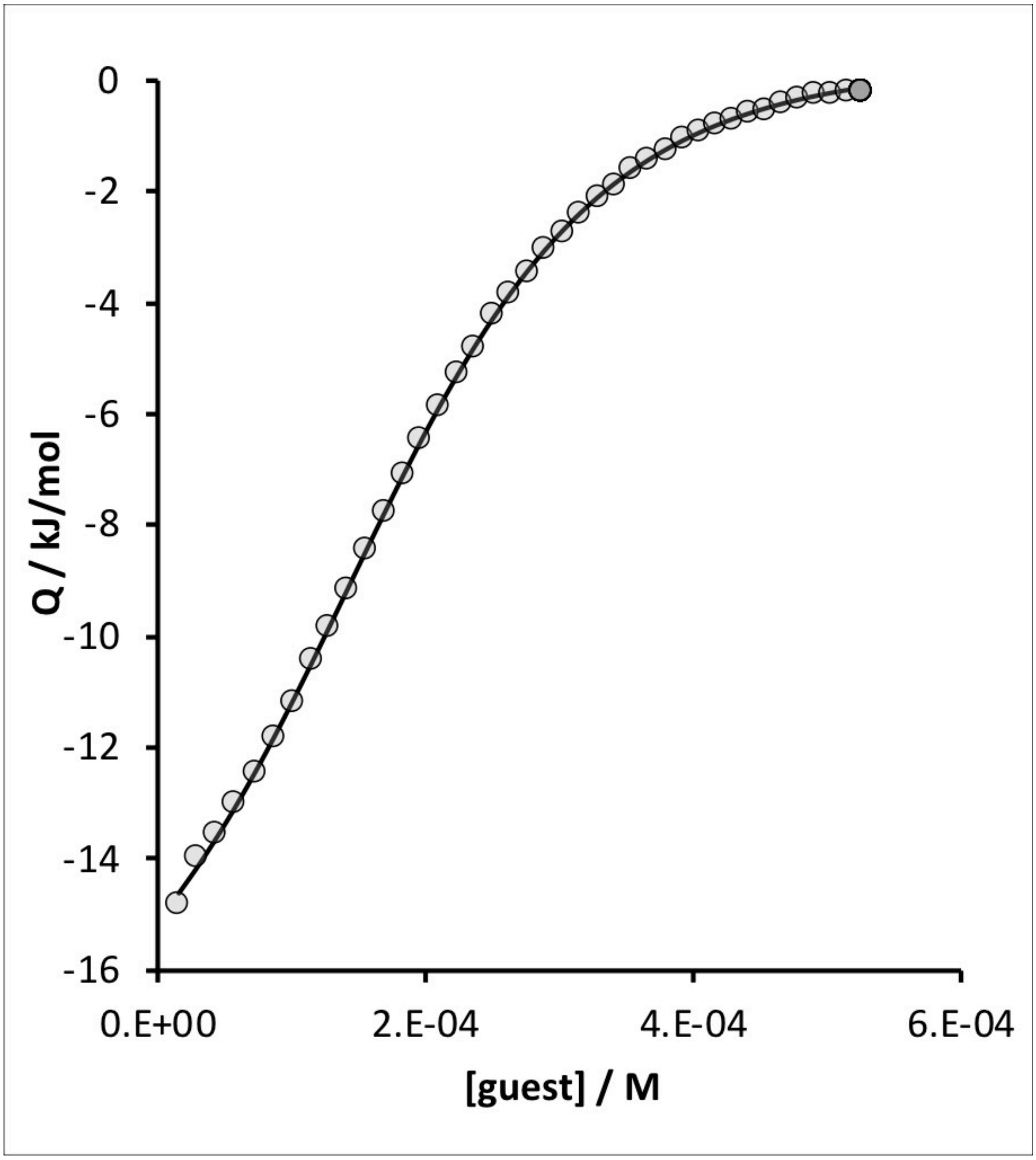

Figure S100. ITC titration of **ADA** (3.0 mM in the syringe) into **DAD** (0.29 mM in the cell) in toluene at 298 K. The line of best fit to an isotherm that allows for formation of the 1:1 complex as well as dimerization of both host and guest corresponds to *K* = 21,000 M^{-1} and $\Delta H°$ = -50 kJ mol^{-1}.

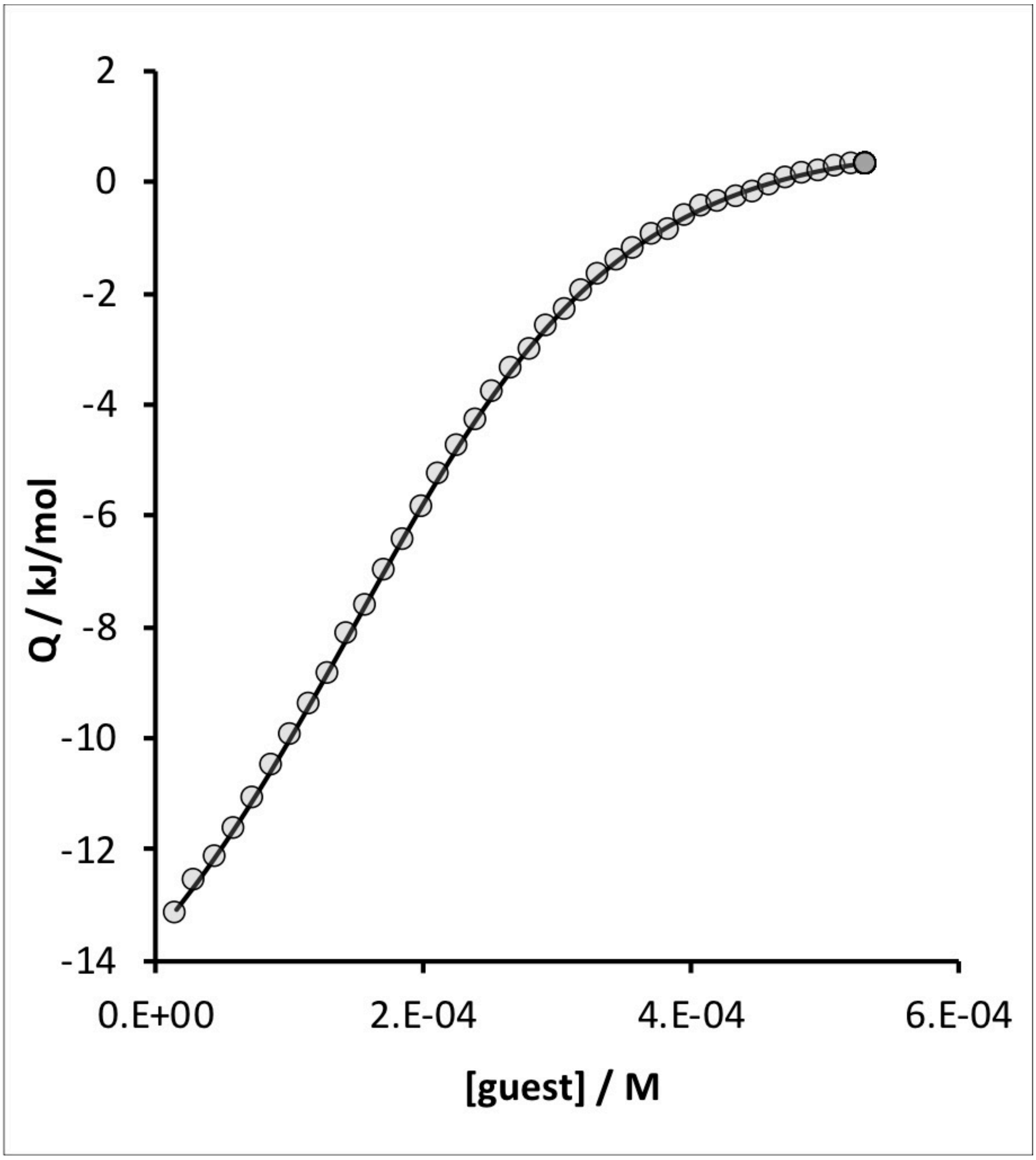

Figure S101. ITC titration of **DAD** (3.0 mM in the syringe) into **ADA** (0.27 mM in the cell) in toluene at 298 K. The line of best fit to an isotherm that allows for formation of the 1:1 complex as well as dimerization of both host and guest corresponds to K = 20,000 M^{-1} and $\Delta H°$ = -50 kJ mol^{-1}.

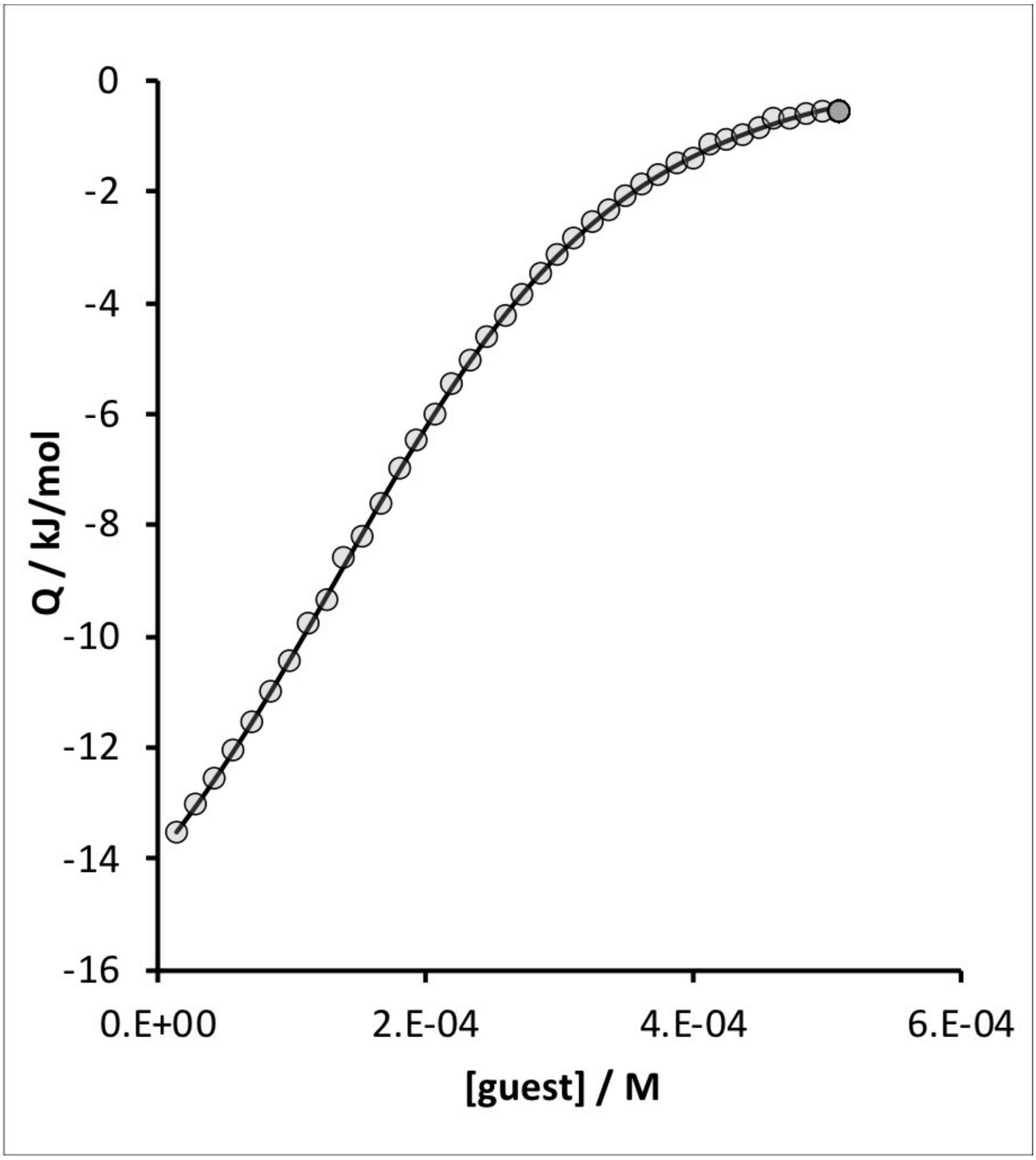

Figure S102. ITC titration of **ADA** (2.9 mM in the syringe) into **ADD** (0.30 mM in the cell) in toluene at 298 K. The line of best fit to an isotherm that allows for formation of the 1:1 complex as well as dimerization of both host and guest corresponds to K = 18,000 M^{-1} and $\Delta H°$ = -48 kJ mol^{-1}.

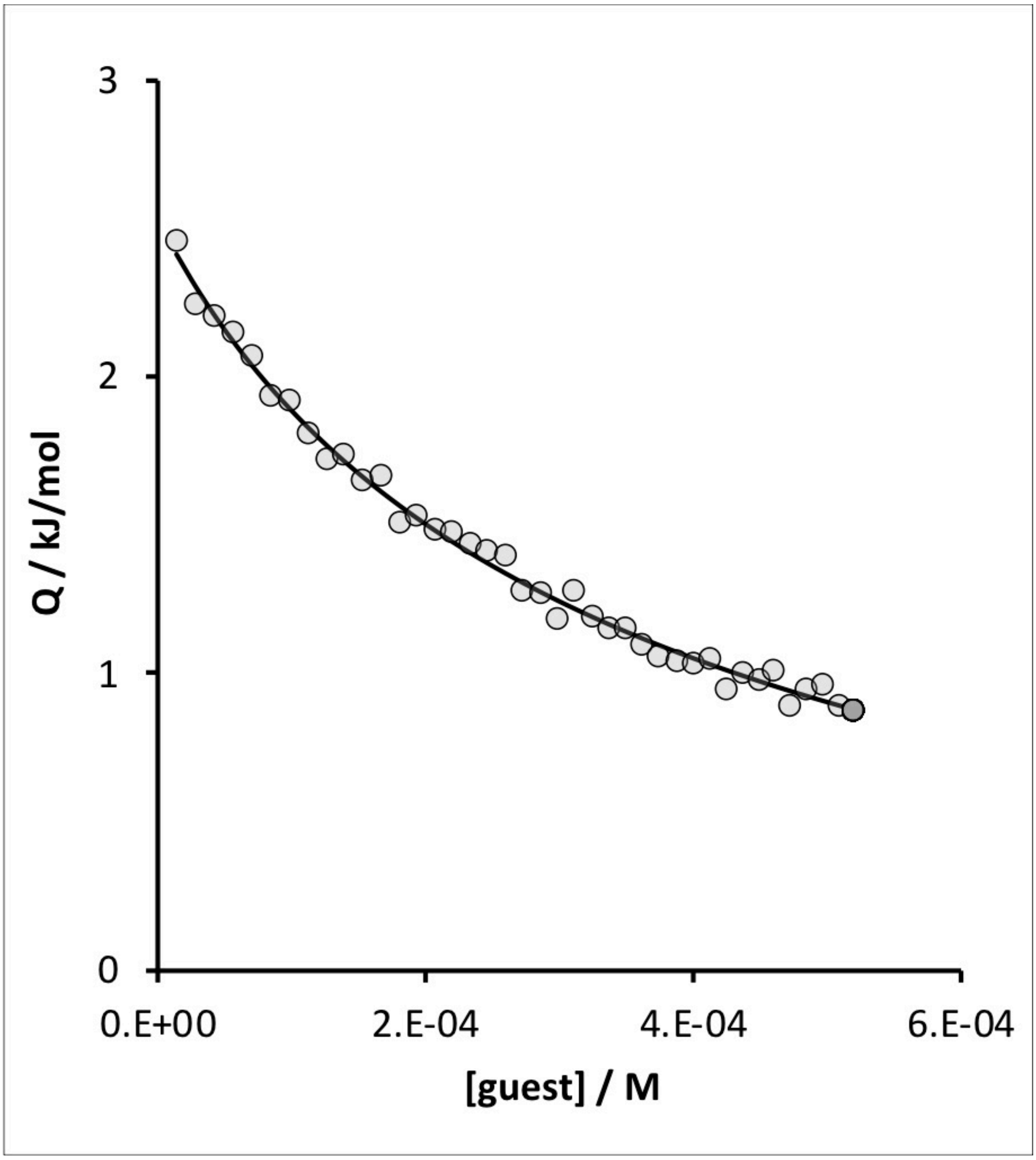

Figure S103. ITC titration of **ADA** (2.9 mM in the syringe) into **AAD** (0.30 mM in the cell) in toluene at 298 K. The line of best fit to an isotherm that allows for formation of the 1:1 complex as well as dimerization of both host and guest corresponds to K = 3,900 M^{-1} and $\Delta H°$ = -38 kJ mol^{-1}.

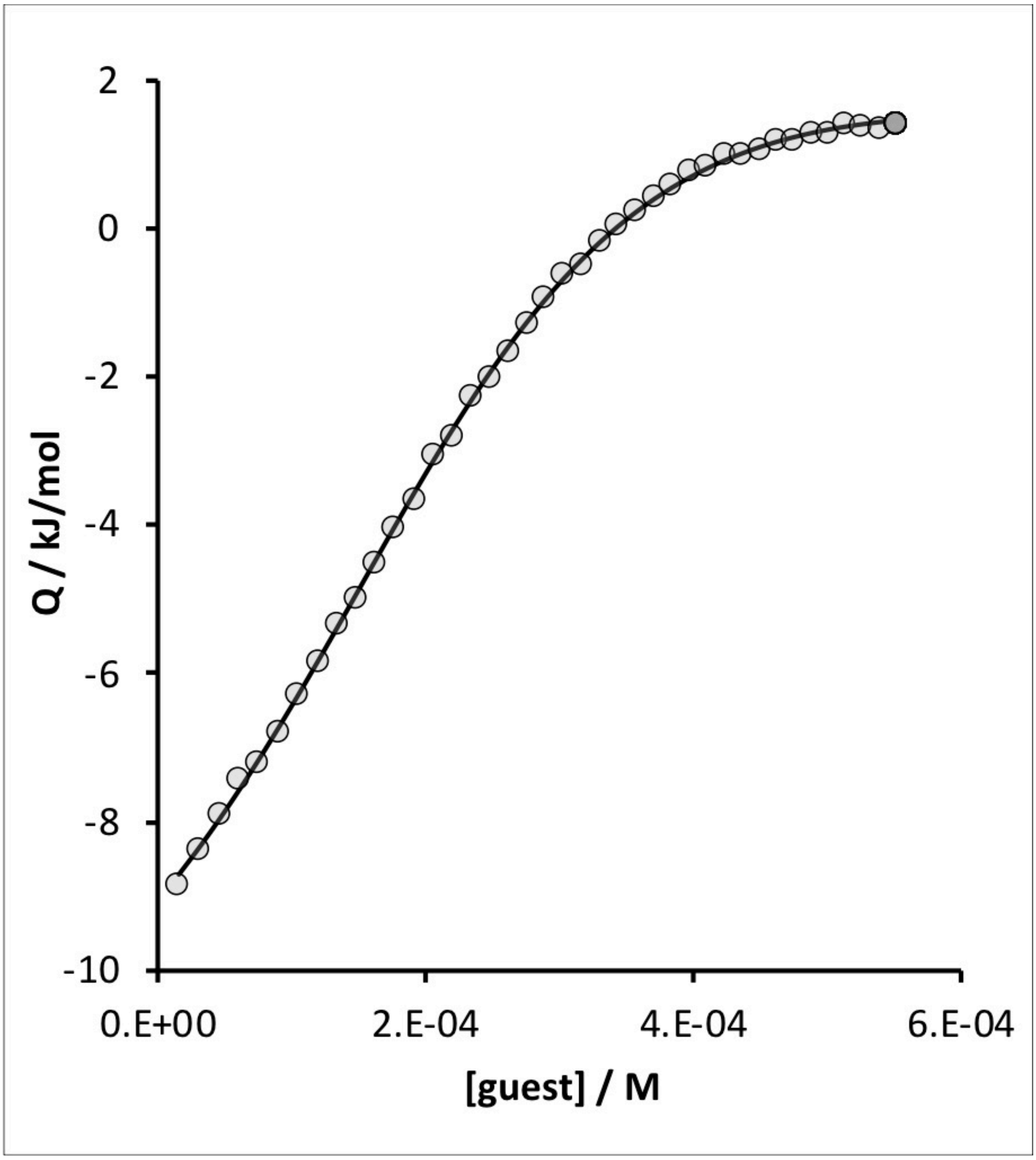

Figure S104. ITC titration of **AAD** (3.1 mM in the syringe) into **DAD** (0.31 mM in the cell) in toluene at 298 K. The line of best fit to an isotherm that allows for formation of the 1:1 complex as well as dimerization of both host and guest corresponds to K = 14,000 M^{-1} and $\Delta H°$ = -45 kJ mol^{-1}.

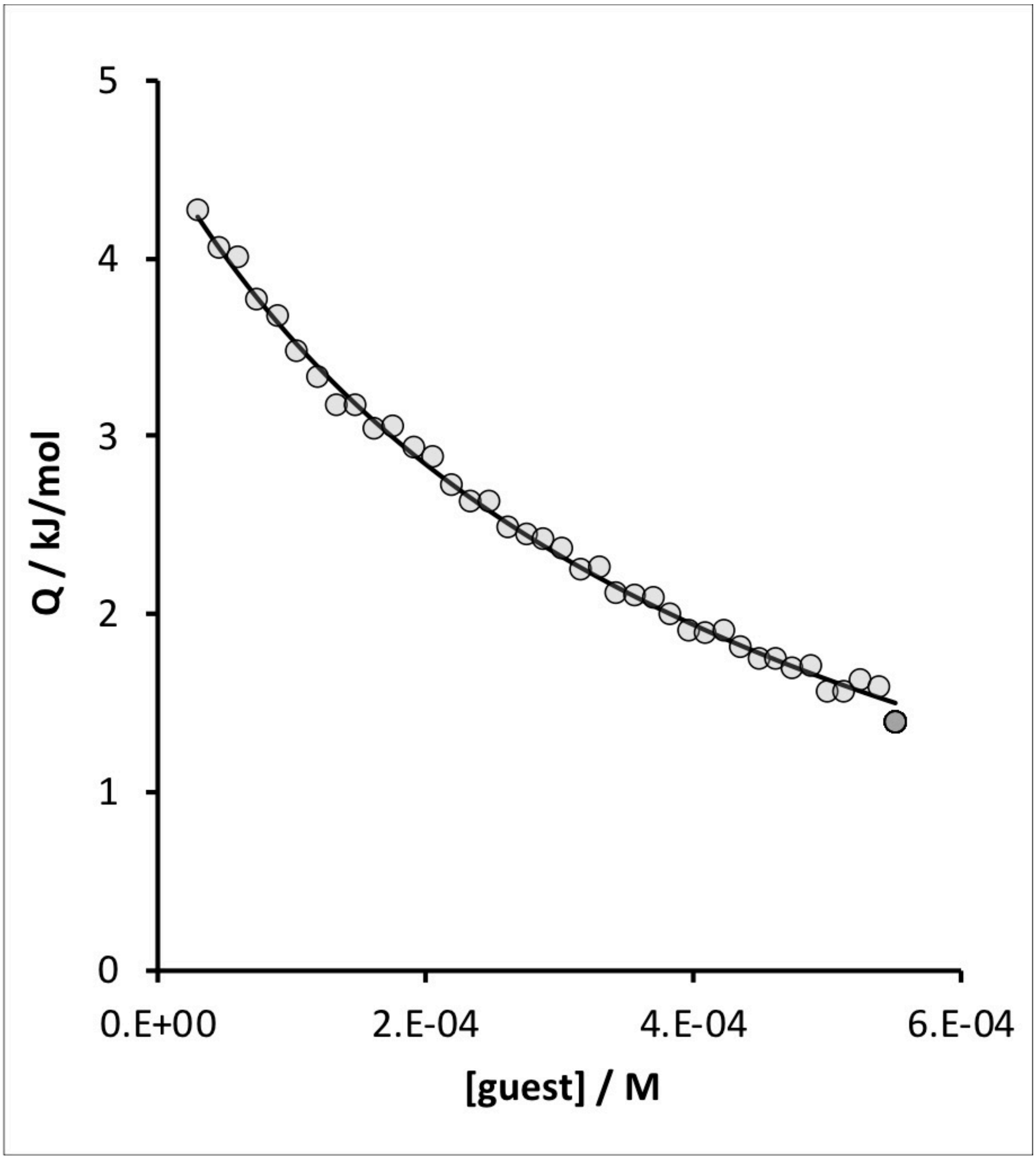

Figure S105. ITC titration of **ADD** (3.1 mM in the syringe) into **DAD** (0.31 mM in the cell) in toluene at 298 K. The line of best fit to an isotherm that allows for formation of the 1:1 complex as well as dimerization of both host and guest corresponds to *K* = 2,900 M^{-1} and $\Delta H°$ = -34 kJ mol^{-1}.

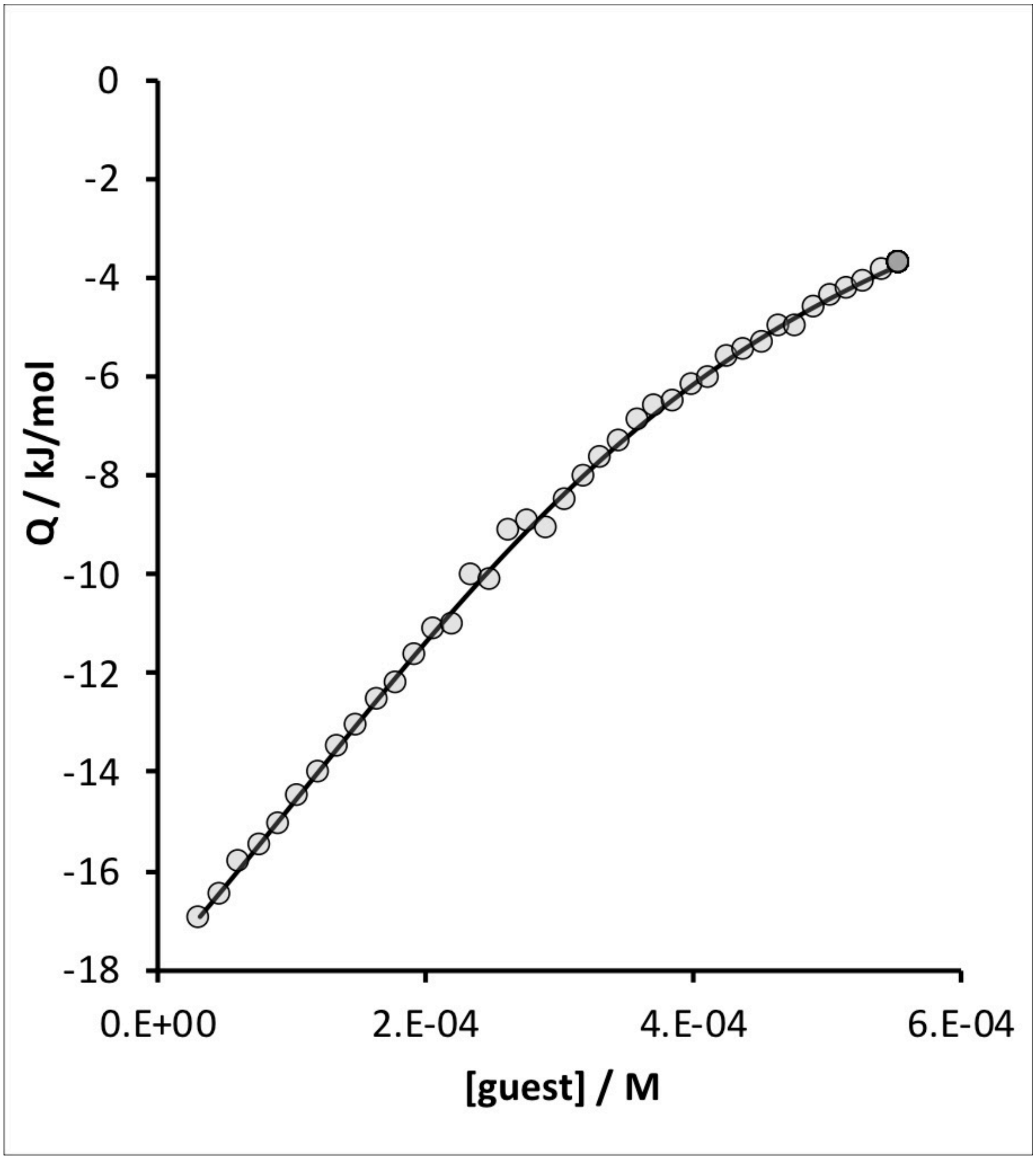

Figure S106. ITC titration of **DDA** (3.1 mM in the syringe) into **AAA** (0.64 mM in the cell) in toluene at 298 K. The line of best fit to an isotherm that allows for formation of the 1:1 complex as well as dimerization of the guest corresponds to K = 4,100 M^{-1} and $\Delta H°$ = -42 kJ mol^{-1}.

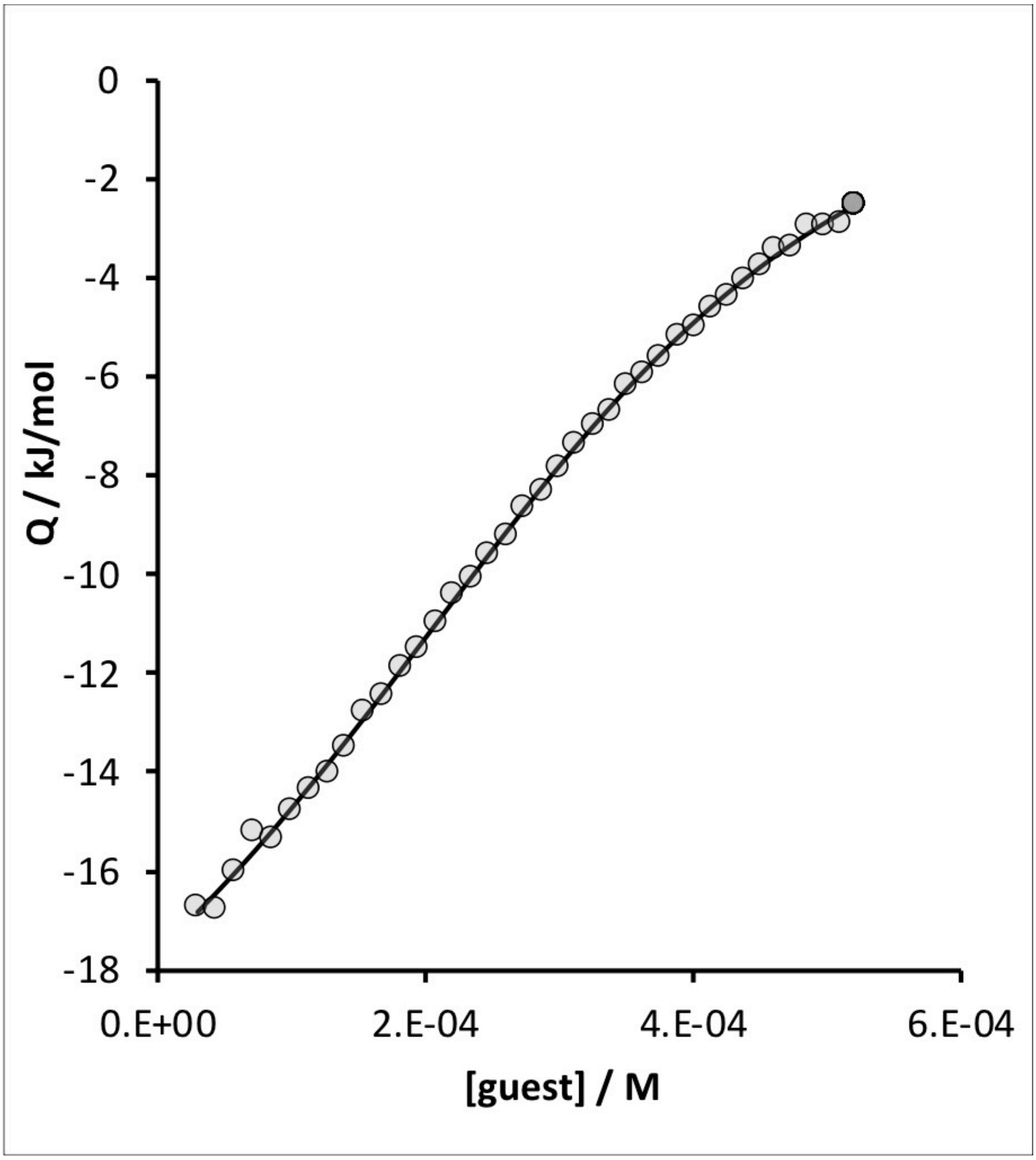

Figure S107. ITC titration of **DAD** (2.9 mM in the syringe) into **AAA** (0.64 mM in the cell) in toluene at 298 K. The line of best fit to an isotherm that allows for formation of the 1:1 complex as well as dimerization of the guest corresponds to K = 7,000 M^{-1} and $\Delta H°$ = -40 kJ mol^{-1}.

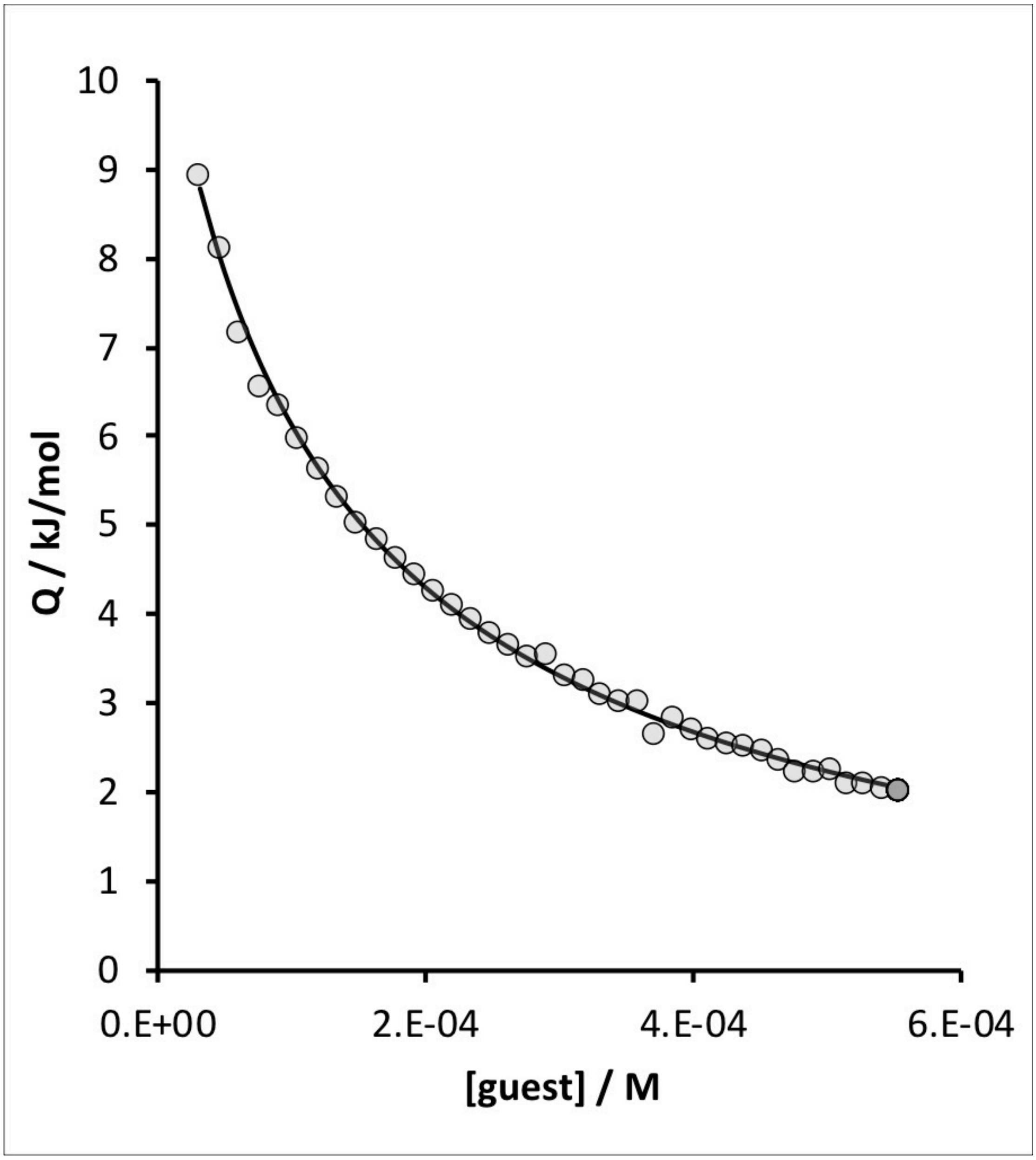

Figure S108. ITC titration of **AAD** (3.1 mM in the syringe) into **AAA** (0.64 mM in the cell) in toluene at 298 K. The line of best fit to an isotherm that allows for dimerization of the guest with a self-association constant of 1,500 M^{-1} and $\Delta H°$ = -27 kJ mol^{-1}.

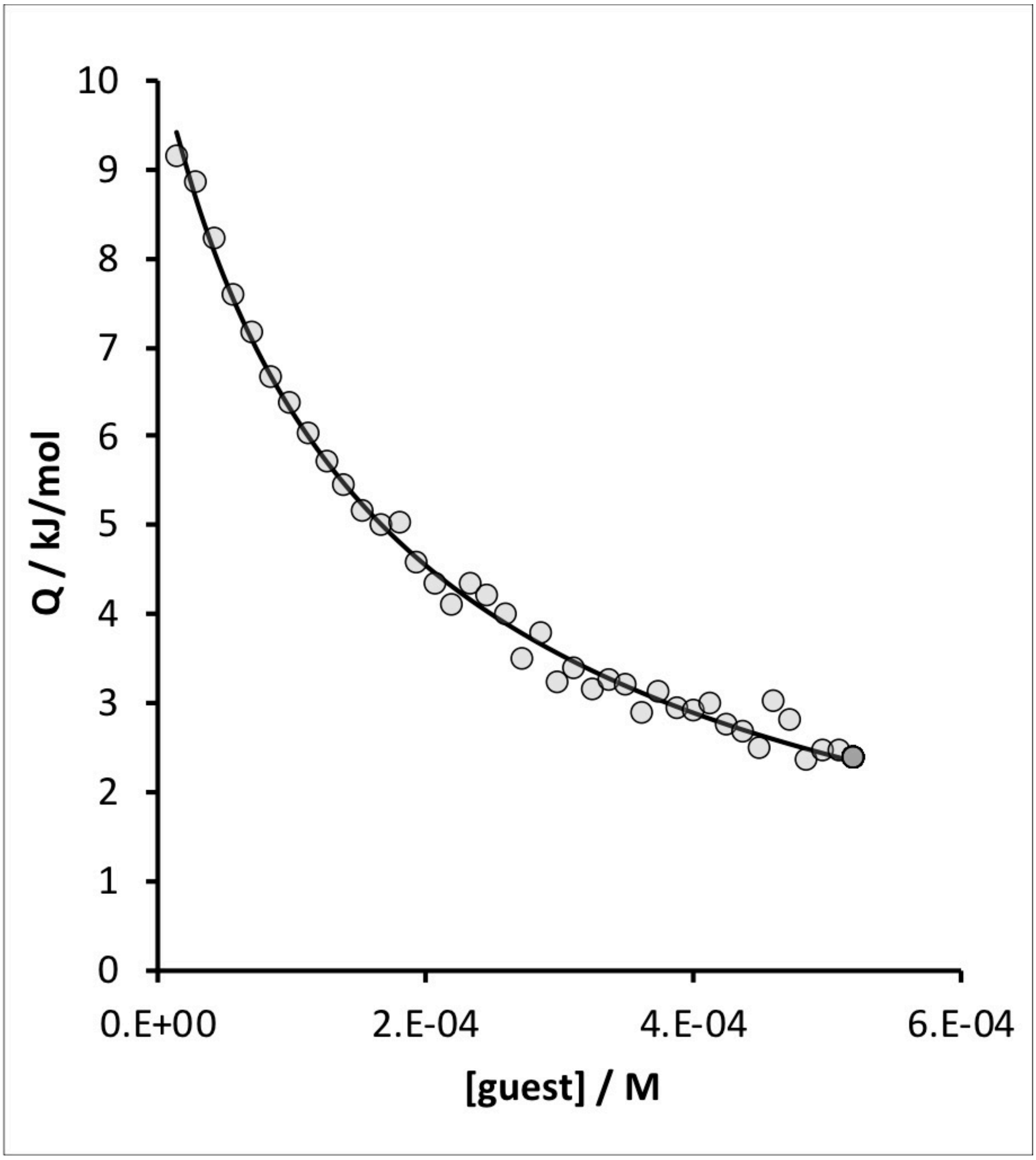

Figure S109. ITC titration of **ADA** (2.9 mM in the syringe) into **AAA** (0.64 mM in the cell) in toluene at 298 K. The line of best fit to an isotherm that allows for dimerization of the guest with a self-association constant of 1,100 M^{-1} and $\Delta H°$ = -27 kJ mol^{-1}.

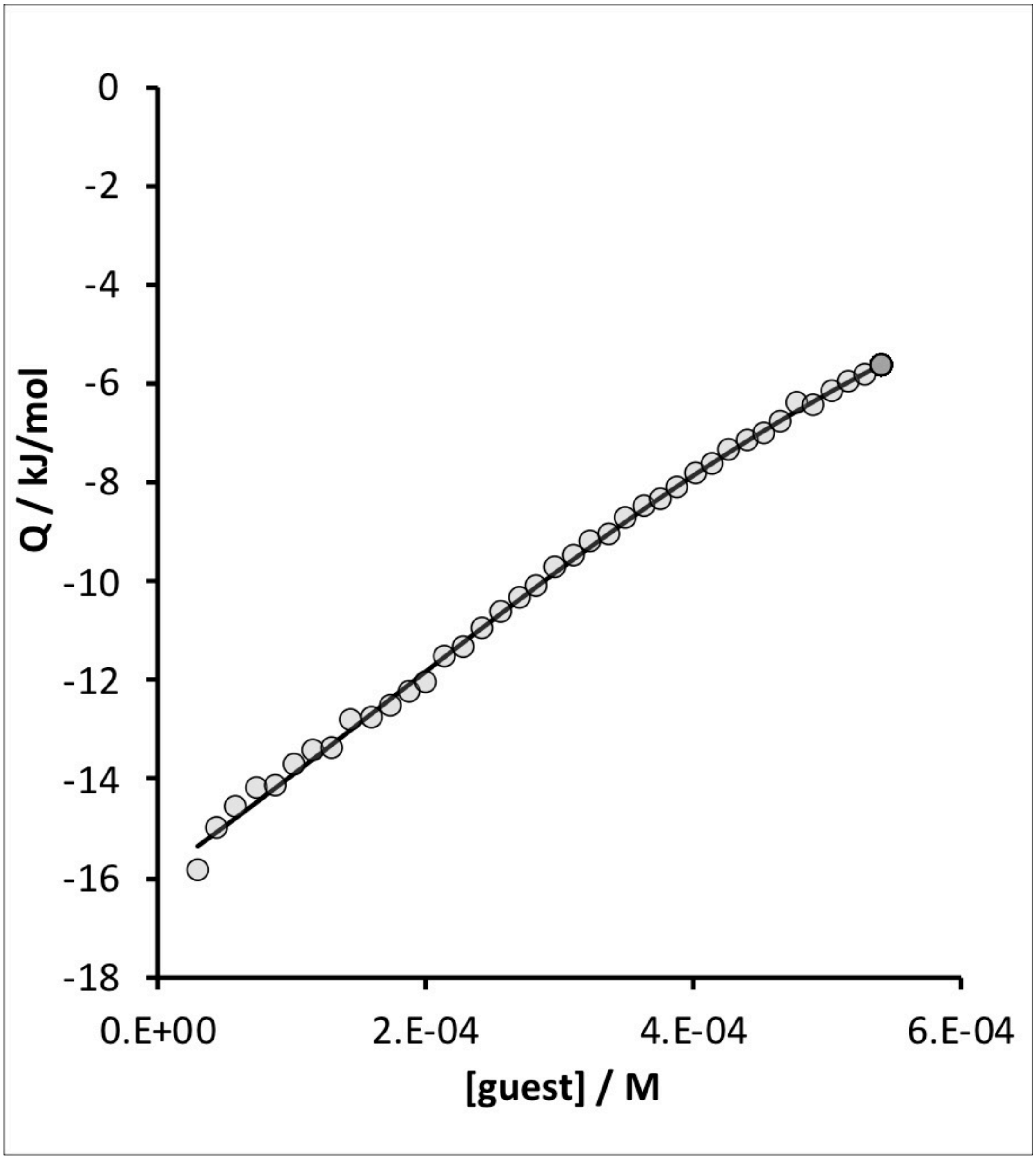

Figure S110. ITC titration of **AAD** (3.0 mM in the syringe) into **DDD** (0.66 mM in the cell) in toluene at 298 K. The line of best fit to an isotherm that allows for formation of the 1:1 complex as well as dimerization of the guest corresponds to K = 2,600 M^{-1} and $\Delta H°$ = -40 kJ mol^{-1}.

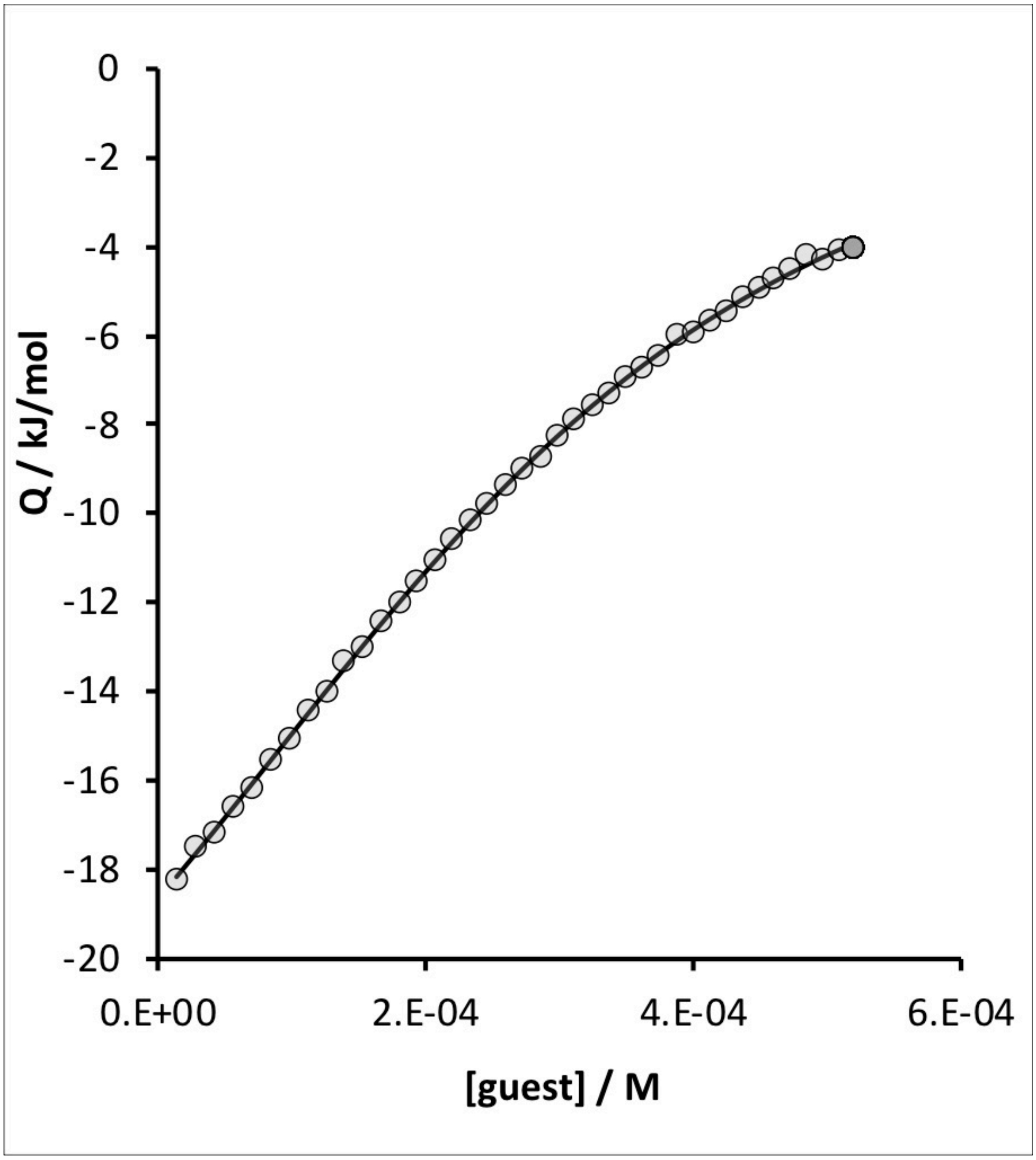

Figure S111. ITC titration of **ADA** (2.9 mM in the syringe) into **DDD** (0.66 mM in the cell) in toluene at 298 K. The line of best fit to an isotherm that allows for formation of the 1:1 complex as well as dimerization of the guest corresponds to K = 4,700 M^{-1} and $\Delta H°$ = -44 kJ mol^{-1}.

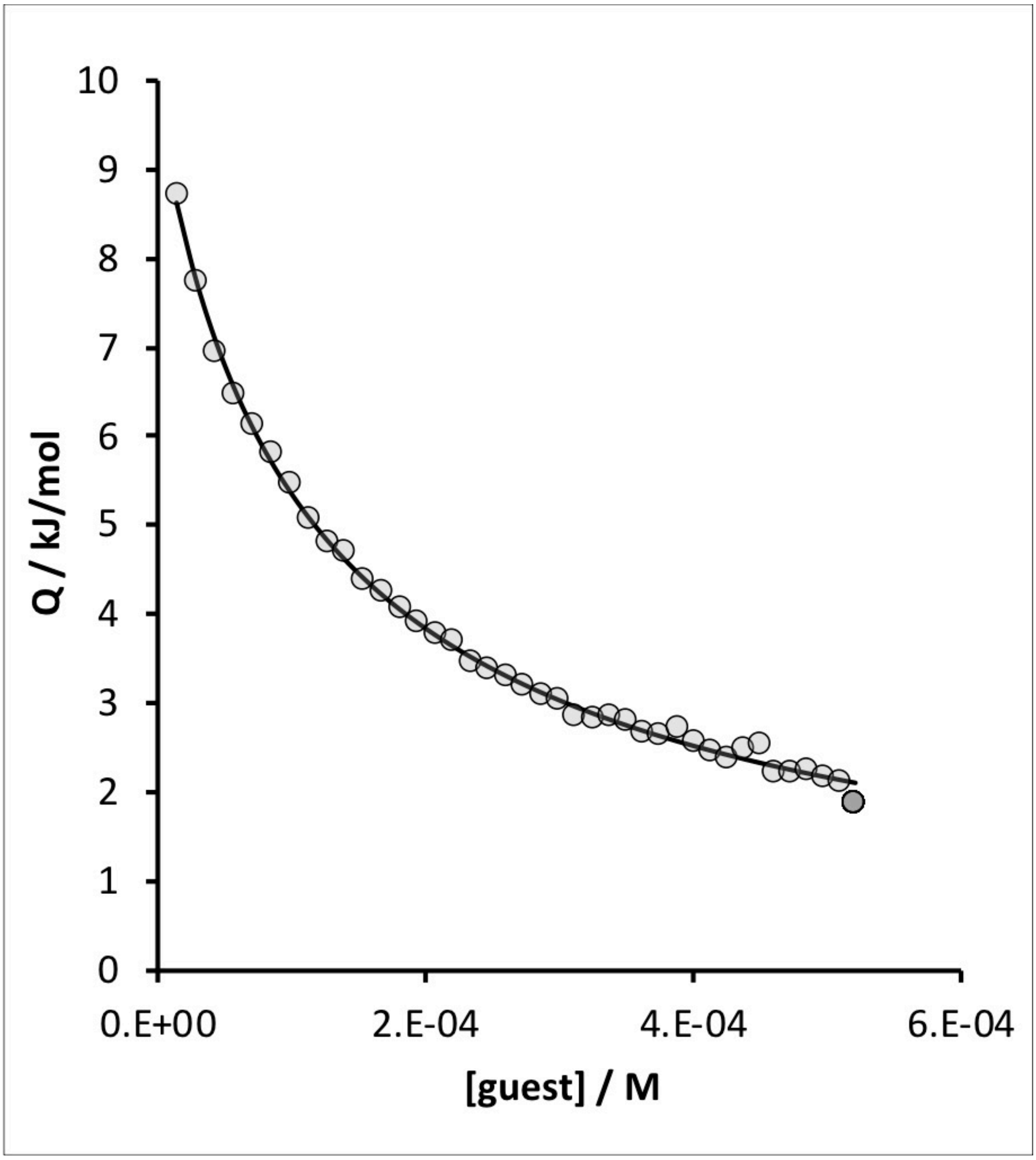

Figure S112. ITC titration of **DAD** (2.9 mM in the syringe) into **DDD** (0.30 mM in the cell) in toluene at 298 K. The line of best fit to an isotherm that only allows for dimerization of the guest with a self-association constant of 1,700 M^{-1} and $\Delta H°$ = -23 kJ mol^{-1}.

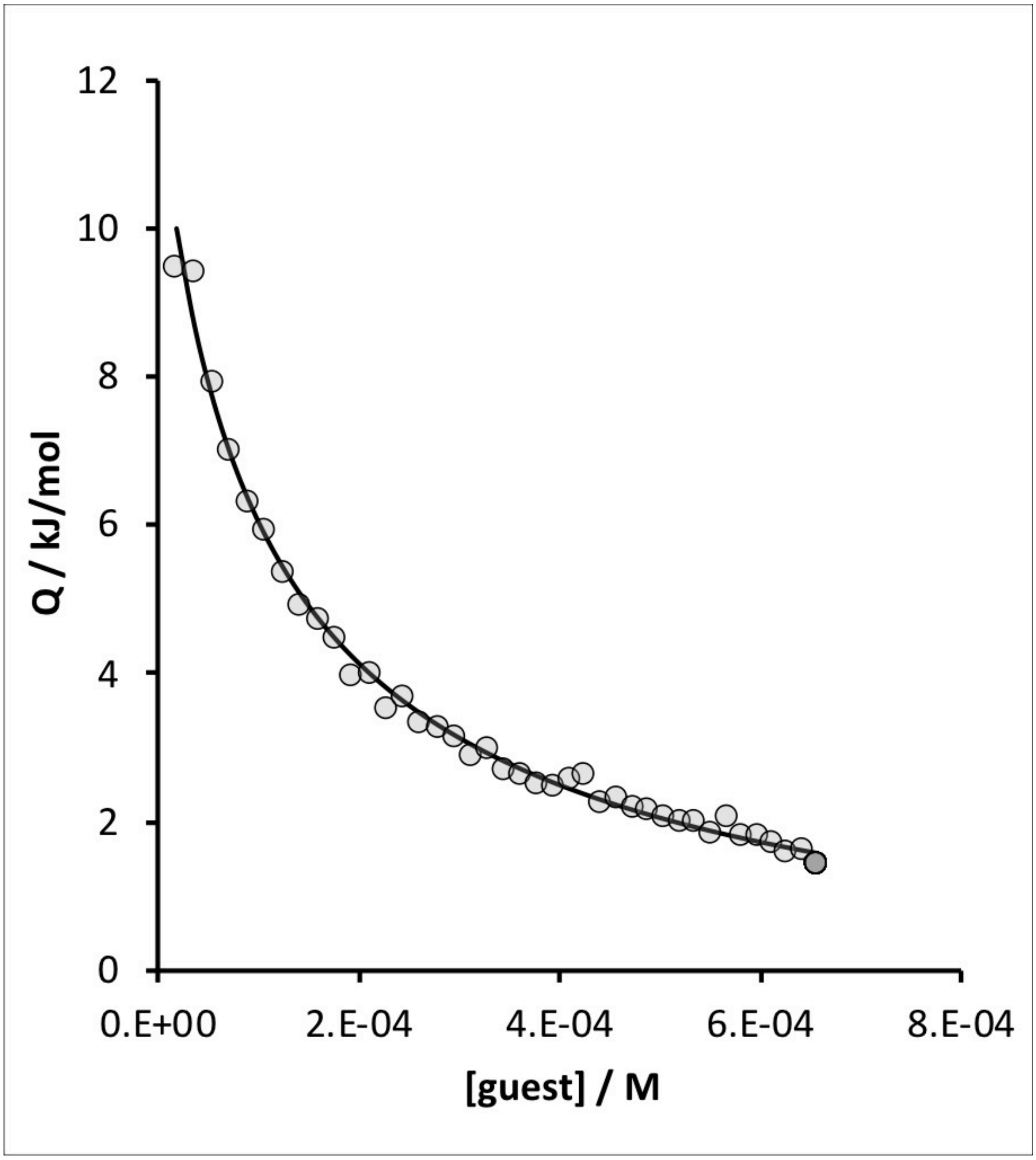

Figure S113. ITC titration of **DDA** (3.7 mM in the syringe) into **DDD** (0.37 mM in the cell) in toluene at 298 K. The line of best fit to an isotherm that only allows for dimerization of the guest with a self-association constant of 1,900 M^{-1} and $\Delta H°$ = -28 kJ mol^{-1}.

Table S1. Summary of thermodynamic parameters measured by ITC titration experiments in toluene at 298 K for all pairwise combinations of 3-mers.[a]

	Complex	log*K* / M^{-1}	$\Delta G°$ kJ mol^{-1}	$\Delta H°$ kJ mol^{-1}	$\Delta S°$ J K^{-1} mol^{-1}
complementary	**AAA•DDD**	5.2	-29.5	-57	-92
	**AAD•ADD**	4.2	-23.9	-45	-71
	**ADA•DAD**	4.3	-24.6	-50	-85
1 mismatch	**ADA•AAD**	3.6	-20.5	-38	-59
	**DAD•ADD**	3.5	-19.8	-34	-48
	**AAA•ADD**	3.6	-20.6	-42	-72
	**AAA•DAD**	3.8	-22.0	-40	-60
	**DDD•AAD**	3.4	-19.5	-40	-69
	**DDD•ADA**	3.7	-21.0	-44	-77
2 mismatches	**ADA•ADD**	4.3	-24.3	-48	-80
	**DAD•AAD**	4.1	-23.7	-45	-71
	**AAA•ADA**[b]	-	-	-	-
	**AAA•AAD**[b]	-	-	-	-
	**DDD•DAD**[b]	-	-	-	-
	**DDD•ADD**[b]	-	-	-	-

[a] Errors based on repeat experiments are 0.1 in log*K*, 0.5 kJ mol^{-1} in $\Delta G°$, 5 kJ mol^{-1} in $\Delta H°$, and 20 J K^{-1} mol^{-1} in $\Delta S°$. [b] For these titrations, dimerization of the guest is more favorable than formation of the host•guest complex, so there is insufficient complex formed to characterize the thermodynamic properties, and the ITC data fit well to a model that only allows for dimerization of the guest.
